# Ethnic differences in the indirect effects of the COVID-19 pandemic on clinical monitoring and hospitalisations for non-COVID conditions in England: a population-based, observational cohort study using the OpenSAFELY platform

**DOI:** 10.1016/j.eclinm.2023.102077

**Published:** 2023-06-29

**Authors:** Ruth E. Costello, John Tazare, Dominik Piehlmaier, Emily Herrett, Edward P.K. Parker, Bang Zheng, Kathryn E. Mansfield, Alasdair D. Henderson, Helena Carreira, Patrick Bidulka, Angel Y.S. Wong, Charlotte Warren-Gash, Joseph F. Hayes, Jennifer K. Quint, Brian MacKenna, Amir Mehrkar, Rosalind M. Eggo, Srinivasa Vittal Katikireddi, Laurie Tomlinson, Sinéad M. Langan, Rohini Mathur, LH&W NCS (or CONVALESCENCE) Collaborative, Nishi Chaturvedi, Chloe Park, Alisia Carnemolla, Dylan Williams, Anika Knueppel, Andy Boyd, Emma L. Turner, Katharine M. Evans, Richard Thomas, Samantha Berman, Stela McLachlan, Matthew Crane, Rebecca Whitehorn, Jacqui Oakley, Diane Foster, Hannah Woodward, Kirsteen C. Campbell, Nicholas Timpson, Alex Kwong, Ana Goncalves Soares, Gareth Griffith, Renin Toms, Louise Jones, Herbert Annie, Ruth Mitchell, Tom Palmer, Jonathan Sterne, Venexia Walker, Lizzie Huntley, Laura Fox, Rachel Denholm, Rochelle Knight, Kate Northstone, Arun Kanagaratnam, Elsie Horne, Harriet Forbes, Teri North, Kurt Taylor, Marwa A.L. Arab, Scott Walker, Jose I.C. Coronado, Arun S. Karthikeyan, George Ploubidis, Bettina Moltrecht, Charlotte Booth, Sam Parsons, Bozena Wielgoszewska, Charis Bridger-Staatz, Claire Steves, Ellen Thompson, Paz Garcia, Nathan Cheetham, Ruth Bowyer, Maxim Freydin, Amy Roberts, Ben Goldacre, Alex Walker, Jess Morley, William Hulme, Linda Nab, Louis Fisher, Brian MacKenna, Colm Andrews, Helen Curtis, Lisa Hopcroft, Amelia Green, Praveetha Patalay, Jane Maddock, Kishan Patel, Jean Stafford, Wels Jacques, Kate Tilling, John Macleod, Eoin McElroy, Anoop Shah, Richard Silverwood, Spiros Denaxas, Robin Flaig, Daniel McCartney, Archie Campbell, Laurie Tomlinson, John Tazare, Bang Zheng, Liam Smeeth, Emily Herrett, Thomas Cowling, Kate Mansfield, Ruth E. Costello, Kevin Wang, Kathryn Mansfield, Viyaasan Mahalingasivam, Ian Douglas, Sinead Langan, Sinead Brophy, Michael Parker, Jonathan Kennedy, Rosie McEachan, John Wright, Kathryn Willan, Ellena Badrick, Gillian Santorelli, Tiffany Yang, Bo Hou, Andrew Steptoe, Di Gessa Giorgio, Jingmin Zhu, Paola Zaninotto, Angela Wood, Genevieve Cezard, Samantha Ip, Tom Bolton, Alexia Sampri, Elena Rafeti, Fatima Almaghrabi, Aziz Sheikh, Syed A. Shah, Vittal Katikireddi, Richard Shaw, Olivia Hamilton, Michael Green, Theocharis Kromydas, Daniel Kopasker, Felix Greaves, Robert Willans, Fiona Glen, Steve Sharp, Alun Hughes, Andrew Wong, Lee Hamill Howes, Alicja Rapala, Lidia Nigrelli, Fintan McArdle, Chelsea Beckford, Betty Raman, Richard Dobson, Amos Folarin, Callum Stewart, Yatharth Ranjan, Jd Carpentieri, Laura Sheard, Chao Fang, Sarah Baz, Andy Gibson, John Kellas, Stefan Neubauer, Stefan Piechnik, Elena Lukaschuk, Laura C. Saunders, James M. Wild, Stephen Smith, Peter Jezzard, Elizabeth Tunnicliffe, Zeena-Britt Sanders, Lucy Finnigan, Vanessa Ferreira, Mark Green, Rebecca Rhead, Milla Kibble, Yinghui Wei, Agnieszka Lemanska, Francisco Perez-Reche, Dominik Piehlmaier, Lucy Teece, Edward Parker, The OpenSAFELY collaborative, Alex J. Walker, Brian MacKenna, Peter Inglesby, Ben Goldacre, Helen J. Curtis, Caroline E. Morton, Jessica Morley, Amir Mehrkar, Sebastian C.J. Bacon, George Hickman, Richard Croker, David Evans, Tom Ward, Nicholas J. DeVito, Louis Fisher, Amelia C.A. Green, Jon Massey, Rebecca M. Smith, William J. Hulme, Simon Davy, Colm D. Andrews, Lisa E.M. Hopcroft, Henry Drysdale, Iain Dillingham, Robin Y. Park, Rose Higgins, Christine Cunningham, Milan Wiedemann, Linda Nab, Steven Maude, Orla Macdonald, Ben F.C. Butler-Cole, Thomas O'Dwyer, Catherine L. Stables, Christopher Wood, Andrew D. Brown, Victoria Speed, Lucy Bridges, Andrea L. Schaffer, Caroline E. Walters, Christopher T. Rentsch, Krishnan Bhaskaran, Anna Schultze, Elizabeth J. Williamson, Helen I. McDonald, Laurie A. Tomlinson, Rohini Mathur, Rosalind M. Eggo, Kevin Wing, Angel Y.S. Wong, John Tazare, Richard Grieve, Daniel J. Grint, Sinead Langan, Kathryn E. Mansfield, Ian J. Douglas, Stephen J.W. Evans, Liam Smeeth, Jemma L. Walker, Viyaasan Mahalingasivam, Harriet Forbes, Thomas E. Cowling, Emily L. Herrett, Ruth E. Costello, Bang Zheng, Edward P.K. Parker, Christopher Bates, Jonathan Cockburn, John Parry, Frank Hester, Sam Harper, Shaun O'Hanlon, Alex Eavis, Richard Jarvis, Dima Avramov, Paul Griffiths, Aaron Fowles, Nasreen Parkes, Brian Nicholson, Rafael Perera, David Harrison, Kamlesh Khunti, Jonathan AC. Sterne, Jennifer Quint

**Affiliations:** a London School of Hygiene and Tropical Medicine, London, UK; b Bennett Institute for Applied Data Science, Nuffield Department of Primary Care Health Sciences, University of Oxford, Oxford, UK; c University of Sussex Business School, Jubilee Building, Brighton, UK; d Division of Psychiatry, University College London, London, UK; e Faculty of Medicine, National Heart & Lung Institute, Imperial College London, London, UK; f MRC/CSO Social & Public Health Sciences Unit, University of Glasgow, Glasgow, UK; g Centre for Primary Care, Wolfson Institute of Population Health, Queen Mary, University of London, London, UK

**Keywords:** Ethnic differences, Pandemic, Healthcare utilisation

## Abstract

**Background:**

The COVID-19 pandemic disrupted healthcare and may have impacted ethnic inequalities in healthcare. We aimed to describe the impact of pandemic-related disruption on ethnic differences in clinical monitoring and hospital admissions for non-COVID conditions in England.

**Methods:**

In this population-based, observational cohort study we used primary care electronic health record data with linkage to hospital episode statistics data and mortality data within OpenSAFELY, a data analytics platform created, with approval of NHS England, to address urgent COVID-19 research questions. We included adults aged 18 years and over registered with a TPP practice between March 1, 2018, and April 30, 2022. We excluded those with missing age, sex, geographic region, or Index of Multiple Deprivation. We grouped ethnicity (exposure), into five categories: White, Asian, Black, Other, and Mixed. We used interrupted time-series regression to estimate ethnic differences in clinical monitoring frequency (blood pressure and Hba1c measurements, chronic obstructive pulmonary disease and asthma annual reviews) before and after March 23, 2020. We used multivariable Cox regression to quantify ethnic differences in hospitalisations related to diabetes, cardiovascular disease, respiratory disease, and mental health before and after March 23, 2020.

**Findings:**

Of 33,510,937 registered with a GP as of 1st January 2020, 19,064,019 were adults, alive and registered for at least 3 months, 3,010,751 met the exclusion criteria and 1,122,912 were missing ethnicity. This resulted in 14,930,356 adults with known ethnicity (92% of sample): 86.6% were White, 7.3% Asian, 2.6% Black, 1.4% Mixed ethnicity, and 2.2% Other ethnicities. Clinical monitoring did not return to pre-pandemic levels for any ethnic group. Ethnic differences were apparent pre-pandemic, except for diabetes monitoring, and remained unchanged, except for blood pressure monitoring in those with mental health conditions where differences narrowed during the pandemic. For those of Black ethnicity, there were seven additional admissions for diabetic ketoacidosis per month during the pandemic, and relative ethnic differences narrowed during the pandemic compared to the White ethnic group (Pre-pandemic hazard ratio (HR): 0.50, 95% confidence interval (CI) 0.41, 0.60, Pandemic HR: 0.75, 95% CI: 0.65, 0.87). There was increased admissions for heart failure during the pandemic for all ethnic groups, though highest in those of White ethnicity (heart failure risk difference: 5.4). Relatively, ethnic differences narrowed for heart failure admission in those of Asian (Pre-pandemic HR 1.56, 95% CI 1.49, 1.64, Pandemic HR 1.24, 95% CI 1.19, 1.29) and Black ethnicity (Pre-pandemic HR 1.41, 95% CI: 1.30, 1.53, Pandemic HR: 1.16, 95% CI 1.09, 1.25) compared with White ethnicity. For other outcomes the pandemic had minimal impact on ethnic differences.

**Interpretation:**

Our study suggests that ethnic differences in clinical monitoring and hospitalisations remained largely unchanged during the pandemic for most conditions. Key exceptions were hospitalisations for diabetic ketoacidosis and heart failure, which warrant further investigation to understand the causes.

**Funding:**

10.13039/100009660LSHTM COVID-19 Response Grant (DONAT15912).

**Research in context**

**Evidence before this study**

We searched MEDLINE from inception to 7th September 2022, for articles published in English, including the title/abstract search terms (healthcare disruption OR indirect impact OR miss∗ diagnos∗ OR delayed diagnos∗ OR service disruption) AND (sars-cov-2 OR covid-19 OR pandemic OR lockdown) AND (ethnic∗). Of the seven studies identified, two broadly investigated the indirect impacts of the pandemic on non-COVID outcomes and reported ethnic differences. However, these two only included data until January 2021 at the latest. Other studies investigated just one disease area such as dementia or diabetes and frequently did not have the power to investigate specific ethnic groups.

**Added value of this study**

This is one of the largest studies to describe how the pandemic impacted ethnic differences in clinical monitoring at primary care and hospital admissions for non-COVID conditions (across four disease areas: cardiovascular disease, diabetes mellitus, respiratory disease and mental health) in England. A study population of nearly 15 million people, allowed the examination of five ethnic groups, and data until April 2022 allowed the evaluation of impacts for a longer period than previous studies. We showed that clinical monitoring had still not returned to pre-pandemic levels even by April 2022. Ethnic differences in clinical monitoring were seen pre-pandemic, though not in diabetes measures, these differences were either not impacted or reduced during the pandemic. We also showed that there were ethnic differences in hospital admissions, for many outcomes the pandemic did not impact these differences but there were some exceptions, in particular for diabetic ketoacidosis admissions in those of Black ethnicity and heart failure admissions for those of Black and Asian ethnicities.

**Implications of all the available evidence**

We found that the pandemic reduced ethnic inequalities for some outcomes (in hospitalisations for diabetic ketoacidosis and heart failure). However, these were driven by greater absolute increases in admissions for Black and Asian groups (diabetic ketoacidosis) and white groups (heart failure), which warrant further investigation to understand the underlying causes.

## Introduction

The COVID-19 pandemic directly impacted healthcare services across the world. This happened to different degrees in different countries, but had not recovered to pre-pandemic levels by the end of 2020.^1^, ^2^, ^3^, ^4^, ^5^ In the UK, primary care contacts and hospital admissions for a range of physical and mental health conditions decreased dramatically during 2020, most notably for anxiety, depression, chronic obstructive pulmonary disease (COPD) and cancer.^6^^,^^7^ COVID-19 disproportionately affects minority ethnic populations in the UK, with a higher risk of adverse COVID-19 outcomes, particularly in those of South Asian ethnicity.^8^ In addition to inequalities in the direct consequences of COVID-19, indirect healthcare consequences of the pandemic may also be unequal.^9^ In England, the pandemic impacted healthcare services differently across ethnic groups during 2020, with greater reductions in scheduled and unscheduled admissions, in those of non-White ethnicity compared with pre-pandemic.^7^^,^^10^ Differences have also been seen within specific disease areas.^11^^,^^12^ For example, there were increases in diabetic ketoacidosis (DKA) admissions for those of non-White ethnicity during the first wave of the pandemic.^12^ However, often studies have lacked sufficient power to compare specific ethnic groups, resulting in ‘White’ vs ‘non-White’ comparisons.^11^^,^^12^ Moreover, few studies have examined the impact of the pandemic on healthcare services beyond 2020.

Clinical monitoring refers to health measurements, such as blood pressure, that take place in primary care. These measures aim to prevent serious illness by identifying disease at an earlier stage and ensure known diseases are well managed.^13^^,^^14^ During the pandemic resources were diverted to COVID-19 related work, resulting in a reduction in monitoring.^15^ In people with diabetes reduced monitoring was associated with increased non-COVID mortality.^16^ This may be true for other diseases. Therefore, we chose to investigate clinical monitoring and hospitalisations within four disease areas (cardiovascular disease (CVD), diabetes mellitus (DM), respiratory disease and mental health), to align with previous work,^6^ and because these disease areas have evidence of ethnic differences in incidence and management.^17^, ^18^, ^19^, ^20^, ^21^ We aimed to determine the impact of the pandemic on ethnic differences in clinical monitoring and hospital admissions for non-COVID related conditions in England between 2020 and 2022.

## Methods

### Study design and data source

We conducted a population-based observational cohort study using OpenSAFELY-TPP, a data analytics platform created on behalf of NHS England to address urgent COVID-19 research questions (https://opensafely.org). Pseudonymised primary care electronic health records (EHR) from primary care software provider TPP, covering approximately 40%, and broadly representative of, the population of England,^22^ were linked to inpatient admissions data from the Hospital Episode Statistics for England Admitted Patient Care dataset (HES-APC) and mortality data from the Office for National Statistics (ONS). Data include pseudonymized data such as coded diagnoses, medications and physiological parameters. No free text data are included.

This study was approved by the Health Research Authority (REC reference 20/LO/0651) and by the LSHTM Ethics Board (reference 21863). See supplementary materials for further information governance details. This manuscript adheres to the RECORD guidelines.

### Study population

The study included adults aged 18 years and over registered with a TPP practice between 1st March 2018 and 30th April 2022, with at least three months of registration prior to study entry (further detail: statistical analysis section). People were excluded if age, sex, geographic region, or Index of Multiple Deprivation (IMD) were missing, as missingness may indicate poor data quality. People were also excluded if their household size was greater than 15, to exclude people living in institutions, e.g. care home residents, who may have different clinical monitoring and hospital admissions patterns compared with the general population. People were followed from the start of the study period until the earliest of death, de-registration from the primary care practice, latest data availability, or the end of the study. Four disease-specific sub-populations were identified (described in the outcomes section).

### Procedures

The primary exposure was self-reported ethnicity defined using SNOMED CT morbidity codes in the primary care record. Where unavailable, information was supplemented with secondary care data.^23^ Ethnic groups were combined into the 2001 census categories, as follows: White (White British, White Irish, other White), Asian (Indian, Pakistani, Bangladeshi, other South Asian), Black (African, Caribbean, other Black), Mixed (White and Asian, White and African, White and Caribbean, other Mixed) and Other (Chinese, Arab, all others).

**Fig. 1:**
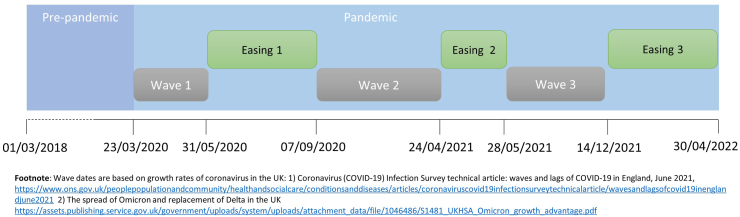
Study time-periods.

Ethnic differences in outcomes were compared: 1) before and after the introduction of lockdown in the UK on 23rd March 2020^24^ (defined as pre-pandemic and pandemic time); and 2) across six time periods during the pandemic (Fig. 1).

Study outcomes included clinical monitoring activities and hospital admissions related to four disease areas: DM, CVD, respiratory disease and mental health (Table 1).

**Table 1:** Outcome definitions and study populations.

	Disease area			
	Diabetes	Cardiovascular disease	Respiratory disease	Mental health
**Clinical monitoring**				
Outcomes measured	HbA1c, blood pressure	Blood pressure	COPD annual review,^1^ asthma annual review^2^	Blood pressure
Study population	People with Type 1 DM or Type 2 DM as coded on primary care record any time prior to study entry	People with coronary heart disease, history of stroke or TIA as coded in primary care record any time prior to study entry	^1^ people aged >40 with a COPD code prior to study entry,^2^ people with an asthma code in primary care record in the 3 years prior to study entry	People with schizophrenia, bipolar disorder and other psychoses as coded in primary care record any time prior to study entry
**Hospital admissions**				
Outcomes measured	Admission with primary reason type 1 DM, type 2 DM or diabetic ketoacidosis	Admission with primary reason MI, stroke, heart failure or VTE	Admission with primary reason asthma exacerbation in those with asthma^1^ or COPD exacerbation in those with COPD^2^	Admission with primary reason depression or anxiety
Study population	People with type 1 DM or type 2 DM code in primary care record any time prior to study entry	General adult population	^1^ age >40 with COPD code prior to study entry,^2^ asthma code in primary care record in the 3 years prior to study entry	General adult population

HbA1c: Haemoglobin A1c, COPD: chronic obstructive pulmonary disease, DM: diabetes mellitus, TIA: transient ischaemic attack, MI: myocardial infarction, VTE: venous thromboembolism.

Demographic characteristics included age, sex, sustainability and transformation partnership (STP) region (NHS administrative geographical area), urban-rural classifier, deprivation, and shielding status. Deprivation was measured using quintiles of IMD based on a person's postcode. People classed as extremely clinically vulnerable and therefore advised to shield were identified through SNOMED CT codes.^25^

### Statistical methods

The characteristics of the overall cohort on 1st January 2019, 2020 and 2021 were described by ethnic group. Two methods were used to estimate the impact of the pandemic on ethnic differences in outcomes: 1) interrupted time-series analysis; and 2) survival analysis.

We calculated monthly crude rates of each clinical monitoring outcome, stratified by ethnicity. To measure monthly crude rates, study eligibility was assessed each month and individuals were included in the denominator for the whole month if they were eligible on the first of the month. Outcomes were counted once each month, but people could appear in multiple months if they had repeated records of the outcome in different months.

Monthly rates of each outcome were modelled in an ordinary least squares regression model with Newey–West heteroskedasticity-consistent standard errors and one lag to account for autocorrelation (Further details in supplementary materials).^26^, ^27^, ^28^, ^29^ The interruption was set at 23rd March 2020 (introduction of lockdown restrictions). To account for seasonal variation in outcome rates, season was included in the model as a four-level categorical variable: March–May, June–August, September–November, December–February. The rates were modelled with an interaction term for ethnicity to assess whether the ethnic patterning of outcomes changed from pre-pandemic to pandemic time.

Due to small numbers (n < 10 per ethnic group in any single month) ethnic differences in hospital admissions could not be analysed using interrupted time-series analysis.

We estimated hospital admission rates, by ethnic group, across eight pre-defined time periods (Fig. 1). People who met the inclusion criteria at the start of each time-period were identified and followed from the start of the time-period until the earliest of death, de-registration from primary care practice, latest data availability, the end of the time-period, or until the first event for each hospital admission outcome.

We used Cox proportional hazards regression to estimate ethnic differences in time to first non-COVID-19 related hospital admission within each time period. We examined the proportional hazards assumptions using graphical methods and tests based on Schoenfeld residuals. We initially adjusted for age and sex, and then additionally for potential confounders: urban-rural classifier, deprivation, and shielding status. White ethnicity was the reference group and all models were clustered by STP region. To quantify the relative difference for each ethnic group, the ratio of HRs was calculated as the HR for each pandemic time period divided by the pre-pandemic HR. A ratio below one indicates a lower relative hazard of admission during the pandemic compared with pre-pandemic, a ratio above one indicates a higher relative hazard of admission during the pandemic time period. As a sensitivity analysis we also ran the models with each non-White ethnic group as the reference to determine the pairwise comparisons.

We used Python 3.8 for data management, Stata 17 for statistical analyses and R version 4.2.1 for HR plots. All code is shared for review and re-use under open licences at GitHubcom/OpenSAFELY. Code for data management and analysis, as well as codelists is archived online at https://github.com/opensafely/covid-collateral-research.

### Role of the funding source

The funders of the study had no involvement in the study design, data analysis, interpretation, writing of the paper and the decision to submit for publication. REC, JT, DP, EH, BZ, EPKP, BM, RME and RM had access to the data. All authors contributed to and approved the final manuscript, and accept responsibility to submit for publication.

## Results

As of 1st January 2020, 16,053,268 people met the inclusion criteria (Supplementary materials). Ethnicity was missing for 1,122,912 (7%). Of those with known ethnicity, 12,926,485 (86.6%) were White, 1,096,398 (7.3%) were Asian, 381,441 (2.6%) were Black, 201,747 (1.4%) were of Mixed ethnicity, and 324,285 (2.2%) were of Other ethnicities. Compared with all other ethnic groups, those of White ethnicity were older, and lived in less deprived and more rural locations (Table 2). CVD was the most common comorbidity in those of White ethnicity (11.4%) while Type 2 DM was the most common comorbidity in those of Asian (12.6%) and Black (9.1%) ethnicities. Characteristics as of 1st January 2019 and 2021 were similar (Supplementary materials).

**Table 2:** Baseline characteristics on 1st January 2020 by ethnic group.

	All	White	Asian	Black	Mixed	Other	Missing
	N = 16,053,268	N = 12,926,485	N = 1,096,398	N = 381,441	N = 201,747	N = 324,285	N = 1,122,912
Age category							
18–40 years	5,773,317 (36.0%)	4,227,311 (32.7%)	546,311 (49.8%)	169,643 (44.5%)	117,724 (58.4%)	185,369 (57.2%)	526,959 (46.9%)
41–60 years	5,284,691 (32.9%)	4,207,590 (32.6%)	382,006 (34.8%)	158,168 (41.5%)	63,377 (31.4%)	99,890 (30.8%)	373,660 (33.3%)
61–80 years	3,983,115 (24.8%)	3,554,397 (27.5%)	142,755 (13.0%)	43,560 (11.4%)	17,691 (8.8%)	33,639 (10.4%)	191,073 (17.0%)
>80 years	1,012,145 (6.3%)	937,187 (7.3%)	25,326 (2.3%)	10,070 (2.6%)	2955 (1.5%)	5387 (1.7%)	31,220 (2.8%)
Sex							
Male	7,921,166 (49.3%)	6,210,432 (48.0%)	558,938 (51.0%)	188,610 (49.4%)	96,550 (47.9%)	163,616 (50.5%)	703,020 (62.6%)
IMD							
1 (most deprived)	3,174,183 (19.8%)	2,320,768 (18.0%)	356,411 (32.5%)	155,272 (40.7%)	58,165 (28.8%)	82,342 (25.4%)	201,225 (17.9%)
2	3,232,330 (20.1%)	2,478,666 (19.2%)	309,455 (28.2%)	98,646 (25.9%)	47,107 (23.3%)	79,919 (24.6%)	218,537 (19.5%)
3	3,475,747 (21.7%)	2,848,543 (22.0%)	212,185 (19.4%)	64,707 (17.0%)	40,553 (20.1%)	67,290 (20.8%)	242,469 (21.6%)
4	3,240,603 (20.2%)	2,755,299 (21.3%)	126,438 (11.5%)	38,687 (10.1%)	31,583 (15.7%)	53,188 (16.4%)	235,408 (21.0%)
5 (least deprived)	2,930,405 (18.3%)	2,523,209 (19.5%)	91,909 (8.4%)	24,129 (6.3%)	24,339 (12.1%)	41,546 (12.8%)	225,273 (20.1%)
Type 1 diabetes							
Yes	113,640 (0.7%)	99,350 (0.8%)	7163 (0.7%)	2950 (0.8%)	1160 (0.6%)	1225 (0.4%)	1792 (0.2%)
Type 2 diabetes							
Yes	1,158,128 (7.2%)	928,618 (7.2%)	138,022 (12.6%)	34,800 (9.1%)	10,831 (5.4%)	15,239 (4.7%)	30,618 (2.7%)
Asthma^a^							
Yes	1,399,693 (8.7%)	1,215,301 (9.4%)	83,180 (7.6%)	25,166 (6.6%)	16,664 (8.3%)	13,551 (4.2%)	45,831 (4.1%)
COPD^b^							
Yes	516,877 (3.2%)	490,897 (3.8%)	10,066 (0.9%)	3205 (0.8%)	1728 (0.9%)	2339 (0.7%)	8642 (0.8%)
CVD^c^							
Yes	1,835,952 (11.4%)	1,686,486 (13.0%)	69,528 (6.3%)	21,660 (5.7%)	9170 (4.5%)	12,608 (3.9%)	36,500 (3.3%)
Serious mental illness^d^							
Yes	194,273 (1.2%)	160,501 (1.2%)	13,824 (1.3%)	8162 (2.1%)	3793 (1.9%)	3119 (1.0%)	4874 (0.4%)

a Asthma code in primary care record in the 3 years prior to study entry.

b Age >40 with COPD code prior to study entry.

c Coronary heart disease, history of stroke or TIA as coded in primary care record any time prior to study entry.

d Schizophrenia, bipolar disorder and other psychoses as coded in primary care record any time prior to study entry.

### Clinical monitoring

**Fig. 2:**
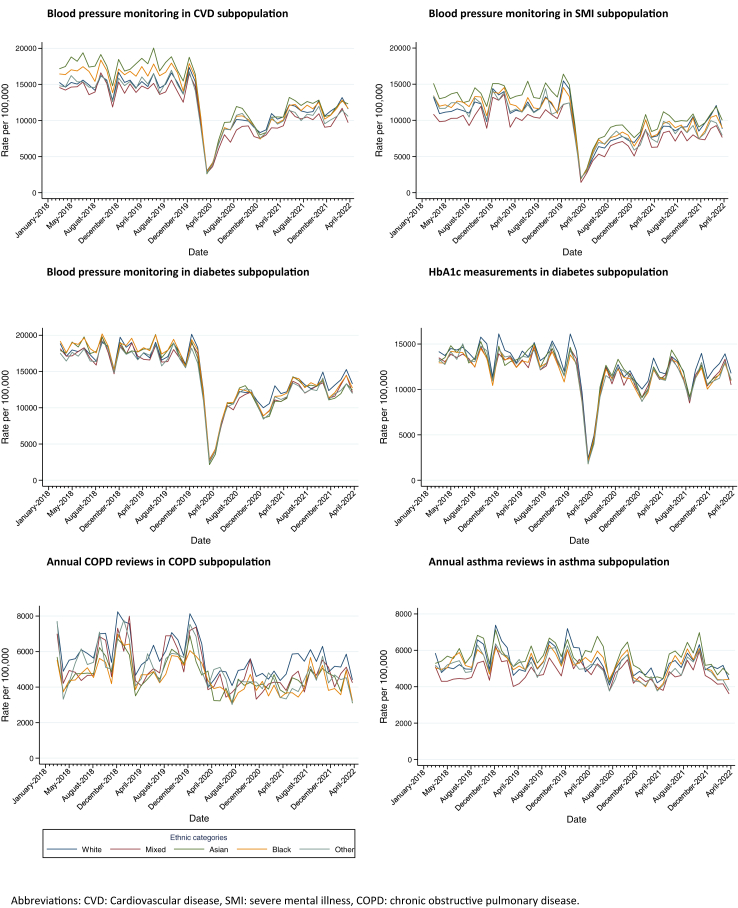
Monthly rates of clinical monitoring by ethnic group.

The monthly frequency of all clinical monitoring outcomes decreased after the start of the pandemic. The change between pre-pandemic and pandemic time was most pronounced for blood pressure monitoring across all disease areas and smallest for asthma annual reviews. Monitoring did not recover completely by April 2022 for most outcomes, with HbA1c monitoring recovering the most and asthma annual reviews remaining fairly constant at the lower rate (Fig. 2 and supplementary figure for the rate change).

### Pandemic impact on ethnic differences in clinical monitoring

Interrupted time-series analysis indicated that, across the whole study period, ethnic differences in HbA1c monitoring were very small amongst people with diabetes. Amongst people with asthma and COPD, compared with people of White ethnicity, people of Mixed ethnicity received fewer asthma reviews and people of all minority ethnic groups received fewer COPD annual reviews. Blood pressure monitoring varied depending on the disease group. While blood pressure monitoring did not vary by ethnicity in those with diabetes, in those with CVD, blood pressure monitoring was lowest in those of Mixed ethnicity and highest in those of Asian and Black ethnicities. In those with serious mental illness, blood pressure monitoring was lowest in those of Mixed ethnicity and highest in those of Asian ethnicity (Fig. 2).

Ethnic patterning of clinical monitoring remained unchanged between the pre-pandemic and pandemic periods for all outcomes, except for blood pressure monitoring in those with severe mental illness, where those of Asian ethnicity had fewer blood pressure measurements after the start of the pandemic. For blood pressure monitoring in those with CVD, though not significant in the time-series analysis, we noted that differences narrowed between all ethnic groups during the pandemic, though remained below pre-pandemic levels for all groups (Fig. 2).

### Rates of hospital admissions

Compared to pre-pandemic time, rates of hospital admissions increased for stroke and heart failure in the White ethnic group, with five additional admissions per month during the pandemic (stroke rate difference (RD) 5.2, heart failure RD: 5.4). For other ethnic groups and other CVD outcomes, differences in hospital admission rates were small (RD ≤ 3). Amongst people with diabetes, DKA admissions increased during the pandemic, most notably for people of Black ethnicity, with seven additional admissions per month (RD: 7.26). Hospital admissions for anxiety and depression were low for all ethnic groups (<1 per month) with less than one additional admission per month during the pandemic compared to pre-pandemic. Asthma and COPD hospital admissions decreased during the pandemic (Supplementary materials).

### Pandemic impact on ethnic differences in hospital admissions

#### CVD

Prior to the pandemic, compared with those of White ethnicity, the age and sex adjusted hazard of stroke admission was higher in those of Black ethnicity. Hazards of VTE admission were lower in all minority ethnic groups compared with those of White ethnicity. Hazards of heart failure admission were higher in those of Black and Asian ethnicity, and similar or lower in Mixed and Other ethnicities compared to White. Hazards of MI related admissions were higher in those of Asian ethnicity, and lower in all other minority ethnic groups, compared to those of White ethnicity.

**Fig. 3:**
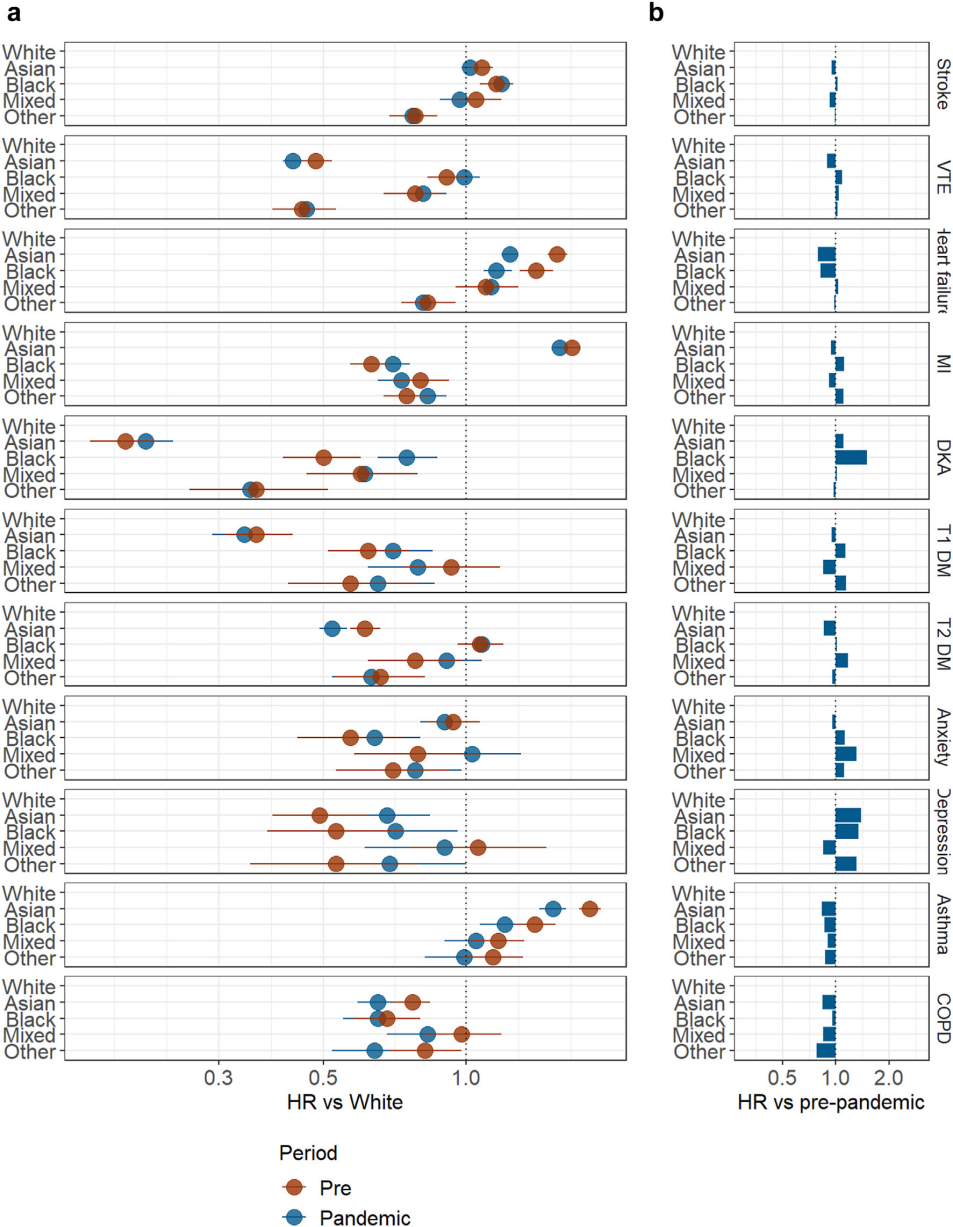
a) Age and sex adjusted hazard ratios pre- (red) and pandemic (blue) for each non-White ethnic group versus White, where dots represent hazard ratios and the error bars represent 95% confidence intervals b) Ratio of hazard ratios for each ethnic group.

While the ethnic patterning of hospital admissions for stroke, VTE, and MI remained unchanged between the pre-pandemic and pandemic periods, ethnic differences in heart failure admissions narrowed during the pandemic in those of Asian (Pre-pandemic HR 1.56, 95% CI 1.49, 1.64, Pandemic HR 1.24, 95% CI 1.19, 1.29) and Black ethnicity (Pre-pandemic HR 1.41, 95% CI: 1.30, 1.53, Pandemic HR: 1.16, 95% CI 1.09, 1.25) (Fig. 3, Supplementary materials).

When comparing across wave and easing periods, ethnic differences for stroke and VTE remained small and consistent across the periods. The relative hazard of heart failure admission was lower for those of Black and Asian ethnicity in all pandemic waves compared with pre-pandemic. The hazard of MI admission in those of Black ethnicity was lower in Wave 1 compared with the pre-pandemic period and higher than pre-pandemic in all other waves (Supplementary materials).

#### Diabetes

In age and sex adjusted analysis, all ethnic groups had a lower hazard of each outcome prior to and during the pandemic compared with White ethnicity. For DKA, the hazard of admission was higher during the pandemic for those of Black ethnicity (Pre-pandemic HR: 0.50, 95% CI 0.41, 0.60, Pandemic HR: 0.75, 95% CI: 0.65, 0.87), meaning differences between Black and White groups narrowed during the pandemic. For Type 2 DM, the hazard of admission decreased for those of Asian ethnicity (Pre-pandemic HR 0.61, 95% CI 0.57, 0.66, Pandemic HR 0.52, 95% CI 0.49, 0.56), meaning differences between Asian and White groups widened.

When the pandemic period was split into wave and easing periods, the increase in DKA admissions in those of Black ethnicity was seen across all waves. There was a relative decline in admissions for Type 1 and Type 2 DM in those of Asian ethnicity across Waves 1 (23rd March 2020–30 May 2020), easing 1 (31 May 2020–6 September 2020), and Wave 2 (7 September 2020–23 April 2021) in particular (Fig. S3, Supplementary materials).

#### Respiratory

For COPD, age and sex adjusted hazard of admission was lower, or similar, in all ethnic groups compared with White before and during the pandemic. Ethnic differences widened during the pandemic, most notably for Asian groups relative to White (Pre-pandemic HR 0.77, 95% CI 0.70, 0.84, pandemic HR 0.65, 95% CI 0.59, 0.71).

For asthma, the Asian, Black and Mixed ethnic groups had a higher age and sex adjusted hazard of admission compared with those of White ethnicity prior to the pandemic. When comparing HRs between pre-pandemic and pandemic time periods, ethnic differences attenuated for all ethnic groups relative to those of White ethnicity, the biggest reduction was seen in those of Asian ethnicity (Pre-pandemic HR 1.83, 95% CI 1.74, 1.93, pandemic HR 1.53 (95% CI 1.43, 1.63)).

For both COPD and asthma admissions, the same patterns were seen across all pandemic time periods but differences reduced during later periods (Fig. S3, Supplementary materials).

#### Mental health

All minority ethnic groups had lower or similar age and sex adjusted hazard of admission for both anxiety and depression, compared to those of White ethnicity, both before and during the pandemic. When comparing HRs between pre-pandemic and pandemic time periods for each ethnic group, differences between White and Mixed ethnicity were removed for anxiety related admissions during the pandemic (Pre-pandemic HR 0.79, 95% CI: 0.58, 1.07, Pandemic HR 1.03, 95% CI 0.81, 1.31). Ethnic differences in depression related admissions narrowed for those of Asian and Black relative to those of White ethnicity during the pandemic, (Asian ethnicity: Pre-pandemic HR 0.49, 95% CI 0.39, 0.62, Pandemic HR 0.68, 95% CI 0.55, 0.84, Black ethnicity: Pre-pandemic HR 0.53, 95% CI 0.38, 0.74, Pandemic HR 0.71, 95% CI 0.52, 0.96) (Fig. S3, Supplementary materials).

For all hospital admission outcomes, additional adjustment for urban-rural classifier, deprivation, and shielding status made minimal difference to results (Supplementary materials).

When reviewing the proportional hazards across all models we found that due to the very large sample size, some very minor and clinically unimportant differences in proportional hazard ratios demonstrated low P-values. The Schoenfeld residual plots showed extreme values but did not indicate violation of the proportional hazard's assumption (supplementary materials). We recommend, the HRs we present should be interpreted as a weighted average of the time-varying HRs within each time period.^30^

#### Sensitivity analyses

The models with non-White reference groups largely showed similar results and supports our findings (Supplementary materials).

## Discussion

We found that, as of April 2022, primary care clinical monitoring across a range of conditions had still not returned to pre-pandemic levels. We saw ethnic differences in CVD, respiratory and mental health clinical monitoring, though there were some positive findings; ethnic differences in monitoring for people with diabetes were small, and although ethnic differences were apparent for other disease groups, these differences either remained the same, or were narrowed during the pandemic. In terms of hospital admissions, after accounting for age and sex, many ethnic differences remained unchanged during the pandemic, though there were some notable exceptions: ethnic differences attenuated during the pandemic for DKA admissions in the Black ethnic group and for heart failure admission for those of Asian and Black ethnicities relative to White. However there were different mechanisms for these changes, for DKA admissions there was an absolute increase in rates in those of Black ethnicity that was not seen in other ethnic groups. For heart failure, there was an absolute increase in rates for all ethnic groups, but the biggest increase was seen in those of White ethnicity, narrowing relative differences.

Previous studies have shown that healthcare services in the UK, both broadly and within specific disease areas, were disrupted until the end of 2020.^6^^,^^7^^,^^31^, ^32^, ^33^, ^34^, ^35^ We show that, for the clinical monitoring measures we used, disruption is still ongoing in 2022. We do not know if this is due to the health service being stretched^36^ or people feeling reluctant to visit healthcare services, particularly if they are vulnerable. Although missed monitoring may represent appropriate reprioritisation of services,^37^ it may also represent missed opportunities for early diagnosis and prevention of serious outcomes. It is important to understand the characteristics of groups receiving less frequent care to determine whether these groups require targeted intervention. For example, in those with diabetes, reductions in routine diabetes monitoring have been associated with excess diabetes-related mortality.^16^

We showed that those of Black ethnicity had lower hazards of DKA admissions during both periods relative to those of White ethnicity, however the HR attenuated during the pandemic, indicating higher hazard of admission during the pandemic both in relative and absolute terms. Previous studies had shown ethnic differences in admissions for DKA in 2020, with increased admissions in non-White ethnic groups in the UK and non-Hispanic Black ethnicities in the US.^12^^,^^38^ We have not explored the reasons for the increase seen, although other studies have suggested it could be due to COVID-19 infection, which is known to disproportionately affect ethnic minorities, or worsening glycaemic control due to social restrictions.^39^

For those of Asian and Black ethnicities, we saw that the relative hazard of heart failure admission was attenuated during the pandemic, indicating a lower hazard of admission. This was similar to a study in the USA where a higher proportion of heart failure admissions were for those of non-Hispanic White ethnicity.^40^ Other studies in the general population have shown reductions in heart failure admissions during the pandemic (until mid-2020) compared to pre-pandemic.^2^^,^^41^^,^^42^ We saw similar heart failure admission rates during Wave 1 compared to pre-pandemic. Reductions in heart failure admissions could represent missed opportunities for preventive care, as studies have found increases in heart failure mortality alongside decreases in admissions for heart failure, particularly where there were large reductions in admissions.^41^^,^^43^ Similar reductions in admissions due to increased mortality are possible for all outcomes where admissions were reduced during the pandemic. Alternatively, reductions could indicate lower severity of these conditions, or these individuals may have been admitted with COVID as a primary diagnosis.

There was a higher hazard of admission for depression in those of Asian and Black ethnicities, compared with White ethnicity, although in absolute terms the number of additional admissions was small. A study in the UK and US reported higher levels of depression and anxiety in ethnic minorities during the pandemic.^44^ Studies using data from a household survey in the UK in April 2020 found the highest levels of psychological distress in those of Asian ethnicity.^44^^,^^45^ It is possible that this translated to hospital admissions later in the pandemic. In addition, COVID-19 itself has been associated with mental health symptoms.^46^ As COVID-19 disproportionately affects ethnic minorities this could explain the increase in admissions relative to those of White ethnicity. Hospital admissions data only capture the most serious mental health cases, therefore exploration of other types of data (such as primary care records, patient-reported and mental health services data) may provide more insight.

A strength of this study is that we could investigate hospital admissions during periods of strict and relaxed restrictions into 2022. Broadly we saw that admissions were lower during Wave 1, which would be expected given the tight restrictions and uncertainty at that time. Many of the ethnic differences that were seen were consistent across all waves, though incidence rates were often highest during periods of easing restrictions, particularly the second period of easing (April 2021–May 2021). Further strengths of this study were the study population size, with 16 million people included. This allowed us to identify differences between individual ethnic groups rather than combining all ethnic minorities into one group. There were limitations: we were reliant on coding to identify exposures and outcomes, therefore misclassification is possible. If specific ethnic groups were less likely to present at healthcare services there could be differential misclassification for outcomes, although this may have less effect on hospitalisation outcomes due to their serious nature. Ethnicity information was missing for a small proportion of the population, we also did not have the power to investigate subcategories within the five ethnic groups. Further to this the Other ethnic group was quite large and heterogeneous so difficult to make conclusions about. We only examined a small number of clinical monitoring measures within each disease area, though these measures are part of the Quality and Outcomes Framework^14^ followed by GPs across the UK, they may not be representative of all clinical monitoring. We acknowledge that the use of Newey–West estimators may result in over-reject the null hypothesis and inflate the type I error rate. However we used an autoregressive approach to improve the performance of the models.^47^ This study was primarily descriptive, therefore we did not explore potential explanatory factors for the differences seen or the impact of COVID-19. As our study was descriptive we did not adjust the Cox model 95% confidence intervals for potential multiplicity, therefore there is potential for Type 1 error where the null hypothesis that there are no ethnic differences in outcomes, is incorrectly rejected. We also examined clinical monitoring and hospitalisations separately therefore could not examine the influence of clinical monitoring adherence on hospitalisations.

The mechanisms underlying ethnic differences observed in our study are likely to be complex and disease specific, including genetic risk factors, differential exposure and vulnerability to COVID-19 and potential inequalities in health seeking behaviour and access to healthcare.^9^ Further research into the causal mechanisms, within disease areas where ethnic differences have been seen, is warranted, particularly where there is evidence of similar trends in other countries, which could indicate a universal root cause.

In conclusion, these results demonstrate a consistent patterning of ethnic differences in relation to primary care monitoring of chronic conditions and hospital admissions in England, that has persisted over the period of the COVID-19 pandemic. It is critical to understand the causes of some of the differences identified and whether they represent inequities in access to or quality of care.

## Contributors

REC, JT, DP, EH, HC, PB, JKQ, LT, SML & RM were involved in the development of the study. REC, JT, DP, EH, BZ, EPKP, BM, RME and RM had access to the data. REC, JT, EH, BZ, EPKP and RM verified the underlying data. REC, JT, DP, EH and RM were responsible for data management and statistical analysis. REC and RM wrote the first draft of the manuscript. All authors contributed to and approved the final manuscript, and accept responsibility to submit for publication.

## Data sharing statement

All data were linked, stored and analysed securely within the OpenSAFELY platform https://opensafely.org/. All code is shared openly for review and re-use under MIT open license (https://github.com/opensafely/covid-collateral-research).

## Declaration of interests

SVK was co-chair of the Scottish Government's Expert Reference Group on Ethnicity and COVID-19 and a member of the Scientific Advisory Group on Emergencies (SAGE) subgroup on ethnicity. RM and RME were members of the SAGE subgroup on ethnicity. REC has personal shares in AstraZeneca (AZ) unrelated to this work. BMK is also employed by NHS England (all declarations are openly available at: https://www.whopaysthisdoctor.org/doctor/491/active). JFH has grant funding from UKRI and the Wellcome Trust, has a patent with Juli Health unrelated to this work and has received consultancy fees from Juli Health and the Wellcome Trust unrelated to this work. RM is supported by Barts Charity (MGU0504), receives salary contributions from Genes & Health and has received consultancy fees from AMGEN. JKQ has grants from MRC, HDR UK, GlaxoSmithKline (GSK), BI, asthma + lung UK, and AZ and has received fees from GSK, Evidera, AZ and Insmed. SL was co-founder and co-chair of the RECORD steering committee and has a leadership role at Health Data Research UK. KM has received consultancy fees from AMGEN. LT has grant funding from MRC, the Wellcome Trust and is funded by an NIHR Research Professorship (NIHR302405), has consulted for Bayer and is on the MHRA expert advisory group (Women's health) and a member of 4 non-industry funded trial advisory committees (unpaid). All other authors declare no competing interests. AM has received consultancy fees from induction health and is a member of RCGP health informatics group and the NHS Digital GP data Professional Advisory Group.
