# Supplementary material for: Ethnic differences in the indirect effects of the COVID-19 pandemic on clinical monitoring and hospitalisations for non-COVID conditions in England: a population-based, observational cohort study using the OpenSAFELY platform
Source: eClinicalMedicine. 2023 Jun 29;61:102077. doi: 10.1016/j.eclinm.2023.102077 (PMC10331810; doi:10.1016/j.eclinm.2023.102077)
Supplement: Supplementary materials [file mmc1.docx]

**Supplementary Materials**

**Table of Contents:**

[Further information governance details 2](#_Toc136939503)

[Further details of time-series analysis 3](#_Toc136939504)

[Figure 1: Flowchart of study population 1](#_Toc136939505)

[Table 1: Baseline characteristic as of 1^st^ January 2019 1](#_Toc136939506)

[Table 2: Baseline characteristic as of 1^st^ January 2021 5](#_Toc136939507)

[Table 3: Results from Cox proportional hazards regression models 10](#_Toc136939508)

[Figure 2: Monthly rate of change in clinical monitoring by ethnic group. 0](#_Toc136939509)

[Figure 3: a) Age and sex adjusted hazard ratios pre-pandemic and each pandemic time-period for each non-White ethnic group versus White, b) Ratio of hazard ratios for each ethnic group. 1](#_Toc136939510)

[Figure 4: a) Fully adjusted hazard ratios pre-pandemic and pandemic for each non-White ethnic group versus White, b) Ratio of hazard ratios for each ethnic group. 2](#_Toc136939511)

[Figure 5: a) Fully adjusted hazard ratios pre-pandemic and each pandemic time-period for each non-White ethnic group versus White, b) Ratio of hazard ratios for each ethnic group. 3](#_Toc136939512)

[Figure 6: a) Fully adjusted hazard ratios pre-pandemic and pandemic for each ethnic group versus Asian, b) Ratio of hazard ratios for each ethnic group 4](#_Toc136939513)

[Figure 7: a) Fully adjusted hazard ratios pre-pandemic and pandemic for each ethnic group versus Black, b) Ratio of hazard ratios for each ethnic group 5](#_Toc136939514)

[Figure 8: a) Fully adjusted hazard ratios pre-pandemic and pandemic for each ethnic group versus Mixed, b) Ratio of hazard ratios for each ethnic group 6](#_Toc136939515)

[Figure 9: a) Fully adjusted hazard ratios pre-pandemic and pandemic for each ethnic group versus Other ethnic group, b) Ratio of hazard ratios for each ethnic group 7](#_Toc136939516)

[Table 4: Number of events, rate, hazard ratio and confidence intervals by ethnic group, time period and outcome with those of Asian ethnicity as the reference group. 8](#_Toc136939517)

[Table 5: : Number of events, rate, hazard ratio and confidence intervals by ethnic group, time period and outcome with those of Black ethnicity as the reference group. 31](#_Toc136939518)

[Table 6: Number of events, rate, hazard ratio and confidence intervals by ethnic group, time period and outcome with those of Mixed ethnicity as the reference group. 52](#_Toc136939519)

[Table 7: Number of events, rate, hazard ratio and confidence intervals by ethnic group, time period and outcome with those of Other ethnicity as the reference group. 74](#_Toc136939520)

[Schoenfeld residual plots for pre-pandemic and pandemic time-periods where scaled residuals for each ethnicity versus White are shown for each outcome. All plots are for age and sex adjusted models. 95](#_Toc136939521)

### Further information governance details

**Information governance and ethical approval**

NHS England is the data controller for OpenSAFELY-TPP; TPP is the data processor; all study authors using OpenSAFELY have the approval of NHS England. This implementation of OpenSAFELY is hosted within the TPP environment which is accredited to the ISO 27001 information security standard and is NHS IG Toolkit compliant.

Patient data has been pseudonymised for analysis and linkage using industry standard cryptographic hashing techniques; all pseudonymised datasets transmitted for linkage onto OpenSAFELY are encrypted; access to the platform is via a virtual private network (VPN) connection, restricted to a small group of researchers; the researchers hold contracts with NHS England and only access the platform to initiate database queries and statistical models; all database activity is logged; only aggregate statistical outputs leave the platform environment following best practice for anonymisation of results such as statistical disclosure control for low cell counts.

The OpenSAFELY research platform adheres to the obligations of the UK General Data Protection Regulation (GDPR) and the Data Protection Act 2018. In March 2020, the Secretary of State for Health and Social Care used powers under the UK Health Service (Control of Patient Information) Regulations 2002 (COPI) to require organisations to process confidential patient information for the purposes of protecting public health, providing healthcare services to the public and monitoring and managing the COVID-19 outbreak and incidents of exposure; this sets aside the requirement for patient consent. This was extended in November 2022 for the NHS England OpenSAFELY COVID-19 research platform. In some cases of data sharing, the common law duty of confidence is met using, for example, patient consent or support from the Health Research Authority Confidentiality Advisory Group.

Taken together, these provide the legal bases to link patient datasets on the OpenSAFELY platform. GP practices, from which the primary care data are obtained, are required to share relevant health information to support the public health response to the pandemic, and have been informed of the OpenSAFELY analytics platform.

Further information: <https://www.opensafely.org/policies-for-researchers/#fn:3>

### Further details of time-series analysis

Lags were determined by running a series of model checks, including autoregression and partial autoregression plots. There was no statistically significant autocorrelation beyond time-adjacent observations (i.e., beyond y_t and y_t+1). Therefore, lags of 1 were chosen in our models. Given the finite sample structure, we utilised the Barlett (i.e., triangle) kernel for our time-series analysis as per default. There is some evidence that this may introduce negative bias compared to the theoretically optimal Quadratic Spectral kernel.^1^

1. Kolokotrones, T., Stock, J. H. & Walker, C. D. Is Newey–West optimal among first-order kernels? *J. Econom.* (2023) doi:10.1016/j.jeconom.2022.12.013.

### Figure 1: Flowchart of study population


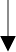


6517 died prior to 1^st^ January 2020


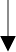


23,847,127 were registered more than 3 months

9,663,810 <3 months follow-up

33,510,937 registered with general practice using TPP software as of 1^st^ January 2020

23,840,610 were alive on 1^st^ January 2020


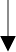


19,064,019 met age inclusion

4,776,591 Age <18 or >110

18,724,113 IMD inclusion criteria


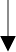


339,458 IMD unknown


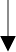


448 Sex indeterminate or Unknown


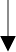


16,053,268 included in the study

2,670,845 Household size missing or >15

19,063,571 met sex inclusion criteria

### Table 1: Baseline characteristic as of 1^st^ January 2019

|  |  | **All** | **White** | **Asian** | **Black** | **Mixed** | **Other** | **Missing** |
| --- | --- | --- | --- | --- | --- | --- | --- | --- |
|  |  | N=15,240,839 | N=12,371,877 | N=1,007,021 | N=346,912 | N=181,304 | N=282,820 | N=1,050,905 |
| Age category | 18 - 40 years | 5,432,500 (35.6%) | 4,024,489 (32.5%) | 505,586 (50.2%) | 153,544 (44.3%) | 104,940 (57.9%) | 158,923 (56.2%) | 485,018 (46.2%) |
|  | 41 - 60 years | 5,119,204 (33.6%) | 4,116,363 (33.3%) | 348,220 (34.6%) | 145,973 (42.1%) | 57,928 (32.0%) | 88,967 (31.5%) | 361,753 (34.4%) |
|  | 61 - 80 years | 3,806,610 (25.0%) | 3,412,570 (27.6%) | 131,804 (13.1%) | 38,850 (11.2%) | 15,906 (8.8%) | 30,389 (10.7%) | 177,091 (16.9%) |
|  | >80 years | 882,525 (5.8%) | 818,455 (6.6%) | 21,411 (2.1%) | 8,545 (2.5%) | 2,530 (1.4%) | 4,541 (1.6%) | 27,043 (2.6%) |
| Sex | F | 7,719,630 (50.7%) | 6,432,324 (52.0%) | 491,714 (48.8%) | 174,987 (50.4%) | 94,282 (52.0%) | 138,316 (48.9%) | 388,007 (36.9%) |
|  | M | 7,521,209 (49.3%) | 5,939,553 (48.0%) | 515,307 (51.2%) | 171,925 (49.6%) | 87,022 (48.0%) | 144,504 (51.1%) | 662,898 (63.1%) |
| Index of Multiple Deprivation | 1 (most deprived) | 3,007,600 (19.7%) | 2,221,345 (18.0%) | 331,524 (32.9%) | 142,192 (41.0%) | 52,965 (29.2%) | 72,741 (25.7%) | 186,833 (17.8%) |
|  | 2 | 3,049,732 (20.0%) | 2,363,372 (19.1%) | 283,693 (28.2%) | 89,603 (25.8%) | 42,204 (23.3%) | 68,809 (24.3%) | 202,051 (19.2%) |
|  | 3 | 3,292,336 (21.6%) | 2,719,238 (22.0%) | 193,541 (19.2%) | 58,671 (16.9%) | 36,292 (20.0%) | 58,172 (20.6%) | 226,422 (21.5%) |
|  | 4 | 3,086,652 (20.3%) | 2,640,859 (21.3%) | 114,658 (11.4%) | 34,807 (10.0%) | 28,064 (15.5%) | 46,514 (16.4%) | 221,750 (21.1%) |
|  | 5 (least deprived) | 2,804,519 (18.4%) | 2,427,063 (19.6%) | 83,605 (8.3%) | 21,639 (6.2%) | 21,779 (12.0%) | 36,584 (12.9%) | 213,849 (20.3%) |
| Region of England | East | 3,532,801 (23.2%) | 2,938,198 (23.8%) | 161,695 (16.1%) | 84,468 (24.4%) | 42,113 (23.2%) | 50,739 (18.0%) | 255,588 (24.3%) |
|  | East Midlands | 2,697,760 (17.7%) | 2,190,436 (17.7%) | 196,420 (19.5%) | 57,020 (16.4%) | 31,163 (17.2%) | 40,191 (14.2%) | 182,530 (17.4%) |
|  | London | 1,063,184 ( 7.0%) | 549,965 (4.4%) | 238,368 (23.7%) | 87,064 (25.1%) | 34,363 (19.0%) | 81,035 (28.7%) | 72,389 (6.9%) |
|  | North East | 743,619 ( 4.9%) | 646,011 (5.2%) | 27,746 (2.8%) | 11,090 (3.2%) | 6,991 (3.9%) | 12,888 (4.6%) | 38,893 (3.7%) |
|  | North West | 1,339,595 ( 8.8%) | 1,195,017 (9.7%) | 37,968 (3.8%) | 8,697 (2.5%) | 6,568 (3.6%) | 14,186 (5.0%) | 77,159 (7.3%) |
|  | South East | 1,037,712 (6.8%) | 869,892 (7.0%) | 30,496 (3.0%) | 12,605 (3.6%) | 11,818 (6.5%) | 18,846 (6.7%) | 94,055 (9.0%) |
|  | South West | 2,091,775 (13.7%) | 1,848,089 (14.9%) | 35,328 (3.5%) | 13,086 (3.8%) | 15,332 (8.5%) | 20,869 (7.4%) | 159,071 (15.1%) |
|  | West Midlands | 593,795 ( 3.9%) | 394,074 (3.2%) | 95,684 (9.5%) | 38,005 (11.0%) | 12,713 (7.0%) | 14,706 (5.2%) | 38,613 (3.7%) |
|  | Yorkshire and The Humber | 2,133,793 (14.0%) | 1,734,854 (14.0%) | 182,772 (18.2%) | 34,642 (10.0%) | 20,094 (11.1%) | 29,207 (10.3%) | 132,224 (12.6%) |
| Urban rural classification | Urban major conurbation | 3,066,978 (20.1%) | 1,997,151 (16.1%) | 521,390 (51.8%) | 167,994 (48.4%) | 66,479 (36.7%) | 122,857 (43.4%) | 191,107 (18.2%) |
|  | Urban minor conurbation | 979,968 ( 6.4%) | 814,565 (6.6%) | 47,257 (4.7%) | 24,653 (7.1%) | 13,392 (7.4%) | 19,095 (6.8%) | 61,006 (5.8%) |
|  | Urban city and town | 7,908,893 (51.9%) | 6,574,073 (53.1%) | 412,136 (40.9%) | 142,326 (41.0%) | 86,661 (47.8%) | 124,389 (44.0%) | 569,308 (54.2%) |
|  | Urban city and town in sparse setting | 24,929 (0.2%) | 23,084 (0.2%) | 113 (0.0%) | 38 (0.0%) | 109 (0.1%) | 85 (0.0%) | 1,500 (0.1%) |
|  | Rural town and fringe | 1,747,230 (11.5%) | 1,584,313 (12.8%) | 15,976 (1.6%) | 7,571 (2.2%) | 8,385 (4.6%) | 9,901 (3.5%) | 121,084 (11.5%) |
|  | Rural town and fringe in sparse setting | 79,194 ( 0.5%) | 73,971 (0.6%) | 325 (0.0%) | 122 (0.0%) | 253 (0.1%) | 333 (0.1%) | 4,190 (0.4%) |
|  | Rural village and dispersed | 1,329,607 (8.7%) | 1,207,948 (9.8%) | 9,628 (1.0%) | 4,069 (1.2%) | 5,737 (3.2%) | 5,939 (2.1%) | 96,286 (9.2%) |
|  | Rural village and dispersed in a sparse setting | 104,040 (0.7%) | 96,772 (0.8%) | 196 (0.0%) | 139 (0.0%) | 288 (0.2%) | 221 (0.1%) | 6,424 (0.6%) |
| Comorbidities | | | | | | | | |
| Type 1 diabetes | Yes | 105,683 (0.7%) | 92,832 (0.8%) | 6,418 (0.6%) | 2,679 (0.8%) | 1,054 (0.6%) | 1,055 (0.4%) | 1,645 (0.2%) |
| Type 2 diabetes | Yes | 1,057,308 (6.9%) | 851,943 (6.9%) | 124,074 (12.3%) | 30,755 (8.9%) | 9,512 (5.2%) | 13,235 (4.7%) | 27,789 (2.6%) |
| Asthma | Yes | 1,313,516 (8.6%) | 1,143,753 (9.2%) | 76,631 (7.6%) | 22,959 (6.6%) | 14,989 (8.3%) | 11,987 (4.2%) | 43,197 (4.1%) |
| COPD | Yes | 467,964 (3.1%) | 444,769 (3.6%) | 9,061 (0.9%) | 2,837 (0.8%) | 1,544 (0.9%) | 2,060 (0.7%) | 7,693 (0.7%) |
| CVD | Yes | 1,674,585 (11.0%) | 1,539,691 (12.4%) | 62,594 (6.2%) | 19,032 (5.5%) | 8,112 (4.5%) | 11,044 (3.9%) | 34,112 (3.2%) |
| Serious mental illness | Yes | 179,207 (1.2%) | 148,412 (1.2%) | 12,730 (1.3%) | 7,322 (2.1%) | 3,392 (1.9%) | 2,716 (1.0%) | 4,635 (0.4%) |

###

###

### Table 2: Baseline characteristic as of 1^st^ January 2021

|  |  | **All** | **White** | **Asian** | **Black** | **Mixed** | **Other** | **Missing** |
| --- | --- | --- | --- | --- | --- | --- | --- | --- |
|  |  | N=15,992,957 | N=12,825,103 | N=1,111,024 | N=385,424 | N=204,428 | N=326,105 | N=1,140,873 |
| Age category | 18 - 40 years | 5,634,744 (35.2%) | 4,101,246 (32.0%) | 535,641 (48.2%) | 166,533 (43.2%) | 116,559 (57.0%) | 180,827 (55.5%) | 533,938 (46.8%) |
|  | 41 - 60 years | 5,283,834 (33.0%) | 4,177,166 (32.6%) | 400,813 (36.1%) | 161,473 (41.9%) | 65,860 (32.2%) | 104,278 (32.0%) | 374,244 (32.8%) |
|  | 61 - 80 years | 4,045,375 (25.3%) | 3,595,619 (28.0%) | 148,221 (13.3%) | 46,927 (12.2%) | 18,886 (9.2%) | 35,415 (10.9%) | 200,307 (17.6%) |
|  | >80 years | 1,029,004 (6.4%) | 951,072 (7.4%) | 26,349 (2.4%) | 10,491 (2.7%) | 3,123 (1.5%) | 5,585 (1.7%) | 32,384 (2.8%) |
| Sex | F | 8,085,727 (50.6%) | 6,654,191 (51.9%) | 543,796 (48.9%) | 194,200 (50.4%) | 105,959 (51.8%) | 160,233 (49.1%) | 427,348 (37.5%) |
|  | M | 7,907,230 (49.4%) | 6,170,912 (48.1%) | 567,228 (51.1%) | 191,224 (49.6%) | 98,469 (48.2%) | 165,872 (50.9%) | 713,525 (62.5%) |
| Index of Multiple Deprivation | 1 (most deprived) | 3,166,772 (19.8%) | 2,299,523 (17.9%) | 362,601 (32.6%) | 156,915 (40.7%) | 59,028 (28.9%) | 82,975 (25.4%) | 205,730 (18.0%) |
|  | 2 | 3,215,702 (20.1%) | 2,454,567 (19.1%) | 311,692 (28.1%) | 99,506 (25.8%) | 47,549 (23.3%) | 79,964 (24.5%) | 222,424 (19.5%) |
|  | 3 | 3,458,394 (21.6%) | 2,824,498 (22.0%) | 214,656 (19.3%) | 65,140 (16.9%) | 40,778 (19.9%) | 67,498 (20.7%) | 245,824 (21.5%) |
|  | 4 | 3,228,301 (20.2%) | 2,735,763 (21.3%) | 128,652 (11.6%) | 39,375 (10.2%) | 32,090 (15.7%) | 53,855 (16.5%) | 238,566 (20.9%) |
|  | 5 (least deprived) | 2,923,788 (18.3%) | 2,510,752 (19.6%) | 93,423 (8.4%) | 24,488 (6.4%) | 24,983 (12.2%) | 41,813 (12.8%) | 228,329 (20.0%) |
| Region of England | East | 3,691,064 (23.1%) | 3,032,904 (23.7%) | 182,711 (16.5%) | 94,237 (24.5%) | 47,380 (23.2%) | 58,447 (17.9%) | 275,385 (24.2%) |
|  | East Midlands | 2,812,881 (17.6%) | 2,256,061 (17.6%) | 215,304 (19.4%) | 63,814 (16.6%) | 34,971 (17.1%) | 45,331 (13.9%) | 197,400 (17.3%) |
|  | London | 1,185,496 (7.4%) | 605,860 (4.7%) | 260,939 (23.5%) | 93,735 (24.3%) | 38,732 (19.0%) | 95,549 (29.3%) | 90,681 (8.0%) |
|  | North East | 772,600 (4.8%) | 664,054 (5.2%) | 30,705 (2.8%) | 12,612 (3.3%) | 7,952 (3.9%) | 14,814 (4.5%) | 42,463 (3.7%) |
|  | North West | 1,387,136 (8.7%) | 1,228,698 (9.6%) | 42,081 (3.8%) | 10,095 (2.6%) | 7,439 (3.6%) | 16,204 (5.0%) | 82,619 (7.2%) |
|  | South East | 1,089,882 (6.8%) | 908,295 (7.1%) | 34,249 (3.1%) | 14,366 (3.7%) | 13,398 (6.6%) | 21,196 (6.5%) | 98,378 (8.6%) |
|  | South West | 2,195,020 (13.7%) | 1,928,114 (15.0%) | 40,686 (3.7%) | 15,080 (3.9%) | 17,511 (8.6%) | 23,953 (7.3%) | 169,676 (14.9%) |
|  | West Midlands | 628,325 (3.9%) | 407,817 (3.2%) | 105,971 (9.5%) | 41,891 (10.9%) | 14,246 (7.0%) | 16,842 (5.2%) | 41,558 (3.6%) |
|  | Yorkshire and The Humber | 2,222,859 (13.9%) | 1,787,635 (13.9%) | 197,747 (17.8%) | 39,245 (10.2%) | 22,602 (11.1%) | 33,572 (10.3%) | 142,058 (12.5%) |
| Urban rural classification | Urban major conurbation | 3,283,118 (20.5%) | 2,092,377 (16.3%) | 569,677 (51.3%) | 184,038 (47.7%) | 74,485 (36.4%) | 143,092 (43.9%) | 219,449 (19.2%) |
|  | Urban minor conurbation | 1,022,460 (6.4%) | 838,184 (6.5%) | 52,641 (4.7%) | 27,951 (7.3%) | 15,194 (7.4%) | 21,988 (6.7%) | 66,502 (5.8%) |
|  | Urban city and town | 8,272,377 (51.7%) | 6,801,598 (53.0%) | 459,001 (41.3%) | 159,705 (41.4%) | 97,924 (47.9%) | 142,299 (43.6%) | 611,850 (53.6%) |
|  | Urban city and town in sparse setting | 25,786 (0.2%) | 23,895 (0.2%) | 114 (0.0%) | 36 (0.0%) | 111 (0.1%) | 88 (0.0%) | 1,542 (0.1%) |
|  | Rural town and fringe | 1,815,101 (11.3%) | 1,638,882 (12.8%) | 18,123 (1.6%) | 8,677 (2.3%) | 9,702 (4.7%) | 11,267 (3.5%) | 128,450 (11.3%) |
|  | Rural town and fringe in sparse setting | 81,602 (0.5%) | 76,103 (0.6%) | 340 (0.0%) | 139 (0.0%) | 253 (0.1%) | 370 (0.1%) | 4,397 (0.4%) |
|  | Rural village and dispersed | 1,384,339 (8.7%) | 1,253,632 (9.8%) | 10,902 (1.0%) | 4,733 (1.2%) | 6,446 (3.2%) | 6,749 (2.1%) | 101,877 (8.9%) |
|  | Rural village and dispersed in a sparse setting | 108,174 (0.7%) | 100,432 (0.8%) | 226 (0.0%) | 145 (0.0%) | 313 (0.2%) | 252 (0.1%) | 6,806 (0.6%) |
| Comorbidities | | | | | | | | |
| Type 1 diabetes | Yes | 115,310 (0.7%) | 100,585 (0.8%) | 7,374 (0.7%) | 3,062 (0.8%) | 1,183 (0.6%) | 1,258 (0.4%) | 1,848 (0.2%) |
| Type 2 diabetes | Yes | 1,179,533 (7.4%) | 941,921 (7.3%) | 142,426 (12.8%) | 36,051 (9.4%) | 11,322 (5.5%) | 16,075 (4.9%) | 31,738 (2.8%) |
| Asthma | Yes | 1,412,135 (8.8%) | 1,223,503 (9.5%) | 85,138 (7.7%) | 25,797 (6.7%) | 17,259 (8.4%) | 13,810 (4.2%) | 46,628 (4.1%) |
| COPD | Yes | 510,957 (3.2%) | 484,478 (3.8%) | 10,210 (0.9%) | 3,264 (0.8%) | 1,812 (0.9%) | 2,459 (0.8%) | 8,734 (0.8%) |
| CVD | Yes | 1,852,628 (11.6%) | 1,699,419 (13.3%) | 71,573 (6.4%) | 22,479 (5.8%) | 9,543 (4.7%) | 13,137 (4.0%) | 36,477 (3.2%) |
| Serious mental illness | Yes | 196,196 (1.2%) | 161,622 (1.3%) | 14,237 (1.3%) | 8,327 (2.2%) | 3,915 (1.9%) | 3,219 (1.0%) | 4,876 (0.4%) |

### Table 3: Results from Cox proportional hazards regression models

| **Outcome** | **Period** | **Ethnicity** | **Denominator** | **Events** | **Total person months** | **Rate per month** | **Unadjusted** | | **Age and sex adjusted** | | **Fully adjusted*** | | **Rate difference versus Pre period** |
| --- | --- | --- | --- | --- | --- | --- | --- | --- | --- | --- | --- | --- | --- |
|  |  |  |  |  |  |  | **Hazard ratio** | **95% confidence interval** | **Hazard ratio** | **95% confidence interval** | **Hazard ratio** | **95% confidence interval** |  |
| Stroke | Pre | White | 11951861 | 42033 | 299064047 | 14.05 | Reference |  |  |  |  |  |  |
| Stroke | Pre | Asian | 947198 | 1835 | 23717609 | 7.74 | 0.62 | (0.59, 0.65) | 1.08 | (1.03, 1.14) | 0.96 | (0.92, 1.01) |  |
| Stroke | Pre | Black | 323687 | 683 | 8104085 | 8.43 | 0.68 | (0.63, 0.74) | 1.16 | (1.07, 1.25) | 0.98 | (0.91, 1.06) |  |
| Stroke | Pre | Mixed | 167862 | 249 | 4204083 | 5.92 | 0.46 | (0.41, 0.52) | 1.05 | (0.93, 1.19) | 0.97 | (0.85, 1.1) |  |
| Stroke | Pre | Other | 255875 | 311 | 6408427 | 4.85 | 0.39 | (0.35, 0.43) | 0.78 | (0.69, 0.87) | 0.75 | (0.67, 0.84) |  |
| Stroke | Pandemic | White | 13007778 | 62025 | 321371341 | 19.3 | Reference |  |  |  |  |  | 5.25 |
| Stroke | Pandemic | Asian | 1116583 | 2669 | 27539949 | 9.69 | 0.56 | (0.54, 0.59) | 1.02 | (0.98, 1.07) | 0.92 | (0.88, 0.96) | 1.95 |
| Stroke | Pandemic | Black | 389039 | 1107 | 9530751 | 11.62 | 0.68 | (0.64, 0.72) | 1.19 | (1.12, 1.26) | 1.02 | (0.96, 1.09) | 3.19 |
| Stroke | Pandemic | Mixed | 206060 | 357 | 5033434 | 7.09 | 0.4 | (0.36, 0.44) | 0.97 | (0.88, 1.08) | 0.9 | (0.81, 1) | 1.17 |
| Stroke | Pandemic | Other | 334274 | 485 | 8011353 | 6.05 | 0.35 | (0.32, 0.39) | 0.77 | (0.7, 0.84) | 0.74 | (0.68, 0.81) | 1.20 |
| Stroke | Wave 1 | White | 13008002 | 4886 | 29408734 | 16.61 | Reference |  |  |  |  |  | 2.56 |
| Stroke | Wave 1 | Asian | 1116586 | 210 | 2525075 | 8.32 | 0.56 | (0.48, 0.64) | 1.01 | (0.88, 1.17) | 0.9 | (0.78, 1.04) | 0.58 |
| Stroke | Wave 1 | Black | 389021 | 79 | 879114 | 8.99 | 0.62 | (0.5, 0.78) | 1.08 | (0.86, 1.36) | 0.92 | (0.73, 1.15) | 0.56 |
| Stroke | Wave 1 | Mixed | 206091 | 22 | 465920 | 4.72 | 0.31 | (0.2, 0.47) | 0.77 | (0.5, 1.17) | 0.7 | (0.46, 1.07) | -1.20 |
| Stroke | Wave 1 | Other | 334305 | 35 | 754927 | 4.64 | 0.31 | (0.22, 0.43) | 0.7 | (0.5, 0.97) | 0.66 | (0.47, 0.93) | -0.21 |
| Stroke | Easing 1 | White | 13059172 | 8104 | 42509205 | 19.06 | Reference |  |  |  |  |  | 5.01 |
| Stroke | Easing 1 | Asian | 1128784 | 343 | 3674505 | 9.33 | 0.55 | (0.5, 0.62) | 1.02 | (0.91, 1.14) | 0.91 | (0.81, 1.02) | 1.59 |
| Stroke | Easing 1 | Black | 393360 | 143 | 1279287 | 11.18 | 0.67 | (0.57, 0.79) | 1.19 | (1, 1.41) | 1.02 | (0.86, 1.21) | 2.75 |
| Stroke | Easing 1 | Mixed | 208793 | 49 | 678885 | 7.22 | 0.41 | (0.31, 0.55) | 1.03 | (0.78, 1.37) | 0.95 | (0.72, 1.26) | 1.30 |
| Stroke | Easing 1 | Other | 338288 | 66 | 1098220 | 6.01 | 0.36 | (0.28, 0.46) | 0.81 | (0.63, 1.03) | 0.78 | (0.61, 0.99) | 1.16 |
| Stroke | Wave 2 | White | 12967133 | 19186 | 97788237 | 19.62 | Reference |  |  |  |  |  | 5.57 |
| Stroke | Wave 2 | Asian | 1122606 | 839 | 8458675 | 9.92 | 0.57 | (0.53, 0.61) | 1.04 | (0.96, 1.11) | 0.93 | (0.86, 1) | 2.18 |
| Stroke | Wave 2 | Black | 390035 | 338 | 2934168 | 11.52 | 0.66 | (0.59, 0.74) | 1.16 | (1.04, 1.3) | 1 | (0.89, 1.11) | 3.09 |
| Stroke | Wave 2 | Mixed | 207204 | 115 | 1555135 | 7.39 | 0.41 | (0.34, 0.49) | 1.01 | (0.84, 1.22) | 0.94 | (0.78, 1.13) | 1.47 |
| Stroke | Wave 2 | Other | 332901 | 156 | 2485211 | 6.28 | 0.36 | (0.31, 0.42) | 0.8 | (0.68, 0.94) | 0.76 | (0.65, 0.9) | 1.43 |
| Stroke | Easing 2 | White | 12640411 | 2984 | 13461555 | 22.17 | Reference |  |  |  |  |  | 8.12 |
| Stroke | Easing 2 | Asian | 1095134 | 114 | 1166215 | 9.78 | 0.5 | (0.41, 0.61) | 0.9 | (0.74, 1.09) | 0.8 | (0.66, 0.97) | 2.04 |
| Stroke | Easing 2 | Black | 379052 | 59 | 403523 | 14.62 | 0.76 | (0.59, 0.99) | 1.33 | (1.02, 1.73) | 1.11 | (0.85, 1.45) | 6.19 |
| Stroke | Easing 2 | Mixed | 201010 | 12 | 213928 | 5.61 | 0.28 | (0.16, 0.49) | 0.68 | (0.38, 1.19) | 0.62 | (0.35, 1.09) | -0.31 |
| Stroke | Easing 2 | Other | 317013 | 23 | 337061 | 6.82 | 0.35 | (0.23, 0.52) | 0.75 | (0.49, 1.13) | 0.71 | (0.47, 1.08) | 1.97 |
| Stroke | Wave 3 | White | 12602238 | 16633 | 83144516 | 20 | Reference |  |  |  |  |  | 5.95 |
| Stroke | Wave 3 | Asian | 1091687 | 756 | 7193014 | 10.51 | 0.59 | (0.55, 0.64) | 1.07 | (0.99, 1.16) | 0.96 | (0.89, 1.03) | 2.77 |
| Stroke | Wave 3 | Black | 377684 | 312 | 2482157 | 12.57 | 0.71 | (0.63, 0.79) | 1.22 | (1.09, 1.37) | 1.04 | (0.93, 1.17) | 4.14 |
| Stroke | Wave 3 | Mixed | 200227 | 98 | 1314892 | 7.45 | 0.41 | (0.33, 0.5) | 0.97 | (0.8, 1.19) | 0.9 | (0.73, 1.1) | 1.53 |
| Stroke | Wave 3 | Other | 314766 | 148 | 2054547 | 7.2 | 0.41 | (0.34, 0.48) | 0.87 | (0.74, 1.02) | 0.83 | (0.7, 0.98) | 2.35 |
| Stroke | Easing 3 | White | 12411670 | 11376 | 55895206 | 20.35 | Reference |  |  |  |  |  | 6.30 |
| Stroke | Easing 3 | Asian | 1074695 | 487 | 4837290 | 10.07 | 0.56 | (0.51, 0.61) | 1.02 | (0.93, 1.12) | 0.92 | (0.84, 1.02) | 2.33 |
| Stroke | Easing 3 | Black | 369667 | 210 | 1661122 | 12.64 | 0.7 | (0.61, 0.81) | 1.23 | (1.07, 1.41) | 1.06 | (0.92, 1.22) | 4.21 |
| Stroke | Easing 3 | Mixed | 197112 | 71 | 885370 | 8.020001 | 0.43 | (0.34, 0.54) | 1.05 | (0.83, 1.33) | 0.98 | (0.77, 1.24) | 2.10 |
| Stroke | Easing 3 | Other | 304150 | 76 | 1360142 | 5.59 | 0.31 | (0.25, 0.39) | 0.67 | (0.53, 0.84) | 0.64 | (0.51, 0.8) | 0.74 |
| VTE | Pre | White | 11951881 | 28859 | 299215565 | 9.64 | Reference |  |  |  |  |  |  |
| VTE | Pre | Asian | 947196 | 752 | 23730820 | 3.17 | 0.36 | (0.34, 0.39) | 0.48 | (0.45, 0.52) | 0.42 | (0.39, 0.45) |  |
| VTE | Pre | Black | 323685 | 501 | 8106277 | 6.18 | 0.7 | (0.64, 0.77) | 0.91 | (0.83, 1) | 0.74 | (0.68, 0.81) |  |
| VTE | Pre | Mixed | 167862 | 195 | 4204866 | 4.64 | 0.51 | (0.44, 0.59) | 0.78 | (0.67, 0.89) | 0.71 | (0.61, 0.81) |  |
| VTE | Pre | Other | 255875 | 181 | 6409861 | 2.82 | 0.32 | (0.28, 0.37) | 0.45 | (0.39, 0.53) | 0.44 | (0.38, 0.51) |  |
| VTE | Pandemic | White | 13007823 | 38710 | 321532860 | 12.04 | Reference |  |  |  |  |  | 2.40 |
| VTE | Pandemic | Asian | 1116586 | 939 | 27555834 | 3.41 | 0.32 | (0.3, 0.34) | 0.43 | (0.41, 0.46) | 0.38 | (0.35, 0.4) | 0.24 |
| VTE | Pandemic | Black | 389040 | 785 | 9532588 | 8.23 | 0.75 | (0.7, 0.8) | 0.99 | (0.92, 1.07) | 0.81 | (0.76, 0.87) | 2.05 |
| VTE | Pandemic | Mixed | 206060 | 290 | 5033737 | 5.76 | 0.51 | (0.45, 0.57) | 0.81 | (0.72, 0.91) | 0.74 | (0.66, 0.83) | 1.12 |
| VTE | Pandemic | Other | 334273 | 275 | 8013201 | 3.43 | 0.31 | (0.28, 0.35) | 0.46 | (0.41, 0.52) | 0.45 | (0.4, 0.51) | 0.61 |
| VTE | Wave 1 | White | 13008047 | 2634 | 29410861 | 8.96 | Reference |  |  |  |  |  | -0.68 |
| VTE | Wave 1 | Asian | 1116589 | 59 | 2525212 | 2.34 | 0.28 | (0.22, 0.37) | 0.38 | (0.29, 0.49) | 0.32 | (0.25, 0.42) | -0.83 |
| VTE | Wave 1 | Black | 389022 | 65 | 879116 | 7.39 | 0.89 | (0.69, 1.14) | 1.17 | (0.91, 1.51) | 0.93 | (0.72, 1.19) | 1.21 |
| VTE | Wave 1 | Mixed | 206091 | 17 | 465927 | 3.65 | 0.43 | (0.27, 0.69) | 0.69 | (0.43, 1.11) | 0.62 | (0.38, 1) | -0.99 |
| VTE | Wave 1 | Other | 334304 | 17 | 754936 | 2.25 | 0.27 | (0.17, 0.44) | 0.41 | (0.25, 0.66) | 0.4 | (0.25, 0.64) | -0.57 |
| VTE | Easing 1 | White | 13059222 | 5157 | 42512892 | 12.13 | Reference |  |  |  |  |  | 2.49 |
| VTE | Easing 1 | Asian | 1128788 | 116 | 3674863 | 3.16 | 0.29 | (0.24, 0.35) | 0.39 | (0.33, 0.48) | 0.34 | (0.28, 0.41) | -0.01 |
| VTE | Easing 1 | Black | 393361 | 93 | 1279337 | 7.27 | 0.66 | (0.54, 0.81) | 0.86 | (0.7, 1.06) | 0.69 | (0.56, 0.85) | 1.09 |
| VTE | Easing 1 | Mixed | 208793 | 36 | 678890 | 5.3 | 0.46 | (0.33, 0.65) | 0.73 | (0.53, 1.02) | 0.66 | (0.48, 0.92) | 0.66 |
| VTE | Easing 1 | Other | 338288 | 39 | 1098249 | 3.55 | 0.32 | (0.23, 0.44) | 0.47 | (0.34, 0.65) | 0.46 | (0.33, 0.63) | 0.73 |
| VTE | Wave 2 | White | 12967150 | 12627 | 97803435 | 12.91 | Reference |  |  |  |  |  | 3.27 |
| VTE | Wave 2 | Asian | 1122608 | 324 | 8460268 | 3.83 | 0.34 | (0.3, 0.38) | 0.47 | (0.42, 0.52) | 0.41 | (0.36, 0.45) | 0.66 |
| VTE | Wave 2 | Black | 390036 | 253 | 2934299 | 8.62 | 0.74 | (0.65, 0.84) | 0.98 | (0.87, 1.12) | 0.8 | (0.71, 0.91) | 2.44 |
| VTE | Wave 2 | Mixed | 207205 | 101 | 1555115 | 6.49 | 0.54 | (0.44, 0.66) | 0.87 | (0.72, 1.06) | 0.79 | (0.65, 0.97) | 1.85 |
| VTE | Wave 2 | Other | 332902 | 80 | 2485458 | 3.22 | 0.28 | (0.22, 0.34) | 0.42 | (0.34, 0.52) | 0.41 | (0.32, 0.51) | 0.40 |
| VTE | Easing 2 | White | 12640457 | 1801 | 13462095 | 13.38 | Reference |  |  |  |  |  | 3.74 |
| VTE | Easing 2 | Asian | 1095139 | 41 | 1166258 | 3.52 | 0.29 | (0.21, 0.4) | 0.4 | (0.29, 0.55) | 0.34 | (0.25, 0.47) | 0.35 |
| VTE | Easing 2 | Black | 379052 | 38 | 403537 | 9.42 | 0.76 | (0.55, 1.05) | 1.02 | (0.74, 1.42) | 0.82 | (0.59, 1.14) | 3.24 |
| VTE | Easing 2 | Mixed | 201011 | 14 | 213929 | 6.54 | 0.52 | (0.31, 0.88) | 0.84 | (0.5, 1.43) | 0.76 | (0.45, 1.3) | 1.90 |
| VTE | Easing 2 | Other | 317012 | 12 | 337066 | 3.56 | 0.29 | (0.16, 0.51) | 0.44 | (0.25, 0.77) | 0.42 | (0.24, 0.75) | 0.74 |
| VTE | Wave 3 | White | 12602274 | 10091 | 83160465 | 12.13 | Reference |  |  |  |  |  | 2.49 |
| VTE | Wave 3 | Asian | 1091690 | 249 | 7194558 | 3.46 | 0.33 | (0.29, 0.37) | 0.44 | (0.38, 0.5) | 0.38 | (0.33, 0.43) | 0.29 |
| VTE | Wave 3 | Black | 377684 | 202 | 2482470 | 8.14 | 0.74 | (0.64, 0.85) | 0.97 | (0.84, 1.11) | 0.79 | (0.68, 0.91) | 1.96 |
| VTE | Wave 3 | Mixed | 200227 | 80 | 1314939 | 6.08 | 0.54 | (0.43, 0.67) | 0.83 | (0.67, 1.04) | 0.76 | (0.61, 0.95) | 1.44 |
| VTE | Wave 3 | Other | 314767 | 80 | 2054751 | 3.89 | 0.35 | (0.28, 0.43) | 0.51 | (0.41, 0.64) | 0.49 | (0.39, 0.61) | 1.07 |
| VTE | Easing 3 | White | 12411687 | 6834 | 55902234 | 12.22 | Reference |  |  |  |  |  | 2.58 |
| VTE | Easing 3 | Asian | 1074698 | 184 | 4837902 | 3.8 | 0.36 | (0.31, 0.41) | 0.49 | (0.42, 0.56) | 0.43 | (0.37, 0.5) | 0.63 |
| VTE | Easing 3 | Black | 369670 | 136 | 1661279 | 8.19 | 0.73 | (0.62, 0.87) | 0.98 | (0.82, 1.16) | 0.83 | (0.7, 0.99) | 2.01 |
| VTE | Easing 3 | Mixed | 197112 | 46 | 885409 | 5.2 | 0.46 | (0.34, 0.61) | 0.72 | (0.54, 0.97) | 0.67 | (0.5, 0.9) | 0.56 |
| VTE | Easing 3 | Other | 304148 | 41 | 1360203 | 3.01 | 0.27 | (0.2, 0.37) | 0.4 | (0.29, 0.55) | 0.39 | (0.28, 0.53) | 0.19 |
| Heart failure | Pre | White | 11951886 | 31037 | 299209045 | 10.37 | Reference |  |  |  |  |  |  |
| Heart failure | Pre | Asian | 947193 | 1925 | 23716286 | 8.12 | 0.78 | (0.74, 0.82) | 1.56 | (1.49, 1.64) | 1.22 | (1.16, 1.28) |  |
| Heart failure | Pre | Black | 323687 | 597 | 8104779 | 7.37 | 0.71 | (0.66, 0.77) | 1.41 | (1.3, 1.53) | 0.99 | (0.91, 1.08) |  |
| Heart failure | Pre | Mixed | 167861 | 166 | 4204968 | 3.95 | 0.39 | (0.33, 0.45) | 1.1 | (0.95, 1.29) | 0.9 | (0.77, 1.05) |  |
| Heart failure | Pre | Other | 255875 | 222 | 6409411 | 3.46 | 0.35 | (0.3, 0.39) | 0.83 | (0.73, 0.95) | 0.77 | (0.67, 0.88) |  |
| Heart failure | Pandemic | White | 13007814 | 50729 | 321522961 | 15.78 | Reference |  |  |  |  |  | 5.41 |
| Heart failure | Pandemic | Asian | 1116584 | 2508 | 27542692 | 9.11 | 0.59 | (0.57, 0.62) | 1.24 | (1.19, 1.29) | 0.99 | (0.94, 1.03) | 0.99 |
| Heart failure | Pandemic | Black | 389038 | 842 | 9532933 | 8.83 | 0.58 | (0.54, 0.62) | 1.16 | (1.09, 1.25) | 0.84 | (0.79, 0.9) | 1.46 |
| Heart failure | Pandemic | Mixed | 206060 | 284 | 5034330 | 5.64 | 0.37 | (0.33, 0.42) | 1.13 | (1, 1.27) | 0.94 | (0.83, 1.05) | 1.69 |
| Heart failure | Pandemic | Other | 334275 | 362 | 8013004 | 4.52 | 0.3 | (0.27, 0.34) | 0.81 | (0.73, 0.9) | 0.74 | (0.67, 0.82) | 1.06 |
| Heart failure | Wave 1 | White | 13008038 | 3540 | 29410481 | 12.04 | Reference |  |  |  |  |  | 1.67 |
| Heart failure | Wave 1 | Asian | 1116587 | 185 | 2525095 | 7.33 | 0.63 | (0.54, 0.74) | 1.32 | (1.13, 1.54) | 1.04 | (0.89, 1.22) | -0.79 |
| Heart failure | Wave 1 | Black | 389020 | 65 | 879134 | 7.39 | 0.65 | (0.51, 0.84) | 1.31 | (1.02, 1.68) | 0.94 | (0.73, 1.21) | 0.02 |
| Heart failure | Wave 1 | Mixed | 206091 | 17 | 465929 | 3.65 | 0.32 | (0.2, 0.51) | 0.98 | (0.61, 1.58) | 0.81 | (0.5, 1.31) | -0.30 |
| Heart failure | Wave 1 | Other | 334306 | 27 | 754939 | 3.58 | 0.31 | (0.21, 0.45) | 0.83 | (0.56, 1.23) | 0.76 | (0.51, 1.12) | 0.12 |
| Heart failure | Easing 1 | White | 13059194 | 6970 | 42510238 | 16.4 | Reference |  |  |  |  |  | 6.03 |
| Heart failure | Easing 1 | Asian | 1128784 | 376 | 3674476 | 10.23 | 0.66 | (0.59, 0.74) | 1.39 | (1.25, 1.56) | 1.09 | (0.97, 1.21) | 2.11 |
| Heart failure | Easing 1 | Black | 393360 | 121 | 1279279 | 9.46 | 0.61 | (0.51, 0.73) | 1.23 | (1.02, 1.48) | 0.87 | (0.72, 1.04) | 2.09 |
| Heart failure | Easing 1 | Mixed | 208793 | 38 | 678881 | 5.6 | 0.36 | (0.26, 0.49) | 1.11 | (0.81, 1.53) | 0.91 | (0.66, 1.25) | 1.65 |
| Heart failure | Easing 1 | Other | 338286 | 37 | 1098249 | 3.37 | 0.22 | (0.16, 0.3) | 0.6 | (0.43, 0.83) | 0.54 | (0.39, 0.75) | -0.09 |
| Heart failure | Wave 2 | White | 12967138 | 16378 | 97799415 | 16.75 | Reference |  |  |  |  |  | 6.38 |
| Heart failure | Wave 2 | Asian | 1122605 | 844 | 8458766 | 9.98 | 0.61 | (0.57, 0.66) | 1.28 | (1.19, 1.37) | 1 | (0.93, 1.08) | 1.86 |
| Heart failure | Wave 2 | Black | 390036 | 311 | 2934208 | 10.6 | 0.66 | (0.59, 0.74) | 1.32 | (1.18, 1.48) | 0.94 | (0.84, 1.06) | 3.23 |
| Heart failure | Wave 2 | Mixed | 207204 | 89 | 1555247 | 5.72 | 0.35 | (0.29, 0.43) | 1.08 | (0.88, 1.33) | 0.89 | (0.72, 1.1) | 1.77 |
| Heart failure | Wave 2 | Other | 332902 | 131 | 2485311 | 5.27 | 0.33 | (0.28, 0.4) | 0.9 | (0.76, 1.07) | 0.82 | (0.69, 0.97) | 1.81 |
| Heart failure | Easing 2 | White | 12640422 | 3464 | 13461250 | 25.73 | Reference |  |  |  |  |  | 15.36 |
| Heart failure | Easing 2 | Asian | 1095135 | 199 | 1166170 | 17.06 | 0.69 | (0.59, 0.8) | 1.49 | (1.28, 1.73) | 1.16 | (0.99, 1.35) | 8.94 |
| Heart failure | Easing 2 | Black | 379052 | 52 | 403527 | 12.89 | 0.52 | (0.4, 0.69) | 1.07 | (0.81, 1.42) | 0.75 | (0.57, 0.99) | 5.52 |
| Heart failure | Easing 2 | Mixed | 201011 | 19 | 213926 | 8.88 | 0.36 | (0.23, 0.56) | 1.14 | (0.72, 1.79) | 0.93 | (0.59, 1.46) | 4.93 |
| Heart failure | Easing 2 | Other | 317012 | 30 | 337056 | 8.9 | 0.37 | (0.26, 0.53) | 1.02 | (0.71, 1.47) | 0.92 | (0.64, 1.32) | 5.44 |
| Heart failure | Wave 3 | White | 12602204 | 14580 | 83149534 | 17.53 | Reference |  |  |  |  |  | 7.16 |
| Heart failure | Wave 3 | Asian | 1091682 | 767 | 7192954 | 10.66 | 0.6 | (0.56, 0.65) | 1.25 | (1.16, 1.35) | 0.98 | (0.91, 1.06) | 2.54 |
| Heart failure | Wave 3 | Black | 377683 | 239 | 2482401 | 9.63 | 0.55 | (0.48, 0.62) | 1.08 | (0.95, 1.23) | 0.77 | (0.67, 0.87) | 2.26 |
| Heart failure | Wave 3 | Mixed | 200224 | 87 | 1314956 | 6.62 | 0.38 | (0.31, 0.47) | 1.14 | (0.92, 1.41) | 0.94 | (0.76, 1.16) | 2.67 |
| Heart failure | Wave 3 | Other | 314766 | 119 | 2054623 | 5.79 | 0.34 | (0.28, 0.41) | 0.89 | (0.74, 1.06) | 0.8 | (0.67, 0.96) | 2.33 |
| Heart failure | Easing 3 | White | 12411683 | 11000 | 55896009 | 19.68 | Reference |  |  |  |  |  | 9.31 |
| Heart failure | Easing 3 | Asian | 1074692 | 571 | 4837135 | 11.8 | 0.61 | (0.56, 0.66) | 1.28 | (1.17, 1.4) | 1.02 | (0.93, 1.12) | 3.68 |
| Heart failure | Easing 3 | Black | 369669 | 187 | 1661225 | 11.26 | 0.58 | (0.5, 0.67) | 1.16 | (1, 1.34) | 0.84 | (0.73, 0.98) | 3.89 |
| Heart failure | Easing 3 | Mixed | 197111 | 65 | 885385 | 7.34 | 0.38 | (0.3, 0.49) | 1.16 | (0.91, 1.48) | 0.97 | (0.76, 1.24) | 3.39 |
| Heart failure | Easing 3 | Other | 304148 | 69 | 1360166 | 5.07 | 0.27 | (0.21, 0.34) | 0.7 | (0.55, 0.89) | 0.64 | (0.5, 0.81) | 1.61 |
| MI | Pre | White | 11951868 | 41681 | 299044045 | 13.94 | Reference |  |  |  |  |  |  |
| MI | Pre | Asian | 947193 | 3206 | 23700749 | 13.53 | 1.06 | (1.02, 1.1) | 1.68 | (1.62, 1.75) | 1.46 | (1.41, 1.52) |  |
| MI | Pre | Black | 323687 | 425 | 8107170 | 5.24 | 0.42 | (0.38, 0.46) | 0.63 | (0.57, 0.69) | 0.52 | (0.47, 0.57) |  |
| MI | Pre | Mixed | 167862 | 212 | 4204384 | 5.04 | 0.39 | (0.34, 0.45) | 0.8 | (0.7, 0.92) | 0.72 | (0.63, 0.83) |  |
| MI | Pre | Other | 255872 | 329 | 6407889 | 5.13 | 0.41 | (0.37, 0.46) | 0.75 | (0.67, 0.84) | 0.71 | (0.64, 0.79) |  |
| MI | Pandemic | White | 13007792 | 49713 | 321413154 | 15.47 | Reference |  |  |  |  |  | 1.53 |
| MI | Pandemic | Asian | 1116586 | 3864 | 27525109 | 14.04 | 0.99 | (0.96, 1.03) | 1.58 | (1.53, 1.64) | 1.4 | (1.35, 1.45) | 0.51 |
| MI | Pandemic | Black | 389038 | 603 | 9534880 | 6.32 | 0.46 | (0.42, 0.5) | 0.7 | (0.64, 0.76) | 0.59 | (0.54, 0.64) | 1.08 |
| MI | Pandemic | Mixed | 206059 | 252 | 5033918 | 5.01 | 0.35 | (0.31, 0.4) | 0.73 | (0.65, 0.83) | 0.67 | (0.59, 0.76) | -0.03 |
| MI | Pandemic | Other | 334275 | 480 | 8011100 | 5.99 | 0.44 | (0.4, 0.48) | 0.83 | (0.76, 0.91) | 0.79 | (0.72, 0.86) | 0.86 |
| MI | Wave 1 | White | 13008016 | 3585 | 29409854 | 12.19 | Reference |  |  |  |  |  | -1.75 |
| MI | Wave 1 | Asian | 1116589 | 271 | 2525011 | 10.73 | 0.96 | (0.84, 1.09) | 1.51 | (1.32, 1.72) | 1.31 | (1.15, 1.5) | -2.80 |
| MI | Wave 1 | Black | 389020 | 33 | 879155 | 3.75 | 0.35 | (0.25, 0.49) | 0.52 | (0.37, 0.74) | 0.43 | (0.3, 0.61) | -1.49 |
| MI | Wave 1 | Mixed | 206090 | 17 | 465920 | 3.65 | 0.33 | (0.2, 0.53) | 0.68 | (0.42, 1.09) | 0.61 | (0.38, 0.99) | -1.39 |
| MI | Wave 1 | Other | 334306 | 21 | 754936 | 2.78 | 0.25 | (0.16, 0.38) | 0.47 | (0.3, 0.73) | 0.44 | (0.28, 0.69) | -2.35 |
| MI | Easing 1 | White | 13059185 | 6342 | 42511189 | 14.92 | Reference |  |  |  |  |  | 0.98 |
| MI | Easing 1 | Asian | 1128784 | 459 | 3674351 | 12.49 | 0.95 | (0.86, 1.05) | 1.55 | (1.4, 1.72) | 1.35 | (1.22, 1.49) | -1.04 |
| MI | Easing 1 | Black | 393362 | 71 | 1279392 | 5.55 | 0.43 | (0.34, 0.55) | 0.67 | (0.53, 0.85) | 0.56 | (0.44, 0.71) | 0.31 |
| MI | Easing 1 | Mixed | 208793 | 31 | 678906 | 4.57 | 0.34 | (0.24, 0.48) | 0.74 | (0.52, 1.05) | 0.67 | (0.47, 0.95) | -0.47 |
| MI | Easing 1 | Other | 338288 | 66 | 1098217 | 6.01 | 0.47 | (0.37, 0.59) | 0.92 | (0.72, 1.18) | 0.87 | (0.68, 1.11) | 0.88 |
| MI | Wave 2 | White | 12967152 | 15520 | 97794466 | 15.87 | Reference |  |  |  |  |  | 1.93 |
| MI | Wave 2 | Asian | 1122602 | 1172 | 8457313 | 13.86 | 0.97 | (0.92, 1.04) | 1.58 | (1.48, 1.68) | 1.39 | (1.31, 1.48) | 0.33 |
| MI | Wave 2 | Black | 390035 | 182 | 2934586 | 6.2 | 0.45 | (0.39, 0.52) | 0.7 | (0.6, 0.81) | 0.59 | (0.51, 0.68) | 0.96 |
| MI | Wave 2 | Mixed | 207205 | 81 | 1555226 | 5.21 | 0.36 | (0.29, 0.45) | 0.78 | (0.62, 0.96) | 0.71 | (0.57, 0.88) | 0.17 |
| MI | Wave 2 | Other | 332901 | 152 | 2485197 | 6.12 | 0.44 | (0.38, 0.52) | 0.86 | (0.73, 1.01) | 0.82 | (0.7, 0.97) | 0.99 |
| MI | Easing 2 | White | 12640414 | 2489 | 13461750 | 18.49 | Reference |  |  |  |  |  | 4.55 |
| MI | Easing 2 | Asian | 1095133 | 172 | 1166183 | 14.75 | 0.88 | (0.75, 1.03) | 1.44 | (1.22, 1.7) | 1.29 | (1.09, 1.52) | 1.22 |
| MI | Easing 2 | Black | 379051 | 39 | 403537 | 9.66 | 0.6 | (0.43, 0.82) | 0.94 | (0.68, 1.3) | 0.81 | (0.59, 1.12) | 4.42 |
| MI | Easing 2 | Mixed | redacted |  |  |  |  |  |  |  |  |  |  |
| MI | Easing 2 | Other | 317013 | 19 | 337062 | 5.64 | 0.34 | (0.22, 0.54) | 0.67 | (0.43, 1.06) | 0.64 | (0.41, 1.01) | 0.51 |
| MI | Wave 3 | White | 12602261 | 13201 | 83150575 | 15.88 | Reference |  |  |  |  |  | 1.94 |
| MI | Wave 3 | Asian | 1091685 | 1085 | 7191830 | 15.09 | 1.02 | (0.95, 1.09) | 1.64 | (1.53, 1.75) | 1.44 | (1.34, 1.54) | 1.56 |
| MI | Wave 3 | Black | 377686 | 169 | 2482621 | 6.81 | 0.47 | (0.4, 0.55) | 0.72 | (0.62, 0.84) | 0.6 | (0.52, 0.7) | 1.57 |
| MI | Wave 3 | Mixed | 200225 | 60 | 1314987 | 4.56 | 0.31 | (0.24, 0.4) | 0.65 | (0.5, 0.84) | 0.59 | (0.46, 0.76) | -0.48 |
| MI | Wave 3 | Other | 314768 | 133 | 2054569 | 6.47 | 0.46 | (0.39, 0.54) | 0.86 | (0.73, 1.02) | 0.82 | (0.69, 0.97) | 1.34 |
| MI | Easing 3 | White | 12411685 | 9252 | 55898155 | 16.55 | Reference |  |  |  |  |  | 2.61 |
| MI | Easing 3 | Asian | 1074694 | 783 | 4836634 | 16.19 | 1.03 | (0.95, 1.11) | 1.65 | (1.52, 1.78) | 1.45 | (1.34, 1.57) | 2.66 |
| MI | Easing 3 | Black | 369668 | 115 | 1661334 | 6.92 | 0.46 | (0.38, 0.55) | 0.69 | (0.58, 0.84) | 0.58 | (0.48, 0.7) | 1.68 |
| MI | Easing 3 | Mixed | 197112 | 53 | 885392 | 5.99 | 0.39 | (0.3, 0.51) | 0.81 | (0.62, 1.07) | 0.74 | (0.57, 0.97) | 0.95 |
| MI | Easing 3 | Other | 304150 | 98 | 1360084 | 7.21 | 0.48 | (0.39, 0.59) | 0.89 | (0.73, 1.09) | 0.84 | (0.69, 1.03) | 2.08 |
| DKA | Pre | White | 841518 | 4898 | 21022238 | 23.3 | Reference |  |  |  |  |  |  |
| DKA | Pre | Asian | 115505 | 188 | 2892286 | 6.5 | 0.26 | (0.23, 0.31) | 0.19 | (0.16, 0.22) | 0.17 | (0.15, 0.2) |  |
| DKA | Pre | Black | 28706 | 121 | 717714 | 16.86 | 0.67 | (0.55, 0.8) | 0.5 | (0.41, 0.6) | 0.45 | (0.37, 0.54) |  |
| DKA | Pre | Mixed | 9109 | 53 | 227450 | 23.3 | 0.96 | (0.73, 1.26) | 0.6 | (0.46, 0.79) | 0.56 | (0.43, 0.73) |  |
| DKA | Pre | Other | 12222 | 34 | 305866 | 11.12 | 0.45 | (0.32, 0.63) | 0.36 | (0.26, 0.51) | 0.34 | (0.24, 0.48) |  |
| DKA | Pandemic | White | 995361 | 5666 | 24030509 | 23.58 | Reference |  |  |  |  |  | 0.28 |
| DKA | Pandemic | Asian | 142353 | 244 | 3500862 | 6.97 | 0.29 | (0.25, 0.33) | 0.21 | (0.19, 0.24) | 0.2 | (0.17, 0.22) | 0.47 |
| DKA | Pandemic | Black | 36530 | 214 | 887198 | 24.12 | 0.97 | (0.84, 1.11) | 0.75 | (0.65, 0.87) | 0.67 | (0.58, 0.77) | 7.26 |
| DKA | Pandemic | Mixed | 11738 | 65 | 286457 | 22.69 | 0.93 | (0.73, 1.19) | 0.61 | (0.48, 0.78) | 0.56 | (0.44, 0.72) | -0.61 |
| DKA | Pandemic | Other | 16225 | 43 | 394708 | 10.89 | 0.44 | (0.32, 0.59) | 0.35 | (0.26, 0.48) | 0.33 | (0.25, 0.45) | -0.23 |
| DKA | Wave 1 | White | 995392 | 588 | 2242264 | 26.22 | Reference |  |  |  |  |  | 2.92 |
| DKA | Wave 1 | Asian | 142342 | 23 | 321261 | 7.16 | 0.28 | (0.18, 0.42) | 0.2 | (0.13, 0.3) | 0.17 | (0.11, 0.27) | 0.66 |
| DKA | Wave 1 | Black | 36526 | 21 | 82234 | 25.54 | 0.98 | (0.62, 1.55) | 0.74 | (0.47, 1.16) | 0.61 | (0.39, 0.96) | 8.68 |
| DKA | Wave 1 | Mixed | redacted |  |  |  |  |  |  |  |  |  |  |
| DKA | Wave 1 | Other | redacted |  |  |  |  |  |  |  |  |  |  |
| DKA | Easing 1 | White | 992829 | 891 | 3223673 | 27.64 | Reference |  |  |  |  |  | 4.34 |
| DKA | Easing 1 | Asian | 142905 | 31 | 465180 | 6.66 | 0.23 | (0.16, 0.33) | 0.16 | (0.11, 0.24) | 0.15 | (0.1, 0.21) | 0.16 |
| DKA | Easing 1 | Black | 36652 | 37 | 119132 | 31.06 | 1.01 | (0.72, 1.43) | 0.78 | (0.56, 1.1) | 0.68 | (0.48, 0.95) | 14.20 |
| DKA | Easing 1 | Mixed | redacted |  |  |  |  |  |  |  |  |  |  |
| DKA | Easing 1 | Other | redacted |  |  |  |  |  |  |  |  |  |  |
| DKA | Wave 2 | White | 993240 | 2005 | 7442036 | 26.94 | Reference |  |  |  |  |  | 3.64 |
| DKA | Wave 2 | Asian | 143331 | 89 | 1079487 | 8.24 | 0.3 | (0.24, 0.38) | 0.22 | (0.18, 0.28) | 0.2 | (0.16, 0.25) | 1.74 |
| DKA | Wave 2 | Black | 36803 | 65 | 276312 | 23.52 | 0.87 | (0.67, 1.12) | 0.67 | (0.52, 0.86) | 0.58 | (0.45, 0.74) | 6.66 |
| DKA | Wave 2 | Mixed | 11829 | 33 | 88847 | 37.14 | 1.37 | (0.97, 1.94) | 0.87 | (0.61, 1.22) | 0.79 | (0.56, 1.12) | 13.84 |
| DKA | Wave 2 | Other | 16467 | 13 | 123610 | 10.52 | 0.38 | (0.22, 0.67) | 0.3 | (0.17, 0.52) | 0.28 | (0.16, 0.49) | -0.60 |
| DKA | Easing 2 | White | 997807 | 363 | 1062042 | 34.18 | Reference |  |  |  |  |  | 10.88 |
| DKA | Easing 2 | Asian | 144411 | 17 | 153794 | 11.05 | 0.27 | (0.16, 0.45) | 0.21 | (0.13, 0.34) | 0.18 | (0.11, 0.3) | 4.55 |
| DKA | Easing 2 | Black | 37160 | 13 | 39575 | 32.85 | 0.79 | (0.44, 1.4) | 0.62 | (0.35, 1.1) | 0.53 | (0.3, 0.95) | 15.99 |
| DKA | Easing 2 | Mixed | redacted |  |  |  |  |  |  |  |  |  |  |
| DKA | Easing 2 | Other | redacted |  |  |  |  |  |  |  |  |  |  |
| DKA | Wave 3 | White | 999832 | 1974 | 6568038 | 30.05 | Reference |  |  |  |  |  | 6.75 |
| DKA | Wave 3 | Asian | 144709 | 84 | 955023 | 8.8 | 0.29 | (0.23, 0.37) | 0.22 | (0.17, 0.27) | 0.2 | (0.16, 0.25) | 2.30 |
| DKA | Wave 3 | Black | 37309 | 70 | 245487 | 28.51 | 0.92 | (0.72, 1.17) | 0.71 | (0.56, 0.91) | 0.63 | (0.49, 0.8) | 11.65 |
| DKA | Wave 3 | Mixed | 12024 | 25 | 79183 | 31.57 | 1.04 | (0.7, 1.54) | 0.65 | (0.44, 0.97) | 0.6 | (0.41, 0.9) | 8.27 |
| DKA | Wave 3 | Other | 16735 | 18 | 110066 | 16.35 | 0.54 | (0.34, 0.86) | 0.42 | (0.27, 0.68) | 0.4 | (0.25, 0.64) | 5.23 |
| DKA | Easing 3 | White | 1004850 | 1402 | 4506363 | 31.11 | Reference |  |  |  |  |  | 7.81 |
| DKA | Easing 3 | Asian | 146317 | 65 | 658544 | 9.87 | 0.3 | (0.23, 0.39) | 0.23 | (0.17, 0.29) | 0.21 | (0.16, 0.27) | 3.37 |
| DKA | Easing 3 | Black | 37795 | 55 | 169620 | 32.43 | 0.95 | (0.72, 1.26) | 0.74 | (0.56, 0.98) | 0.67 | (0.51, 0.89) | 15.57 |
| DKA | Easing 3 | Mixed | 12233 | 17 | 54888 | 30.97 | 0.93 | (0.58, 1.51) | 0.6 | (0.37, 0.97) | 0.57 | (0.35, 0.92) | 7.67 |
| DKA | Easing 3 | Other | 16941 | 13 | 76042 | 17.1 | 0.5 | (0.29, 0.88) | 0.4 | (0.23, 0.7) | 0.38 | (0.22, 0.66) | 5.98 |
| T1 DM | Pre | White | 87911 | 5492 | 2126366 | 258.28 | Reference |  |  |  |  |  |  |
| T1 DM | Pre | Asian | 5914 | 141 | 146226 | 96.43 | 0.33 | (0.28, 0.39) | 0.36 | (0.31, 0.43) | 0.31 | (0.26, 0.37) |  |
| T1 DM | Pre | Black | 2450 | 105 | 59963 | 175.11 | 0.59 | (0.49, 0.72) | 0.62 | (0.51, 0.76) | 0.5 | (0.41, 0.62) |  |
| T1 DM | Pre | Mixed | 956 | 70 | 22919 | 305.42 | 1.09 | (0.86, 1.39) | 0.93 | (0.74, 1.18) | 0.82 | (0.65, 1.04) |  |
| T1 DM | Pre | Other | 949 | 40 | 23225 | 172.23 | 0.59 | (0.43, 0.81) | 0.57 | (0.42, 0.79) | 0.52 | (0.38, 0.71) |  |
| T1 DM | Pandemic | White | 100454 | 5530 | 2385604 | 231.81 | Reference |  |  |  |  |  | -26.47 |
| T1 DM | Pandemic | Asian | 7274 | 143 | 175440 | 81.51 | 0.31 | (0.26, 0.37) | 0.34 | (0.29, 0.41) | 0.29 | (0.24, 0.34) | -14.92 |
| T1 DM | Pandemic | Black | 3004 | 125 | 70660 | 176.9 | 0.68 | (0.57, 0.81) | 0.7 | (0.59, 0.85) | 0.55 | (0.46, 0.67) | 1.79 |
| T1 DM | Pandemic | Mixed | 1180 | 63 | 27871 | 226.04 | 0.92 | (0.71, 1.18) | 0.79 | (0.62, 1.02) | 0.68 | (0.53, 0.88) | -79.38 |
| T1 DM | Pandemic | Other | 1255 | 52 | 29573 | 175.84 | 0.68 | (0.52, 0.9) | 0.65 | (0.5, 0.86) | 0.58 | (0.44, 0.77) | 3.61 |
| T1 DM | Wave 1 | White | 100463 | 616 | 226053 | 272.5 | Reference |  |  |  |  |  | 14.22 |
| T1 DM | Wave 1 | Asian | 7275 | 13 | 16397 | 79.28 | 0.31 | (0.18, 0.54) | 0.34 | (0.19, 0.59) | 0.27 | (0.15, 0.47) | -17.15 |
| T1 DM | Wave 1 | Black | 3001 | 11 | 6726 | 163.55 | 0.64 | (0.35, 1.18) | 0.66 | (0.36, 1.22) | 0.48 | (0.26, 0.89) | -11.56 |
| T1 DM | Wave 1 | Mixed | redacted |  |  |  |  |  |  |  |  |  |  |
| T1 DM | Wave 1 | Other | redacted |  |  |  |  |  |  |  |  |  |  |
| T1 DM | Easing 1 | White | 100755 | 1042 | 325820 | 319.81 | Reference |  |  |  |  |  | 61.53 |
| T1 DM | Easing 1 | Asian | 7311 | 23 | 23742 | 96.87 | 0.26 | (0.17, 0.4) | 0.3 | (0.19, 0.45) | 0.25 | (0.16, 0.38) | 0.44 |
| T1 DM | Easing 1 | Black | 3027 | 29 | 9786 | 296.35 | 0.8 | (0.55, 1.17) | 0.83 | (0.57, 1.22) | 0.66 | (0.45, 0.96) | 121.24 |
| T1 DM | Easing 1 | Mixed | 1194 | 14 | 3863 | 362.4 | 1.04 | (0.61, 1.76) | 0.88 | (0.52, 1.49) | 0.76 | (0.45, 1.3) | 56.98 |
| T1 DM | Easing 1 | Other | redacted |  |  |  |  |  |  |  |  |  |  |
| T1 DM | Wave 2 | White | 100749 | 2105 | 749144 | 280.99 | Reference |  |  |  |  |  | 22.71 |
| T1 DM | Wave 2 | Asian | 7314 | 50 | 54660 | 91.47 | 0.29 | (0.22, 0.39) | 0.33 | (0.24, 0.43) | 0.27 | (0.2, 0.35) | -4.96 |
| T1 DM | Wave 2 | Black | 3052 | 46 | 22633 | 203.24 | 0.65 | (0.48, 0.88) | 0.67 | (0.5, 0.91) | 0.51 | (0.38, 0.69) | 28.13 |
| T1 DM | Wave 2 | Mixed | 1187 | 30 | 8769 | 342.1 | 1.16 | (0.81, 1.67) | 0.98 | (0.68, 1.41) | 0.83 | (0.58, 1.19) | 36.68 |
| T1 DM | Wave 2 | Other | 1263 | 15 | 9374 | 160.02 | 0.52 | (0.31, 0.87) | 0.5 | (0.3, 0.83) | 0.44 | (0.26, 0.73) | -12.21 |
| T1 DM | Easing 2 | White | 100533 | 408 | 106809 | 381.99 | Reference |  |  |  |  |  | 123.71 |
| T1 DM | Easing 2 | Asian | 7334 | 14 | 7801 | 179.46 | 0.43 | (0.25, 0.74) | 0.47 | (0.27, 0.82) | 0.39 | (0.22, 0.67) | 83.03 |
| T1 DM | Easing 2 | Black | redacted |  |  |  |  |  |  |  |  |  |  |
| T1 DM | Easing 2 | Mixed | redacted |  |  |  |  |  |  |  |  |  |  |
| T1 DM | Easing 2 | Other | redacted |  |  |  |  |  |  |  |  |  |  |
| T1 DM | Wave 3 | White | 100692 | 1975 | 656541 | 300.82 | Reference |  |  |  |  |  | 42.54 |
| T1 DM | Wave 3 | Asian | 7359 | 58 | 48340 | 119.98 | 0.38 | (0.29, 0.49) | 0.41 | (0.32, 0.54) | 0.35 | (0.27, 0.46) | 23.55 |
| T1 DM | Wave 3 | Black | 3075 | 63 | 19944 | 315.88 | 0.98 | (0.76, 1.27) | 1.01 | (0.78, 1.31) | 0.81 | (0.62, 1.06) | 140.77 |
| T1 DM | Wave 3 | Mixed | 1172 | 27 | 7621 | 354.27 | 1.14 | (0.78, 1.68) | 0.94 | (0.64, 1.38) | 0.83 | (0.57, 1.22) | 48.85 |
| T1 DM | Wave 3 | Other | 1257 | 17 | 8156 | 208.44 | 0.66 | (0.4, 1.06) | 0.62 | (0.38, 1) | 0.55 | (0.34, 0.9) | 36.21 |
| T1 DM | Easing 3 | White | 100982 | 1461 | 450456 | 324.34 | Reference |  |  |  |  |  | 66.06 |
| T1 DM | Easing 3 | Asian | 7439 | 46 | 33317 | 138.07 | 0.38 | (0.28, 0.52) | 0.42 | (0.31, 0.56) | 0.35 | (0.26, 0.47) | 41.64 |
| T1 DM | Easing 3 | Black | 3126 | 33 | 13941 | 236.72 | 0.65 | (0.46, 0.92) | 0.66 | (0.47, 0.95) | 0.52 | (0.36, 0.74) | 61.61 |
| T1 DM | Easing 3 | Mixed | 1196 | 18 | 5313 | 338.81 | 0.98 | (0.61, 1.56) | 0.8 | (0.5, 1.28) | 0.7 | (0.44, 1.12) | 33.39 |
| T1 DM | Easing 3 | Other | 1243 | 16 | 5550 | 288.3 | 0.81 | (0.49, 1.33) | 0.75 | (0.46, 1.24) | 0.66 | (0.4, 1.1) | 116.07 |
| T2 DM | Pre | White | 791860 | 8195 | 19744444 | 41.51 | Reference |  |  |  |  |  |  |
| T2 DM | Pre | Asian | 114073 | 783 | 2849512 | 27.48 | 0.63 | (0.58, 0.68) | 0.61 | (0.57, 0.66) | 0.55 | (0.5, 0.59) |  |
| T2 DM | Pre | Black | 27906 | 348 | 695157 | 50.06 | 1.1 | (0.98, 1.23) | 1.07 | (0.96, 1.2) | 0.85 | (0.76, 0.95) |  |
| T2 DM | Pre | Mixed | 8600 | 76 | 214680 | 35.4 | 0.79 | (0.63, 1) | 0.78 | (0.62, 0.98) | 0.67 | (0.53, 0.84) |  |
| T2 DM | Pre | Other | 11805 | 87 | 294856 | 29.51 | 0.67 | (0.54, 0.83) | 0.66 | (0.53, 0.82) | 0.59 | (0.48, 0.74) |  |
| T2 DM | Pandemic | White | 941455 | 11040 | 22648478 | 48.74 | Reference |  |  |  |  |  | 7.23 |
| T2 DM | Pandemic | Asian | 140656 | 892 | 3452607 | 25.84 | 0.52 | (0.48, 0.56) | 0.52 | (0.49, 0.56) | 0.47 | (0.43, 0.5) | -1.64 |
| T2 DM | Pandemic | Black | 35567 | 482 | 861384 | 55.96 | 1.08 | (0.98, 1.19) | 1.08 | (0.98, 1.19) | 0.86 | (0.78, 0.95) | 5.90 |
| T2 DM | Pandemic | Mixed | 11131 | 124 | 271208 | 45.72 | 0.9 | (0.75, 1.07) | 0.91 | (0.76, 1.08) | 0.78 | (0.65, 0.93) | 10.32 |
| T2 DM | Pandemic | Other | 15672 | 122 | 380633 | 32.05 | 0.63 | (0.52, 0.75) | 0.63 | (0.52, 0.75) | 0.57 | (0.47, 0.68) | 2.54 |
| T2 DM | Wave 1 | White | 941483 | 870 | 2120264 | 41.03 | Reference |  |  |  |  |  | -0.48 |
| T2 DM | Wave 1 | Asian | 140644 | 57 | 317405 | 17.96 | 0.45 | (0.34, 0.6) | 0.44 | (0.33, 0.59) | 0.38 | (0.29, 0.51) | -9.52 |
| T2 DM | Wave 1 | Black | 35564 | 40 | 80057 | 49.96 | 1.2 | (0.86, 1.67) | 1.17 | (0.84, 1.63) | 0.88 | (0.63, 1.22) | -0.10 |
| T2 DM | Wave 1 | Mixed | redacted |  |  |  |  |  |  |  |  |  |  |
| T2 DM | Wave 1 | Other | 15687 | 11 | 35357 | 31.11 | 0.74 | (0.4, 1.35) | 0.73 | (0.4, 1.33) | 0.63 | (0.35, 1.16) | 1.60 |
| T2 DM | Easing 1 | White | 938717 | 1720 | 3046173 | 56.46 | Reference |  |  |  |  |  | 14.95 |
| T2 DM | Easing 1 | Asian | 141193 | 122 | 459473 | 26.55 | 0.46 | (0.38, 0.56) | 0.47 | (0.39, 0.57) | 0.42 | (0.34, 0.51) | -0.93 |
| T2 DM | Easing 1 | Black | 35668 | 77 | 115876 | 66.45 | 1.09 | (0.86, 1.38) | 1.1 | (0.87, 1.4) | 0.86 | (0.68, 1.1) | 16.39 |
| T2 DM | Easing 1 | Mixed | 11151 | 13 | 36284 | 35.83 | 0.6 | (0.35, 1.04) | 0.61 | (0.36, 1.06) | 0.52 | (0.3, 0.91) | 0.43 |
| T2 DM | Easing 1 | Other | 15777 | 20 | 51264 | 39.01 | 0.66 | (0.42, 1.02) | 0.66 | (0.43, 1.04) | 0.6 | (0.38, 0.93) | 9.50 |
| T2 DM | Wave 2 | White | 939511 | 3523 | 7032361 | 50.1 | Reference |  |  |  |  |  | 8.59 |
| T2 DM | Wave 2 | Asian | 141615 | 291 | 1065878 | 27.3 | 0.53 | (0.46, 0.6) | 0.52 | (0.46, 0.59) | 0.46 | (0.4, 0.52) | -0.18 |
| T2 DM | Wave 2 | Black | 35811 | 160 | 268616 | 59.56 | 1.11 | (0.94, 1.31) | 1.09 | (0.93, 1.29) | 0.85 | (0.72, 1.01) | 9.50 |
| T2 DM | Wave 2 | Mixed | 11220 | 46 | 84263 | 54.59 | 1.03 | (0.77, 1.39) | 1.03 | (0.76, 1.38) | 0.87 | (0.65, 1.17) | 19.19 |
| T2 DM | Wave 2 | Other | 15915 | 43 | 119405 | 36.01 | 0.67 | (0.49, 0.91) | 0.66 | (0.49, 0.9) | 0.59 | (0.43, 0.8) | 6.50 |
| T2 DM | Easing 2 | White | 945374 | 624 | 1006055 | 62.02 | Reference |  |  |  |  |  | 20.51 |
| T2 DM | Easing 2 | Asian | 142718 | 61 | 151971 | 40.14 | 0.63 | (0.48, 0.84) | 0.65 | (0.48, 0.86) | 0.57 | (0.43, 0.77) | 12.66 |
| T2 DM | Easing 2 | Black | 36188 | 31 | 38532 | 80.45 | 1.21 | (0.83, 1.76) | 1.22 | (0.83, 1.78) | 0.95 | (0.65, 1.39) | 30.39 |
| T2 DM | Easing 2 | Mixed | redacted |  |  |  |  |  |  |  |  |  |  |
| T2 DM | Easing 2 | Other | redacted |  |  |  |  |  |  |  |  |  |  |
| T2 DM | Wave 3 | White | 947473 | 3295 | 6218927 | 52.98 | Reference |  |  |  |  |  | 11.47 |
| T2 DM | Wave 3 | Asian | 143015 | 289 | 943099 | 30.64 | 0.57 | (0.5, 0.64) | 0.57 | (0.5, 0.65) | 0.51 | (0.45, 0.58) | 3.16 |
| T2 DM | Wave 3 | Black | 36330 | 127 | 238909 | 53.16 | 0.93 | (0.77, 1.12) | 0.94 | (0.78, 1.13) | 0.75 | (0.62, 0.9) | 3.10 |
| T2 DM | Wave 3 | Mixed | 11430 | 44 | 75226 | 58.49 | 1.04 | (0.77, 1.41) | 1.06 | (0.79, 1.43) | 0.92 | (0.68, 1.24) | 23.09 |
| T2 DM | Wave 3 | Other | 16203 | 39 | 106510 | 36.62 | 0.65 | (0.47, 0.9) | 0.66 | (0.48, 0.9) | 0.59 | (0.43, 0.82) | 7.11 |
| T2 DM | Easing 3 | White | 953549 | 2229 | 4273597 | 52.16 | Reference |  |  |  |  |  | 10.65 |
| T2 DM | Easing 3 | Asian | 144636 | 182 | 650752 | 27.97 | 0.5 | (0.43, 0.59) | 0.51 | (0.43, 0.6) | 0.46 | (0.39, 0.55) | 0.49 |
| T2 DM | Easing 3 | Black | 36828 | 113 | 165179 | 68.41 | 1.2 | (0.98, 1.46) | 1.21 | (0.99, 1.48) | 0.99 | (0.81, 1.21) | 18.35 |
| T2 DM | Easing 3 | Mixed | 11643 | 32 | 52210 | 61.29 | 1.11 | (0.78, 1.58) | 1.13 | (0.79, 1.61) | 0.99 | (0.7, 1.41) | 25.89 |
| T2 DM | Easing 3 | Other | 16434 | 13 | 73768 | 17.62 | 0.31 | (0.18, 0.54) | 0.31 | (0.18, 0.54) | 0.29 | (0.17, 0.5) | -11.89 |
| Anxiety | Pre | White | 11951915 | 4087 | 299505903 | 1.36 | Reference |  |  |  |  |  |  |
| Anxiety | Pre | Asian | 947198 | 279 | 23736235 | 1.18 | 0.88 | (0.78, 1) | 0.94 | (0.83, 1.07) | 0.8 | (0.71, 0.91) |  |
| Anxiety | Pre | Black | 323687 | 60 | 8111470 | 0.74 | 0.54 | (0.42, 0.7) | 0.57 | (0.44, 0.74) | 0.47 | (0.36, 0.6) |  |
| Anxiety | Pre | Mixed | 167862 | 42 | 4206466 | 1 | 0.74 | (0.55, 1.01) | 0.79 | (0.58, 1.07) | 0.71 | (0.52, 0.96) |  |
| Anxiety | Pre | Other | 255875 | 53 | 6411343 | 0.83 | 0.66 | (0.5, 0.86) | 0.7 | (0.53, 0.92) | 0.67 | (0.51, 0.87) |  |
| Anxiety | Pandemic | White | 13007851 | 4604 | 321888666 | 1.43 | Reference |  |  |  |  |  | 0.07 |
| Anxiety | Pandemic | Asian | 1116587 | 316 | 27562426 | 1.15 | 0.87 | (0.77, 0.97) | 0.9 | (0.8, 1.01) | 0.78 | (0.69, 0.88) | -0.03 |
| Anxiety | Pandemic | Black | 389040 | 80 | 9540013 | 0.84 | 0.62 | (0.49, 0.77) | 0.64 | (0.51, 0.8) | 0.53 | (0.42, 0.66) | 0.10 |
| Anxiety | Pandemic | Mixed | 206060 | 68 | 5035801 | 1.35 | 1 | (0.79, 1.28) | 1.03 | (0.81, 1.31) | 0.94 | (0.74, 1.2) | 0.35 |
| Anxiety | Pandemic | Other | 334275 | 76 | 8015275 | 0.95 | 0.75 | (0.6, 0.94) | 0.78 | (0.62, 0.98) | 0.74 | (0.59, 0.93) | 0.12 |
| Anxiety | Wave 1 | White | 13008075 | 365 | 29413099 | 1.24 | Reference |  |  |  |  |  | -0.12 |
| Anxiety | Wave 1 | Asian | 1116590 | 27 | 2525244 | 1.07 | 1.06 | (0.71, 1.59) | 1.11 | (0.74, 1.67) | 0.94 | (0.62, 1.42) | -0.11 |
| Anxiety | Wave 1 | Black | redacted |  |  |  |  |  |  |  |  |  |  |
| Anxiety | Wave 1 | Mixed | redacted |  |  |  |  |  |  |  |  |  |  |
| Anxiety | Wave 1 | Other | redacted |  |  |  |  |  |  |  |  |  |  |
| Anxiety | Easing 1 | White | 13059230 | 725 | 42519578 | 1.71 | Reference |  |  |  |  |  | 0.35 |
| Anxiety | Easing 1 | Asian | 1128788 | 45 | 3674977 | 1.22 | 0.8 | (0.58, 1.09) | 0.85 | (0.62, 1.17) | 0.72 | (0.52, 0.98) | 0.04 |
| Anxiety | Easing 1 | Black | redacted |  |  |  |  |  |  |  |  |  |  |
| Anxiety | Easing 1 | Mixed | 208793 | 15 | 678914 | 2.21 | 1.39 | (0.83, 2.32) | 1.48 | (0.88, 2.48) | 1.32 | (0.79, 2.21) | 1.21 |
| Anxiety | Easing 1 | Other | 338288 | 12 | 1098296 | 1.09 | 0.75 | (0.42, 1.33) | 0.81 | (0.45, 1.43) | 0.76 | (0.43, 1.35) | 0.26 |
| Anxiety | Wave 2 | White | 12967234 | 1399 | 97842210 | 1.43 | Reference |  |  |  |  |  | 0.07 |
| Anxiety | Wave 2 | Asian | 1122609 | 82 | 8461143 | 0.97 | 0.73 | (0.58, 0.92) | 0.78 | (0.61, 0.98) | 0.68 | (0.54, 0.86) | -0.21 |
| Anxiety | Wave 2 | Black | 390037 | 29 | 2935073 | 0.99 | 0.74 | (0.51, 1.07) | 0.78 | (0.54, 1.13) | 0.65 | (0.45, 0.95) | 0.25 |
| Anxiety | Wave 2 | Mixed | 207205 | 27 | 1555417 | 1.74 | 1.3 | (0.88, 1.9) | 1.36 | (0.93, 2) | 1.26 | (0.86, 1.85) | 0.74 |
| Anxiety | Wave 2 | Other | 332902 | 17 | 2485666 | 0.68 | 0.54 | (0.33, 0.88) | 0.58 | (0.36, 0.93) | 0.55 | (0.34, 0.89) | -0.15 |
| Anxiety | Easing 2 | White | 12640489 | 237 | 13462945 | 1.76 | Reference |  |  |  |  |  | 0.40 |
| Anxiety | Easing 2 | Asian | 1095138 | 19 | 1166268 | 1.63 | 0.97 | (0.59, 1.58) | 1.03 | (0.63, 1.68) | 0.89 | (0.54, 1.46) | 0.45 |
| Anxiety | Easing 2 | Black | redacted |  |  |  |  |  |  |  |  |  |  |
| Anxiety | Easing 2 | Mixed | redacted |  |  |  |  |  |  |  |  |  |  |
| Anxiety | Easing 2 | Other | redacted |  |  |  |  |  |  |  |  |  |  |
| Anxiety | Wave 3 | White | 12602332 | 1185 | 83187132 | 1.42 | Reference |  |  |  |  |  | 0.06 |
| Anxiety | Wave 3 | Asian | 1091692 | 94 | 7195031 | 1.31 | 1.03 | (0.83, 1.29) | 1.05 | (0.84, 1.31) | 0.93 | (0.75, 1.17) | 0.13 |
| Anxiety | Wave 3 | Black | 377686 | 21 | 2483064 | 0.85 | 0.62 | (0.4, 0.97) | 0.64 | (0.41, 0.99) | 0.55 | (0.35, 0.84) | 0.11 |
| Anxiety | Wave 3 | Mixed | 200227 | 15 | 1315159 | 1.14 | 0.87 | (0.52, 1.44) | 0.86 | (0.52, 1.44) | 0.8 | (0.48, 1.34) | 0.14 |
| Anxiety | Wave 3 | Other | 314768 | 22 | 2054920 | 1.07 | 0.88 | (0.58, 1.35) | 0.89 | (0.58, 1.37) | 0.85 | (0.56, 1.31) | 0.24 |
| Anxiety | Easing 3 | White | 12411761 | 691 | 55915750 | 1.24 | Reference |  |  |  |  |  | -0.12 |
| Anxiety | Easing 3 | Asian | 1074699 | 48 | 4838179 | 0.99 | 0.84 | (0.62, 1.14) | 0.85 | (0.63, 1.15) | 0.75 | (0.55, 1.02) | -0.19 |
| Anxiety | Easing 3 | Black | redacted |  |  |  |  |  |  |  |  |  |  |
| Anxiety | Easing 3 | Mixed | redacted |  |  |  |  |  |  |  |  |  |  |
| Anxiety | Easing 3 | Other | 304150 | 11 | 1360271 | 0.81 | 0.72 | (0.4, 1.31) | 0.73 | (0.4, 1.33) | 0.7 | (0.38, 1.28) | -0.02 |
| Depression | Pre | White | 11951915 | 2218 | 299528329 | 0.74 | Reference |  |  |  |  |  |  |
| Depression | Pre | Asian | 947198 | 83 | 23738551 | 0.35 | 0.5 | (0.4, 0.62) | 0.49 | (0.39, 0.62) | 0.4 | (0.32, 0.51) |  |
| Depression | Pre | Black | 323687 | 35 | 8111737 | 0.43 | 0.52 | (0.37, 0.73) | 0.53 | (0.38, 0.74) | 0.4 | (0.29, 0.56) |  |
| Depression | Pre | Mixed | 167862 | 35 | 4206541 | 0.83 | 1.08 | (0.77, 1.5) | 1.06 | (0.76, 1.48) | 0.92 | (0.66, 1.29) |  |
| Depression | Pre | Other | 255875 | 23 | 6411754 | 0.36 | 0.53 | (0.35, 0.8) | 0.53 | (0.35, 0.79) | 0.48 | (0.32, 0.73) |  |
| Depression | Pandemic | White | 13007854 | 2181 | 321918259 | 0.68 | Reference |  |  |  |  |  | -0.06 |
| Depression | Pandemic | Asian | 1116587 | 97 | 27564956 | 0.35 | 0.63 | (0.51, 0.78) | 0.68 | (0.55, 0.84) | 0.57 | (0.46, 0.7) | 0.00 |
| Depression | Pandemic | Black | 389040 | 41 | 9540463 | 0.43 | 0.65 | (0.47, 0.88) | 0.71 | (0.52, 0.96) | 0.56 | (0.41, 0.76) | 0.00 |
| Depression | Pandemic | Mixed | 206060 | 27 | 5036547 | 0.54 | 0.83 | (0.56, 1.21) | 0.9 | (0.61, 1.31) | 0.8 | (0.54, 1.17) | -0.29 |
| Depression | Pandemic | Other | 334275 | 29 | 8015812 | 0.36 | 0.64 | (0.44, 0.92) | 0.69 | (0.48, 1) | 0.64 | (0.44, 0.92) | 0.00 |
| Depression | Wave 1 | White | 13008078 | 185 | 29413291 | 0.63 | Reference |  |  |  |  |  | -0.11 |
| Depression | Wave 1 | Asian | redacted |  |  |  |  |  |  |  |  |  |  |
| Depression | Wave 1 | Black | redacted |  |  |  |  |  |  |  |  |  |  |
| Depression | Wave 1 | Mixed | redacted |  |  |  |  |  |  |  |  |  |  |
| Depression | Wave 1 | Other | redacted |  |  |  |  |  |  |  |  |  |  |
| Depression | Easing 1 | White | 13059246 | 406 | 42520051 | 0.95 | Reference |  |  |  |  |  | 0.21 |
| Depression | Easing 1 | Asian | 1128788 | 16 | 3675015 | 0.44 | 0.61 | (0.36, 1.01) | 0.63 | (0.38, 1.06) | 0.52 | (0.31, 0.87) | 0.09 |
| Depression | Easing 1 | Black | 393362 | 12 | 1279474 | 0.94 | 1.01 | (0.56, 1.8) | 1.06 | (0.59, 1.9) | 0.81 | (0.45, 1.46) | 0.51 |
| Depression | Easing 1 | Mixed | redacted |  |  |  |  |  |  |  |  |  |  |
| Depression | Easing 1 | Other | redacted |  |  |  |  |  |  |  |  |  |  |
| Depression | Wave 2 | White | 12967236 | 697 | 97844796 | 0.71 | Reference |  |  |  |  |  | -0.03 |
| Depression | Wave 2 | Asian | 1122609 | 29 | 8461282 | 0.34 | 0.55 | (0.38, 0.81) | 0.59 | (0.4, 0.86) | 0.5 | (0.34, 0.73) | -0.01 |
| Depression | Wave 2 | Black | 390037 | 12 | 2935109 | 0.41 | 0.56 | (0.31, 0.99) | 0.6 | (0.34, 1.08) | 0.49 | (0.27, 0.87) | -0.02 |
| Depression | Wave 2 | Mixed | redacted |  |  |  |  |  |  |  |  |  |  |
| Depression | Wave 2 | Other | redacted |  |  |  |  |  |  |  |  |  |  |
| Depression | Easing 2 | White | 12640487 | 108 | 13463005 | 0.8 | Reference |  |  |  |  |  | 0.06 |
| Depression | Easing 2 | Asian | redacted |  |  |  |  |  |  |  |  |  |  |
| Depression | Easing 2 | Black | redacted |  |  |  |  |  |  |  |  |  |  |
| Depression | Easing 2 | Mixed | redacted |  |  |  |  |  |  |  |  |  |  |
| Depression | Easing 2 | Other | redacted |  |  |  |  |  |  |  |  |  |  |
| Depression | Wave 3 | White | 12602338 | 504 | 83189402 | 0.61 | Reference |  |  |  |  |  | -0.13 |
| Depression | Wave 3 | Asian | 1091692 | 26 | 7195242 | 0.36 | 0.75 | (0.5, 1.12) | 0.81 | (0.54, 1.22) | 0.67 | (0.45, 1.02) | 0.01 |
| Depression | Wave 3 | Black | redacted |  |  |  |  |  |  |  |  |  |  |
| Depression | Wave 3 | Mixed | redacted |  |  |  |  |  |  |  |  |  |  |
| Depression | Wave 3 | Other | redacted |  |  |  |  |  |  |  |  |  |  |
| Depression | Easing 3 | White | 12411765 | 310 | 55916565 | 0.55 | Reference |  |  |  |  |  | -0.19 |
| Depression | Easing 3 | Asian | 1074699 | 15 | 4838248 | 0.31 | 0.6 | (0.35, 1.02) | 0.67 | (0.39, 1.14) | 0.55 | (0.32, 0.95) | -0.04 |
| Depression | Easing 3 | Black | redacted |  |  |  |  |  |  |  |  |  |  |
| Depression | Easing 3 | Mixed | redacted |  |  |  |  |  |  |  |  |  |  |
| Depression | Easing 3 | Other | redacted |  |  |  |  |  |  |  |  |  |  |
| Asthma | Pre | White | 1104943 | 14508 | 27504757 | 52.75 | Reference |  |  |  |  |  |  |
| Asthma | Pre | Asian | 72238 | 1870 | 1785399 | 104.74 | 1.78 | (1.69, 1.88) | 1.83 | (1.74, 1.93) | 1.57 | (1.49, 1.65) |  |
| Asthma | Pre | Black | 21726 | 436 | 538585 | 80.95 | 1.41 | (1.27, 1.55) | 1.4 | (1.27, 1.55) | 1.16 | (1.05, 1.28) |  |
| Asthma | Pre | Mixed | 14090 | 227 | 350142 | 64.83 | 1.16 | (1.02, 1.33) | 1.17 | (1.02, 1.33) | 1.1 | (0.96, 1.25) |  |
| Asthma | Pre | Other | 11220 | 176 | 278865 | 63.11 | 1.12 | (0.96, 1.3) | 1.14 | (0.98, 1.32) | 1.14 | (0.98, 1.33) |  |
| Asthma | Pandemic | White | 1230577 | 9804 | 30424471 | 32.22 | Reference |  |  |  |  |  | -20.53 |
| Asthma | Pandemic | Asian | 84572 | 1128 | 2090544 | 53.96 | 1.51 | (1.41, 1.61) | 1.53 | (1.43, 1.63) | 1.33 | (1.25, 1.42) | -50.78 |
| Asthma | Pandemic | Black | 25738 | 279 | 632429 | 44.12 | 1.23 | (1.09, 1.4) | 1.21 | (1.07, 1.36) | 1.02 | (0.9, 1.15) | -36.83 |
| Asthma | Pandemic | Mixed | 17059 | 155 | 418542 | 37.03 | 1.09 | (0.93, 1.27) | 1.05 | (0.9, 1.24) | 0.99 | (0.85, 1.17) | -27.80 |
| Asthma | Pandemic | Other | 13884 | 115 | 340269 | 33.8 | 0.99 | (0.82, 1.19) | 0.99 | (0.82, 1.19) | 0.99 | (0.82, 1.19) | -29.31 |
| Asthma | Wave 1 | White | 1230560 | 1011 | 2780998 | 36.35 | Reference |  |  |  |  |  | -16.40 |
| Asthma | Wave 1 | Asian | 84566 | 68 | 191126 | 35.58 | 1.02 | (0.79, 1.32) | 1.03 | (0.8, 1.34) | 0.87 | (0.67, 1.13) | -69.16 |
| Asthma | Wave 1 | Black | 25739 | 18 | 58115 | 30.97 | 0.97 | (0.6, 1.55) | 0.93 | (0.58, 1.5) | 0.77 | (0.48, 1.24) | -49.98 |
| Asthma | Wave 1 | Mixed | 17045 | 12 | 38540 | 31.14 | 0.92 | (0.52, 1.63) | 0.9 | (0.51, 1.6) | 0.86 | (0.48, 1.52) | -33.69 |
| Asthma | Wave 1 | Other | redacted |  |  |  |  |  |  |  |  |  |  |
| Asthma | Easing 1 | White | 1239227 | 1453 | 4033325 | 36.02 | Reference |  |  |  |  |  | -16.73 |
| Asthma | Easing 1 | Asian | 85465 | 110 | 278243 | 39.53 | 1.05 | (0.86, 1.29) | 1.06 | (0.87, 1.3) | 0.87 | (0.71, 1.07) | -65.21 |
| Asthma | Easing 1 | Black | 25983 | 34 | 84514 | 40.23 | 1.05 | (0.75, 1.49) | 1.02 | (0.72, 1.44) | 0.82 | (0.58, 1.16) | -40.72 |
| Asthma | Easing 1 | Mixed | 17414 | 19 | 56614 | 33.56 | 0.9 | (0.57, 1.41) | 0.86 | (0.55, 1.36) | 0.8 | (0.51, 1.27) | -31.27 |
| Asthma | Easing 1 | Other | 14127 | 16 | 45931 | 34.83 | 0.97 | (0.59, 1.6) | 0.96 | (0.58, 1.58) | 0.98 | (0.6, 1.61) | -28.28 |
| Asthma | Wave 2 | White | 1235344 | 3169 | 9316516 | 34.01 | Reference |  |  |  |  |  | -18.74 |
| Asthma | Wave 2 | Asian | 85717 | 311 | 646184 | 48.13 | 1.29 | (1.14, 1.46) | 1.29 | (1.14, 1.46) | 1.1 | (0.97, 1.24) | -56.61 |
| Asthma | Wave 2 | Black | 25961 | 101 | 195315 | 51.71 | 1.39 | (1.14, 1.71) | 1.33 | (1.09, 1.64) | 1.1 | (0.89, 1.35) | -29.24 |
| Asthma | Wave 2 | Mixed | 17437 | 42 | 130946 | 32.07 | 0.9 | (0.66, 1.22) | 0.84 | (0.62, 1.14) | 0.79 | (0.58, 1.07) | -32.76 |
| Asthma | Wave 2 | Other | 14058 | 33 | 105601 | 31.25 | 0.88 | (0.62, 1.24) | 0.86 | (0.61, 1.22) | 0.88 | (0.62, 1.24) | -31.86 |
| Asthma | Easing 2 | White | 1207813 | 677 | 1286418 | 52.63 | Reference |  |  |  |  |  | -0.12 |
| Asthma | Easing 2 | Asian | 84468 | 84 | 89957 | 93.38 | 1.49 | (1.17, 1.9) | 1.48 | (1.16, 1.89) | 1.23 | (0.96, 1.58) | -11.36 |
| Asthma | Easing 2 | Black | 25562 | 20 | 27222 | 73.47 | 1.19 | (0.75, 1.88) | 1.14 | (0.72, 1.79) | 0.92 | (0.58, 1.46) | -7.48 |
| Asthma | Easing 2 | Mixed | redacted |  |  |  |  |  |  |  |  |  |  |
| Asthma | Easing 2 | Other | redacted |  |  |  |  |  |  |  |  |  |  |
| Asthma | Wave 3 | White | 1207064 | 3813 | 7964754 | 47.87 | Reference |  |  |  |  |  | -4.88 |
| Asthma | Wave 3 | Asian | 84457 | 482 | 556459 | 86.62 | 1.51 | (1.37, 1.68) | 1.53 | (1.38, 1.69) | 1.33 | (1.2, 1.47) | -18.12 |
| Asthma | Wave 3 | Black | 25521 | 120 | 168043 | 71.41 | 1.25 | (1.04, 1.51) | 1.21 | (1, 1.46) | 1.03 | (0.85, 1.24) | -9.54 |
| Asthma | Wave 3 | Mixed | 17084 | 75 | 112349 | 66.76 | 1.25 | (0.99, 1.58) | 1.18 | (0.94, 1.49) | 1.12 | (0.89, 1.41) | 1.93 |
| Asthma | Wave 3 | Other | 13693 | 52 | 89958 | 57.8 | 1.04 | (0.79, 1.38) | 1.03 | (0.78, 1.36) | 1.03 | (0.78, 1.36) | -5.31 |
| Asthma | Easing 3 | White | 1197790 | 2330 | 5394041 | 43.2 | Reference |  |  |  |  |  | -9.55 |
| Asthma | Easing 3 | Asian | 84615 | 327 | 380842 | 85.86 | 1.7 | (1.5, 1.92) | 1.73 | (1.52, 1.96) | 1.5 | (1.32, 1.7) | -18.88 |
| Asthma | Easing 3 | Black | 25401 | 76 | 114225 | 66.54 | 1.32 | (1.04, 1.67) | 1.29 | (1.02, 1.63) | 1.08 | (0.86, 1.37) | -14.41 |
| Asthma | Easing 3 | Mixed | 16959 | 46 | 76258 | 60.32 | 1.28 | (0.95, 1.72) | 1.26 | (0.94, 1.69) | 1.18 | (0.88, 1.58) | -4.51 |
| Asthma | Easing 3 | Other | 13585 | 35 | 61096 | 57.29 | 1.2 | (0.86, 1.69) | 1.21 | (0.86, 1.7) | 1.2 | (0.85, 1.68) | -5.82 |
| COPD | Pre | White | 409471 | 34264 | 9831716 | 348.5 | Reference |  |  |  |  |  |  |
| COPD | Pre | Asian | 8290 | 531 | 200489 | 264.85 | 0.73 | (0.67, 0.79) | 0.77 | (0.7, 0.84) | 0.73 | (0.67, 0.8) |  |
| COPD | Pre | Black | 2556 | 144 | 62265 | 231.27 | 0.64 | (0.54, 0.75) | 0.68 | (0.58, 0.8) | 0.63 | (0.53, 0.74) |  |
| COPD | Pre | Mixed | 1379 | 107 | 33202 | 322.27 | 0.91 | (0.76, 1.11) | 0.98 | (0.81, 1.19) | 0.96 | (0.79, 1.16) |  |
| COPD | Pre | Other | 1851 | 123 | 44939 | 273.7 | 0.78 | (0.65, 0.93) | 0.82 | (0.69, 0.98) | 0.81 | (0.68, 0.97) |  |
| COPD | Pandemic | White | 497200 | 33511 | 11478967 | 291.93 | Reference |  |  |  |  |  | -56.57 |
| COPD | Pandemic | Asian | 10220 | 443 | 240147 | 184.47 | 0.61 | (0.56, 0.68) | 0.65 | (0.59, 0.71) | 0.6 | (0.54, 0.66) | -80.38 |
| COPD | Pandemic | Black | 3277 | 138 | 77025 | 179.16 | 0.6 | (0.51, 0.71) | 0.65 | (0.55, 0.77) | 0.58 | (0.49, 0.69) | -52.11 |
| COPD | Pandemic | Mixed | 1766 | 92 | 41773 | 220.24 | 0.76 | (0.62, 0.93) | 0.83 | (0.68, 1.02) | 0.81 | (0.66, 1) | -102.03 |
| COPD | Pandemic | Other | 2405 | 97 | 56013 | 173.17 | 0.6 | (0.49, 0.73) | 0.64 | (0.52, 0.78) | 0.62 | (0.51, 0.76) | -100.53 |
| COPD | Wave 1 | White | 497196 | 3088 | 1114997 | 276.95 | Reference |  |  |  |  |  | -71.55 |
| COPD | Wave 1 | Asian | 10221 | 32 | 22957 | 139.39 | 0.49 | (0.34, 0.69) | 0.5 | (0.35, 0.72) | 0.46 | (0.32, 0.65) | -125.46 |
| COPD | Wave 1 | Black | redacted |  |  |  |  |  |  |  |  |  |  |
| COPD | Wave 1 | Mixed | redacted |  |  |  |  |  |  |  |  |  |  |
| COPD | Wave 1 | Other | redacted |  |  |  |  |  |  |  |  |  |  |
| COPD | Easing 1 | White | 493737 | 5495 | 1591832 | 345.2 | Reference |  |  |  |  |  | -3.30 |
| COPD | Easing 1 | Asian | 10226 | 64 | 33100 | 193.36 | 0.53 | (0.41, 0.68) | 0.55 | (0.43, 0.71) | 0.51 | (0.39, 0.65) | -71.49 |
| COPD | Easing 1 | Black | 3267 | 22 | 10548 | 208.57 | 0.56 | (0.36, 0.85) | 0.6 | (0.39, 0.91) | 0.54 | (0.35, 0.82) | -22.70 |
| COPD | Easing 1 | Mixed | 1768 | 14 | 5735 | 244.1 | 0.68 | (0.4, 1.15) | 0.74 | (0.44, 1.26) | 0.72 | (0.43, 1.22) | -78.17 |
| COPD | Easing 1 | Other | 2407 | 16 | 7765 | 206.06 | 0.57 | (0.35, 0.93) | 0.6 | (0.36, 0.98) | 0.59 | (0.36, 0.96) | -67.64 |
| COPD | Wave 2 | White | 489153 | 10994 | 3607014 | 304.8 | Reference |  |  |  |  |  | -43.70 |
| COPD | Wave 2 | Asian | 10158 | 146 | 75174 | 194.22 | 0.62 | (0.53, 0.73) | 0.65 | (0.55, 0.77) | 0.6 | (0.51, 0.71) | -70.63 |
| COPD | Wave 2 | Black | 3229 | 50 | 23911 | 209.11 | 0.68 | (0.51, 0.89) | 0.72 | (0.55, 0.96) | 0.65 | (0.49, 0.86) | -22.16 |
| COPD | Wave 2 | Mixed | 1784 | 30 | 13200 | 227.26 | 0.75 | (0.52, 1.07) | 0.83 | (0.58, 1.18) | 0.81 | (0.56, 1.15) | -95.01 |
| COPD | Wave 2 | Other | 2372 | 32 | 17549 | 182.35 | 0.61 | (0.43, 0.86) | 0.64 | (0.45, 0.91) | 0.63 | (0.44, 0.89) | -91.35 |
| COPD | Easing 2 | White | 478271 | 2532 | 507374 | 499.04 | Reference |  |  |  |  |  | 150.54 |
| COPD | Easing 2 | Asian | 10036 | 30 | 10656 | 281.52 | 0.56 | (0.39, 0.8) | 0.6 | (0.42, 0.87) | 0.55 | (0.38, 0.79) | 16.67 |
| COPD | Easing 2 | Black | 3242 | 11 | 3446 | 319.17 | 0.64 | (0.35, 1.16) | 0.7 | (0.38, 1.27) | 0.62 | (0.34, 1.13) | 87.90 |
| COPD | Easing 2 | Mixed | redacted |  |  |  |  |  |  |  |  |  |  |
| COPD | Easing 2 | Other | redacted |  |  |  |  |  |  |  |  |  |  |
| COPD | Wave 3 | White | 477395 | 12942 | 3083669 | 419.69 | Reference |  |  |  |  |  | 71.19 |
| COPD | Wave 3 | Asian | 10023 | 181 | 65161 | 277.77 | 0.64 | (0.55, 0.74) | 0.68 | (0.59, 0.79) | 0.63 | (0.54, 0.73) | 12.92 |
| COPD | Wave 3 | Black | 3229 | 51 | 21022 | 242.61 | 0.57 | (0.43, 0.75) | 0.62 | (0.47, 0.81) | 0.55 | (0.42, 0.72) | 11.34 |
| COPD | Wave 3 | Mixed | 1809 | 30 | 11751 | 255.29 | 0.61 | (0.43, 0.88) | 0.68 | (0.47, 0.97) | 0.66 | (0.46, 0.95) | -66.98 |
| COPD | Wave 3 | Other | 2422 | 42 | 15769 | 266.35 | 0.65 | (0.48, 0.88) | 0.69 | (0.51, 0.94) | 0.69 | (0.51, 0.93) | -7.35 |
| COPD | Easing 3 | White | 472883 | 9016 | 2093051 | 430.76 | Reference |  |  |  |  |  | 82.26 |
| COPD | Easing 3 | Asian | 9987 | 132 | 44390 | 297.37 | 0.67 | (0.56, 0.79) | 0.71 | (0.6, 0.85) | 0.65 | (0.55, 0.78) | 32.52 |
| COPD | Easing 3 | Black | 3230 | 41 | 14355 | 285.62 | 0.66 | (0.48, 0.9) | 0.72 | (0.53, 0.98) | 0.64 | (0.47, 0.87) | 54.35 |
| COPD | Easing 3 | Mixed | 1849 | 31 | 8222 | 377.01 | 0.89 | (0.63, 1.27) | 1 | (0.7, 1.43) | 0.98 | (0.69, 1.39) | 54.74 |
| COPD | Easing 3 | Other | 2428 | 31 | 10808 | 286.83 | 0.69 | (0.49, 0.99) | 0.75 | (0.52, 1.06) | 0.74 | (0.52, 1.05) | 13.13 |

### Figure 2: Monthly rate of change in clinical monitoring by ethnic group.

**Blood pressure monitoring in SMI subpopulation**

**Blood pressure monitoring in CVD subpopulation**


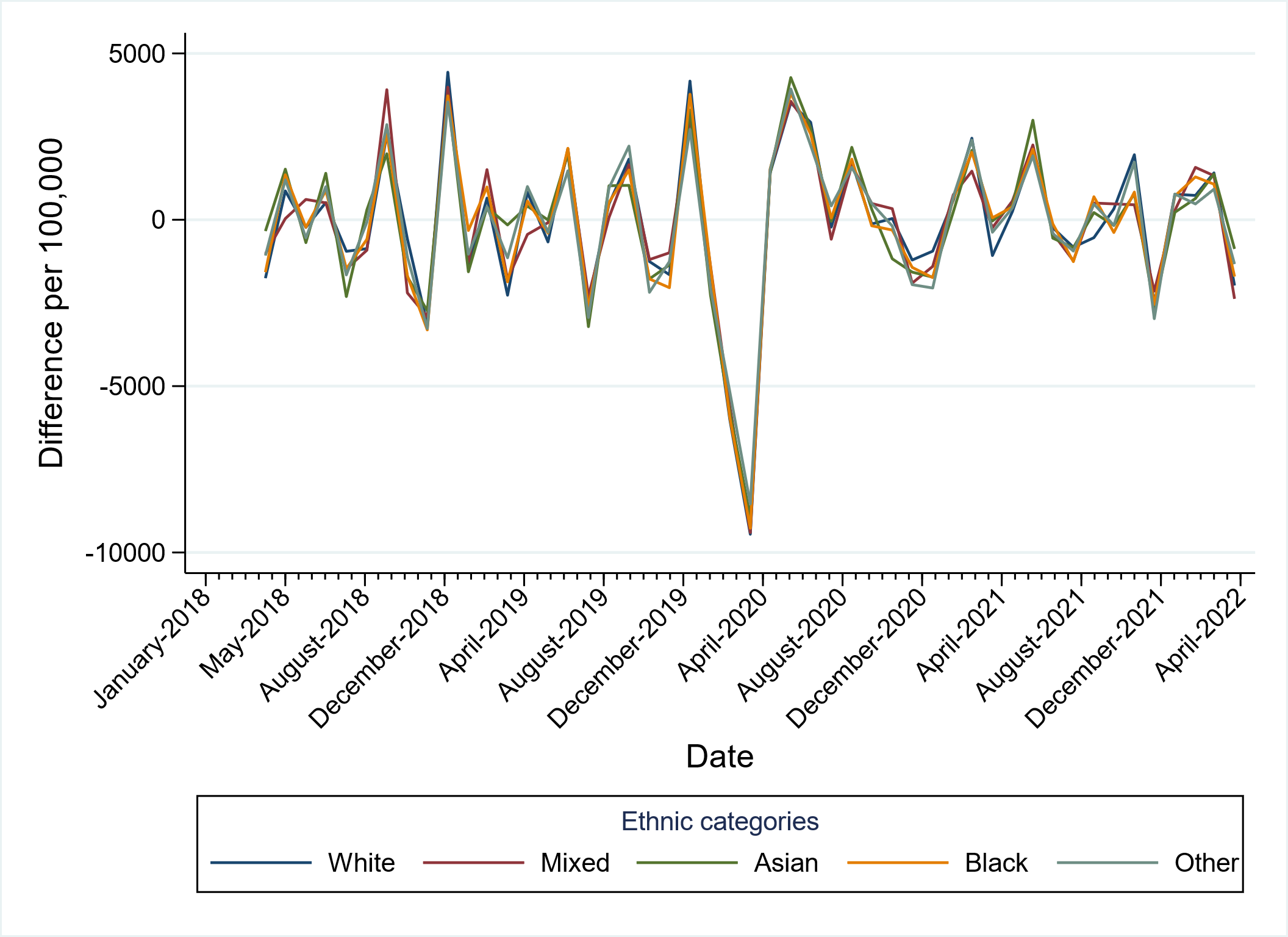

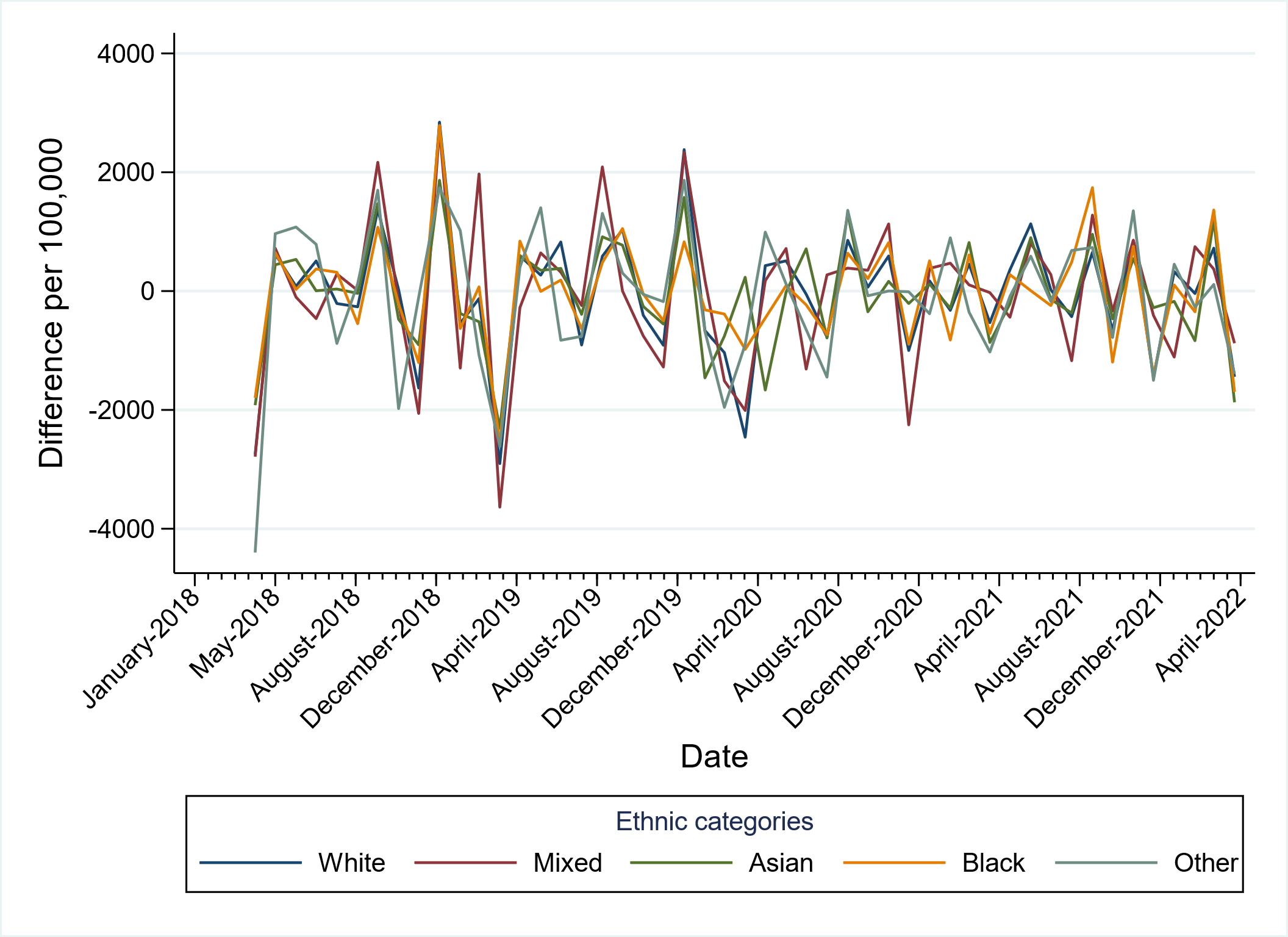

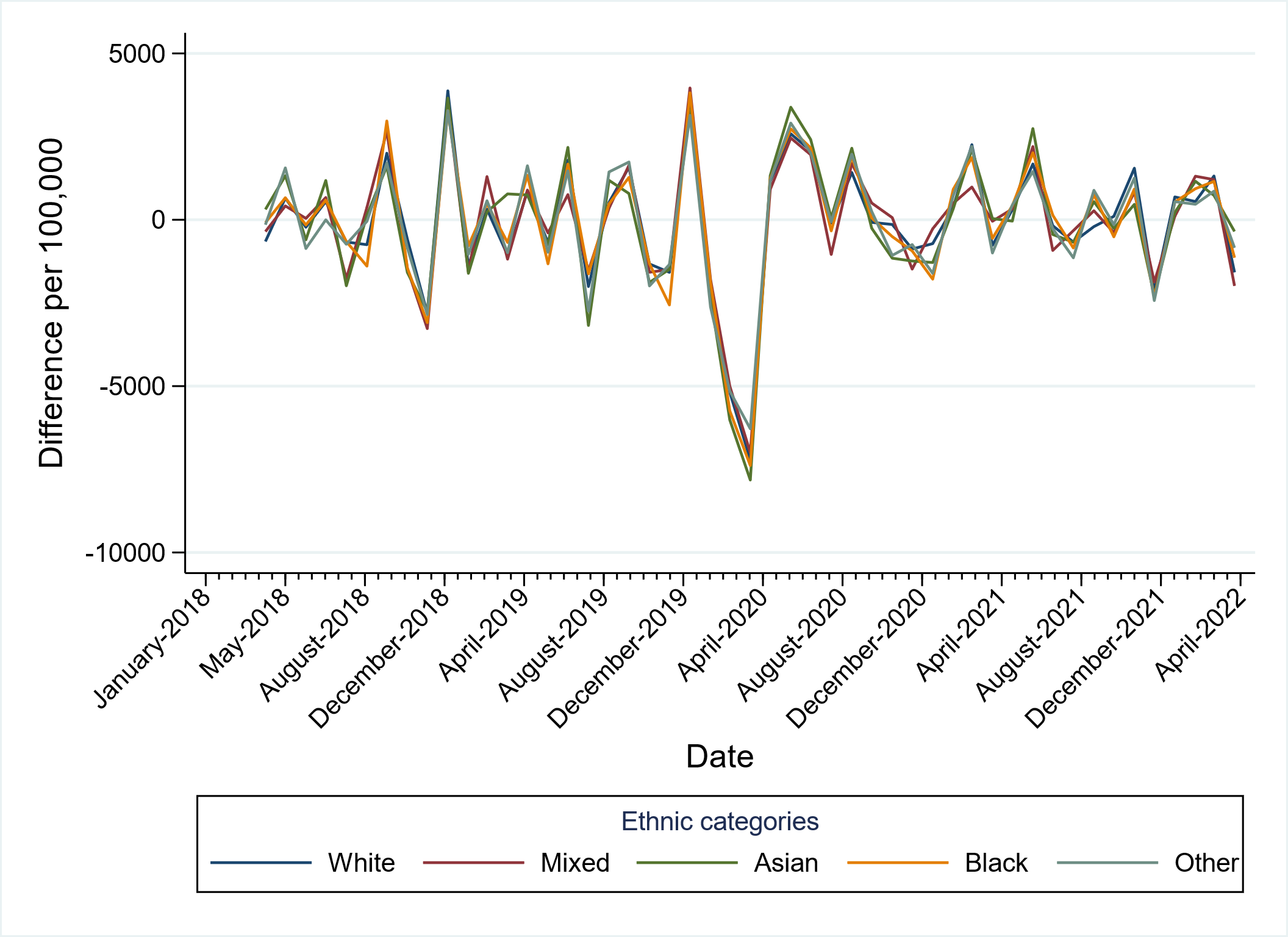

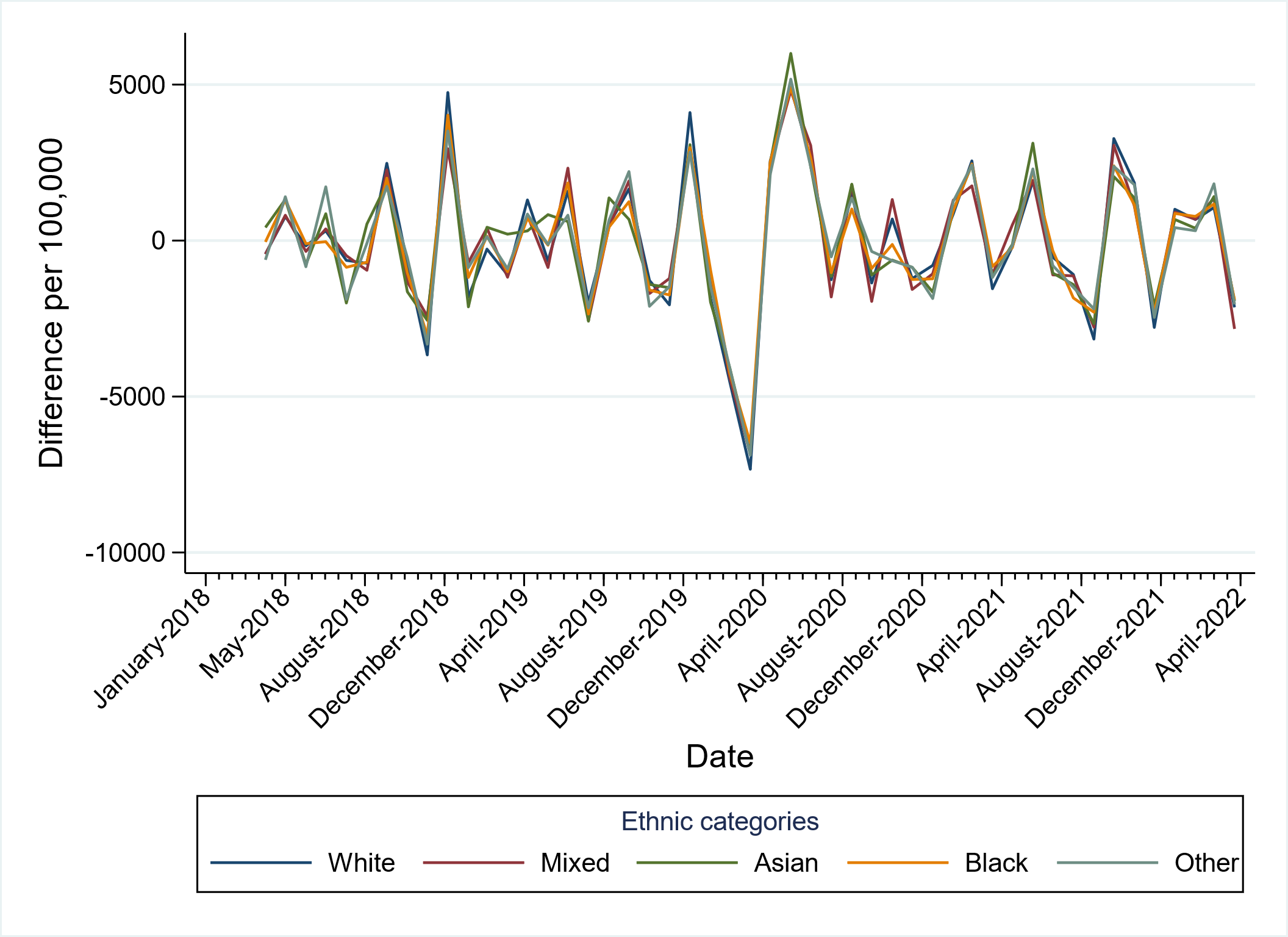


**Annual asthma reviews in asthma subpopulation**

**Annual COPD reviews in COPD subpopulation**

**Blood pressure monitoring in diabetes subpopulation**

**HbA1c measurements in diabetes subpopulation**


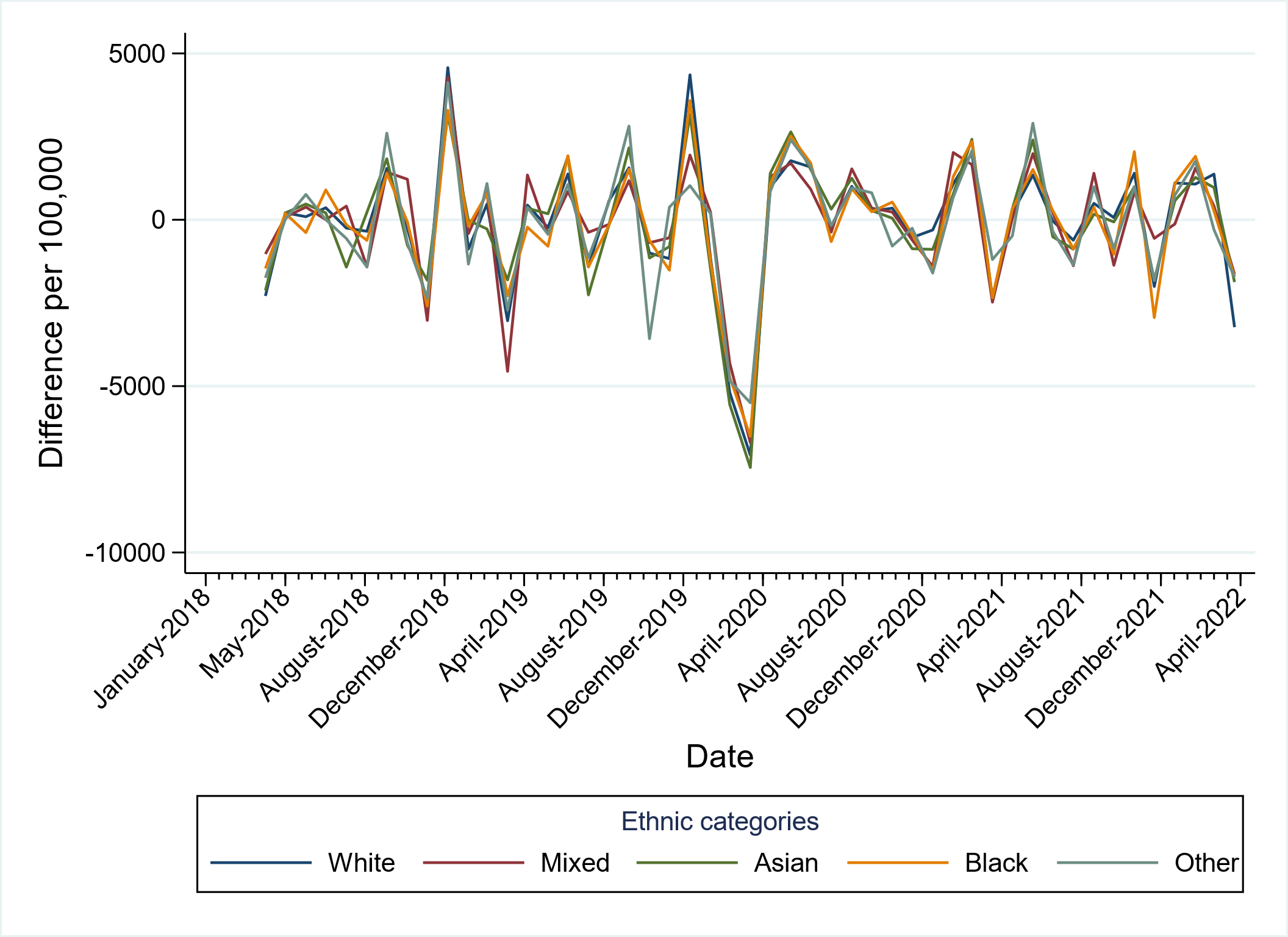

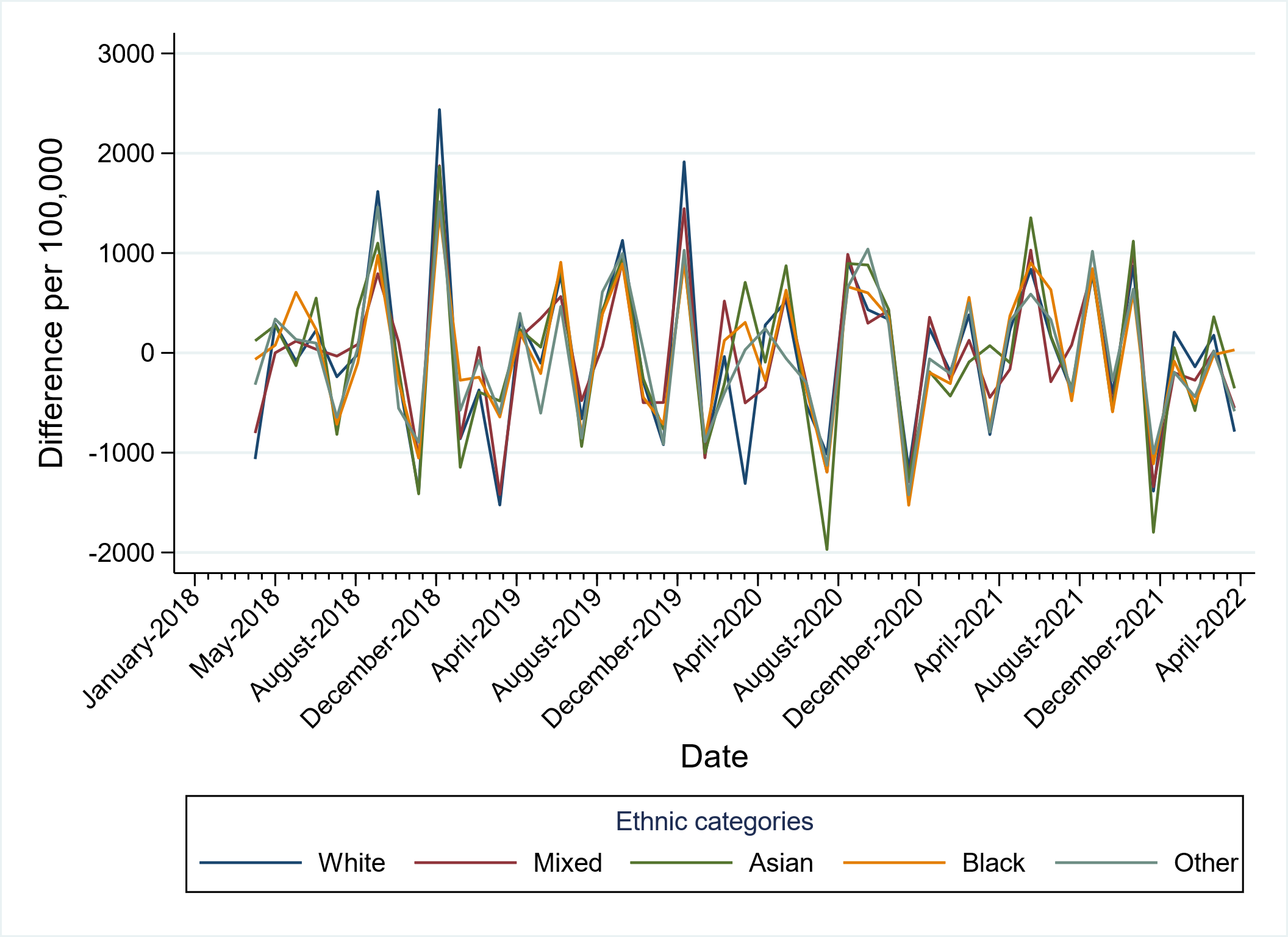


Abbreviations: CVD: Cardiovascular disease, SMI: severe mental illness, COPD: chronic obstructive pulmonary disease.

Note: Difference refers to the difference between the current months rate and the previous months rate.

### Figure 3: a) Age and sex adjusted hazard ratios pre-pandemic and each pandemic time-period for each non-White ethnic group versus White, b) Ratio of hazard ratios for each ethnic group.


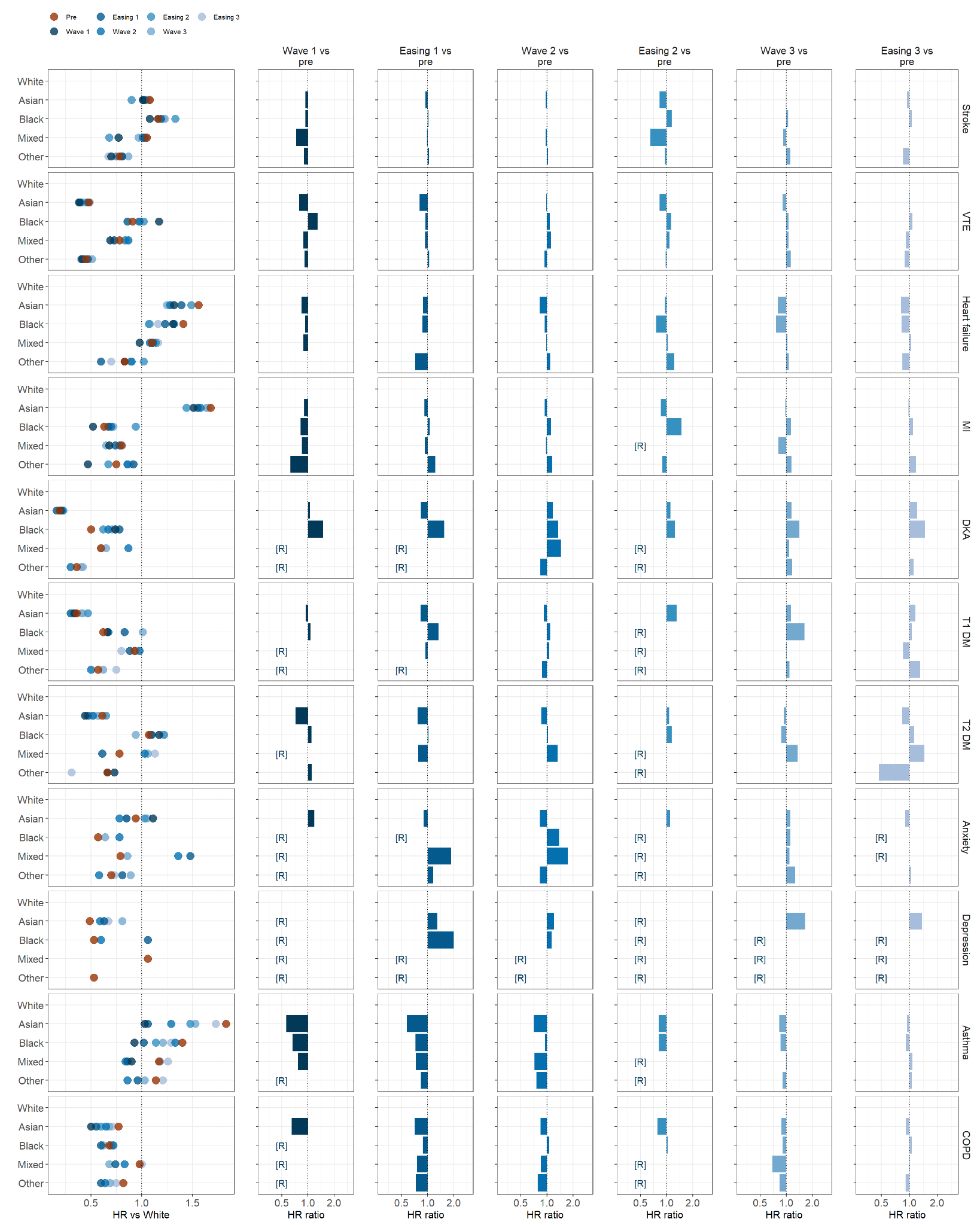


### Figure 4: a) Fully adjusted hazard ratios pre-pandemic and pandemic for each non-White ethnic group versus White, b) Ratio of hazard ratios for each ethnic group.


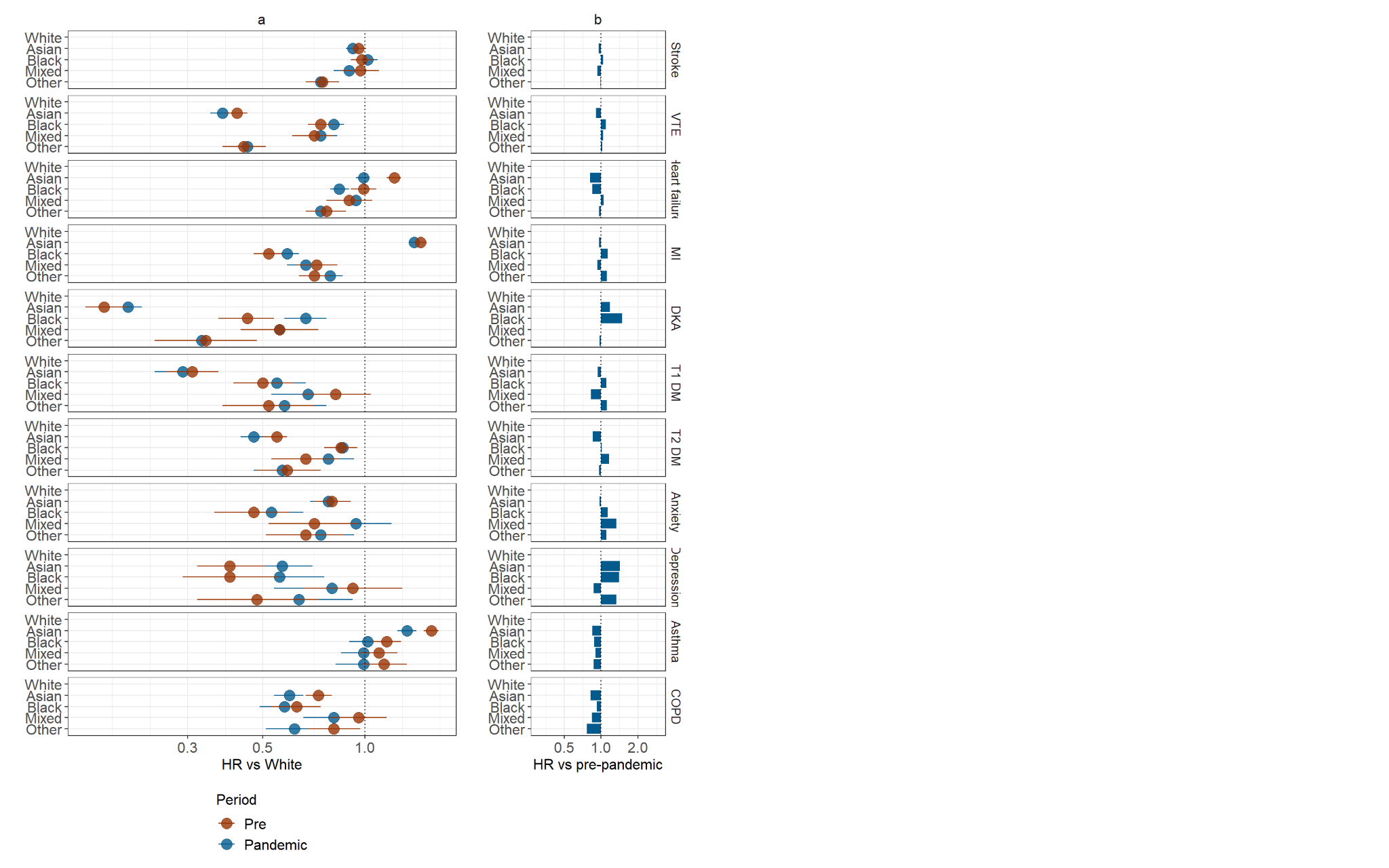


### Figure 5: a) Fully adjusted hazard ratios pre-pandemic and each pandemic time-period for each non-White ethnic group versus White, b) Ratio of hazard ratios for each ethnic group.


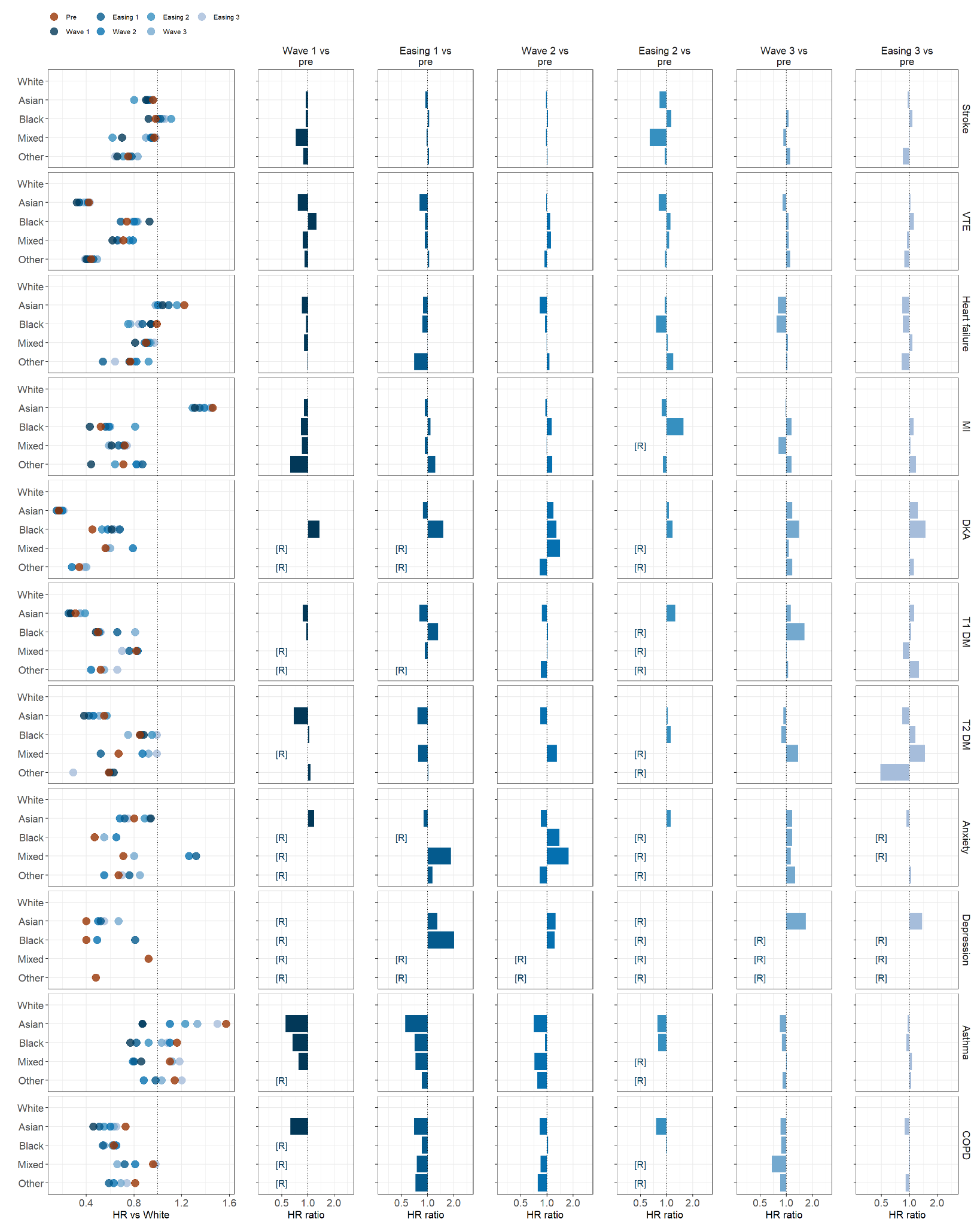


### Figure 6: a) Fully adjusted hazard ratios pre-pandemic and pandemic for each ethnic group versus Asian, b) Ratio of hazard ratios for each ethnic group


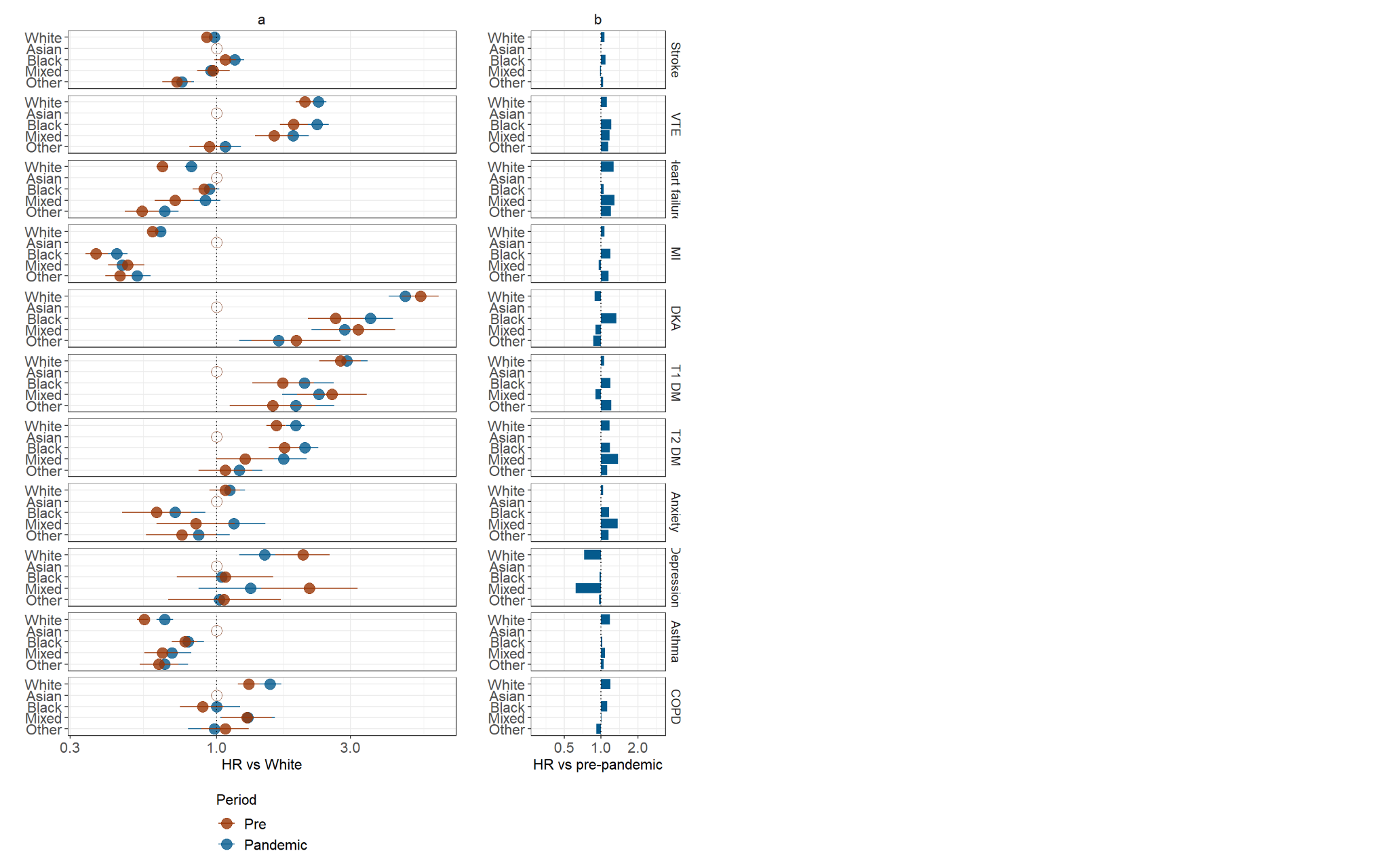


### Figure 7: a) Fully adjusted hazard ratios pre-pandemic and pandemic for each ethnic group versus Black, b) Ratio of hazard ratios for each ethnic group


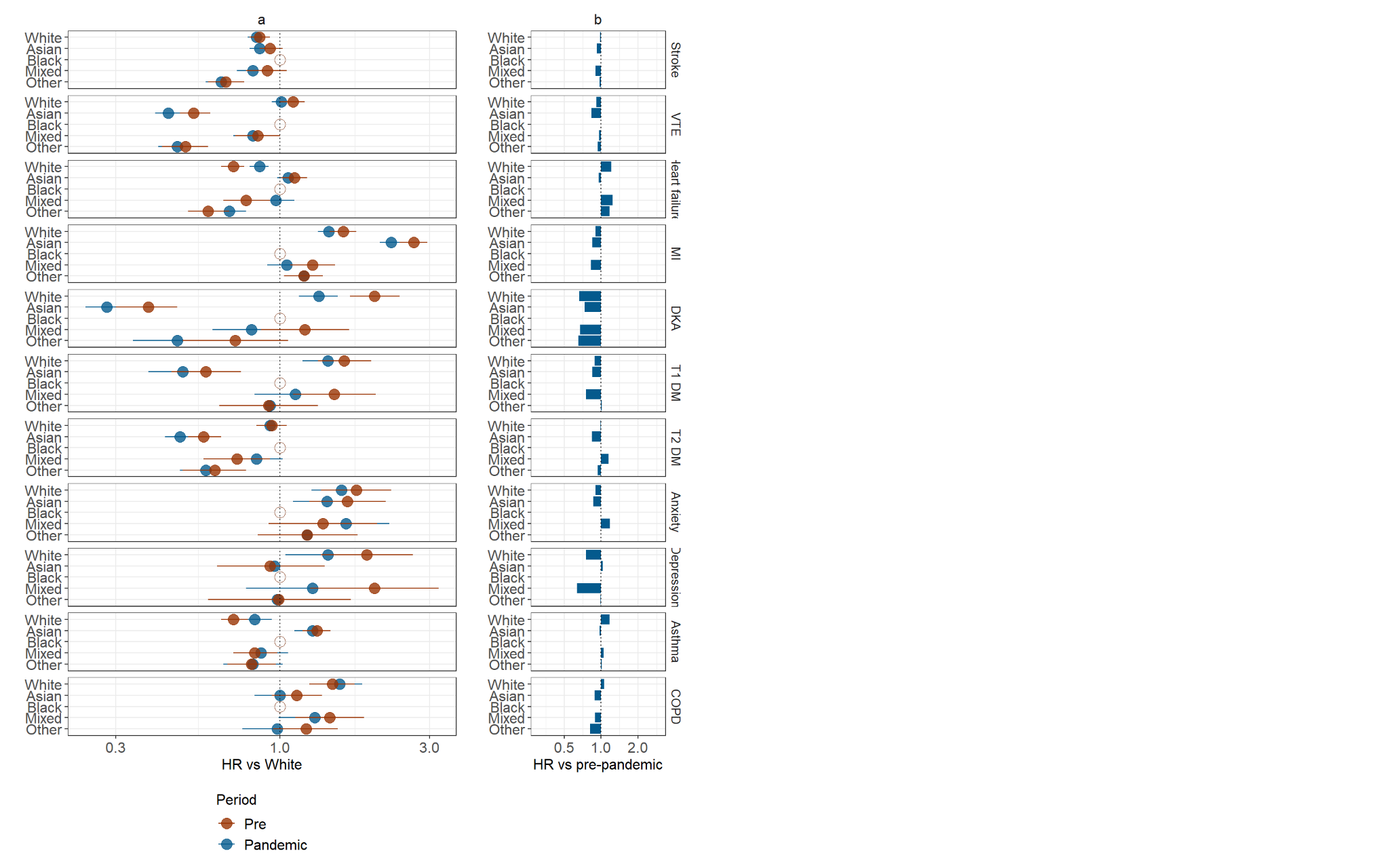


### Figure 8: a) Fully adjusted hazard ratios pre-pandemic and pandemic for each ethnic group versus Mixed, b) Ratio of hazard ratios for each ethnic group


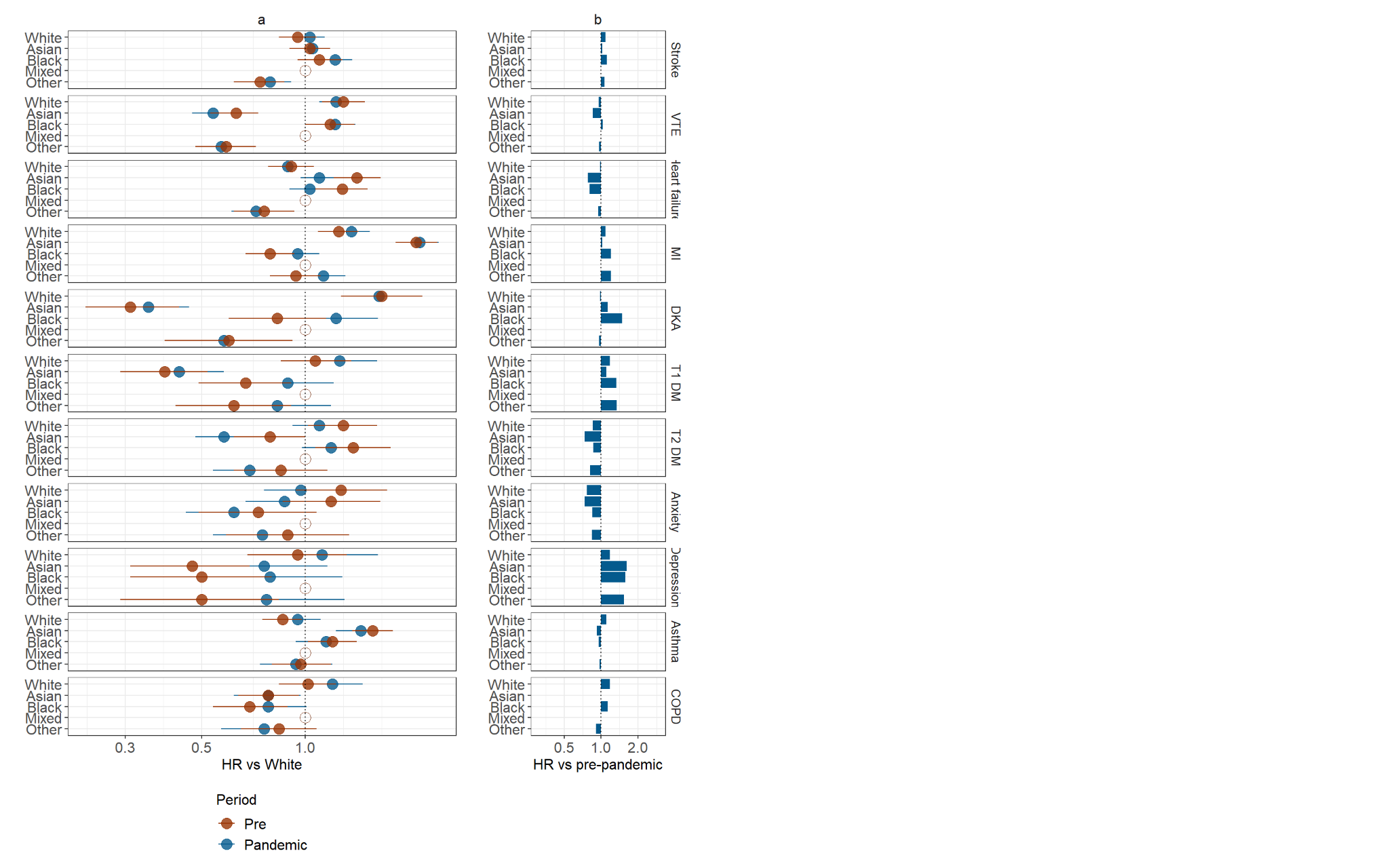


### Figure 9: a) Fully adjusted hazard ratios pre-pandemic and pandemic for each ethnic group versus Other ethnic group, b) Ratio of hazard ratios for each ethnic group


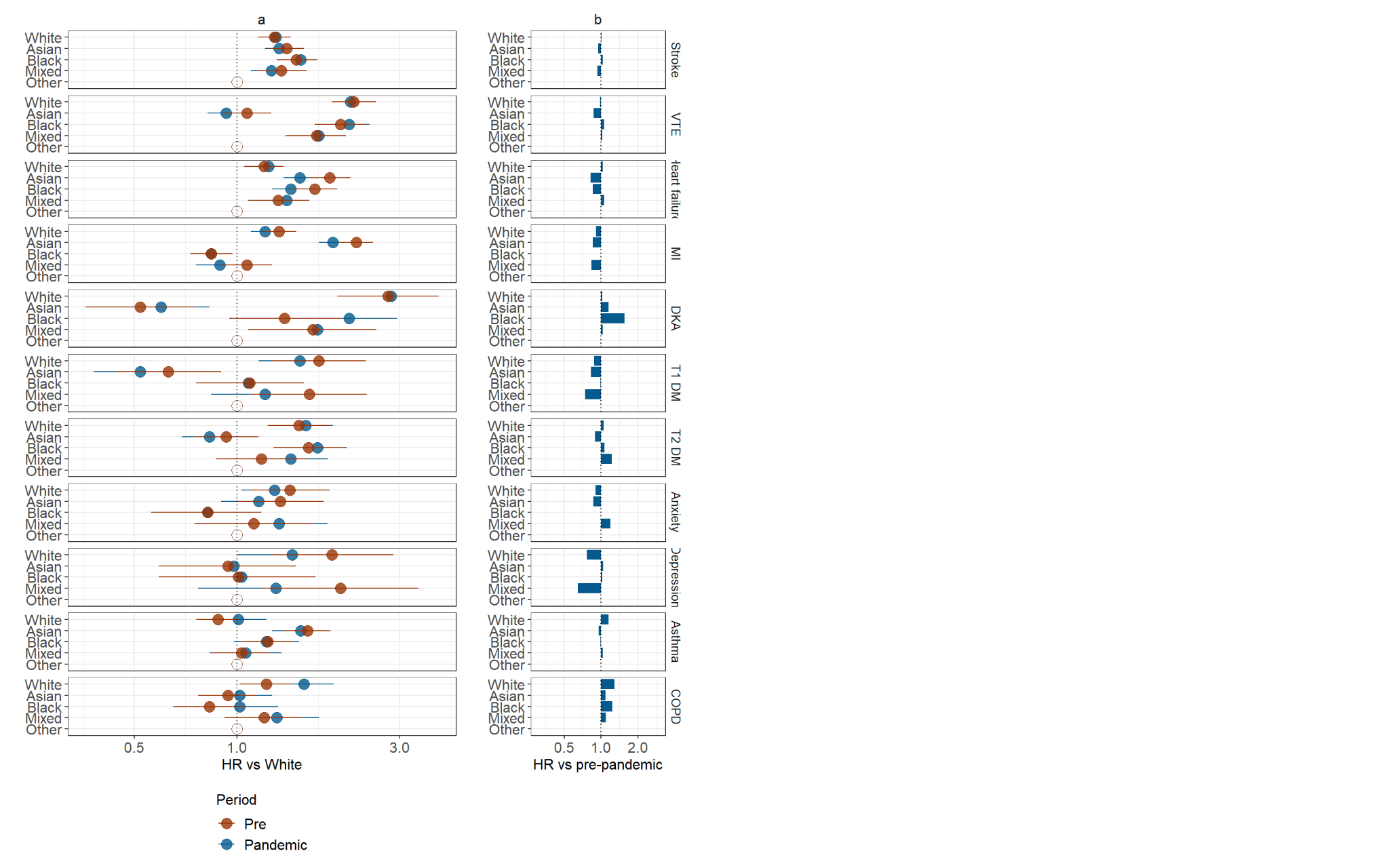


### Table 4: Number of events, rate, hazard ratio and confidence intervals by ethnic group, time period and outcome with those of Asian ethnicity as the reference group.

| **Outcome** | **Period** | **Ethnicity** | **Denominator** | **Events** | **Total person weeks** | **Rate** | **Unadjusted** | | **Age and sex adjusted** | | **Fully adjusted** | |
| --- | --- | --- | --- | --- | --- | --- | --- | --- | --- | --- | --- | --- |
|  |  |  |  |  |  |  | **Hazard ratio** | **95% confidence interval** | **Hazard ratio** | **95% confidence interval** | **Hazard ratio** | **95% confidence interval** |
| Stroke | Pre | White | 11951861 | 42033 | 299064047 | 14.05 | 1.61 | (1.54, 1.69) | 0.92 | (0.88, 0.97) | 1.04 | (0.99, 1.09) |
| Stroke | Pre | Asian | 947198 | 1835 | 23717609 | 7.74 | Reference |  | Reference |  | Reference |  |
| Stroke | Pre | Black | 323687 | 683 | 8104085 | 8.43 | 1.1 | (1, 1.2) | 1.07 | (0.98, 1.17) | 1.02 | (0.93, 1.11) |
| Stroke | Pre | Mixed | 167862 | 249 | 4204083 | 5.92 | 0.74 | (0.65, 0.85) | 0.97 | (0.85, 1.11) | 1 | (0.88, 1.14) |
| Stroke | Pre | Other | 255875 | 311 | 6408427 | 4.85 | 0.62 | (0.55, 0.7) | 0.72 | (0.64, 0.81) | 0.78 | (0.69, 0.88) |
| Stroke | Pandemic | White | 13007778 | 62025 | 321371341 | 19.3 | 1.77 | (1.7, 1.84) | 0.98 | (0.94, 1.02) | 1.09 | (1.04, 1.13) |
| Stroke | Pandemic | Asian | 1116583 | 2669 | 27539949 | 9.69 | Reference |  | Reference |  | Reference |  |
| Stroke | Pandemic | Black | 389039 | 1107 | 9530751 | 11.62 | 1.21 | (1.12, 1.29) | 1.16 | (1.08, 1.25) | 1.11 | (1.03, 1.19) |
| Stroke | Pandemic | Mixed | 206060 | 357 | 5033434 | 7.09 | 0.71 | (0.64, 0.79) | 0.95 | (0.85, 1.06) | 0.98 | (0.88, 1.09) |
| Stroke | Pandemic | Other | 334274 | 485 | 8011353 | 6.05 | 0.63 | (0.57, 0.69) | 0.75 | (0.68, 0.83) | 0.8 | (0.73, 0.89) |
| Stroke | Wave 1 | White | 13008002 | 4886 | 29408734 | 16.61 | 1.79 | (1.55, 2.06) | 0.99 | (0.85, 1.14) | 1.11 | (0.96, 1.29) |
| Stroke | Wave 1 | Asian | 1116586 | 210 | 2525075 | 8.32 | Reference |  | Reference |  | Reference |  |
| Stroke | Wave 1 | Black | 389021 | 79 | 879114 | 8.99 | 1.11 | (0.86, 1.44) | 1.07 | (0.82, 1.38) | 1.02 | (0.78, 1.32) |
| Stroke | Wave 1 | Mixed | 206091 | 22 | 465920 | 4.72 | 0.55 | (0.36, 0.86) | 0.76 | (0.49, 1.18) | 0.78 | (0.5, 1.21) |
| Stroke | Wave 1 | Other | 334305 | 35 | 754927 | 4.64 | 0.55 | (0.39, 0.79) | 0.69 | (0.48, 0.98) | 0.74 | (0.51, 1.05) |
| Stroke | Easing 1 | White | 13059172 | 8104 | 42509205 | 19.06 | 1.8 | (1.61, 2.02) | 0.98 | (0.88, 1.1) | 1.1 | (0.98, 1.23) |
| Stroke | Easing 1 | Asian | 1128784 | 343 | 3674505 | 9.33 | Reference |  | Reference |  | Reference |  |
| Stroke | Easing 1 | Black | 393360 | 143 | 1279287 | 11.18 | 1.21 | (1, 1.47) | 1.17 | (0.96, 1.42) | 1.12 | (0.92, 1.36) |
| Stroke | Easing 1 | Mixed | 208793 | 49 | 678885 | 7.22 | 0.75 | (0.55, 1.01) | 1.01 | (0.75, 1.37) | 1.05 | (0.77, 1.41) |
| Stroke | Easing 1 | Other | 338288 | 66 | 1098220 | 6.01 | 0.65 | (0.5, 0.84) | 0.8 | (0.61, 1.04) | 0.85 | (0.65, 1.11) |
| Stroke | Wave 2 | White | 12967133 | 19186 | 97788237 | 19.62 | 1.77 | (1.64, 1.9) | 0.97 | (0.9, 1.04) | 1.08 | (1, 1.16) |
| Stroke | Wave 2 | Asian | 1122606 | 839 | 8458675 | 9.92 | Reference |  | Reference |  | Reference |  |
| Stroke | Wave 2 | Black | 390035 | 338 | 2934168 | 11.52 | 1.17 | (1.03, 1.33) | 1.12 | (0.99, 1.27) | 1.07 | (0.94, 1.22) |
| Stroke | Wave 2 | Mixed | 207204 | 115 | 1555135 | 7.39 | 0.72 | (0.6, 0.88) | 0.98 | (0.8, 1.19) | 1.01 | (0.83, 1.23) |
| Stroke | Wave 2 | Other | 332901 | 156 | 2485211 | 6.28 | 0.64 | (0.54, 0.75) | 0.77 | (0.65, 0.91) | 0.82 | (0.69, 0.98) |
| Stroke | Easing 2 | White | 12640411 | 2984 | 13461555 | 22.17 | 1.99 | (1.64, 2.41) | 1.11 | (0.91, 1.35) | 1.25 | (1.03, 1.52) |
| Stroke | Easing 2 | Asian | 1095134 | 114 | 1166215 | 9.78 | Reference |  | Reference |  | Reference |  |
| Stroke | Easing 2 | Black | 379052 | 59 | 403523 | 14.62 | 1.52 | (1.11, 2.09) | 1.47 | (1.08, 2.02) | 1.39 | (1.02, 1.91) |
| Stroke | Easing 2 | Mixed | 201010 | 12 | 213928 | 5.61 | 0.56 | (0.31, 1.01) | 0.75 | (0.41, 1.36) | 0.77 | (0.43, 1.4) |
| Stroke | Easing 2 | Other | 317013 | 23 | 337061 | 6.82 | 0.69 | (0.44, 1.08) | 0.83 | (0.53, 1.3) | 0.89 | (0.57, 1.39) |
| Stroke | Wave 3 | White | 12602238 | 16633 | 83144516 | 20 | 1.69 | (1.56, 1.82) | 0.93 | (0.86, 1.01) | 1.04 | (0.97, 1.13) |
| Stroke | Wave 3 | Asian | 1091687 | 756 | 7193014 | 10.51 | Reference |  | Reference |  | Reference |  |
| Stroke | Wave 3 | Black | 377684 | 312 | 2482157 | 12.57 | 1.19 | (1.05, 1.36) | 1.14 | (1, 1.3) | 1.09 | (0.95, 1.24) |
| Stroke | Wave 3 | Mixed | 200227 | 98 | 1314892 | 7.45 | 0.68 | (0.55, 0.85) | 0.91 | (0.74, 1.12) | 0.94 | (0.76, 1.16) |
| Stroke | Wave 3 | Other | 314766 | 148 | 2054547 | 7.2 | 0.68 | (0.57, 0.81) | 0.81 | (0.68, 0.97) | 0.87 | (0.73, 1.03) |
| Stroke | Easing 3 | White | 12411670 | 11376 | 55895206 | 20.35 | 1.8 | (1.64, 1.97) | 0.98 | (0.89, 1.08) | 1.08 | (0.98, 1.19) |
| Stroke | Easing 3 | Asian | 1074695 | 487 | 4837290 | 10.07 | Reference |  | Reference |  | Reference |  |
| Stroke | Easing 3 | Black | 369667 | 210 | 1661122 | 12.64 | 1.26 | (1.07, 1.48) | 1.2 | (1.02, 1.41) | 1.15 | (0.98, 1.35) |
| Stroke | Easing 3 | Mixed | 197112 | 71 | 885370 | 8.020001 | 0.77 | (0.6, 0.99) | 1.03 | (0.8, 1.32) | 1.06 | (0.82, 1.36) |
| Stroke | Easing 3 | Other | 304150 | 76 | 1360142 | 5.59 | 0.55 | (0.44, 0.71) | 0.65 | (0.51, 0.83) | 0.69 | (0.54, 0.88) |
| VTE | Pre | White | 11951881 | 28859 | 299215565 | 9.64 | 2.75 | (2.55, 2.96) | 2.06 | (1.91, 2.22) | 2.38 | (1.91, 2.22) |
| VTE | Pre | Asian | 947196 | 752 | 23730820 | 3.17 | Reference |  | Reference |  | Reference |  |
| VTE | Pre | Black | 323685 | 501 | 8106277 | 6.18 | 1.93 | (1.72, 2.16) | 1.88 | (1.68, 2.11) | 1.77 | (1.68, 2.11) |
| VTE | Pre | Mixed | 167862 | 195 | 4204866 | 4.64 | 1.41 | (1.2, 1.65) | 1.6 | (1.37, 1.87) | 1.68 | (1.37, 1.87) |
| VTE | Pre | Other | 255875 | 181 | 6409861 | 2.82 | 0.88 | (0.75, 1.04) | 0.94 | (0.8, 1.1) | 1.05 | (0.8, 1.1) |
| VTE | Pandemic | White | 13007823 | 38710 | 321532860 | 12.04 | 3.13 | (2.93, 3.34) | 2.3 | (2.15, 2.46) | 2.65 | (2.15, 2.46) |
| VTE | Pandemic | Asian | 1116586 | 939 | 27555834 | 3.41 | Reference |  | Reference |  | Reference |  |
| VTE | Pandemic | Black | 389040 | 785 | 9532588 | 8.23 | 2.34 | (2.13, 2.57) | 2.28 | (2.08, 2.51) | 2.15 | (2.08, 2.51) |
| VTE | Pandemic | Mixed | 206060 | 290 | 5033737 | 5.76 | 1.6 | (1.4, 1.82) | 1.87 | (1.64, 2.13) | 1.96 | (1.64, 2.13) |
| VTE | Pandemic | Other | 334273 | 275 | 8013201 | 3.43 | 0.97 | (0.85, 1.11) | 1.07 | (0.94, 1.22) | 1.19 | (0.94, 1.22) |
| VTE | Wave 1 | White | 13008047 | 2634 | 29410861 | 8.96 | 3.56 | (2.74, 4.62) | 2.63 | (2.02, 3.43) | 3.09 | (2.02, 3.43) |
| VTE | Wave 1 | Asian | 1116589 | 59 | 2525212 | 2.34 | Reference |  | Reference |  | Reference |  |
| VTE | Wave 1 | Black | 389022 | 65 | 879116 | 7.39 | 3.16 | (2.22, 4.5) | 3.08 | (2.17, 4.39) | 2.87 | (2.17, 4.39) |
| VTE | Wave 1 | Mixed | 206091 | 17 | 465927 | 3.65 | 1.53 | (0.89, 2.62) | 1.81 | (1.06, 3.11) | 1.92 | (1.06, 3.11) |
| VTE | Wave 1 | Other | 334304 | 17 | 754936 | 2.25 | 0.96 | (0.56, 1.66) | 1.08 | (0.63, 1.86) | 1.23 | (0.63, 1.86) |
| VTE | Easing 1 | White | 13059222 | 5157 | 42512892 | 12.13 | 3.42 | (2.83, 4.12) | 2.54 | (2.1, 3.07) | 2.98 | (2.1, 3.07) |
| VTE | Easing 1 | Asian | 1128788 | 116 | 3674863 | 3.16 | Reference |  | Reference |  | Reference |  |
| VTE | Easing 1 | Black | 393361 | 93 | 1279337 | 7.27 | 2.25 | (1.71, 2.96) | 2.19 | (1.66, 2.87) | 2.04 | (1.66, 2.87) |
| VTE | Easing 1 | Mixed | 208793 | 36 | 678890 | 5.3 | 1.59 | (1.09, 2.31) | 1.87 | (1.28, 2.71) | 1.97 | (1.28, 2.71) |
| VTE | Easing 1 | Other | 338288 | 39 | 1098249 | 3.55 | 1.09 | (0.75, 1.56) | 1.2 | (0.84, 1.73) | 1.36 | (0.84, 1.73) |
| VTE | Wave 2 | White | 12967150 | 12627 | 97803435 | 12.91 | 2.94 | (2.63, 3.29) | 2.14 | (1.91, 2.4) | 2.47 | (1.91, 2.4) |
| VTE | Wave 2 | Asian | 1122608 | 324 | 8460268 | 3.83 | Reference |  | Reference |  | Reference |  |
| VTE | Wave 2 | Black | 390036 | 253 | 2934299 | 8.62 | 2.17 | (1.84, 2.55) | 2.11 | (1.79, 2.48) | 1.98 | (1.79, 2.48) |
| VTE | Wave 2 | Mixed | 207205 | 101 | 1555115 | 6.49 | 1.59 | (1.27, 1.99) | 1.87 | (1.49, 2.34) | 1.96 | (1.49, 2.34) |
| VTE | Wave 2 | Other | 332902 | 80 | 2485458 | 3.22 | 0.81 | (0.64, 1.04) | 0.9 | (0.7, 1.15) | 1 | (0.7, 1.15) |
| VTE | Easing 2 | White | 12640457 | 1801 | 13462095 | 13.38 | 3.46 | (2.53, 4.74) | 2.51 | (1.83, 3.45) | 2.94 | (1.83, 3.45) |
| VTE | Easing 2 | Asian | 1095139 | 41 | 1166258 | 3.52 | Reference |  | Reference |  | Reference |  |
| VTE | Easing 2 | Black | 379052 | 38 | 403537 | 9.42 | 2.64 | (1.69, 4.1) | 2.57 | (1.65, 4) | 2.4 | (1.65, 4) |
| VTE | Easing 2 | Mixed | 201011 | 14 | 213929 | 6.54 | 1.8 | (0.98, 3.3) | 2.12 | (1.15, 3.89) | 2.24 | (1.15, 3.89) |
| VTE | Easing 2 | Other | 317012 | 12 | 337066 | 3.56 | 0.99 | (0.52, 1.89) | 1.09 | (0.57, 2.08) | 1.24 | (0.57, 2.08) |
| VTE | Wave 3 | White | 12602274 | 10091 | 83160465 | 12.13 | 3.07 | (2.71, 3.49) | 2.29 | (2.01, 2.6) | 2.65 | (2.01, 2.6) |
| VTE | Wave 3 | Asian | 1091690 | 249 | 7194558 | 3.46 | Reference |  | Reference |  | Reference |  |
| VTE | Wave 3 | Black | 377684 | 202 | 2482470 | 8.14 | 2.26 | (1.88, 2.72) | 2.21 | (1.83, 2.66) | 2.08 | (1.83, 2.66) |
| VTE | Wave 3 | Mixed | 200227 | 80 | 1314939 | 6.08 | 1.65 | (1.28, 2.12) | 1.91 | (1.48, 2.46) | 2.01 | (1.48, 2.46) |
| VTE | Wave 3 | Other | 314767 | 80 | 2054751 | 3.89 | 1.07 | (0.83, 1.37) | 1.16 | (0.9, 1.5) | 1.3 | (0.9, 1.5) |
| VTE | Easing 3 | White | 12411687 | 6834 | 55902234 | 12.22 | 2.81 | (2.42, 3.27) | 2.06 | (1.77, 2.4) | 2.32 | (1.77, 2.4) |
| VTE | Easing 3 | Asian | 1074698 | 184 | 4837902 | 3.8 | Reference |  | Reference |  | Reference |  |
| VTE | Easing 3 | Black | 369670 | 136 | 1661279 | 8.19 | 2.07 | (1.65, 2.58) | 2.01 | (1.61, 2.51) | 1.92 | (1.61, 2.51) |
| VTE | Easing 3 | Mixed | 197112 | 46 | 885409 | 5.2 | 1.28 | (0.93, 1.77) | 1.49 | (1.08, 2.06) | 1.55 | (1.08, 2.06) |
| VTE | Easing 3 | Other | 304148 | 41 | 1360203 | 3.01 | 0.76 | (0.54, 1.06) | 0.83 | (0.59, 1.16) | 0.9 | (0.59, 1.16) |
| Heart failure | Pre | White | 11951886 | 31037 | 299209045 | 10.37 | 1.29 | (1.23, 1.35) | 0.64 | (0.61, 0.67) | 0.82 | (0.78, 0.86) |
| Heart failure | Pre | Asian | 947193 | 1925 | 23716286 | 8.12 | Reference |  | Reference |  | Reference |  |
| Heart failure | Pre | Black | 323687 | 597 | 8104779 | 7.37 | 0.92 | (0.84, 1.01) | 0.9 | (0.82, 0.99) | 0.81 | (0.74, 0.89) |
| Heart failure | Pre | Mixed | 167861 | 166 | 4204968 | 3.95 | 0.5 | (0.42, 0.58) | 0.71 | (0.6, 0.83) | 0.74 | (0.63, 0.87) |
| Heart failure | Pre | Other | 255875 | 222 | 6409411 | 3.46 | 0.44 | (0.39, 0.51) | 0.54 | (0.47, 0.62) | 0.63 | (0.55, 0.72) |
| Heart failure | Pandemic | White | 13007814 | 50729 | 321522961 | 15.78 | 1.68 | (1.62, 1.75) | 0.81 | (0.77, 0.84) | 1.01 | (0.97, 1.06) |
| Heart failure | Pandemic | Asian | 1116584 | 2508 | 27542692 | 9.11 | Reference |  | Reference |  | Reference |  |
| Heart failure | Pandemic | Black | 389038 | 842 | 9532933 | 8.83 | 0.98 | (0.9, 1.06) | 0.94 | (0.87, 1.02) | 0.85 | (0.79, 0.92) |
| Heart failure | Pandemic | Mixed | 206060 | 284 | 5034330 | 5.64 | 0.62 | (0.55, 0.7) | 0.91 | (0.81, 1.03) | 0.95 | (0.84, 1.08) |
| Heart failure | Pandemic | Other | 334275 | 362 | 8013004 | 4.52 | 0.51 | (0.46, 0.57) | 0.65 | (0.58, 0.73) | 0.75 | (0.67, 0.84) |
| Heart failure | Wave 1 | White | 13008038 | 3540 | 29410481 | 12.04 | 1.58 | (1.36, 1.84) | 0.76 | (0.65, 0.89) | 0.96 | (0.82, 1.12) |
| Heart failure | Wave 1 | Asian | 1116587 | 185 | 2525095 | 7.33 | Reference |  | Reference |  | Reference |  |
| Heart failure | Wave 1 | Black | 389020 | 65 | 879134 | 7.39 | 1.03 | (0.78, 1.37) | 0.99 | (0.75, 1.31) | 0.9 | (0.68, 1.2) |
| Heart failure | Wave 1 | Mixed | 206091 | 17 | 465929 | 3.65 | 0.5 | (0.3, 0.82) | 0.74 | (0.45, 1.22) | 0.78 | (0.47, 1.28) |
| Heart failure | Wave 1 | Other | 334306 | 27 | 754939 | 3.58 | 0.48 | (0.32, 0.73) | 0.63 | (0.42, 0.95) | 0.73 | (0.48, 1.1) |
| Heart failure | Easing 1 | White | 13059194 | 6970 | 42510238 | 16.4 | 1.51 | (1.36, 1.68) | 0.72 | (0.64, 0.8) | 0.92 | (0.83, 1.03) |
| Heart failure | Easing 1 | Asian | 1128784 | 376 | 3674476 | 10.23 | Reference |  | Reference |  | Reference |  |
| Heart failure | Easing 1 | Black | 393360 | 121 | 1279279 | 9.46 | 0.92 | (0.75, 1.13) | 0.88 | (0.72, 1.08) | 0.8 | (0.65, 0.98) |
| Heart failure | Easing 1 | Mixed | 208793 | 38 | 678881 | 5.6 | 0.54 | (0.39, 0.75) | 0.8 | (0.57, 1.11) | 0.84 | (0.6, 1.17) |
| Heart failure | Easing 1 | Other | 338286 | 37 | 1098249 | 3.37 | 0.33 | (0.23, 0.47) | 0.43 | (0.3, 0.6) | 0.5 | (0.35, 0.7) |
| Heart failure | Wave 2 | White | 12967138 | 16378 | 97799415 | 16.75 | 1.63 | (1.52, 1.76) | 0.78 | (0.73, 0.84) | 1 | (0.93, 1.07) |
| Heart failure | Wave 2 | Asian | 1122605 | 844 | 8458766 | 9.98 | Reference |  | Reference |  | Reference |  |
| Heart failure | Wave 2 | Black | 390036 | 311 | 2934208 | 10.6 | 1.07 | (0.94, 1.22) | 1.03 | (0.91, 1.18) | 0.94 | (0.83, 1.07) |
| Heart failure | Wave 2 | Mixed | 207204 | 89 | 1555247 | 5.72 | 0.58 | (0.46, 0.72) | 0.85 | (0.68, 1.05) | 0.89 | (0.72, 1.11) |
| Heart failure | Wave 2 | Other | 332902 | 131 | 2485311 | 5.27 | 0.55 | (0.45, 0.66) | 0.71 | (0.59, 0.85) | 0.82 | (0.68, 0.98) |
| Heart failure | Easing 2 | White | 12640422 | 3464 | 13461250 | 25.73 | 1.45 | (1.25, 1.68) | 0.67 | (0.58, 0.78) | 0.87 | (0.74, 1.01) |
| Heart failure | Easing 2 | Asian | 1095135 | 199 | 1166170 | 17.06 | Reference |  | Reference |  | Reference |  |
| Heart failure | Easing 2 | Black | 379052 | 52 | 403527 | 12.89 | 0.76 | (0.56, 1.03) | 0.72 | (0.53, 0.98) | 0.65 | (0.48, 0.88) |
| Heart failure | Easing 2 | Mixed | 201011 | 19 | 213926 | 8.88 | 0.52 | (0.32, 0.83) | 0.77 | (0.48, 1.23) | 0.8 | (0.5, 1.29) |
| Heart failure | Easing 2 | Other | 317012 | 30 | 337056 | 8.9 | 0.54 | (0.36, 0.79) | 0.69 | (0.47, 1.01) | 0.8 | (0.54, 1.17) |
| Heart failure | Wave 3 | White | 12602204 | 14580 | 83149534 | 17.53 | 1.65 | (1.53, 1.78) | 0.8 | (0.74, 0.86) | 1.02 | (0.94, 1.1) |
| Heart failure | Wave 3 | Asian | 1091682 | 767 | 7192954 | 10.66 | Reference |  | Reference |  | Reference |  |
| Heart failure | Wave 3 | Black | 377683 | 239 | 2482401 | 9.63 | 0.91 | (0.79, 1.05) | 0.86 | (0.75, 1) | 0.78 | (0.67, 0.9) |
| Heart failure | Wave 3 | Mixed | 200224 | 87 | 1314956 | 6.62 | 0.63 | (0.5, 0.78) | 0.92 | (0.73, 1.14) | 0.96 | (0.77, 1.19) |
| Heart failure | Wave 3 | Other | 314766 | 119 | 2054623 | 5.79 | 0.56 | (0.46, 0.68) | 0.71 | (0.58, 0.86) | 0.82 | (0.67, 0.99) |
| Heart failure | Easing 3 | White | 12411683 | 11000 | 55896009 | 19.68 | 1.65 | (1.51, 1.8) | 0.78 | (0.72, 0.86) | 0.98 | (0.9, 1.07) |
| Heart failure | Easing 3 | Asian | 1074692 | 571 | 4837135 | 11.8 | Reference |  | Reference |  | Reference |  |
| Heart failure | Easing 3 | Black | 369669 | 187 | 1661225 | 11.26 | 0.96 | (0.81, 1.13) | 0.91 | (0.77, 1.07) | 0.83 | (0.7, 0.97) |
| Heart failure | Easing 3 | Mixed | 197111 | 65 | 885385 | 7.34 | 0.63 | (0.48, 0.81) | 0.91 | (0.7, 1.17) | 0.95 | (0.73, 1.23) |
| Heart failure | Easing 3 | Other | 304148 | 69 | 1360166 | 5.07 | 0.45 | (0.35, 0.57) | 0.55 | (0.43, 0.71) | 0.63 | (0.49, 0.8) |
| MI | Pre | White | 11951868 | 41681 | 299044045 | 13.94 | 0.95 | (0.91, 0.98) | 0.59 | (0.57, 0.62) | 0.68 | (0.66, 0.71) |
| MI | Pre | Asian | 947193 | 3206 | 23700749 | 13.53 | Reference |  | Reference |  | Reference |  |
| MI | Pre | Black | 323687 | 425 | 8107170 | 5.24 | 0.4 | (0.36, 0.44) | 0.37 | (0.34, 0.41) | 0.35 | (0.32, 0.39) |
| MI | Pre | Mixed | 167862 | 212 | 4204384 | 5.04 | 0.37 | (0.32, 0.43) | 0.48 | (0.41, 0.55) | 0.49 | (0.43, 0.57) |
| MI | Pre | Other | 255872 | 329 | 6407889 | 5.13 | 0.39 | (0.35, 0.44) | 0.45 | (0.4, 0.5) | 0.49 | (0.43, 0.55) |
| MI | Pandemic | White | 13007792 | 49713 | 321413154 | 15.47 | 1.01 | (0.98, 1.04) | 0.63 | (0.61, 0.65) | 0.72 | (0.69, 0.74) |
| MI | Pandemic | Asian | 1116586 | 3864 | 27525109 | 14.04 | Reference |  | Reference |  | Reference |  |
| MI | Pandemic | Black | 389038 | 603 | 9534880 | 6.32 | 0.46 | (0.43, 0.51) | 0.44 | (0.41, 0.48) | 0.42 | (0.39, 0.46) |
| MI | Pandemic | Mixed | 206059 | 252 | 5033918 | 5.01 | 0.36 | (0.31, 0.41) | 0.46 | (0.41, 0.53) | 0.48 | (0.42, 0.55) |
| MI | Pandemic | Other | 334275 | 480 | 8011100 | 5.99 | 0.44 | (0.4, 0.49) | 0.52 | (0.48, 0.58) | 0.56 | (0.51, 0.62) |
| MI | Wave 1 | White | 13008016 | 3585 | 29409854 | 12.19 | 1.04 | (0.92, 1.19) | 0.66 | (0.58, 0.76) | 0.76 | (0.67, 0.87) |
| MI | Wave 1 | Asian | 1116589 | 271 | 2525011 | 10.73 | Reference |  | Reference |  | Reference |  |
| MI | Wave 1 | Black | 389020 | 33 | 879155 | 3.75 | 0.36 | (0.25, 0.52) | 0.35 | (0.24, 0.5) | 0.33 | (0.23, 0.47) |
| MI | Wave 1 | Mixed | 206090 | 17 | 465920 | 3.65 | 0.34 | (0.21, 0.56) | 0.45 | (0.27, 0.73) | 0.47 | (0.29, 0.76) |
| MI | Wave 1 | Other | 334306 | 21 | 754936 | 2.78 | 0.26 | (0.16, 0.4) | 0.31 | (0.2, 0.49) | 0.34 | (0.21, 0.53) |
| MI | Easing 1 | White | 13059185 | 6342 | 42511189 | 14.92 | 1.05 | (0.95, 1.16) | 0.64 | (0.58, 0.71) | 0.74 | (0.67, 0.82) |
| MI | Easing 1 | Asian | 1128784 | 459 | 3674351 | 12.49 | Reference |  | Reference |  | Reference |  |
| MI | Easing 1 | Black | 393362 | 71 | 1279392 | 5.55 | 0.46 | (0.35, 0.58) | 0.43 | (0.34, 0.56) | 0.41 | (0.32, 0.53) |
| MI | Easing 1 | Mixed | 208793 | 31 | 678906 | 4.57 | 0.36 | (0.25, 0.52) | 0.47 | (0.33, 0.68) | 0.49 | (0.34, 0.71) |
| MI | Easing 1 | Other | 338288 | 66 | 1098217 | 6.01 | 0.49 | (0.38, 0.63) | 0.59 | (0.46, 0.77) | 0.64 | (0.5, 0.83) |
| MI | Wave 2 | White | 12967152 | 15520 | 97794466 | 15.87 | 1.03 | (0.96, 1.09) | 0.63 | (0.6, 0.68) | 0.72 | (0.67, 0.77) |
| MI | Wave 2 | Asian | 1122602 | 1172 | 8457313 | 13.86 | Reference |  | Reference |  | Reference |  |
| MI | Wave 2 | Black | 390035 | 182 | 2934586 | 6.2 | 0.46 | (0.4, 0.54) | 0.44 | (0.38, 0.52) | 0.42 | (0.36, 0.5) |
| MI | Wave 2 | Mixed | 207205 | 81 | 1555226 | 5.21 | 0.37 | (0.3, 0.47) | 0.49 | (0.39, 0.62) | 0.51 | (0.41, 0.64) |
| MI | Wave 2 | Other | 332901 | 152 | 2485197 | 6.12 | 0.46 | (0.39, 0.54) | 0.55 | (0.46, 0.65) | 0.59 | (0.5, 0.7) |
| MI | Easing 2 | White | 12640414 | 2489 | 13461750 | 18.49 | 1.14 | (0.97, 1.34) | 0.7 | (0.59, 0.82) | 0.77 | (0.66, 0.91) |
| MI | Easing 2 | Asian | 1095133 | 172 | 1166183 | 14.75 | Reference |  | Reference |  | Reference |  |
| MI | Easing 2 | Black | 379051 | 39 | 403537 | 9.66 | 0.68 | (0.48, 0.97) | 0.65 | (0.46, 0.93) | 0.63 | (0.44, 0.89) |
| MI | Easing 2 | Mixed | redacted |  |  |  |  |  |  |  |  |  |
| MI | Easing 2 | Other | 317013 | 19 | 337062 | 5.64 | 0.39 | (0.24, 0.63) | 0.47 | (0.29, 0.75) | 0.5 | (0.31, 0.8) |
| MI | Wave 3 | White | 12602261 | 13201 | 83150575 | 15.88 | 0.98 | (0.92, 1.05) | 0.61 | (0.57, 0.65) | 0.7 | (0.65, 0.74) |
| MI | Wave 3 | Asian | 1091685 | 1085 | 7191830 | 15.09 | Reference |  | Reference |  | Reference |  |
| MI | Wave 3 | Black | 377686 | 169 | 2482621 | 6.81 | 0.46 | (0.39, 0.54) | 0.44 | (0.37, 0.52) | 0.42 | (0.36, 0.49) |
| MI | Wave 3 | Mixed | 200225 | 60 | 1314987 | 4.56 | 0.3 | (0.23, 0.39) | 0.4 | (0.31, 0.51) | 0.41 | (0.32, 0.53) |
| MI | Wave 3 | Other | 314768 | 133 | 2054569 | 6.47 | 0.45 | (0.38, 0.54) | 0.53 | (0.44, 0.63) | 0.57 | (0.47, 0.68) |
| MI | Easing 3 | White | 12411685 | 9252 | 55898155 | 16.55 | 0.97 | (0.9, 1.05) | 0.61 | (0.56, 0.66) | 0.69 | (0.64, 0.75) |
| MI | Easing 3 | Asian | 1074694 | 783 | 4836634 | 16.19 | Reference |  | Reference |  | Reference |  |
| MI | Easing 3 | Black | 369668 | 115 | 1661334 | 6.92 | 0.44 | (0.36, 0.54) | 0.42 | (0.35, 0.51) | 0.4 | (0.33, 0.49) |
| MI | Easing 3 | Mixed | 197112 | 53 | 885392 | 5.99 | 0.38 | (0.29, 0.5) | 0.49 | (0.37, 0.65) | 0.51 | (0.39, 0.68) |
| MI | Easing 3 | Other | 304150 | 98 | 1360084 | 7.21 | 0.47 | (0.38, 0.58) | 0.54 | (0.44, 0.67) | 0.58 | (0.47, 0.72) |
| DKA | Pre | White | 841518 | 4898 | 21022238 | 23.3 | 3.78 | (3.25, 4.4) | 5.32 | (4.57, 6.18) | 5.79 | (4.98, 6.74) |
| DKA | Pre | Asian | 115505 | 188 | 2892286 | 6.5 | Reference |  | Reference |  | Reference |  |
| DKA | Pre | Black | 28706 | 121 | 717714 | 16.86 | 2.52 | (2, 3.17) | 2.65 | (2.11, 3.34) | 2.58 | (2.05, 3.25) |
| DKA | Pre | Mixed | 9109 | 53 | 227450 | 23.3 | 3.63 | (2.67, 4.92) | 3.19 | (2.35, 4.33) | 3.24 | (2.38, 4.39) |
| DKA | Pre | Other | 12222 | 34 | 305866 | 11.12 | 1.7 | (1.18, 2.45) | 1.92 | (1.33, 2.76) | 1.97 | (1.36, 2.84) |
| DKA | Pandemic | White | 995361 | 5666 | 24030509 | 23.58 | 3.49 | (3.06, 3.99) | 4.7 | (4.11, 5.37) | 5.12 | (4.48, 5.85) |
| DKA | Pandemic | Asian | 142353 | 244 | 3500862 | 6.97 | Reference |  | Reference |  | Reference |  |
| DKA | Pandemic | Black | 36530 | 214 | 887198 | 24.12 | 3.38 | (2.81, 4.06) | 3.53 | (2.94, 4.25) | 3.41 | (2.84, 4.1) |
| DKA | Pandemic | Mixed | 11738 | 65 | 286457 | 22.69 | 3.24 | (2.47, 4.27) | 2.86 | (2.18, 3.76) | 2.89 | (2.2, 3.8) |
| DKA | Pandemic | Other | 16225 | 43 | 394708 | 10.89 | 1.53 | (1.11, 2.12) | 1.66 | (1.2, 2.3) | 1.7 | (1.23, 2.36) |
| DKA | Wave 1 | White | 995392 | 588 | 2242264 | 26.22 | 3.64 | (2.36, 5.6) | 5.08 | (3.31, 7.82) | 5.8 | (3.77, 8.92) |
| DKA | Wave 1 | Asian | 142342 | 23 | 321261 | 7.16 | Reference |  | Reference |  | Reference |  |
| DKA | Wave 1 | Black | 36526 | 21 | 82234 | 25.54 | 3.57 | (1.97, 6.47) | 3.75 | (2.07, 6.79) | 3.55 | (1.96, 6.43) |
| DKA | Wave 1 | Mixed | redacted |  |  |  |  |  |  |  |  |  |
| DKA | Wave 1 | Other | redacted |  |  |  |  |  |  |  |  |  |
| DKA | Easing 1 | White | 992829 | 891 | 3223673 | 27.64 | 4.41 | (3.04, 6.39) | 6.08 | (4.21, 8.79) | 6.74 | (4.66, 9.75) |
| DKA | Easing 1 | Asian | 142905 | 31 | 465180 | 6.66 | Reference |  | Reference |  | Reference |  |
| DKA | Easing 1 | Black | 36652 | 37 | 119132 | 31.06 | 4.47 | (2.77, 7.21) | 4.75 | (2.94, 7.66) | 4.56 | (2.82, 7.36) |
| DKA | Easing 1 | Mixed | redacted |  |  |  |  |  |  |  |  |  |
| DKA | Easing 1 | Other | redacted |  |  |  |  |  |  |  |  |  |
| DKA | Wave 2 | White | 993240 | 2005 | 7442036 | 26.94 | 3.28 | (2.63, 4.09) | 4.45 | (3.57, 5.55) | 4.94 | (3.96, 6.16) |
| DKA | Wave 2 | Asian | 143331 | 89 | 1079487 | 8.24 | Reference |  | Reference |  | Reference |  |
| DKA | Wave 2 | Black | 36803 | 65 | 276312 | 23.52 | 2.86 | (2.07, 3.94) | 2.96 | (2.15, 4.08) | 2.84 | (2.06, 3.92) |
| DKA | Wave 2 | Mixed | 11829 | 33 | 88847 | 37.14 | 4.5 | (3.01, 6.72) | 3.85 | (2.58, 5.75) | 3.91 | (2.62, 5.84) |
| DKA | Wave 2 | Other | 16467 | 13 | 123610 | 10.52 | 1.26 | (0.7, 2.26) | 1.35 | (0.75, 2.41) | 1.39 | (0.78, 2.5) |
| DKA | Easing 2 | White | 997807 | 363 | 1062042 | 34.18 | 3.67 | (2.21, 6.09) | 4.84 | (2.92, 8) | 5.44 | (3.28, 9) |
| DKA | Easing 2 | Asian | 144411 | 17 | 153794 | 11.05 | Reference |  | Reference |  | Reference |  |
| DKA | Easing 2 | Black | 37160 | 13 | 39575 | 32.85 | 2.88 | (1.4, 5.95) | 3 | (1.45, 6.19) | 2.9 | (1.4, 5.98) |
| DKA | Easing 2 | Mixed | redacted |  |  |  |  |  |  |  |  |  |
| DKA | Easing 2 | Other | redacted |  |  |  |  |  |  |  |  |  |
| DKA | Wave 3 | White | 999832 | 1974 | 6568038 | 30.05 | 3.4 | (2.71, 4.27) | 4.61 | (3.68, 5.78) | 5.04 | (4.02, 6.33) |
| DKA | Wave 3 | Asian | 144709 | 84 | 955023 | 8.8 | Reference |  | Reference |  | Reference |  |
| DKA | Wave 3 | Black | 37309 | 70 | 245487 | 28.51 | 3.12 | (2.27, 4.28) | 3.27 | (2.38, 4.5) | 3.17 | (2.3, 4.36) |
| DKA | Wave 3 | Mixed | 12024 | 25 | 79183 | 31.57 | 3.52 | (2.25, 5.51) | 3 | (1.92, 4.69) | 3.05 | (1.95, 4.77) |
| DKA | Wave 3 | Other | 16735 | 18 | 110066 | 16.35 | 1.83 | (1.1, 3.05) | 1.96 | (1.17, 3.26) | 2.02 | (1.21, 3.36) |
| DKA | Easing 3 | White | 1004850 | 1402 | 4506363 | 31.11 | 3.34 | (2.57, 4.33) | 4.42 | (3.41, 5.73) | 4.74 | (3.65, 6.14) |
| DKA | Easing 3 | Asian | 146317 | 65 | 658544 | 9.87 | Reference |  | Reference |  | Reference |  |
| DKA | Easing 3 | Black | 37795 | 55 | 169620 | 32.43 | 3.17 | (2.21, 4.54) | 3.29 | (2.29, 4.71) | 3.18 | (2.22, 4.57) |
| DKA | Easing 3 | Mixed | 12233 | 17 | 54888 | 30.97 | 3.12 | (1.83, 5.33) | 2.66 | (1.56, 4.55) | 2.7 | (1.58, 4.6) |
| DKA | Easing 3 | Other | 16941 | 13 | 76042 | 17.1 | 1.68 | (0.93, 3.06) | 1.77 | (0.98, 3.22) | 1.81 | (1, 3.29) |
| T1 DM | Pre | White | 87911 | 5492 | 2126366 | 258.28 | 3.02 | (2.54, 3.58) | 2.76 | (2.32, 3.27) | 3.2 | (2.69, 3.8) |
| T1 DM | Pre | Asian | 5914 | 141 | 146226 | 96.43 | Reference |  | Reference |  | Reference |  |
| T1 DM | Pre | Black | 2450 | 105 | 59963 | 175.11 | 1.79 | (1.39, 2.3) | 1.72 | (1.34, 2.22) | 1.62 | (1.25, 2.08) |
| T1 DM | Pre | Mixed | 956 | 70 | 22919 | 305.42 | 3.3 | (2.47, 4.39) | 2.57 | (1.93, 3.43) | 2.63 | (1.97, 3.5) |
| T1 DM | Pre | Other | 949 | 40 | 23225 | 172.23 | 1.78 | (1.25, 2.53) | 1.58 | (1.11, 2.25) | 1.67 | (1.17, 2.37) |
| T1 DM | Pandemic | White | 100454 | 5530 | 2385604 | 231.81 | 3.18 | (2.68, 3.78) | 2.91 | (2.46, 3.45) | 3.45 | (2.91, 4.09) |
| T1 DM | Pandemic | Asian | 7274 | 143 | 175440 | 81.51 | Reference |  | Reference |  | Reference |  |
| T1 DM | Pandemic | Black | 3004 | 125 | 70660 | 176.9 | 2.16 | (1.7, 2.75) | 2.05 | (1.61, 2.61) | 1.91 | (1.5, 2.43) |
| T1 DM | Pandemic | Mixed | 1180 | 63 | 27871 | 226.04 | 2.92 | (2.17, 3.93) | 2.31 | (1.71, 3.11) | 2.36 | (1.75, 3.18) |
| T1 DM | Pandemic | Other | 1255 | 52 | 29573 | 175.84 | 2.16 | (1.57, 2.98) | 1.91 | (1.39, 2.62) | 2.02 | (1.47, 2.77) |
| T1 DM | Wave 1 | White | 100463 | 616 | 226053 | 272.5 | 3.22 | (1.84, 5.66) | 2.96 | (1.69, 5.19) | 3.74 | (2.13, 6.55) |
| T1 DM | Wave 1 | Asian | 7275 | 13 | 16397 | 79.28 | Reference |  | Reference |  | Reference |  |
| T1 DM | Wave 1 | Black | 3001 | 11 | 6726 | 163.55 | 2.07 | (0.92, 4.61) | 1.97 | (0.88, 4.4) | 1.81 | (0.81, 4.05) |
| T1 DM | Wave 1 | Mixed | redacted |  |  |  |  |  |  |  |  |  |
| T1 DM | Wave 1 | Other | redacted |  |  |  |  |  |  |  |  |  |
| T1 DM | Easing 1 | White | 100755 | 1042 | 325820 | 319.81 | 3.81 | (2.49, 5.82) | 3.39 | (2.22, 5.17) | 4.02 | (2.64, 6.14) |
| T1 DM | Easing 1 | Asian | 7311 | 23 | 23742 | 96.87 | Reference |  | Reference |  | Reference |  |
| T1 DM | Easing 1 | Black | 3027 | 29 | 9786 | 296.35 | 3.05 | (1.76, 5.28) | 2.83 | (1.63, 4.89) | 2.65 | (1.53, 4.58) |
| T1 DM | Easing 1 | Mixed | 1194 | 14 | 3863 | 362.4 | 3.94 | (2.03, 7.67) | 2.98 | (1.53, 5.79) | 3.07 | (1.57, 5.97) |
| T1 DM | Easing 1 | Other | redacted |  |  |  |  |  |  |  |  |  |
| T1 DM | Wave 2 | White | 100749 | 2105 | 749144 | 280.99 | 3.4 | (2.55, 4.53) | 3.08 | (2.31, 4.1) | 3.76 | (2.82, 5.02) |
| T1 DM | Wave 2 | Asian | 7314 | 50 | 54660 | 91.47 | Reference |  | Reference |  | Reference |  |
| T1 DM | Wave 2 | Black | 3052 | 46 | 22633 | 203.24 | 2.22 | (1.48, 3.31) | 2.07 | (1.39, 3.09) | 1.91 | (1.28, 2.86) |
| T1 DM | Wave 2 | Mixed | 1187 | 30 | 8769 | 342.1 | 3.94 | (2.5, 6.2) | 3.02 | (1.92, 4.76) | 3.12 | (1.98, 4.92) |
| T1 DM | Wave 2 | Other | 1263 | 15 | 9374 | 160.02 | 1.77 | (0.99, 3.15) | 1.53 | (0.86, 2.73) | 1.66 | (0.93, 2.95) |
| T1 DM | Easing 2 | White | 100533 | 408 | 106809 | 381.99 | 2.35 | (1.35, 4.07) | 2.12 | (1.23, 3.68) | 2.57 | (1.48, 4.46) |
| T1 DM | Easing 2 | Asian | 7334 | 14 | 7801 | 179.46 | Reference |  | Reference |  | Reference |  |
| T1 DM | Easing 2 | Black | redacted |  |  |  |  |  |  |  |  |  |
| T1 DM | Easing 2 | Mixed | redacted |  |  |  |  |  |  |  |  |  |
| T1 DM | Easing 2 | Other | redacted |  |  |  |  |  |  |  |  |  |
| T1 DM | Wave 3 | White | 100692 | 1975 | 656541 | 300.82 | 2.66 | (2.03, 3.48) | 2.43 | (1.86, 3.17) | 2.85 | (2.17, 3.72) |
| T1 DM | Wave 3 | Asian | 7359 | 58 | 48340 | 119.98 | Reference |  | Reference |  | Reference |  |
| T1 DM | Wave 3 | Black | 3075 | 63 | 19944 | 315.88 | 2.61 | (1.83, 3.74) | 2.46 | (1.72, 3.51) | 2.31 | (1.62, 3.3) |
| T1 DM | Wave 3 | Mixed | 1172 | 27 | 7621 | 354.27 | 3.05 | (1.93, 4.81) | 2.28 | (1.44, 3.6) | 2.37 | (1.5, 3.74) |
| T1 DM | Wave 3 | Other | 1257 | 17 | 8156 | 208.44 | 1.75 | (1.02, 3) | 1.49 | (0.87, 2.57) | 1.58 | (0.92, 2.72) |
| T1 DM | Easing 3 | White | 100982 | 1461 | 450456 | 324.34 | 2.63 | (1.94, 3.56) | 2.41 | (1.78, 3.26) | 2.88 | (2.13, 3.9) |
| T1 DM | Easing 3 | Asian | 7439 | 46 | 33317 | 138.07 | Reference |  | Reference |  | Reference |  |
| T1 DM | Easing 3 | Black | 3126 | 33 | 13941 | 236.72 | 1.7 | (1.09, 2.67) | 1.6 | (1.02, 2.5) | 1.5 | (0.96, 2.35) |
| T1 DM | Easing 3 | Mixed | 1196 | 18 | 5313 | 338.81 | 2.58 | (1.49, 4.45) | 1.93 | (1.12, 3.33) | 2.02 | (1.17, 3.49) |
| T1 DM | Easing 3 | Other | 1243 | 16 | 5550 | 288.3 | 2.12 | (1.2, 3.74) | 1.82 | (1.03, 3.22) | 1.91 | (1.08, 3.39) |
| T2 DM | Pre | White | 791860 | 8195 | 19744444 | 41.51 | 1.59 | (1.47, 1.72) | 1.63 | (1.5, 1.76) | 1.83 | (1.69, 1.99) |
| T2 DM | Pre | Asian | 114073 | 783 | 2849512 | 27.48 | Reference |  | Reference |  | Reference |  |
| T2 DM | Pre | Black | 27906 | 348 | 695157 | 50.06 | 1.75 | (1.54, 1.99) | 1.74 | (1.53, 1.98) | 1.55 | (1.37, 1.76) |
| T2 DM | Pre | Mixed | 8600 | 76 | 214680 | 35.4 | 1.26 | (0.99, 1.6) | 1.26 | (1, 1.6) | 1.22 | (0.96, 1.55) |
| T2 DM | Pre | Other | 11805 | 87 | 294856 | 29.51 | 1.07 | (0.85, 1.33) | 1.07 | (0.86, 1.34) | 1.09 | (0.87, 1.36) |
| T2 DM | Pandemic | White | 941455 | 11040 | 22648478 | 48.74 | 1.92 | (1.79, 2.07) | 1.91 | (1.77, 2.06) | 2.14 | (1.99, 2.31) |
| T2 DM | Pandemic | Asian | 140656 | 892 | 3452607 | 25.84 | Reference |  | Reference |  | Reference |  |
| T2 DM | Pandemic | Black | 35567 | 482 | 861384 | 55.96 | 2.08 | (1.86, 2.32) | 2.06 | (1.84, 2.3) | 1.84 | (1.65, 2.06) |
| T2 DM | Pandemic | Mixed | 11131 | 124 | 271208 | 45.72 | 1.73 | (1.43, 2.08) | 1.73 | (1.44, 2.09) | 1.68 | (1.39, 2.02) |
| T2 DM | Pandemic | Other | 15672 | 122 | 380633 | 32.05 | 1.2 | (0.99, 1.45) | 1.2 | (0.99, 1.45) | 1.21 | (1, 1.47) |
| T2 DM | Wave 1 | White | 941483 | 870 | 2120264 | 41.03 | 2.22 | (1.67, 2.95) | 2.25 | (1.69, 3) | 2.61 | (1.96, 3.48) |
| T2 DM | Wave 1 | Asian | 140644 | 57 | 317405 | 17.96 | Reference |  | Reference |  | Reference |  |
| T2 DM | Wave 1 | Black | 35564 | 40 | 80057 | 49.96 | 2.66 | (1.77, 3.99) | 2.63 | (1.75, 3.95) | 2.29 | (1.52, 3.43) |
| T2 DM | Wave 1 | Mixed | redacted |  |  |  |  |  |  |  |  |  |
| T2 DM | Wave 1 | Other | 15687 | 11 | 35357 | 31.11 | 1.64 | (0.86, 3.13) | 1.64 | (0.86, 3.13) | 1.65 | (0.86, 3.16) |
| T2 DM | Easing 1 | White | 938717 | 1720 | 3046173 | 56.46 | 2.17 | (1.78, 2.63) | 2.13 | (1.74, 2.59) | 2.39 | (1.96, 2.92) |
| T2 DM | Easing 1 | Asian | 141193 | 122 | 459473 | 26.55 | Reference |  | Reference |  | Reference |  |
| T2 DM | Easing 1 | Black | 35668 | 77 | 115876 | 66.45 | 2.36 | (1.77, 3.14) | 2.34 | (1.76, 3.12) | 2.06 | (1.55, 2.75) |
| T2 DM | Easing 1 | Mixed | 11151 | 13 | 36284 | 35.83 | 1.3 | (0.73, 2.3) | 1.31 | (0.74, 2.32) | 1.25 | (0.71, 2.22) |
| T2 DM | Easing 1 | Other | 15777 | 20 | 51264 | 39.01 | 1.42 | (0.88, 2.28) | 1.41 | (0.88, 2.27) | 1.43 | (0.89, 2.3) |
| T2 DM | Wave 2 | White | 939511 | 3523 | 7032361 | 50.1 | 1.89 | (1.66, 2.15) | 1.91 | (1.68, 2.18) | 2.18 | (1.91, 2.48) |
| T2 DM | Wave 2 | Asian | 141615 | 291 | 1065878 | 27.3 | Reference |  | Reference |  | Reference |  |
| T2 DM | Wave 2 | Black | 35811 | 160 | 268616 | 59.56 | 2.1 | (1.73, 2.55) | 2.09 | (1.72, 2.54) | 1.86 | (1.53, 2.26) |
| T2 DM | Wave 2 | Mixed | 11220 | 46 | 84263 | 54.59 | 1.96 | (1.43, 2.67) | 1.96 | (1.44, 2.68) | 1.9 | (1.39, 2.59) |
| T2 DM | Wave 2 | Other | 15915 | 43 | 119405 | 36.01 | 1.27 | (0.92, 1.75) | 1.27 | (0.92, 1.75) | 1.29 | (0.93, 1.77) |
| T2 DM | Easing 2 | White | 945374 | 624 | 1006055 | 62.02 | 1.58 | (1.19, 2.1) | 1.55 | (1.16, 2.07) | 1.75 | (1.31, 2.34) |
| T2 DM | Easing 2 | Asian | 142718 | 61 | 151971 | 40.14 | Reference |  | Reference |  | Reference |  |
| T2 DM | Easing 2 | Black | 36188 | 31 | 38532 | 80.45 | 1.91 | (1.23, 2.95) | 1.89 | (1.22, 2.92) | 1.65 | (1.07, 2.56) |
| T2 DM | Easing 2 | Mixed | redacted |  |  |  |  |  |  |  |  |  |
| T2 DM | Easing 2 | Other | redacted |  |  |  |  |  |  |  |  |  |
| T2 DM | Wave 3 | White | 947473 | 3295 | 6218927 | 52.98 | 1.77 | (1.55, 2.01) | 1.75 | (1.53, 1.99) | 1.96 | (1.72, 2.24) |
| T2 DM | Wave 3 | Asian | 143015 | 289 | 943099 | 30.64 | Reference |  | Reference |  | Reference |  |
| T2 DM | Wave 3 | Black | 36330 | 127 | 238909 | 53.16 | 1.65 | (1.34, 2.03) | 1.65 | (1.33, 2.03) | 1.47 | (1.19, 1.81) |
| T2 DM | Wave 3 | Mixed | 11430 | 44 | 75226 | 58.49 | 1.84 | (1.34, 2.53) | 1.86 | (1.35, 2.55) | 1.8 | (1.31, 2.47) |
| T2 DM | Wave 3 | Other | 16203 | 39 | 106510 | 36.62 | 1.15 | (0.82, 1.61) | 1.15 | (0.82, 1.6) | 1.16 | (0.83, 1.63) |
| T2 DM | Easing 3 | White | 953549 | 2229 | 4273597 | 52.16 | 1.98 | (1.69, 2.33) | 1.96 | (1.66, 2.31) | 2.16 | (1.83, 2.55) |
| T2 DM | Easing 3 | Asian | 144636 | 182 | 650752 | 27.97 | Reference |  | Reference |  | Reference |  |
| T2 DM | Easing 3 | Black | 36828 | 113 | 165179 | 68.41 | 2.38 | (1.88, 3.01) | 2.37 | (1.87, 3) | 2.14 | (1.69, 2.72) |
| T2 DM | Easing 3 | Mixed | 11643 | 32 | 52210 | 61.29 | 2.2 | (1.51, 3.21) | 2.21 | (1.52, 3.22) | 2.14 | (1.47, 3.12) |
| T2 DM | Easing 3 | Other | 16434 | 13 | 73768 | 17.62 | 0.62 | (0.35, 1.09) | 0.62 | (0.35, 1.08) | 0.62 | (0.35, 1.09) |
| Anxiety | Pre | White | 11951915 | 4087 | 299505903 | 1.36 | 1.13 | (1, 1.28) | 1.07 | (0.94, 1.21) | 1.24 | (1.09, 1.41) |
| Anxiety | Pre | Asian | 947198 | 279 | 23736235 | 1.18 | Reference |  | Reference |  | Reference |  |
| Anxiety | Pre | Black | 323687 | 60 | 8111470 | 0.74 | 0.61 | (0.46, 0.81) | 0.61 | (0.46, 0.81) | 0.58 | (0.44, 0.77) |
| Anxiety | Pre | Mixed | 167862 | 42 | 4206466 | 1 | 0.84 | (0.61, 1.16) | 0.84 | (0.61, 1.16) | 0.88 | (0.64, 1.22) |
| Anxiety | Pre | Other | 255875 | 53 | 6411343 | 0.83 | 0.74 | (0.55, 1) | 0.75 | (0.56, 1) | 0.83 | (0.62, 1.11) |
| Anxiety | Pandemic | White | 13007851 | 4604 | 321888666 | 1.43 | 1.15 | (1.03, 1.3) | 1.11 | (0.99, 1.26) | 1.28 | (1.13, 1.44) |
| Anxiety | Pandemic | Asian | 1116587 | 316 | 27562426 | 1.15 | Reference |  | Reference |  | Reference |  |
| Anxiety | Pandemic | Black | 389040 | 80 | 9540013 | 0.84 | 0.71 | (0.56, 0.91) | 0.71 | (0.56, 0.91) | 0.68 | (0.53, 0.87) |
| Anxiety | Pandemic | Mixed | 206060 | 68 | 5035801 | 1.35 | 1.16 | (0.89, 1.51) | 1.15 | (0.88, 1.49) | 1.21 | (0.93, 1.57) |
| Anxiety | Pandemic | Other | 334275 | 76 | 8015275 | 0.95 | 0.86 | (0.67, 1.11) | 0.86 | (0.67, 1.11) | 0.94 | (0.73, 1.21) |
| Anxiety | Wave 1 | White | 13008075 | 365 | 29413099 | 1.24 | 0.94 | (0.63, 1.41) | 0.9 | (0.6, 1.35) | 1.07 | (0.71, 1.61) |
| Anxiety | Wave 1 | Asian | 1116590 | 27 | 2525244 | 1.07 | Reference |  | Reference |  | Reference |  |
| Anxiety | Wave 1 | Black | redacted |  |  |  |  |  |  |  |  |  |
| Anxiety | Wave 1 | Mixed | redacted |  |  |  |  |  |  |  |  |  |
| Anxiety | Wave 1 | Other | redacted |  |  |  |  |  |  |  |  |  |
| Anxiety | Easing 1 | White | 13059230 | 725 | 42519578 | 1.71 | 1.25 | (0.92, 1.71) | 1.17 | (0.86, 1.6) | 1.39 | (1.02, 1.91) |
| Anxiety | Easing 1 | Asian | 1128788 | 45 | 3674977 | 1.22 | Reference |  | Reference |  | Reference |  |
| Anxiety | Easing 1 | Black | redacted |  |  |  |  |  |  |  |  |  |
| Anxiety | Easing 1 | Mixed | 208793 | 15 | 678914 | 2.21 | 1.74 | (0.97, 3.12) | 1.73 | (0.96, 3.12) | 1.84 | (1.02, 3.3) |
| Anxiety | Easing 1 | Other | 338288 | 12 | 1098296 | 1.09 | 0.94 | (0.5, 1.78) | 0.94 | (0.5, 1.79) | 1.05 | (0.56, 2) |
| Anxiety | Wave 2 | White | 12967234 | 1399 | 97842210 | 1.43 | 1.36 | (1.08, 1.72) | 1.29 | (1.02, 1.63) | 1.47 | (1.16, 1.85) |
| Anxiety | Wave 2 | Asian | 1122609 | 82 | 8461143 | 0.97 | Reference |  | Reference |  | Reference |  |
| Anxiety | Wave 2 | Black | 390037 | 29 | 2935073 | 0.99 | 1.01 | (0.66, 1.54) | 1 | (0.66, 1.54) | 0.96 | (0.63, 1.47) |
| Anxiety | Wave 2 | Mixed | 207205 | 27 | 1555417 | 1.74 | 1.77 | (1.14, 2.74) | 1.76 | (1.14, 2.72) | 1.84 | (1.19, 2.85) |
| Anxiety | Wave 2 | Other | 332902 | 17 | 2485666 | 0.68 | 0.74 | (0.44, 1.25) | 0.74 | (0.44, 1.25) | 0.81 | (0.48, 1.36) |
| Anxiety | Easing 2 | White | 12640489 | 237 | 13462945 | 1.76 | 1.03 | (0.63, 1.69) | 0.97 | (0.59, 1.59) | 1.12 | (0.68, 1.84) |
| Anxiety | Easing 2 | Asian | 1095138 | 19 | 1166268 | 1.63 | Reference |  | Reference |  | Reference |  |
| Anxiety | Easing 2 | Black | redacted |  |  |  |  |  |  |  |  |  |
| Anxiety | Easing 2 | Mixed | redacted |  |  |  |  |  |  |  |  |  |
| Anxiety | Easing 2 | Other | redacted |  |  |  |  |  |  |  |  |  |
| Anxiety | Wave 3 | White | 12602332 | 1185 | 83187132 | 1.42 | 0.97 | (0.78, 1.21) | 0.95 | (0.76, 1.19) | 1.07 | (0.86, 1.34) |
| Anxiety | Wave 3 | Asian | 1091692 | 94 | 7195031 | 1.31 | Reference |  | Reference |  | Reference |  |
| Anxiety | Wave 3 | Black | 377686 | 21 | 2483064 | 0.85 | 0.6 | (0.38, 0.97) | 0.61 | (0.38, 0.98) | 0.58 | (0.36, 0.94) |
| Anxiety | Wave 3 | Mixed | 200227 | 15 | 1315159 | 1.14 | 0.84 | (0.49, 1.45) | 0.82 | (0.47, 1.42) | 0.86 | (0.5, 1.48) |
| Anxiety | Wave 3 | Other | 314768 | 22 | 2054920 | 1.07 | 0.85 | (0.54, 1.36) | 0.85 | (0.53, 1.35) | 0.91 | (0.57, 1.46) |
| Anxiety | Easing 3 | White | 12411761 | 691 | 55915750 | 1.24 | 1.19 | (0.88, 1.62) | 1.18 | (0.87, 1.6) | 1.34 | (0.98, 1.82) |
| Anxiety | Easing 3 | Asian | 1074699 | 48 | 4838179 | 0.99 | Reference |  | Reference |  | Reference |  |
| Anxiety | Easing 3 | Black | redacted |  |  |  |  |  |  |  |  |  |
| Anxiety | Easing 3 | Mixed | redacted |  |  |  |  |  |  |  |  |  |
| Anxiety | Easing 3 | Other | 304150 | 11 | 1360271 | 0.81 | 0.86 | (0.45, 1.66) | 0.86 | (0.45, 1.66) | 0.94 | (0.48, 1.81) |
| Depression | Pre | White | 11951915 | 2218 | 299528329 | 0.74 | 2.02 | (1.61, 2.52) | 2.03 | (1.62, 2.53) | 2.47 | (1.98, 3.1) |
| Depression | Pre | Asian | 947198 | 83 | 23738551 | 0.35 | Reference |  | Reference |  | Reference |  |
| Depression | Pre | Black | 323687 | 35 | 8111737 | 0.43 | 1.05 | (0.71, 1.57) | 1.07 | (0.72, 1.59) | 1 | (0.67, 1.48) |
| Depression | Pre | Mixed | 167862 | 35 | 4206541 | 0.83 | 2.17 | (1.46, 3.22) | 2.14 | (1.44, 3.18) | 2.29 | (1.54, 3.4) |
| Depression | Pre | Other | 255875 | 23 | 6411754 | 0.36 | 1.07 | (0.67, 1.7) | 1.06 | (0.67, 1.69) | 1.2 | (0.75, 1.9) |
| Depression | Pandemic | White | 13007854 | 2181 | 321918259 | 0.68 | 1.59 | (1.29, 1.96) | 1.48 | (1.2, 1.82) | 1.76 | (1.42, 2.17) |
| Depression | Pandemic | Asian | 1116587 | 97 | 27564956 | 0.35 | Reference |  | Reference |  | Reference |  |
| Depression | Pandemic | Black | 389040 | 41 | 9540463 | 0.43 | 1.02 | (0.71, 1.48) | 1.04 | (0.72, 1.5) | 0.98 | (0.68, 1.41) |
| Depression | Pandemic | Mixed | 206060 | 27 | 5036547 | 0.54 | 1.31 | (0.85, 2.01) | 1.32 | (0.86, 2.03) | 1.4 | (0.91, 2.15) |
| Depression | Pandemic | Other | 334275 | 29 | 8015812 | 0.36 | 1.01 | (0.67, 1.54) | 1.02 | (0.67, 1.54) | 1.12 | (0.74, 1.69) |
| Depression | Wave 1 | White | 13008078 | 185 | 29413291 | 0.63 | 1.14 | (0.55, 2.36) | 1.05 | (0.51, 2.18) | 1.2 | (0.58, 2.5) |
| Depression | Wave 1 | Asian | redacted |  |  |  |  |  |  |  |  |  |
| Depression | Wave 1 | Black | redacted |  |  |  |  |  |  |  |  |  |
| Depression | Wave 1 | Mixed | redacted |  |  |  |  |  |  |  |  |  |
| Depression | Wave 1 | Other | redacted |  |  |  |  |  |  |  |  |  |
| Depression | Easing 1 | White | 13059246 | 406 | 42520051 | 0.95 | 1.65 | (0.99, 2.75) | 1.58 | (0.94, 2.63) | 1.93 | (1.15, 3.23) |
| Depression | Easing 1 | Asian | 1128788 | 16 | 3675015 | 0.44 | Reference |  | Reference |  | Reference |  |
| Depression | Easing 1 | Black | 393362 | 12 | 1279474 | 0.94 | 1.66 | (0.78, 3.52) | 1.67 | (0.79, 3.55) | 1.57 | (0.74, 3.32) |
| Depression | Easing 1 | Mixed | redacted |  |  |  |  |  |  |  |  |  |
| Depression | Easing 1 | Other | redacted |  |  |  |  |  |  |  |  |  |
| Depression | Wave 2 | White | 12967236 | 697 | 97844796 | 0.71 | 1.82 | (1.24, 2.66) | 1.71 | (1.16, 2.5) | 2 | (1.36, 2.94) |
| Depression | Wave 2 | Asian | 1122609 | 29 | 8461282 | 0.34 | Reference |  | Reference |  | Reference |  |
| Depression | Wave 2 | Black | 390037 | 12 | 2935109 | 0.41 | 1.01 | (0.52, 1.99) | 1.03 | (0.53, 2.02) | 0.97 | (0.49, 1.91) |
| Depression | Wave 2 | Mixed | redacted |  |  |  |  |  |  |  |  |  |
| Depression | Wave 2 | Other | redacted |  |  |  |  |  |  |  |  |  |
| Depression | Easing 2 | White | 12640487 | 108 | 13463005 | 0.8 | 1.23 | (0.52, 2.87) | 1.1 | (0.47, 2.59) | 1.23 | (0.52, 2.9) |
| Depression | Easing 2 | Asian | redacted |  |  |  |  |  |  |  |  |  |
| Depression | Easing 2 | Black | redacted |  |  |  |  |  |  |  |  |  |
| Depression | Easing 2 | Mixed | redacted |  |  |  |  |  |  |  |  |  |
| Depression | Easing 2 | Other | redacted |  |  |  |  |  |  |  |  |  |
| Depression | Wave 3 | White | 12602338 | 504 | 83189402 | 0.61 | 1.34 | (0.89, 2) | 1.23 | (0.82, 1.85) | 1.48 | (0.98, 2.23) |
| Depression | Wave 3 | Asian | 1091692 | 26 | 7195242 | 0.36 | Reference |  | Reference |  | Reference |  |
| Depression | Wave 3 | Black | redacted |  |  |  |  |  |  |  |  |  |
| Depression | Wave 3 | Mixed | redacted |  |  |  |  |  |  |  |  |  |
| Depression | Wave 3 | Other | redacted |  |  |  |  |  |  |  |  |  |
| Depression | Easing 3 | White | 12411765 | 310 | 55916565 | 0.55 | 1.67 | (0.98, 2.85) | 1.5 | (0.88, 2.57) | 1.81 | (1.06, 3.12) |
| Depression | Easing 3 | Asian | 1074699 | 15 | 4838248 | 0.31 | Reference |  | Reference |  | Reference |  |
| Depression | Easing 3 | Black | redacted |  |  |  |  |  |  |  |  |  |
| Depression | Easing 3 | Mixed | redacted |  |  |  |  |  |  |  |  |  |
| Depression | Easing 3 | Other | redacted |  |  |  |  |  |  |  |  |  |
| Asthma | Pre | White | 1104943 | 14508 | 27504757 | 52.75 | 0.56 | (0.53, 0.59) | 0.55 | (0.52, 0.57) | 0.64 | (0.61, 0.67) |
| Asthma | Pre | Asian | 72238 | 1870 | 1785399 | 104.74 | Reference |  | Reference |  | Reference |  |
| Asthma | Pre | Black | 21726 | 436 | 538585 | 80.95 | 0.79 | (0.71, 0.88) | 0.77 | (0.69, 0.85) | 0.74 | (0.67, 0.82) |
| Asthma | Pre | Mixed | 14090 | 227 | 350142 | 64.83 | 0.65 | (0.57, 0.75) | 0.64 | (0.55, 0.73) | 0.7 | (0.61, 0.8) |
| Asthma | Pre | Other | 11220 | 176 | 278865 | 63.11 | 0.63 | (0.54, 0.73) | 0.62 | (0.53, 0.73) | 0.73 | (0.62, 0.85) |
| Asthma | Pandemic | White | 1230577 | 9804 | 30424471 | 32.22 | 0.66 | (0.62, 0.71) | 0.65 | (0.61, 0.7) | 0.75 | (0.7, 0.8) |
| Asthma | Pandemic | Asian | 84572 | 1128 | 2090544 | 53.96 | Reference |  | Reference |  | Reference |  |
| Asthma | Pandemic | Black | 25738 | 279 | 632429 | 44.12 | 0.82 | (0.72, 0.94) | 0.79 | (0.69, 0.9) | 0.77 | (0.67, 0.88) |
| Asthma | Pandemic | Mixed | 17059 | 155 | 418542 | 37.03 | 0.72 | (0.61, 0.85) | 0.69 | (0.58, 0.81) | 0.75 | (0.63, 0.88) |
| Asthma | Pandemic | Other | 13884 | 115 | 340269 | 33.8 | 0.66 | (0.54, 0.8) | 0.65 | (0.53, 0.79) | 0.74 | (0.61, 0.9) |
| Asthma | Wave 1 | White | 1230560 | 1011 | 2780998 | 36.35 | 0.98 | (0.76, 1.26) | 0.97 | (0.75, 1.25) | 1.15 | (0.88, 1.49) |
| Asthma | Wave 1 | Asian | 84566 | 68 | 191126 | 35.58 | Reference |  | Reference |  | Reference |  |
| Asthma | Wave 1 | Black | 25739 | 18 | 58115 | 30.97 | 0.95 | (0.56, 1.59) | 0.91 | (0.54, 1.53) | 0.88 | (0.52, 1.48) |
| Asthma | Wave 1 | Mixed | 17045 | 12 | 38540 | 31.14 | 0.9 | (0.49, 1.67) | 0.87 | (0.47, 1.62) | 0.98 | (0.53, 1.82) |
| Asthma | Wave 1 | Other | redacted |  |  |  |  |  |  |  |  |  |
| Asthma | Easing 1 | White | 1239227 | 1453 | 4033325 | 36.02 | 0.95 | (0.77, 1.16) | 0.94 | (0.77, 1.16) | 1.14 | (0.93, 1.41) |
| Asthma | Easing 1 | Asian | 85465 | 110 | 278243 | 39.53 | Reference |  | Reference |  | Reference |  |
| Asthma | Easing 1 | Black | 25983 | 34 | 84514 | 40.23 | 1 | (0.68, 1.47) | 0.96 | (0.65, 1.41) | 0.93 | (0.64, 1.37) |
| Asthma | Easing 1 | Mixed | 17414 | 19 | 56614 | 33.56 | 0.85 | (0.52, 1.39) | 0.81 | (0.5, 1.33) | 0.92 | (0.56, 1.5) |
| Asthma | Easing 1 | Other | 14127 | 16 | 45931 | 34.83 | 0.92 | (0.54, 1.56) | 0.9 | (0.53, 1.53) | 1.12 | (0.66, 1.9) |
| Asthma | Wave 2 | White | 1235344 | 3169 | 9316516 | 34.01 | 0.78 | (0.68, 0.88) | 0.77 | (0.68, 0.88) | 0.91 | (0.81, 1.03) |
| Asthma | Wave 2 | Asian | 85717 | 311 | 646184 | 48.13 | Reference |  | Reference |  | Reference |  |
| Asthma | Wave 2 | Black | 25961 | 101 | 195315 | 51.71 | 1.08 | (0.86, 1.35) | 1.03 | (0.82, 1.3) | 1 | (0.8, 1.26) |
| Asthma | Wave 2 | Mixed | 17437 | 42 | 130946 | 32.07 | 0.7 | (0.5, 0.96) | 0.65 | (0.47, 0.9) | 0.72 | (0.52, 0.99) |
| Asthma | Wave 2 | Other | 14058 | 33 | 105601 | 31.25 | 0.68 | (0.48, 0.98) | 0.67 | (0.47, 0.96) | 0.8 | (0.56, 1.15) |
| Asthma | Easing 2 | White | 1207813 | 677 | 1286418 | 52.63 | 0.67 | (0.53, 0.86) | 0.68 | (0.53, 0.86) | 0.81 | (0.63, 1.04) |
| Asthma | Easing 2 | Asian | 84468 | 84 | 89957 | 93.38 | Reference |  | Reference |  | Reference |  |
| Asthma | Easing 2 | Black | 25562 | 20 | 27222 | 73.47 | 0.8 | (0.49, 1.3) | 0.77 | (0.47, 1.25) | 0.75 | (0.46, 1.22) |
| Asthma | Easing 2 | Mixed | redacted |  |  |  |  |  |  |  |  |  |
| Asthma | Easing 2 | Other | redacted |  |  |  |  |  |  |  |  |  |
| Asthma | Wave 3 | White | 1207064 | 3813 | 7964754 | 47.87 | 0.66 | (0.6, 0.73) | 0.65 | (0.59, 0.72) | 0.75 | (0.68, 0.83) |
| Asthma | Wave 3 | Asian | 84457 | 482 | 556459 | 86.62 | Reference |  | Reference |  | Reference |  |
| Asthma | Wave 3 | Black | 25521 | 120 | 168043 | 71.41 | 0.83 | (0.68, 1.01) | 0.79 | (0.65, 0.97) | 0.77 | (0.63, 0.95) |
| Asthma | Wave 3 | Mixed | 17084 | 75 | 112349 | 66.76 | 0.83 | (0.65, 1.06) | 0.77 | (0.61, 0.99) | 0.84 | (0.66, 1.08) |
| Asthma | Wave 3 | Other | 13693 | 52 | 89958 | 57.8 | 0.69 | (0.52, 0.92) | 0.67 | (0.51, 0.9) | 0.77 | (0.58, 1.03) |
| Asthma | Easing 3 | White | 1197790 | 2330 | 5394041 | 43.2 | 0.59 | (0.52, 0.67) | 0.58 | (0.51, 0.66) | 0.67 | (0.59, 0.76) |
| Asthma | Easing 3 | Asian | 84615 | 327 | 380842 | 85.86 | Reference |  | Reference |  | Reference |  |
| Asthma | Easing 3 | Black | 25401 | 76 | 114225 | 66.54 | 0.78 | (0.6, 1) | 0.75 | (0.58, 0.96) | 0.72 | (0.56, 0.93) |
| Asthma | Easing 3 | Mixed | 16959 | 46 | 76258 | 60.32 | 0.75 | (0.55, 1.03) | 0.73 | (0.53, 0.99) | 0.79 | (0.58, 1.07) |
| Asthma | Easing 3 | Other | 13585 | 35 | 61096 | 57.29 | 0.71 | (0.5, 1) | 0.7 | (0.49, 0.99) | 0.8 | (0.56, 1.14) |
| COPD | Pre | White | 409471 | 34264 | 9831716 | 348.5 | 1.37 | (1.26, 1.5) | 1.3 | (1.19, 1.42) | 1.37 | (1.26, 1.5) |
| COPD | Pre | Asian | 8290 | 531 | 200489 | 264.85 | Reference |  | Reference |  | Reference |  |
| COPD | Pre | Black | 2556 | 144 | 62265 | 231.27 | 0.87 | (0.73, 1.05) | 0.89 | (0.74, 1.06) | 0.86 | (0.72, 1.04) |
| COPD | Pre | Mixed | 1379 | 107 | 33202 | 322.27 | 1.26 | (1.02, 1.55) | 1.28 | (1.04, 1.57) | 1.32 | (1.07, 1.62) |
| COPD | Pre | Other | 1851 | 123 | 44939 | 273.7 | 1.07 | (0.88, 1.3) | 1.07 | (0.88, 1.3) | 1.12 | (0.92, 1.36) |
| COPD | Pandemic | White | 497200 | 33511 | 11478967 | 291.93 | 1.63 | (1.48, 1.79) | 1.55 | (1.4, 1.7) | 1.67 | (1.52, 1.84) |
| COPD | Pandemic | Asian | 10220 | 443 | 240147 | 184.47 | Reference |  | Reference |  | Reference |  |
| COPD | Pandemic | Black | 3277 | 138 | 77025 | 179.16 | 0.98 | (0.81, 1.18) | 1 | (0.83, 1.21) | 0.97 | (0.8, 1.18) |
| COPD | Pandemic | Mixed | 1766 | 92 | 41773 | 220.24 | 1.23 | (0.98, 1.54) | 1.29 | (1.03, 1.61) | 1.36 | (1.09, 1.7) |
| COPD | Pandemic | Other | 2405 | 97 | 56013 | 173.17 | 0.98 | (0.78, 1.22) | 0.98 | (0.79, 1.22) | 1.05 | (0.84, 1.3) |
| COPD | Wave 1 | White | 497196 | 3088 | 1114997 | 276.95 | 2.05 | (1.44, 2.92) | 1.98 | (1.39, 2.82) | 2.18 | (1.53, 3.1) |
| COPD | Wave 1 | Asian | 10221 | 32 | 22957 | 139.39 | Reference |  | Reference |  | Reference |  |
| COPD | Wave 1 | Black | redacted |  |  |  |  |  |  |  |  |  |
| COPD | Wave 1 | Mixed | redacted |  |  |  |  |  |  |  |  |  |
| COPD | Wave 1 | Other | redacted |  |  |  |  |  |  |  |  |  |
| COPD | Easing 1 | White | 493737 | 5495 | 1591832 | 345.2 | 1.89 | (1.48, 2.43) | 1.81 | (1.41, 2.33) | 1.98 | (1.54, 2.54) |
| COPD | Easing 1 | Asian | 10226 | 64 | 33100 | 193.36 | Reference |  | Reference |  | Reference |  |
| COPD | Easing 1 | Black | 3267 | 22 | 10548 | 208.57 | 1.05 | (0.65, 1.71) | 1.08 | (0.66, 1.75) | 1.06 | (0.65, 1.72) |
| COPD | Easing 1 | Mixed | 1768 | 14 | 5735 | 244.1 | 1.29 | (0.72, 2.3) | 1.35 | (0.75, 2.4) | 1.43 | (0.8, 2.55) |
| COPD | Easing 1 | Other | 2407 | 16 | 7765 | 206.06 | 1.08 | (0.62, 1.86) | 1.08 | (0.62, 1.87) | 1.16 | (0.67, 2.01) |
| COPD | Wave 2 | White | 489153 | 10994 | 3607014 | 304.8 | 1.61 | (1.36, 1.9) | 1.53 | (1.3, 1.81) | 1.66 | (1.41, 1.96) |
| COPD | Wave 2 | Asian | 10158 | 146 | 75174 | 194.22 | Reference |  | Reference |  | Reference |  |
| COPD | Wave 2 | Black | 3229 | 50 | 23911 | 209.11 | 1.09 | (0.79, 1.5) | 1.11 | (0.81, 1.53) | 1.08 | (0.78, 1.48) |
| COPD | Wave 2 | Mixed | 1784 | 30 | 13200 | 227.26 | 1.2 | (0.81, 1.78) | 1.27 | (0.85, 1.88) | 1.34 | (0.9, 1.98) |
| COPD | Wave 2 | Other | 2372 | 32 | 17549 | 182.35 | 0.98 | (0.67, 1.43) | 0.98 | (0.67, 1.44) | 1.04 | (0.71, 1.53) |
| COPD | Easing 2 | White | 478271 | 2532 | 507374 | 499.04 | 1.79 | (1.24, 2.58) | 1.66 | (1.15, 2.39) | 1.82 | (1.26, 2.63) |
| COPD | Easing 2 | Asian | 10036 | 30 | 10656 | 281.52 | Reference |  | Reference |  | Reference |  |
| COPD | Easing 2 | Black | 3242 | 11 | 3446 | 319.17 | 1.14 | (0.57, 2.27) | 1.16 | (0.58, 2.31) | 1.14 | (0.57, 2.27) |
| COPD | Easing 2 | Mixed | redacted |  |  |  |  |  |  |  |  |  |
| COPD | Easing 2 | Other | redacted |  |  |  |  |  |  |  |  |  |
| COPD | Wave 3 | White | 477395 | 12942 | 3083669 | 419.69 | 1.56 | (1.35, 1.82) | 1.46 | (1.26, 1.7) | 1.59 | (1.37, 1.85) |
| COPD | Wave 3 | Asian | 10023 | 181 | 65161 | 277.77 | Reference |  | Reference |  | Reference |  |
| COPD | Wave 3 | Black | 3229 | 51 | 21022 | 242.61 | 0.89 | (0.65, 1.22) | 0.9 | (0.66, 1.23) | 0.87 | (0.64, 1.19) |
| COPD | Wave 3 | Mixed | 1809 | 30 | 11751 | 255.29 | 0.96 | (0.65, 1.41) | 0.99 | (0.67, 1.46) | 1.05 | (0.71, 1.55) |
| COPD | Wave 3 | Other | 2422 | 42 | 15769 | 266.35 | 1.01 | (0.72, 1.42) | 1.01 | (0.72, 1.42) | 1.09 | (0.78, 1.53) |
| COPD | Easing 3 | White | 472883 | 9016 | 2093051 | 430.76 | 1.5 | (1.26, 1.78) | 1.4 | (1.18, 1.67) | 1.53 | (1.29, 1.83) |
| COPD | Easing 3 | Asian | 9987 | 132 | 44390 | 297.37 | Reference |  | Reference |  | Reference |  |
| COPD | Easing 3 | Black | 3230 | 41 | 14355 | 285.62 | 0.99 | (0.69, 1.4) | 1 | (0.71, 1.43) | 0.97 | (0.69, 1.38) |
| COPD | Easing 3 | Mixed | 1849 | 31 | 8222 | 377.01 | 1.33 | (0.9, 1.97) | 1.41 | (0.95, 2.08) | 1.5 | (1.02, 2.22) |
| COPD | Easing 3 | Other | 2428 | 31 | 10808 | 286.83 | 1.04 | (0.7, 1.54) | 1.05 | (0.71, 1.55) | 1.13 | (0.76, 1.67) |

### Table 5: : Number of events, rate, hazard ratio and confidence intervals by ethnic group, time period and outcome with those of Black ethnicity as the reference group.

| **Outcome** | **Period** | **Ethnicity** | **Denominator** | **Events** | **Total person weeks** | **Rate** | **Unadjusted** | | **Age and sex adjusted** | | **Fully adjusted** | |
| --- | --- | --- | --- | --- | --- | --- | --- | --- | --- | --- | --- | --- |
|  |  |  |  |  |  |  | **Hazard ratio** | **95% confidence interval** | **Hazard ratio** | **95% confidence interval** | **Hazard ratio** | **95% confidence interval** |
| Stroke | Pre | White | 11951861 | 42033 | 299064047 | 14.05 | 1.47 | (1.36, 1.59) | 0.86 | (0.8, 0.93) | 1.02 | (0.94, 1.1) |
| Stroke | Pre | Asian | 947198 | 1835 | 23717609 | 7.74 | 0.91 | (0.83, 1) | 0.93 | (0.86, 1.02) | 0.98 | (0.9, 1.07) |
| Stroke | Pre | Black | 323687 | 683 | 8104085 | 8.43 | Reference |  | Reference |  | Reference |  |
| Stroke | Pre | Mixed | 167862 | 249 | 4204083 | 5.92 | 0.68 | (0.58, 0.78) | 0.91 | (0.78, 1.05) | 0.99 | (0.85, 1.14) |
| Stroke | Pre | Other | 255875 | 311 | 6408427 | 4.85 | 0.57 | (0.5, 0.65) | 0.67 | (0.59, 0.77) | 0.76 | (0.67, 0.87) |
| Stroke | Pandemic | White | 13007778 | 62025 | 321371341 | 19.3 | 1.47 | (1.38, 1.56) | 0.84 | (0.79, 0.89) | 0.98 | (0.92, 1.04) |
| Stroke | Pandemic | Asian | 1116583 | 2669 | 27539949 | 9.69 | 0.83 | (0.77, 0.89) | 0.86 | (0.8, 0.92) | 0.9 | (0.84, 0.97) |
| Stroke | Pandemic | Black | 389039 | 1107 | 9530751 | 11.62 | Reference |  | Reference |  | Reference |  |
| Stroke | Pandemic | Mixed | 206060 | 357 | 5033434 | 7.09 | 0.59 | (0.52, 0.66) | 0.82 | (0.73, 0.92) | 0.88 | (0.78, 0.99) |
| Stroke | Pandemic | Other | 334274 | 485 | 8011353 | 6.05 | 0.52 | (0.47, 0.58) | 0.65 | (0.58, 0.72) | 0.72 | (0.65, 0.81) |
| Stroke | Wave 1 | White | 13008002 | 4886 | 29408734 | 16.61 | 1.61 | (1.29, 2.02) | 0.92 | (0.74, 1.16) | 1.09 | (0.87, 1.37) |
| Stroke | Wave 1 | Asian | 1116586 | 210 | 2525075 | 8.32 | 0.9 | (0.7, 1.17) | 0.94 | (0.72, 1.21) | 0.98 | (0.76, 1.27) |
| Stroke | Wave 1 | Black | 389021 | 79 | 879114 | 8.99 | Reference |  | Reference |  | Reference |  |
| Stroke | Wave 1 | Mixed | 206091 | 22 | 465920 | 4.72 | 0.5 | (0.31, 0.8) | 0.71 | (0.44, 1.14) | 0.77 | (0.48, 1.23) |
| Stroke | Wave 1 | Other | 334305 | 35 | 754927 | 4.64 | 0.5 | (0.33, 0.74) | 0.64 | (0.43, 0.96) | 0.72 | (0.49, 1.08) |
| Stroke | Easing 1 | White | 13059172 | 8104 | 42509205 | 19.06 | 1.49 | (1.26, 1.76) | 0.84 | (0.71, 1) | 0.98 | (0.83, 1.16) |
| Stroke | Easing 1 | Asian | 1128784 | 343 | 3674505 | 9.33 | 0.83 | (0.68, 1) | 0.86 | (0.7, 1.04) | 0.89 | (0.73, 1.09) |
| Stroke | Easing 1 | Black | 393360 | 143 | 1279287 | 11.18 | Reference |  | Reference |  | Reference |  |
| Stroke | Easing 1 | Mixed | 208793 | 49 | 678885 | 7.22 | 0.62 | (0.45, 0.86) | 0.87 | (0.63, 1.2) | 0.93 | (0.67, 1.29) |
| Stroke | Easing 1 | Other | 338288 | 66 | 1098220 | 6.01 | 0.54 | (0.4, 0.72) | 0.68 | (0.51, 0.91) | 0.76 | (0.57, 1.02) |
| Stroke | Wave 2 | White | 12967133 | 19186 | 97788237 | 19.62 | 1.51 | (1.35, 1.68) | 0.86 | (0.77, 0.96) | 1 | (0.9, 1.12) |
| Stroke | Wave 2 | Asian | 1122606 | 839 | 8458675 | 9.92 | 0.85 | (0.75, 0.97) | 0.89 | (0.79, 1.01) | 0.93 | (0.82, 1.06) |
| Stroke | Wave 2 | Black | 390035 | 338 | 2934168 | 11.52 | Reference |  | Reference |  | Reference |  |
| Stroke | Wave 2 | Mixed | 207204 | 115 | 1555135 | 7.39 | 0.62 | (0.5, 0.77) | 0.87 | (0.71, 1.08) | 0.94 | (0.76, 1.16) |
| Stroke | Wave 2 | Other | 332901 | 156 | 2485211 | 6.28 | 0.54 | (0.45, 0.66) | 0.69 | (0.57, 0.83) | 0.77 | (0.63, 0.93) |
| Stroke | Easing 2 | White | 12640411 | 2984 | 13461555 | 22.17 | 1.31 | (1.01, 1.7) | 0.75 | (0.58, 0.98) | 0.9 | (0.69, 1.17) |
| Stroke | Easing 2 | Asian | 1095134 | 114 | 1166215 | 9.78 | 0.66 | (0.48, 0.9) | 0.68 | (0.49, 0.93) | 0.72 | (0.52, 0.98) |
| Stroke | Easing 2 | Black | 379052 | 59 | 403523 | 14.62 | Reference |  | Reference |  | Reference |  |
| Stroke | Easing 2 | Mixed | 201010 | 12 | 213928 | 5.61 | 0.37 | (0.2, 0.68) | 0.51 | (0.27, 0.95) | 0.56 | (0.3, 1.03) |
| Stroke | Easing 2 | Other | 317013 | 23 | 337061 | 6.82 | 0.45 | (0.28, 0.73) | 0.56 | (0.35, 0.91) | 0.64 | (0.39, 1.04) |
| Stroke | Wave 3 | White | 12602238 | 16633 | 83144516 | 20 | 1.41 | (1.26, 1.58) | 0.82 | (0.73, 0.92) | 0.96 | (0.85, 1.08) |
| Stroke | Wave 3 | Asian | 1091687 | 756 | 7193014 | 10.51 | 0.84 | (0.73, 0.96) | 0.88 | (0.77, 1) | 0.92 | (0.8, 1.05) |
| Stroke | Wave 3 | Black | 377684 | 312 | 2482157 | 12.57 | Reference |  | Reference |  | Reference |  |
| Stroke | Wave 3 | Mixed | 200227 | 98 | 1314892 | 7.45 | 0.57 | (0.46, 0.72) | 0.8 | (0.63, 1) | 0.86 | (0.69, 1.08) |
| Stroke | Wave 3 | Other | 314766 | 148 | 2054547 | 7.2 | 0.57 | (0.47, 0.7) | 0.71 | (0.58, 0.86) | 0.8 | (0.65, 0.97) |
| Stroke | Easing 3 | White | 12411670 | 11376 | 55895206 | 20.35 | 1.42 | (1.24, 1.64) | 0.82 | (0.71, 0.94) | 0.94 | (0.82, 1.08) |
| Stroke | Easing 3 | Asian | 1074695 | 487 | 4837290 | 10.07 | 0.79 | (0.67, 0.93) | 0.83 | (0.71, 0.98) | 0.87 | (0.74, 1.02) |
| Stroke | Easing 3 | Black | 369667 | 210 | 1661122 | 12.64 | Reference |  | Reference |  | Reference |  |
| Stroke | Easing 3 | Mixed | 197112 | 71 | 885370 | 8.020001 | 0.61 | (0.47, 0.8) | 0.86 | (0.65, 1.12) | 0.92 | (0.7, 1.2) |
| Stroke | Easing 3 | Other | 304150 | 76 | 1360142 | 5.59 | 0.44 | (0.34, 0.57) | 0.54 | (0.42, 0.71) | 0.6 | (0.46, 0.78) |
| VTE | Pre | White | 11951881 | 28859 | 299215565 | 9.64 | 1.43 | (1.3, 1.56) | 1.1 | (1, 1.2) | 1.34 | (1.23, 1.47) |
| VTE | Pre | Asian | 947196 | 752 | 23730820 | 3.17 | 0.52 | (0.46, 0.58) | 0.53 | (0.48, 0.6) | 0.56 | (0.5, 0.63) |
| VTE | Pre | Black | 323685 | 501 | 8106277 | 6.18 | Reference |  | Reference |  | Reference |  |
| VTE | Pre | Mixed | 167862 | 195 | 4204866 | 4.64 | 0.73 | (0.62, 0.86) | 0.85 | (0.72, 1) | 0.95 | (0.8, 1.12) |
| VTE | Pre | Other | 255875 | 181 | 6409861 | 2.82 | 0.46 | (0.39, 0.54) | 0.5 | (0.42, 0.59) | 0.59 | (0.5, 0.7) |
| VTE | Pandemic | White | 13007823 | 38710 | 321532860 | 12.04 | 1.34 | (1.24, 1.44) | 1.01 | (0.94, 1.08) | 1.23 | (1.14, 1.32) |
| VTE | Pandemic | Asian | 1116586 | 939 | 27555834 | 3.41 | 0.43 | (0.39, 0.47) | 0.44 | (0.4, 0.48) | 0.46 | (0.42, 0.51) |
| VTE | Pandemic | Black | 389040 | 785 | 9532588 | 8.23 | Reference |  | Reference |  | Reference |  |
| VTE | Pandemic | Mixed | 206060 | 290 | 5033737 | 5.76 | 0.68 | (0.6, 0.78) | 0.82 | (0.71, 0.93) | 0.91 | (0.79, 1.04) |
| VTE | Pandemic | Other | 334273 | 275 | 8013201 | 3.43 | 0.42 | (0.36, 0.48) | 0.47 | (0.41, 0.54) | 0.55 | (0.48, 0.63) |
| VTE | Wave 1 | White | 13008047 | 2634 | 29410861 | 8.96 | 1.13 | (0.88, 1.44) | 0.85 | (0.66, 1.1) | 1.08 | (0.84, 1.39) |
| VTE | Wave 1 | Asian | 1116589 | 59 | 2525212 | 2.34 | 0.32 | (0.22, 0.45) | 0.32 | (0.23, 0.46) | 0.35 | (0.25, 0.5) |
| VTE | Wave 1 | Black | 389022 | 65 | 879116 | 7.39 | Reference |  | Reference |  | Reference |  |
| VTE | Wave 1 | Mixed | 206091 | 17 | 465927 | 3.65 | 0.48 | (0.28, 0.82) | 0.59 | (0.34, 1) | 0.67 | (0.39, 1.14) |
| VTE | Wave 1 | Other | 334304 | 17 | 754936 | 2.25 | 0.31 | (0.18, 0.52) | 0.35 | (0.21, 0.6) | 0.43 | (0.25, 0.73) |
| VTE | Easing 1 | White | 13059222 | 5157 | 42512892 | 12.13 | 1.52 | (1.23, 1.87) | 1.16 | (0.94, 1.43) | 1.46 | (1.18, 1.8) |
| VTE | Easing 1 | Asian | 1128788 | 116 | 3674863 | 3.16 | 0.44 | (0.34, 0.58) | 0.46 | (0.35, 0.6) | 0.49 | (0.37, 0.64) |
| VTE | Easing 1 | Black | 393361 | 93 | 1279337 | 7.27 | Reference |  | Reference |  | Reference |  |
| VTE | Easing 1 | Mixed | 208793 | 36 | 678890 | 5.3 | 0.71 | (0.48, 1.04) | 0.85 | (0.58, 1.25) | 0.96 | (0.66, 1.42) |
| VTE | Easing 1 | Other | 338288 | 39 | 1098249 | 3.55 | 0.48 | (0.33, 0.7) | 0.55 | (0.38, 0.8) | 0.67 | (0.46, 0.97) |
| VTE | Wave 2 | White | 12967150 | 12627 | 97803435 | 12.91 | 1.36 | (1.2, 1.54) | 1.02 | (0.9, 1.15) | 1.25 | (1.1, 1.42) |
| VTE | Wave 2 | Asian | 1122608 | 324 | 8460268 | 3.83 | 0.46 | (0.39, 0.54) | 0.47 | (0.4, 0.56) | 0.51 | (0.43, 0.6) |
| VTE | Wave 2 | Black | 390036 | 253 | 2934299 | 8.62 | Reference |  | Reference |  | Reference |  |
| VTE | Wave 2 | Mixed | 207205 | 101 | 1555115 | 6.49 | 0.73 | (0.58, 0.92) | 0.89 | (0.7, 1.12) | 0.99 | (0.79, 1.25) |
| VTE | Wave 2 | Other | 332902 | 80 | 2485458 | 3.22 | 0.37 | (0.29, 0.48) | 0.43 | (0.33, 0.55) | 0.51 | (0.39, 0.65) |
| VTE | Easing 2 | White | 12640457 | 1801 | 13462095 | 13.38 | 1.31 | (0.95, 1.82) | 0.98 | (0.7, 1.36) | 1.22 | (0.88, 1.7) |
| VTE | Easing 2 | Asian | 1095139 | 41 | 1166258 | 3.52 | 0.38 | (0.24, 0.59) | 0.39 | (0.25, 0.61) | 0.42 | (0.27, 0.65) |
| VTE | Easing 2 | Black | 379052 | 38 | 403537 | 9.42 | Reference |  | Reference |  | Reference |  |
| VTE | Easing 2 | Mixed | 201011 | 14 | 213929 | 6.54 | 0.68 | (0.37, 1.26) | 0.82 | (0.45, 1.52) | 0.93 | (0.51, 1.72) |
| VTE | Easing 2 | Other | 317012 | 12 | 337066 | 3.56 | 0.38 | (0.2, 0.72) | 0.43 | (0.22, 0.81) | 0.52 | (0.27, 0.99) |
| VTE | Wave 3 | White | 12602274 | 10091 | 83160465 | 12.13 | 1.36 | (1.18, 1.57) | 1.03 | (0.9, 1.19) | 1.27 | (1.1, 1.47) |
| VTE | Wave 3 | Asian | 1091690 | 249 | 7194558 | 3.46 | 0.44 | (0.37, 0.53) | 0.45 | (0.38, 0.55) | 0.48 | (0.4, 0.58) |
| VTE | Wave 3 | Black | 377684 | 202 | 2482470 | 8.14 | Reference |  | Reference |  | Reference |  |
| VTE | Wave 3 | Mixed | 200227 | 80 | 1314939 | 6.08 | 0.73 | (0.56, 0.94) | 0.86 | (0.67, 1.12) | 0.97 | (0.75, 1.25) |
| VTE | Wave 3 | Other | 314767 | 80 | 2054751 | 3.89 | 0.47 | (0.36, 0.61) | 0.53 | (0.41, 0.68) | 0.62 | (0.48, 0.81) |
| VTE | Easing 3 | White | 12411687 | 6834 | 55902234 | 12.22 | 1.36 | (1.15, 1.62) | 1.02 | (0.86, 1.22) | 1.21 | (1.02, 1.44) |
| VTE | Easing 3 | Asian | 1074698 | 184 | 4837902 | 3.8 | 0.48 | (0.39, 0.6) | 0.5 | (0.4, 0.62) | 0.52 | (0.42, 0.65) |
| VTE | Easing 3 | Black | 369670 | 136 | 1661279 | 8.19 | Reference |  | Reference |  | Reference |  |
| VTE | Easing 3 | Mixed | 197112 | 46 | 885409 | 5.2 | 0.62 | (0.44, 0.87) | 0.74 | (0.53, 1.04) | 0.81 | (0.58, 1.13) |
| VTE | Easing 3 | Other | 304148 | 41 | 1360203 | 3.01 | 0.37 | (0.26, 0.52) | 0.41 | (0.29, 0.58) | 0.47 | (0.33, 0.66) |
| Heart failure | Pre | White | 11951886 | 31037 | 299209045 | 10.37 | 1.4 | (1.29, 1.52) | 0.71 | (0.65, 0.77) | 1.01 | (0.93, 1.09) |
| Heart failure | Pre | Asian | 947193 | 1925 | 23716286 | 8.12 | 1.09 | (0.99, 1.2) | 1.11 | (1.01, 1.22) | 1.23 | (1.12, 1.35) |
| Heart failure | Pre | Black | 323687 | 597 | 8104779 | 7.37 | Reference |  | Reference |  | Reference |  |
| Heart failure | Pre | Mixed | 167861 | 166 | 4204968 | 3.95 | 0.54 | (0.46, 0.64) | 0.78 | (0.66, 0.93) | 0.91 | (0.77, 1.08) |
| Heart failure | Pre | Other | 255875 | 222 | 6409411 | 3.46 | 0.48 | (0.42, 0.57) | 0.59 | (0.51, 0.69) | 0.77 | (0.66, 0.9) |
| Heart failure | Pandemic | White | 13007814 | 50729 | 321522961 | 15.78 | 1.72 | (1.61, 1.85) | 0.86 | (0.8, 0.92) | 1.19 | (1.11, 1.27) |
| Heart failure | Pandemic | Asian | 1116584 | 2508 | 27542692 | 9.11 | 1.02 | (0.95, 1.11) | 1.06 | (0.98, 1.15) | 1.17 | (1.08, 1.27) |
| Heart failure | Pandemic | Black | 389038 | 842 | 9532933 | 8.83 | Reference |  | Reference |  | Reference |  |
| Heart failure | Pandemic | Mixed | 206060 | 284 | 5034330 | 5.64 | 0.64 | (0.56, 0.73) | 0.97 | (0.85, 1.11) | 1.11 | (0.97, 1.27) |
| Heart failure | Pandemic | Other | 334275 | 362 | 8013004 | 4.52 | 0.53 | (0.46, 0.59) | 0.69 | (0.61, 0.78) | 0.88 | (0.77, 0.99) |
| Heart failure | Wave 1 | White | 13008038 | 3540 | 29410481 | 12.04 | 1.53 | (1.19, 1.96) | 0.76 | (0.59, 0.98) | 1.07 | (0.83, 1.37) |
| Heart failure | Wave 1 | Asian | 1116587 | 185 | 2525095 | 7.33 | 0.97 | (0.73, 1.29) | 1.01 | (0.76, 1.34) | 1.11 | (0.83, 1.47) |
| Heart failure | Wave 1 | Black | 389020 | 65 | 879134 | 7.39 | Reference |  | Reference |  | Reference |  |
| Heart failure | Wave 1 | Mixed | 206091 | 17 | 465929 | 3.65 | 0.48 | (0.28, 0.82) | 0.75 | (0.44, 1.28) | 0.86 | (0.51, 1.47) |
| Heart failure | Wave 1 | Other | 334306 | 27 | 754939 | 3.58 | 0.47 | (0.3, 0.74) | 0.64 | (0.4, 1.01) | 0.81 | (0.51, 1.28) |
| Heart failure | Easing 1 | White | 13059194 | 6970 | 42510238 | 16.4 | 1.65 | (1.37, 1.97) | 0.81 | (0.68, 0.98) | 1.16 | (0.96, 1.39) |
| Heart failure | Easing 1 | Asian | 1128784 | 376 | 3674476 | 10.23 | 1.09 | (0.89, 1.34) | 1.13 | (0.92, 1.39) | 1.25 | (1.02, 1.54) |
| Heart failure | Easing 1 | Black | 393360 | 121 | 1279279 | 9.46 | Reference |  | Reference |  | Reference |  |
| Heart failure | Easing 1 | Mixed | 208793 | 38 | 678881 | 5.6 | 0.59 | (0.41, 0.85) | 0.9 | (0.63, 1.3) | 1.05 | (0.73, 1.51) |
| Heart failure | Easing 1 | Other | 338286 | 37 | 1098249 | 3.37 | 0.36 | (0.25, 0.52) | 0.48 | (0.33, 0.7) | 0.62 | (0.43, 0.91) |
| Heart failure | Wave 2 | White | 12967138 | 16378 | 97799415 | 16.75 | 1.52 | (1.36, 1.7) | 0.76 | (0.67, 0.85) | 1.06 | (0.94, 1.19) |
| Heart failure | Wave 2 | Asian | 1122605 | 844 | 8458766 | 9.98 | 0.93 | (0.82, 1.06) | 0.97 | (0.85, 1.1) | 1.06 | (0.93, 1.21) |
| Heart failure | Wave 2 | Black | 390036 | 311 | 2934208 | 10.6 | Reference |  | Reference |  | Reference |  |
| Heart failure | Wave 2 | Mixed | 207204 | 89 | 1555247 | 5.72 | 0.54 | (0.42, 0.68) | 0.82 | (0.65, 1.04) | 0.95 | (0.75, 1.2) |
| Heart failure | Wave 2 | Other | 332902 | 131 | 2485311 | 5.27 | 0.51 | (0.41, 0.62) | 0.68 | (0.56, 0.84) | 0.87 | (0.71, 1.06) |
| Heart failure | Easing 2 | White | 12640422 | 3464 | 13461250 | 25.73 | 1.92 | (1.46, 2.53) | 0.93 | (0.7, 1.23) | 1.34 | (1.01, 1.77) |
| Heart failure | Easing 2 | Asian | 1095135 | 199 | 1166170 | 17.06 | 1.32 | (0.97, 1.8) | 1.38 | (1.02, 1.88) | 1.54 | (1.14, 2.1) |
| Heart failure | Easing 2 | Black | 379052 | 52 | 403527 | 12.89 | Reference |  | Reference |  | Reference |  |
| Heart failure | Easing 2 | Mixed | 201011 | 19 | 213926 | 8.88 | 0.69 | (0.41, 1.16) | 1.06 | (0.63, 1.79) | 1.24 | (0.73, 2.1) |
| Heart failure | Easing 2 | Other | 317012 | 30 | 337056 | 8.9 | 0.71 | (0.45, 1.11) | 0.95 | (0.61, 1.49) | 1.23 | (0.78, 1.92) |
| Heart failure | Wave 3 | White | 12602204 | 14580 | 83149534 | 17.53 | 1.82 | (1.6, 2.07) | 0.93 | (0.81, 1.06) | 1.31 | (1.15, 1.49) |
| Heart failure | Wave 3 | Asian | 1091682 | 767 | 7192954 | 10.66 | 1.1 | (0.95, 1.27) | 1.16 | (1, 1.34) | 1.29 | (1.11, 1.49) |
| Heart failure | Wave 3 | Black | 377683 | 239 | 2482401 | 9.63 | Reference |  | Reference |  | Reference |  |
| Heart failure | Wave 3 | Mixed | 200224 | 87 | 1314956 | 6.62 | 0.69 | (0.54, 0.88) | 1.06 | (0.83, 1.35) | 1.23 | (0.96, 1.57) |
| Heart failure | Wave 3 | Other | 314766 | 119 | 2054623 | 5.79 | 0.62 | (0.5, 0.77) | 0.82 | (0.66, 1.02) | 1.05 | (0.84, 1.31) |
| Heart failure | Easing 3 | White | 12411683 | 11000 | 55896009 | 19.68 | 1.72 | (1.48, 1.99) | 0.86 | (0.75, 1) | 1.19 | (1.02, 1.38) |
| Heart failure | Easing 3 | Asian | 1074692 | 571 | 4837135 | 11.8 | 1.04 | (0.88, 1.23) | 1.1 | (0.93, 1.3) | 1.21 | (1.03, 1.43) |
| Heart failure | Easing 3 | Black | 369669 | 187 | 1661225 | 11.26 | Reference |  | Reference |  | Reference |  |
| Heart failure | Easing 3 | Mixed | 197111 | 65 | 885385 | 7.34 | 0.65 | (0.49, 0.87) | 1 | (0.75, 1.33) | 1.15 | (0.87, 1.53) |
| Heart failure | Easing 3 | Other | 304148 | 69 | 1360166 | 5.07 | 0.47 | (0.35, 0.61) | 0.61 | (0.46, 0.8) | 0.76 | (0.57, 1) |
| MI | Pre | White | 11951868 | 41681 | 299044045 | 13.94 | 2.39 | (2.17, 2.64) | 1.59 | (1.44, 1.75) | 1.93 | (1.75, 2.12) |
| MI | Pre | Asian | 947193 | 3206 | 23700749 | 13.53 | 2.53 | (2.28, 2.8) | 2.67 | (2.41, 2.95) | 2.82 | (2.55, 3.12) |
| MI | Pre | Black | 323687 | 425 | 8107170 | 5.24 | Reference |  | Reference |  | Reference |  |
| MI | Pre | Mixed | 167862 | 212 | 4204384 | 5.04 | 0.94 | (0.8, 1.11) | 1.27 | (1.08, 1.5) | 1.39 | (1.18, 1.64) |
| MI | Pre | Other | 255872 | 329 | 6407889 | 5.13 | 0.99 | (0.86, 1.14) | 1.19 | (1.03, 1.37) | 1.37 | (1.19, 1.59) |
| MI | Pandemic | White | 13007792 | 49713 | 321413154 | 15.47 | 2.17 | (2.01, 2.36) | 1.43 | (1.32, 1.55) | 1.7 | (1.57, 1.85) |
| MI | Pandemic | Asian | 1116586 | 3864 | 27525109 | 14.04 | 2.16 | (1.98, 2.35) | 2.26 | (2.08, 2.47) | 2.37 | (2.18, 2.59) |
| MI | Pandemic | Black | 389038 | 603 | 9534880 | 6.32 | Reference |  | Reference |  | Reference |  |
| MI | Pandemic | Mixed | 206059 | 252 | 5033918 | 5.01 | 0.77 | (0.66, 0.89) | 1.05 | (0.91, 1.22) | 1.14 | (0.99, 1.32) |
| MI | Pandemic | Other | 334275 | 480 | 8011100 | 5.99 | 0.95 | (0.84, 1.07) | 1.19 | (1.05, 1.34) | 1.34 | (1.19, 1.51) |
| MI | Wave 1 | White | 13008016 | 3585 | 29409854 | 12.19 | 2.86 | (2.03, 4.04) | 1.92 | (1.36, 2.71) | 2.32 | (1.64, 3.29) |
| MI | Wave 1 | Asian | 1116589 | 271 | 2525011 | 10.73 | 2.75 | (1.91, 3.94) | 2.9 | (2.02, 4.16) | 3.05 | (2.12, 4.38) |
| MI | Wave 1 | Black | 389020 | 33 | 879155 | 3.75 | Reference |  | Reference |  | Reference |  |
| MI | Wave 1 | Mixed | 206090 | 17 | 465920 | 3.65 | 0.93 | (0.52, 1.68) | 1.3 | (0.72, 2.33) | 1.42 | (0.79, 2.55) |
| MI | Wave 1 | Other | 334306 | 21 | 754936 | 2.78 | 0.7 | (0.4, 1.22) | 0.9 | (0.52, 1.57) | 1.02 | (0.59, 1.78) |
| MI | Easing 1 | White | 13059185 | 6342 | 42511189 | 14.92 | 2.31 | (1.82, 2.92) | 1.48 | (1.17, 1.88) | 1.8 | (1.42, 2.28) |
| MI | Easing 1 | Asian | 1128784 | 459 | 3674351 | 12.49 | 2.2 | (1.71, 2.82) | 2.3 | (1.79, 2.95) | 2.42 | (1.89, 3.11) |
| MI | Easing 1 | Black | 393362 | 71 | 1279392 | 5.55 | Reference |  | Reference |  | Reference |  |
| MI | Easing 1 | Mixed | 208793 | 31 | 678906 | 4.57 | 0.79 | (0.52, 1.2) | 1.09 | (0.72, 1.67) | 1.2 | (0.78, 1.83) |
| MI | Easing 1 | Other | 338288 | 66 | 1098217 | 6.01 | 1.08 | (0.77, 1.51) | 1.37 | (0.98, 1.91) | 1.56 | (1.12, 2.19) |
| MI | Wave 2 | White | 12967152 | 15520 | 97794466 | 15.87 | 2.22 | (1.91, 2.57) | 1.43 | (1.24, 1.66) | 1.7 | (1.46, 1.97) |
| MI | Wave 2 | Asian | 1122602 | 1172 | 8457313 | 13.86 | 2.16 | (1.85, 2.53) | 2.26 | (1.93, 2.64) | 2.36 | (2.02, 2.76) |
| MI | Wave 2 | Black | 390035 | 182 | 2934586 | 6.2 | Reference |  | Reference |  | Reference |  |
| MI | Wave 2 | Mixed | 207205 | 81 | 1555226 | 5.21 | 0.81 | (0.62, 1.05) | 1.11 | (0.85, 1.44) | 1.21 | (0.93, 1.57) |
| MI | Wave 2 | Other | 332901 | 152 | 2485197 | 6.12 | 0.99 | (0.79, 1.22) | 1.24 | (1, 1.53) | 1.39 | (1.12, 1.73) |
| MI | Easing 2 | White | 12640414 | 2489 | 13461750 | 18.49 | 1.67 | (1.21, 2.3) | 1.06 | (0.77, 1.47) | 1.24 | (0.9, 1.71) |
| MI | Easing 2 | Asian | 1095133 | 172 | 1166183 | 14.75 | 1.47 | (1.03, 2.08) | 1.53 | (1.08, 2.17) | 1.6 | (1.13, 2.26) |
| MI | Easing 2 | Black | 379051 | 39 | 403537 | 9.66 | Reference |  | Reference |  | Reference |  |
| MI | Easing 2 | Mixed | redacted |  |  |  |  |  |  |  |  |  |
| MI | Easing 2 | Other | 317013 | 19 | 337062 | 5.64 | 0.58 | (0.33, 1) | 0.71 | (0.41, 1.24) | 0.8 | (0.46, 1.38) |
| MI | Wave 3 | White | 12602261 | 13201 | 83150575 | 15.88 | 2.13 | (1.83, 2.48) | 1.39 | (1.19, 1.62) | 1.66 | (1.42, 1.94) |
| MI | Wave 3 | Asian | 1091685 | 1085 | 7191830 | 15.09 | 2.16 | (1.84, 2.55) | 2.27 | (1.93, 2.68) | 2.38 | (2.03, 2.8) |
| MI | Wave 3 | Black | 377686 | 169 | 2482621 | 6.81 | Reference |  | Reference |  | Reference |  |
| MI | Wave 3 | Mixed | 200225 | 60 | 1314987 | 4.56 | 0.66 | (0.49, 0.88) | 0.9 | (0.67, 1.21) | 0.98 | (0.73, 1.32) |
| MI | Wave 3 | Other | 314768 | 133 | 2054569 | 6.47 | 0.97 | (0.78, 1.22) | 1.2 | (0.96, 1.51) | 1.35 | (1.08, 1.7) |
| MI | Easing 3 | White | 12411685 | 9252 | 55898155 | 16.55 | 2.2 | (1.82, 2.64) | 1.44 | (1.19, 1.73) | 1.72 | (1.42, 2.07) |
| MI | Easing 3 | Asian | 1074694 | 783 | 4836634 | 16.19 | 2.26 | (1.85, 2.74) | 2.37 | (1.95, 2.88) | 2.49 | (2.05, 3.03) |
| MI | Easing 3 | Black | 369668 | 115 | 1661334 | 6.92 | Reference |  | Reference |  | Reference |  |
| MI | Easing 3 | Mixed | 197112 | 53 | 885392 | 5.99 | 0.85 | (0.62, 1.18) | 1.17 | (0.85, 1.62) | 1.27 | (0.92, 1.76) |
| MI | Easing 3 | Other | 304150 | 98 | 1360084 | 7.21 | 1.05 | (0.8, 1.38) | 1.29 | (0.98, 1.69) | 1.45 | (1.11, 1.9) |
| DKA | Pre | White | 841518 | 4898 | 21022238 | 23.3 | 1.5 | (1.25, 1.81) | 2 | (1.67, 2.41) | 2.24 | (1.86, 2.7) |
| DKA | Pre | Asian | 115505 | 188 | 2892286 | 6.5 | 0.4 | (0.32, 0.5) | 0.38 | (0.3, 0.47) | 0.39 | (0.31, 0.49) |
| DKA | Pre | Black | 28706 | 121 | 717714 | 16.86 | Reference |  | Reference |  | Reference |  |
| DKA | Pre | Mixed | 9109 | 53 | 227450 | 23.3 | 1.44 | (1.04, 1.99) | 1.2 | (0.87, 1.66) | 1.25 | (0.91, 1.73) |
| DKA | Pre | Other | 12222 | 34 | 305866 | 11.12 | 0.67 | (0.46, 0.99) | 0.72 | (0.49, 1.06) | 0.76 | (0.52, 1.12) |
| DKA | Pandemic | White | 995361 | 5666 | 24030509 | 23.58 | 1.04 | (0.9, 1.19) | 1.33 | (1.15, 1.53) | 1.5 | (1.3, 1.73) |
| DKA | Pandemic | Asian | 142353 | 244 | 3500862 | 6.97 | 0.3 | (0.25, 0.36) | 0.28 | (0.24, 0.34) | 0.29 | (0.24, 0.35) |
| DKA | Pandemic | Black | 36530 | 214 | 887198 | 24.12 | Reference |  | Reference |  | Reference |  |
| DKA | Pandemic | Mixed | 11738 | 65 | 286457 | 22.69 | 0.96 | (0.73, 1.27) | 0.81 | (0.61, 1.07) | 0.85 | (0.64, 1.12) |
| DKA | Pandemic | Other | 16225 | 43 | 394708 | 10.89 | 0.45 | (0.33, 0.63) | 0.47 | (0.34, 0.65) | 0.5 | (0.36, 0.69) |
| DKA | Wave 1 | White | 995392 | 588 | 2242264 | 26.22 | 1.02 | (0.65, 1.6) | 1.36 | (0.86, 2.12) | 1.63 | (1.04, 2.57) |
| DKA | Wave 1 | Asian | 142342 | 23 | 321261 | 7.16 | 0.28 | (0.15, 0.51) | 0.27 | (0.15, 0.48) | 0.28 | (0.16, 0.51) |
| DKA | Wave 1 | Black | 36526 | 21 | 82234 | 25.54 | Reference |  | Reference |  | Reference |  |
| DKA | Wave 1 | Mixed | redacted |  |  |  |  |  |  |  |  |  |
| DKA | Wave 1 | Other | redacted |  |  |  |  |  |  |  |  |  |
| DKA | Easing 1 | White | 992829 | 891 | 3223673 | 27.64 | 0.99 | (0.7, 1.39) | 1.28 | (0.91, 1.8) | 1.48 | (1.05, 2.08) |
| DKA | Easing 1 | Asian | 142905 | 31 | 465180 | 6.66 | 0.22 | (0.14, 0.36) | 0.21 | (0.13, 0.34) | 0.22 | (0.14, 0.35) |
| DKA | Easing 1 | Black | 36652 | 37 | 119132 | 31.06 | Reference |  | Reference |  | Reference |  |
| DKA | Easing 1 | Mixed | redacted |  |  |  |  |  |  |  |  |  |
| DKA | Easing 1 | Other | redacted |  |  |  |  |  |  |  |  |  |
| DKA | Wave 2 | White | 993240 | 2005 | 7442036 | 26.94 | 1.15 | (0.89, 1.48) | 1.5 | (1.16, 1.94) | 1.74 | (1.35, 2.25) |
| DKA | Wave 2 | Asian | 143331 | 89 | 1079487 | 8.24 | 0.35 | (0.25, 0.48) | 0.34 | (0.24, 0.47) | 0.35 | (0.26, 0.48) |
| DKA | Wave 2 | Black | 36803 | 65 | 276312 | 23.52 | Reference |  | Reference |  | Reference |  |
| DKA | Wave 2 | Mixed | 11829 | 33 | 88847 | 37.14 | 1.57 | (1.04, 2.4) | 1.3 | (0.85, 1.98) | 1.38 | (0.9, 2.09) |
| DKA | Wave 2 | Other | 16467 | 13 | 123610 | 10.52 | 0.44 | (0.24, 0.8) | 0.45 | (0.25, 0.83) | 0.49 | (0.27, 0.89) |
| DKA | Easing 2 | White | 997807 | 363 | 1062042 | 34.18 | 1.27 | (0.72, 2.26) | 1.61 | (0.91, 2.85) | 1.88 | (1.06, 3.33) |
| DKA | Easing 2 | Asian | 144411 | 17 | 153794 | 11.05 | 0.35 | (0.17, 0.72) | 0.33 | (0.16, 0.69) | 0.35 | (0.17, 0.71) |
| DKA | Easing 2 | Black | 37160 | 13 | 39575 | 32.85 | Reference |  | Reference |  | Reference |  |
| DKA | Easing 2 | Mixed | redacted |  |  |  |  |  |  |  |  |  |
| DKA | Easing 2 | Other | redacted |  |  |  |  |  |  |  |  |  |
| DKA | Wave 3 | White | 999832 | 1974 | 6568038 | 30.05 | 1.09 | (0.85, 1.4) | 1.41 | (1.1, 1.8) | 1.59 | (1.24, 2.04) |
| DKA | Wave 3 | Asian | 144709 | 84 | 955023 | 8.8 | 0.32 | (0.23, 0.44) | 0.31 | (0.22, 0.42) | 0.32 | (0.23, 0.43) |
| DKA | Wave 3 | Black | 37309 | 70 | 245487 | 28.51 | Reference |  | Reference |  | Reference |  |
| DKA | Wave 3 | Mixed | 12024 | 25 | 79183 | 31.57 | 1.13 | (0.72, 1.78) | 0.92 | (0.58, 1.45) | 0.96 | (0.61, 1.52) |
| DKA | Wave 3 | Other | 16735 | 18 | 110066 | 16.35 | 0.59 | (0.35, 0.99) | 0.6 | (0.36, 1) | 0.64 | (0.38, 1.07) |
| DKA | Easing 3 | White | 1004850 | 1402 | 4506363 | 31.11 | 1.05 | (0.79, 1.4) | 1.35 | (1.02, 1.78) | 1.49 | (1.12, 1.97) |
| DKA | Easing 3 | Asian | 146317 | 65 | 658544 | 9.87 | 0.32 | (0.22, 0.45) | 0.3 | (0.21, 0.44) | 0.31 | (0.22, 0.45) |
| DKA | Easing 3 | Black | 37795 | 55 | 169620 | 32.43 | Reference |  | Reference |  | Reference |  |
| DKA | Easing 3 | Mixed | 12233 | 17 | 54888 | 30.97 | 0.98 | (0.57, 1.7) | 0.81 | (0.47, 1.4) | 0.85 | (0.49, 1.46) |
| DKA | Easing 3 | Other | 16941 | 13 | 76042 | 17.1 | 0.53 | (0.29, 0.97) | 0.54 | (0.29, 0.99) | 0.57 | (0.31, 1.04) |
| T1 DM | Pre | White | 87911 | 5492 | 2126366 | 258.28 | 1.69 | (1.38, 2.06) | 1.6 | (1.32, 1.95) | 1.98 | (1.62, 2.42) |
| T1 DM | Pre | Asian | 5914 | 141 | 146226 | 96.43 | 0.56 | (0.43, 0.72) | 0.58 | (0.45, 0.75) | 0.62 | (0.48, 0.8) |
| T1 DM | Pre | Black | 2450 | 105 | 59963 | 175.11 | Reference |  | Reference |  | Reference |  |
| T1 DM | Pre | Mixed | 956 | 70 | 22919 | 305.42 | 1.84 | (1.36, 2.5) | 1.49 | (1.1, 2.02) | 1.63 | (1.2, 2.2) |
| T1 DM | Pre | Other | 949 | 40 | 23225 | 172.23 | 1 | (0.69, 1.43) | 0.92 | (0.64, 1.32) | 1.03 | (0.72, 1.48) |
| T1 DM | Pandemic | White | 100454 | 5530 | 2385604 | 231.81 | 1.47 | (1.23, 1.77) | 1.42 | (1.18, 1.7) | 1.8 | (1.5, 2.17) |
| T1 DM | Pandemic | Asian | 7274 | 143 | 175440 | 81.51 | 0.46 | (0.36, 0.59) | 0.49 | (0.38, 0.62) | 0.52 | (0.41, 0.66) |
| T1 DM | Pandemic | Black | 3004 | 125 | 70660 | 176.9 | Reference |  | Reference |  | Reference |  |
| T1 DM | Pandemic | Mixed | 1180 | 63 | 27871 | 226.04 | 1.35 | (1, 1.83) | 1.12 | (0.83, 1.52) | 1.23 | (0.91, 1.67) |
| T1 DM | Pandemic | Other | 1255 | 52 | 29573 | 175.84 | 1 | (0.73, 1.39) | 0.93 | (0.67, 1.28) | 1.05 | (0.76, 1.46) |
| T1 DM | Wave 1 | White | 100463 | 616 | 226053 | 272.5 | 1.56 | (0.85, 2.87) | 1.51 | (0.82, 2.76) | 2.06 | (1.12, 3.8) |
| T1 DM | Wave 1 | Asian | 7275 | 13 | 16397 | 79.28 | 0.48 | (0.22, 1.08) | 0.51 | (0.23, 1.14) | 0.55 | (0.25, 1.23) |
| T1 DM | Wave 1 | Black | 3001 | 11 | 6726 | 163.55 | Reference |  | Reference |  | Reference |  |
| T1 DM | Wave 1 | Mixed | redacted |  |  |  |  |  |  |  |  |  |
| T1 DM | Wave 1 | Other | redacted |  |  |  |  |  |  |  |  |  |
| T1 DM | Easing 1 | White | 100755 | 1042 | 325820 | 319.81 | 1.25 | (0.85, 1.83) | 1.2 | (0.82, 1.75) | 1.52 | (1.04, 2.23) |
| T1 DM | Easing 1 | Asian | 7311 | 23 | 23742 | 96.87 | 0.33 | (0.19, 0.57) | 0.35 | (0.2, 0.61) | 0.38 | (0.22, 0.65) |
| T1 DM | Easing 1 | Black | 3027 | 29 | 9786 | 296.35 | Reference |  | Reference |  | Reference |  |
| T1 DM | Easing 1 | Mixed | 1194 | 14 | 3863 | 362.4 | 1.29 | (0.68, 2.45) | 1.05 | (0.56, 2) | 1.16 | (0.61, 2.2) |
| T1 DM | Easing 1 | Other | redacted |  |  |  |  |  |  |  |  |  |
| T1 DM | Wave 2 | White | 100749 | 2105 | 749144 | 280.99 | 1.53 | (1.14, 2.07) | 1.49 | (1.1, 2) | 1.97 | (1.46, 2.66) |
| T1 DM | Wave 2 | Asian | 7314 | 50 | 54660 | 91.47 | 0.45 | (0.3, 0.67) | 0.48 | (0.32, 0.72) | 0.52 | (0.35, 0.78) |
| T1 DM | Wave 2 | Black | 3052 | 46 | 22633 | 203.24 | Reference |  | Reference |  | Reference |  |
| T1 DM | Wave 2 | Mixed | 1187 | 30 | 8769 | 342.1 | 1.78 | (1.12, 2.82) | 1.46 | (0.92, 2.32) | 1.63 | (1.03, 2.59) |
| T1 DM | Wave 2 | Other | 1263 | 15 | 9374 | 160.02 | 0.8 | (0.45, 1.43) | 0.74 | (0.41, 1.33) | 0.87 | (0.48, 1.55) |
| T1 DM | Easing 2 | White | 100533 | 408 | 106809 | 381.99 | 2.01 | (0.94, 4.32) | 1.93 | (0.9, 4.14) | 2.52 | (1.17, 5.41) |
| T1 DM | Easing 2 | Asian | 7334 | 14 | 7801 | 179.46 | 0.86 | (0.35, 2.13) | 0.91 | (0.37, 2.26) | 0.98 | (0.39, 2.43) |
| T1 DM | Easing 2 | Black | redacted |  |  |  |  |  |  |  |  |  |
| T1 DM | Easing 2 | Mixed | redacted |  |  |  |  |  |  |  |  |  |
| T1 DM | Easing 2 | Other | redacted |  |  |  |  |  |  |  |  |  |
| T1 DM | Wave 3 | White | 100692 | 1975 | 656541 | 300.82 | 1.02 | (0.79, 1.32) | 0.99 | (0.76, 1.28) | 1.23 | (0.95, 1.6) |
| T1 DM | Wave 3 | Asian | 7359 | 58 | 48340 | 119.98 | 0.38 | (0.27, 0.55) | 0.41 | (0.28, 0.58) | 0.43 | (0.3, 0.62) |
| T1 DM | Wave 3 | Black | 3075 | 63 | 19944 | 315.88 | Reference |  | Reference |  | Reference |  |
| T1 DM | Wave 3 | Mixed | 1172 | 27 | 7621 | 354.27 | 1.17 | (0.74, 1.83) | 0.93 | (0.59, 1.46) | 1.02 | (0.65, 1.61) |
| T1 DM | Wave 3 | Other | 1257 | 17 | 8156 | 208.44 | 0.67 | (0.39, 1.14) | 0.61 | (0.36, 1.04) | 0.68 | (0.4, 1.17) |
| T1 DM | Easing 3 | White | 100982 | 1461 | 450456 | 324.34 | 1.54 | (1.08, 2.2) | 1.51 | (1.06, 2.15) | 1.92 | (1.35, 2.74) |
| T1 DM | Easing 3 | Asian | 7439 | 46 | 33317 | 138.07 | 0.59 | (0.37, 0.92) | 0.63 | (0.4, 0.98) | 0.67 | (0.43, 1.04) |
| T1 DM | Easing 3 | Black | 3126 | 33 | 13941 | 236.72 | Reference |  | Reference |  | Reference |  |
| T1 DM | Easing 3 | Mixed | 1196 | 18 | 5313 | 338.81 | 1.51 | (0.85, 2.69) | 1.21 | (0.68, 2.15) | 1.35 | (0.76, 2.4) |
| T1 DM | Easing 3 | Other | 1243 | 16 | 5550 | 288.3 | 1.24 | (0.68, 2.26) | 1.14 | (0.63, 2.07) | 1.28 | (0.7, 2.32) |
| T2 DM | Pre | White | 791860 | 8195 | 19744444 | 41.51 | 0.91 | (0.81, 1.02) | 0.94 | (0.84, 1.05) | 1.18 | (1.06, 1.32) |
| T2 DM | Pre | Asian | 114073 | 783 | 2849512 | 27.48 | 0.57 | (0.5, 0.65) | 0.57 | (0.51, 0.65) | 0.64 | (0.57, 0.73) |
| T2 DM | Pre | Black | 27906 | 348 | 695157 | 50.06 | Reference |  | Reference |  | Reference |  |
| T2 DM | Pre | Mixed | 8600 | 76 | 214680 | 35.4 | 0.72 | (0.56, 0.93) | 0.73 | (0.57, 0.93) | 0.79 | (0.61, 1.01) |
| T2 DM | Pre | Other | 11805 | 87 | 294856 | 29.51 | 0.61 | (0.48, 0.77) | 0.62 | (0.49, 0.78) | 0.7 | (0.55, 0.89) |
| T2 DM | Pandemic | White | 941455 | 11040 | 22648478 | 48.74 | 0.93 | (0.84, 1.02) | 0.93 | (0.84, 1.02) | 1.16 | (1.06, 1.28) |
| T2 DM | Pandemic | Asian | 140656 | 892 | 3452607 | 25.84 | 0.48 | (0.43, 0.54) | 0.48 | (0.43, 0.54) | 0.54 | (0.49, 0.61) |
| T2 DM | Pandemic | Black | 35567 | 482 | 861384 | 55.96 | Reference |  | Reference |  | Reference |  |
| T2 DM | Pandemic | Mixed | 11131 | 124 | 271208 | 45.72 | 0.83 | (0.68, 1.01) | 0.84 | (0.69, 1.02) | 0.91 | (0.75, 1.11) |
| T2 DM | Pandemic | Other | 15672 | 122 | 380633 | 32.05 | 0.58 | (0.47, 0.71) | 0.58 | (0.48, 0.71) | 0.66 | (0.54, 0.8) |
| T2 DM | Wave 1 | White | 941483 | 870 | 2120264 | 41.03 | 0.83 | (0.6, 1.16) | 0.86 | (0.61, 1.19) | 1.14 | (0.82, 1.6) |
| T2 DM | Wave 1 | Asian | 140644 | 57 | 317405 | 17.96 | 0.38 | (0.25, 0.56) | 0.38 | (0.25, 0.57) | 0.44 | (0.29, 0.66) |
| T2 DM | Wave 1 | Black | 35564 | 40 | 80057 | 49.96 | Reference |  | Reference |  | Reference |  |
| T2 DM | Wave 1 | Mixed | redacted |  |  |  |  |  |  |  |  |  |
| T2 DM | Wave 1 | Other | 15687 | 11 | 35357 | 31.11 | 0.62 | (0.32, 1.2) | 0.62 | (0.32, 1.22) | 0.72 | (0.37, 1.41) |
| T2 DM | Easing 1 | White | 938717 | 1720 | 3046173 | 56.46 | 0.92 | (0.72, 1.17) | 0.91 | (0.72, 1.15) | 1.16 | (0.91, 1.48) |
| T2 DM | Easing 1 | Asian | 141193 | 122 | 459473 | 26.55 | 0.42 | (0.32, 0.57) | 0.43 | (0.32, 0.57) | 0.48 | (0.36, 0.65) |
| T2 DM | Easing 1 | Black | 35668 | 77 | 115876 | 66.45 | Reference |  | Reference |  | Reference |  |
| T2 DM | Easing 1 | Mixed | 11151 | 13 | 36284 | 35.83 | 0.55 | (0.31, 0.99) | 0.56 | (0.31, 1.01) | 0.61 | (0.34, 1.09) |
| T2 DM | Easing 1 | Other | 15777 | 20 | 51264 | 39.01 | 0.6 | (0.37, 0.99) | 0.6 | (0.37, 0.99) | 0.69 | (0.42, 1.13) |
| T2 DM | Wave 2 | White | 939511 | 3523 | 7032361 | 50.1 | 0.9 | (0.76, 1.06) | 0.92 | (0.78, 1.08) | 1.17 | (0.99, 1.39) |
| T2 DM | Wave 2 | Asian | 141615 | 291 | 1065878 | 27.3 | 0.48 | (0.39, 0.58) | 0.48 | (0.39, 0.58) | 0.54 | (0.44, 0.65) |
| T2 DM | Wave 2 | Black | 35811 | 160 | 268616 | 59.56 | Reference |  | Reference |  | Reference |  |
| T2 DM | Wave 2 | Mixed | 11220 | 46 | 84263 | 54.59 | 0.93 | (0.67, 1.29) | 0.94 | (0.68, 1.3) | 1.02 | (0.74, 1.42) |
| T2 DM | Wave 2 | Other | 15915 | 43 | 119405 | 36.01 | 0.6 | (0.43, 0.84) | 0.61 | (0.43, 0.85) | 0.69 | (0.49, 0.97) |
| T2 DM | Easing 2 | White | 945374 | 624 | 1006055 | 62.02 | 0.83 | (0.57, 1.21) | 0.82 | (0.56, 1.2) | 1.06 | (0.72, 1.55) |
| T2 DM | Easing 2 | Asian | 142718 | 61 | 151971 | 40.14 | 0.52 | (0.34, 0.81) | 0.53 | (0.34, 0.82) | 0.6 | (0.39, 0.94) |
| T2 DM | Easing 2 | Black | 36188 | 31 | 38532 | 80.45 | Reference |  | Reference |  | Reference |  |
| T2 DM | Easing 2 | Mixed | redacted |  |  |  |  |  |  |  |  |  |
| T2 DM | Easing 2 | Other | redacted |  |  |  |  |  |  |  |  |  |
| T2 DM | Wave 3 | White | 947473 | 3295 | 6218927 | 52.98 | 1.07 | (0.89, 1.29) | 1.06 | (0.88, 1.28) | 1.34 | (1.11, 1.61) |
| T2 DM | Wave 3 | Asian | 143015 | 289 | 943099 | 30.64 | 0.61 | (0.49, 0.75) | 0.61 | (0.49, 0.75) | 0.68 | (0.55, 0.84) |
| T2 DM | Wave 3 | Black | 36330 | 127 | 238909 | 53.16 | Reference |  | Reference |  | Reference |  |
| T2 DM | Wave 3 | Mixed | 11430 | 44 | 75226 | 58.49 | 1.12 | (0.79, 1.58) | 1.13 | (0.8, 1.59) | 1.22 | (0.87, 1.73) |
| T2 DM | Wave 3 | Other | 16203 | 39 | 106510 | 36.62 | 0.7 | (0.49, 1) | 0.7 | (0.49, 1) | 0.79 | (0.55, 1.14) |
| T2 DM | Easing 3 | White | 953549 | 2229 | 4273597 | 52.16 | 0.83 | (0.68, 1.02) | 0.83 | (0.68, 1.01) | 1.01 | (0.82, 1.23) |
| T2 DM | Easing 3 | Asian | 144636 | 182 | 650752 | 27.97 | 0.42 | (0.33, 0.53) | 0.42 | (0.33, 0.53) | 0.47 | (0.37, 0.59) |
| T2 DM | Easing 3 | Black | 36828 | 113 | 165179 | 68.41 | Reference |  | Reference |  | Reference |  |
| T2 DM | Easing 3 | Mixed | 11643 | 32 | 52210 | 61.29 | 0.93 | (0.62, 1.37) | 0.93 | (0.63, 1.38) | 1 | (0.67, 1.48) |
| T2 DM | Easing 3 | Other | 16434 | 13 | 73768 | 17.62 | 0.26 | (0.15, 0.46) | 0.26 | (0.15, 0.46) | 0.29 | (0.16, 0.52) |
| Anxiety | Pre | White | 11951915 | 4087 | 299505903 | 1.36 | 1.84 | (1.43, 2.38) | 1.75 | (1.35, 2.26) | 2.15 | (1.66, 2.78) |
| Anxiety | Pre | Asian | 947198 | 279 | 23736235 | 1.18 | 1.63 | (1.23, 2.16) | 1.64 | (1.24, 2.17) | 1.73 | (1.31, 2.28) |
| Anxiety | Pre | Black | 323687 | 60 | 8111470 | 0.74 | Reference |  | Reference |  | Reference |  |
| Anxiety | Pre | Mixed | 167862 | 42 | 4206466 | 1 | 1.37 | (0.92, 2.03) | 1.37 | (0.92, 2.04) | 1.53 | (1.03, 2.26) |
| Anxiety | Pre | Other | 255875 | 53 | 6411343 | 0.83 | 1.21 | (0.84, 1.75) | 1.22 | (0.85, 1.77) | 1.43 | (0.99, 2.07) |
| Anxiety | Pandemic | White | 13007851 | 4604 | 321888666 | 1.43 | 1.63 | (1.3, 2.03) | 1.57 | (1.26, 1.96) | 1.89 | (1.51, 2.36) |
| Anxiety | Pandemic | Asian | 1116587 | 316 | 27562426 | 1.15 | 1.41 | (1.1, 1.8) | 1.41 | (1.1, 1.8) | 1.48 | (1.16, 1.89) |
| Anxiety | Pandemic | Black | 389040 | 80 | 9540013 | 0.84 | Reference |  | Reference |  | Reference |  |
| Anxiety | Pandemic | Mixed | 206060 | 68 | 5035801 | 1.35 | 1.63 | (1.18, 2.26) | 1.62 | (1.17, 2.23) | 1.78 | (1.29, 2.46) |
| Anxiety | Pandemic | Other | 334275 | 76 | 8015275 | 0.95 | 1.22 | (0.89, 1.66) | 1.22 | (0.89, 1.67) | 1.39 | (1.02, 1.91) |
| Anxiety | Wave 1 | White | 13008075 | 365 | 29413099 | 1.24 | 0.98 | (0.52, 1.85) | 0.95 | (0.5, 1.8) | 1.2 | (0.64, 2.28) |
| Anxiety | Wave 1 | Asian | 1116590 | 27 | 2525244 | 1.07 | 1.04 | (0.5, 2.16) | 1.06 | (0.51, 2.19) | 1.13 | (0.55, 2.34) |
| Anxiety | Wave 1 | Black | redacted |  |  |  |  |  |  |  |  |  |
| Anxiety | Wave 1 | Mixed | redacted |  |  |  |  |  |  |  |  |  |
| Anxiety | Wave 1 | Other | redacted |  |  |  |  |  |  |  |  |  |
| Anxiety | Easing 1 | White | 13059230 | 725 | 42519578 | 1.71 | 2.53 | (1.26, 5.1) | 2.37 | (1.18, 4.79) | 3.02 | (1.5, 6.1) |
| Anxiety | Easing 1 | Asian | 1128788 | 45 | 3674977 | 1.22 | 2.02 | (0.95, 4.3) | 2.03 | (0.95, 4.31) | 2.17 | (1.02, 4.61) |
| Anxiety | Easing 1 | Black | redacted |  |  |  |  |  |  |  |  |  |
| Anxiety | Easing 1 | Mixed | 208793 | 15 | 678914 | 2.21 | 3.51 | (1.49, 8.29) | 3.51 | (1.49, 8.29) | 3.98 | (1.69, 9.4) |
| Anxiety | Easing 1 | Other | 338288 | 12 | 1098296 | 1.09 | 1.9 | (0.78, 4.65) | 1.91 | (0.78, 4.69) | 2.28 | (0.93, 5.6) |
| Anxiety | Wave 2 | White | 12967234 | 1399 | 97842210 | 1.43 | 1.35 | (0.93, 1.96) | 1.28 | (0.89, 1.86) | 1.53 | (1.05, 2.22) |
| Anxiety | Wave 2 | Asian | 1122609 | 82 | 8461143 | 0.97 | 0.99 | (0.65, 1.52) | 1 | (0.65, 1.52) | 1.04 | (0.68, 1.59) |
| Anxiety | Wave 2 | Black | 390037 | 29 | 2935073 | 0.99 | Reference |  | Reference |  | Reference |  |
| Anxiety | Wave 2 | Mixed | 207205 | 27 | 1555417 | 1.74 | 1.75 | (1.04, 2.96) | 1.75 | (1.04, 2.96) | 1.92 | (1.14, 3.25) |
| Anxiety | Wave 2 | Other | 332902 | 17 | 2485666 | 0.68 | 0.73 | (0.4, 1.33) | 0.74 | (0.41, 1.35) | 0.84 | (0.46, 1.53) |
| Anxiety | Easing 2 | White | 12640489 | 237 | 13462945 | 1.76 | 1.79 | (0.66, 4.85) | 1.69 | (0.62, 4.6) | 2.03 | (0.75, 5.52) |
| Anxiety | Easing 2 | Asian | 1095138 | 19 | 1166268 | 1.63 | 1.73 | (0.59, 5.09) | 1.74 | (0.59, 5.12) | 1.81 | (0.61, 5.33) |
| Anxiety | Easing 2 | Black | redacted |  |  |  |  |  |  |  |  |  |
| Anxiety | Easing 2 | Mixed | redacted |  |  |  |  |  |  |  |  |  |
| Anxiety | Easing 2 | Other | redacted |  |  |  |  |  |  |  |  |  |
| Anxiety | Wave 3 | White | 12602332 | 1185 | 83187132 | 1.42 | 1.6 | (1.04, 2.47) | 1.57 | (1.01, 2.42) | 1.83 | (1.18, 2.84) |
| Anxiety | Wave 3 | Asian | 1091692 | 94 | 7195031 | 1.31 | 1.65 | (1.03, 2.66) | 1.65 | (1.02, 2.64) | 1.71 | (1.07, 2.75) |
| Anxiety | Wave 3 | Black | 377686 | 21 | 2483064 | 0.85 | Reference |  | Reference |  | Reference |  |
| Anxiety | Wave 3 | Mixed | 200227 | 15 | 1315159 | 1.14 | 1.39 | (0.71, 2.69) | 1.35 | (0.7, 2.62) | 1.47 | (0.76, 2.85) |
| Anxiety | Wave 3 | Other | 314768 | 22 | 2054920 | 1.07 | 1.41 | (0.78, 2.57) | 1.4 | (0.77, 2.55) | 1.56 | (0.86, 2.85) |
| Anxiety | Easing 3 | White | 12411761 | 691 | 55915750 | 1.24 | 1.93 | (1.03, 3.63) | 1.92 | (1.02, 3.6) | 2.24 | (1.19, 4.22) |
| Anxiety | Easing 3 | Asian | 1074699 | 48 | 4838179 | 0.99 | 1.62 | (0.82, 3.21) | 1.63 | (0.82, 3.22) | 1.67 | (0.85, 3.32) |
| Anxiety | Easing 3 | Black | redacted |  |  |  |  |  |  |  |  |  |
| Anxiety | Easing 3 | Mixed | redacted |  |  |  |  |  |  |  |  |  |
| Anxiety | Easing 3 | Other | 304150 | 11 | 1360271 | 0.81 | 1.39 | (0.59, 3.29) | 1.4 | (0.59, 3.3) | 1.57 | (0.66, 3.7) |
| Depression | Pre | White | 11951915 | 2218 | 299528329 | 0.74 | 1.91 | (1.37, 2.68) | 1.89 | (1.35, 2.65) | 2.48 | (1.77, 3.48) |
| Depression | Pre | Asian | 947198 | 83 | 23738551 | 0.35 | 0.95 | (0.64, 1.41) | 0.93 | (0.63, 1.39) | 1 | (0.67, 1.49) |
| Depression | Pre | Black | 323687 | 35 | 8111737 | 0.43 | Reference |  | Reference |  | Reference |  |
| Depression | Pre | Mixed | 167862 | 35 | 4206541 | 0.83 | 2.06 | (1.29, 3.29) | 2 | (1.25, 3.2) | 2.29 | (1.43, 3.66) |
| Depression | Pre | Other | 255875 | 23 | 6411754 | 0.36 | 1.02 | (0.6, 1.72) | 0.99 | (0.59, 1.68) | 1.2 | (0.71, 2.03) |
| Depression | Pandemic | White | 13007854 | 2181 | 321918259 | 0.68 | 1.55 | (1.13, 2.12) | 1.42 | (1.04, 1.94) | 1.8 | (1.32, 2.46) |
| Depression | Pandemic | Asian | 1116587 | 97 | 27564956 | 0.35 | 0.98 | (0.68, 1.41) | 0.96 | (0.67, 1.38) | 1.02 | (0.71, 1.48) |
| Depression | Pandemic | Black | 389040 | 41 | 9540463 | 0.43 | Reference |  | Reference |  | Reference |  |
| Depression | Pandemic | Mixed | 206060 | 27 | 5036547 | 0.54 | 1.28 | (0.79, 2.08) | 1.27 | (0.78, 2.06) | 1.43 | (0.88, 2.33) |
| Depression | Pandemic | Other | 334275 | 29 | 8015812 | 0.36 | 0.99 | (0.61, 1.59) | 0.98 | (0.61, 1.57) | 1.15 | (0.71, 1.85) |
| Depression | Wave 1 | White | 13008078 | 185 | 29413291 | 0.63 | 4.55 | (0.64, 32.59) | 4.1 | (0.57, 29.4) | 4.96 | (0.69, 35.68) |
| Depression | Wave 1 | Asian | redacted |  |  |  |  |  |  |  |  |  |
| Depression | Wave 1 | Black | redacted |  |  |  |  |  |  |  |  |  |
| Depression | Wave 1 | Mixed | redacted |  |  |  |  |  |  |  |  |  |
| Depression | Wave 1 | Other | redacted |  |  |  |  |  |  |  |  |  |
| Depression | Easing 1 | White | 13059246 | 406 | 42520051 | 0.95 | 0.99 | (0.56, 1.77) | 0.94 | (0.53, 1.68) | 1.23 | (0.69, 2.21) |
| Depression | Easing 1 | Asian | 1128788 | 16 | 3675015 | 0.44 | 0.6 | (0.28, 1.27) | 0.6 | (0.28, 1.27) | 0.64 | (0.3, 1.35) |
| Depression | Easing 1 | Black | 393362 | 12 | 1279474 | 0.94 | Reference |  | Reference |  | Reference |  |
| Depression | Easing 1 | Mixed | redacted |  |  |  |  |  |  |  |  |  |
| Depression | Easing 1 | Other | redacted |  |  |  |  |  |  |  |  |  |
| Depression | Wave 2 | White | 12967236 | 697 | 97844796 | 0.71 | 1.79 | (1.01, 3.19) | 1.65 | (0.93, 2.94) | 2.06 | (1.15, 3.67) |
| Depression | Wave 2 | Asian | 1122609 | 29 | 8461282 | 0.34 | 0.99 | (0.5, 1.94) | 0.97 | (0.49, 1.9) | 1.03 | (0.52, 2.02) |
| Depression | Wave 2 | Black | 390037 | 12 | 2935109 | 0.41 | Reference |  | Reference |  | Reference |  |
| Depression | Wave 2 | Mixed | redacted |  |  |  |  |  |  |  |  |  |
| Depression | Wave 2 | Other | redacted |  |  |  |  |  |  |  |  |  |
| Depression | Easing 2 | White | 12640487 | 108 | 13463005 | 0.8 | 1.63 | (0.4, 6.67) | 1.45 | (0.35, 5.96) | 1.73 | (0.42, 7.16) |
| Depression | Easing 2 | Asian | redacted |  |  |  |  |  |  |  |  |  |
| Depression | Easing 2 | Black | redacted |  |  |  |  |  |  |  |  |  |
| Depression | Easing 2 | Mixed | redacted |  |  |  |  |  |  |  |  |  |
| Depression | Easing 2 | Other | redacted |  |  |  |  |  |  |  |  |  |
| Depression | Wave 3 | White | 12602338 | 504 | 83189402 | 0.61 | 3.57 | (1.33, 9.57) | 3.21 | (1.19, 8.62) | 4.16 | (1.55, 11.18) |
| Depression | Wave 3 | Asian | 1091692 | 26 | 7195242 | 0.36 | 2.67 | (0.93, 7.66) | 2.61 | (0.91, 7.5) | 2.81 | (0.98, 8.05) |
| Depression | Wave 3 | Black | redacted |  |  |  |  |  |  |  |  |  |
| Depression | Wave 3 | Mixed | redacted |  |  |  |  |  |  |  |  |  |
| Depression | Wave 3 | Other | redacted |  |  |  |  |  |  |  |  |  |
| Depression | Easing 3 | White | 12411765 | 310 | 55916565 | 0.55 | 1.55 | (0.68, 3.49) | 1.37 | (0.6, 3.1) | 1.74 | (0.77, 3.94) |
| Depression | Easing 3 | Asian | 1074699 | 15 | 4838248 | 0.31 | 0.93 | (0.36, 2.39) | 0.91 | (0.35, 2.36) | 0.96 | (0.37, 2.48) |
| Depression | Easing 3 | Black | redacted |  |  |  |  |  |  |  |  |  |
| Depression | Easing 3 | Mixed | redacted |  |  |  |  |  |  |  |  |  |
| Depression | Easing 3 | Other | redacted |  |  |  |  |  |  |  |  |  |
| Asthma | Pre | White | 1104943 | 14508 | 27504757 | 52.75 | 0.71 | (0.64, 0.78) | 0.71 | (0.65, 0.79) | 0.86 | (0.78, 0.95) |
| Asthma | Pre | Asian | 72238 | 1870 | 1785399 | 104.74 | 1.27 | (1.14, 1.41) | 1.31 | (1.18, 1.45) | 1.35 | (1.21, 1.5) |
| Asthma | Pre | Black | 21726 | 436 | 538585 | 80.95 | Reference |  | Reference |  | Reference |  |
| Asthma | Pre | Mixed | 14090 | 227 | 350142 | 64.83 | 0.83 | (0.71, 0.97) | 0.83 | (0.71, 0.98) | 0.94 | (0.8, 1.11) |
| Asthma | Pre | Other | 11220 | 176 | 278865 | 63.11 | 0.79 | (0.67, 0.95) | 0.81 | (0.68, 0.97) | 0.98 | (0.82, 1.17) |
| Asthma | Pandemic | White | 1230577 | 9804 | 30424471 | 32.22 | 0.81 | (0.72, 0.91) | 0.83 | (0.73, 0.94) | 0.98 | (0.87, 1.11) |
| Asthma | Pandemic | Asian | 84572 | 1128 | 2090544 | 53.96 | 1.22 | (1.07, 1.39) | 1.27 | (1.11, 1.44) | 1.3 | (1.14, 1.49) |
| Asthma | Pandemic | Black | 25738 | 279 | 632429 | 44.12 | Reference |  | Reference |  | Reference |  |
| Asthma | Pandemic | Mixed | 17059 | 155 | 418542 | 37.03 | 0.88 | (0.72, 1.07) | 0.87 | (0.72, 1.06) | 0.97 | (0.8, 1.19) |
| Asthma | Pandemic | Other | 13884 | 115 | 340269 | 33.8 | 0.8 | (0.64, 1) | 0.82 | (0.66, 1.02) | 0.97 | (0.78, 1.2) |
| Asthma | Wave 1 | White | 1230560 | 1011 | 2780998 | 36.35 | 1.03 | (0.64, 1.66) | 1.07 | (0.67, 1.72) | 1.3 | (0.81, 2.1) |
| Asthma | Wave 1 | Asian | 84566 | 68 | 191126 | 35.58 | 1.06 | (0.63, 1.78) | 1.1 | (0.66, 1.86) | 1.14 | (0.67, 1.91) |
| Asthma | Wave 1 | Black | 25739 | 18 | 58115 | 30.97 | Reference |  | Reference |  | Reference |  |
| Asthma | Wave 1 | Mixed | 17045 | 12 | 38540 | 31.14 | 0.95 | (0.46, 1.98) | 0.97 | (0.46, 2.01) | 1.12 | (0.54, 2.32) |
| Asthma | Wave 1 | Other | redacted |  |  |  |  |  |  |  |  |  |
| Asthma | Easing 1 | White | 1239227 | 1453 | 4033325 | 36.02 | 0.95 | (0.67, 1.34) | 0.98 | (0.69, 1.39) | 1.23 | (0.86, 1.74) |
| Asthma | Easing 1 | Asian | 85465 | 110 | 278243 | 39.53 | 1 | (0.68, 1.47) | 1.04 | (0.71, 1.53) | 1.07 | (0.73, 1.57) |
| Asthma | Easing 1 | Black | 25983 | 34 | 84514 | 40.23 | Reference |  | Reference |  | Reference |  |
| Asthma | Easing 1 | Mixed | 17414 | 19 | 56614 | 33.56 | 0.85 | (0.49, 1.49) | 0.85 | (0.48, 1.49) | 0.98 | (0.56, 1.73) |
| Asthma | Easing 1 | Other | 14127 | 16 | 45931 | 34.83 | 0.92 | (0.51, 1.67) | 0.94 | (0.52, 1.71) | 1.2 | (0.66, 2.18) |
| Asthma | Wave 2 | White | 1235344 | 3169 | 9316516 | 34.01 | 0.72 | (0.58, 0.88) | 0.75 | (0.61, 0.92) | 0.91 | (0.74, 1.12) |
| Asthma | Wave 2 | Asian | 85717 | 311 | 646184 | 48.13 | 0.93 | (0.74, 1.16) | 0.97 | (0.77, 1.21) | 1 | (0.8, 1.25) |
| Asthma | Wave 2 | Black | 25961 | 101 | 195315 | 51.71 | Reference |  | Reference |  | Reference |  |
| Asthma | Wave 2 | Mixed | 17437 | 42 | 130946 | 32.07 | 0.65 | (0.45, 0.93) | 0.63 | (0.44, 0.9) | 0.72 | (0.5, 1.03) |
| Asthma | Wave 2 | Other | 14058 | 33 | 105601 | 31.25 | 0.63 | (0.43, 0.94) | 0.65 | (0.44, 0.96) | 0.8 | (0.54, 1.19) |
| Asthma | Easing 2 | White | 1207813 | 677 | 1286418 | 52.63 | 0.84 | (0.53, 1.32) | 0.88 | (0.56, 1.39) | 1.08 | (0.69, 1.71) |
| Asthma | Easing 2 | Asian | 84468 | 84 | 89957 | 93.38 | 1.25 | (0.77, 2.04) | 1.3 | (0.8, 2.13) | 1.34 | (0.82, 2.18) |
| Asthma | Easing 2 | Black | 25562 | 20 | 27222 | 73.47 | Reference |  | Reference |  | Reference |  |
| Asthma | Easing 2 | Mixed | redacted |  |  |  |  |  |  |  |  |  |
| Asthma | Easing 2 | Other | redacted |  |  |  |  |  |  |  |  |  |
| Asthma | Wave 3 | White | 1207064 | 3813 | 7964754 | 47.87 | 0.8 | (0.66, 0.96) | 0.83 | (0.69, 1) | 0.97 | (0.81, 1.17) |
| Asthma | Wave 3 | Asian | 84457 | 482 | 556459 | 86.62 | 1.21 | (0.99, 1.48) | 1.26 | (1.03, 1.54) | 1.29 | (1.06, 1.58) |
| Asthma | Wave 3 | Black | 25521 | 120 | 168043 | 71.41 | Reference |  | Reference |  | Reference |  |
| Asthma | Wave 3 | Mixed | 17084 | 75 | 112349 | 66.76 | 1 | (0.75, 1.34) | 0.98 | (0.73, 1.31) | 1.09 | (0.82, 1.46) |
| Asthma | Wave 3 | Other | 13693 | 52 | 89958 | 57.8 | 0.83 | (0.6, 1.15) | 0.85 | (0.61, 1.18) | 1 | (0.72, 1.39) |
| Asthma | Easing 3 | White | 1197790 | 2330 | 5394041 | 43.2 | 0.76 | (0.6, 0.96) | 0.77 | (0.61, 0.98) | 0.92 | (0.73, 1.17) |
| Asthma | Easing 3 | Asian | 84615 | 327 | 380842 | 85.86 | 1.29 | (1, 1.65) | 1.34 | (1.04, 1.72) | 1.38 | (1.07, 1.77) |
| Asthma | Easing 3 | Black | 25401 | 76 | 114225 | 66.54 | Reference |  | Reference |  | Reference |  |
| Asthma | Easing 3 | Mixed | 16959 | 46 | 76258 | 60.32 | 0.97 | (0.67, 1.4) | 0.97 | (0.67, 1.4) | 1.09 | (0.75, 1.57) |
| Asthma | Easing 3 | Other | 13585 | 35 | 61096 | 57.29 | 0.91 | (0.61, 1.36) | 0.94 | (0.63, 1.4) | 1.11 | (0.74, 1.65) |
| COPD | Pre | White | 409471 | 34264 | 9831716 | 348.5 | 1.57 | (1.33, 1.85) | 1.47 | (1.24, 1.73) | 1.59 | (1.35, 1.88) |
| COPD | Pre | Asian | 8290 | 531 | 200489 | 264.85 | 1.15 | (0.95, 1.38) | 1.13 | (0.94, 1.36) | 1.16 | (0.97, 1.4) |
| COPD | Pre | Black | 2556 | 144 | 62265 | 231.27 | Reference |  | Reference |  | Reference |  |
| COPD | Pre | Mixed | 1379 | 107 | 33202 | 322.27 | 1.44 | (1.12, 1.85) | 1.44 | (1.12, 1.85) | 1.53 | (1.19, 1.97) |
| COPD | Pre | Other | 1851 | 123 | 44939 | 273.7 | 1.23 | (0.96, 1.56) | 1.21 | (0.95, 1.53) | 1.3 | (1.02, 1.65) |
| COPD | Pandemic | White | 497200 | 33511 | 11478967 | 291.93 | 1.66 | (1.41, 1.97) | 1.55 | (1.31, 1.83) | 1.72 | (1.45, 2.04) |
| COPD | Pandemic | Asian | 10220 | 443 | 240147 | 184.47 | 1.02 | (0.84, 1.24) | 1 | (0.83, 1.21) | 1.03 | (0.85, 1.25) |
| COPD | Pandemic | Black | 3277 | 138 | 77025 | 179.16 | Reference |  | Reference |  | Reference |  |
| COPD | Pandemic | Mixed | 1766 | 92 | 41773 | 220.24 | 1.26 | (0.97, 1.64) | 1.29 | (0.99, 1.68) | 1.4 | (1.07, 1.82) |
| COPD | Pandemic | Other | 2405 | 97 | 56013 | 173.17 | 1 | (0.77, 1.3) | 0.98 | (0.76, 1.27) | 1.08 | (0.83, 1.4) |
| COPD | Wave 1 | White | 497196 | 3088 | 1114997 | 276.95 | 2.96 | (1.41, 6.24) | 2.8 | (1.33, 5.9) | 3.15 | (1.5, 6.64) |
| COPD | Wave 1 | Asian | 10221 | 32 | 22957 | 139.39 | 1.44 | (0.64, 3.27) | 1.41 | (0.62, 3.2) | 1.45 | (0.64, 3.28) |
| COPD | Wave 1 | Black | redacted |  |  |  |  |  |  |  |  |  |
| COPD | Wave 1 | Mixed | redacted |  |  |  |  |  |  |  |  |  |
| COPD | Wave 1 | Other | redacted |  |  |  |  |  |  |  |  |  |
| COPD | Easing 1 | White | 493737 | 5495 | 1591832 | 345.2 | 1.8 | (1.18, 2.74) | 1.68 | (1.1, 2.56) | 1.87 | (1.22, 2.84) |
| COPD | Easing 1 | Asian | 10226 | 64 | 33100 | 193.36 | 0.95 | (0.58, 1.54) | 0.93 | (0.57, 1.51) | 0.94 | (0.58, 1.53) |
| COPD | Easing 1 | Black | 3267 | 22 | 10548 | 208.57 | Reference |  | Reference |  | Reference |  |
| COPD | Easing 1 | Mixed | 1768 | 14 | 5735 | 244.1 | 1.22 | (0.63, 2.39) | 1.25 | (0.64, 2.44) | 1.35 | (0.69, 2.64) |
| COPD | Easing 1 | Other | 2407 | 16 | 7765 | 206.06 | 1.02 | (0.54, 1.94) | 1 | (0.53, 1.91) | 1.1 | (0.58, 2.09) |
| COPD | Wave 2 | White | 489153 | 10994 | 3607014 | 304.8 | 1.48 | (1.12, 1.96) | 1.38 | (1.04, 1.83) | 1.54 | (1.17, 2.04) |
| COPD | Wave 2 | Asian | 10158 | 146 | 75174 | 194.22 | 0.92 | (0.67, 1.27) | 0.9 | (0.65, 1.24) | 0.93 | (0.67, 1.28) |
| COPD | Wave 2 | Black | 3229 | 50 | 23911 | 209.11 | Reference |  | Reference |  | Reference |  |
| COPD | Wave 2 | Mixed | 1784 | 30 | 13200 | 227.26 | 1.11 | (0.7, 1.74) | 1.14 | (0.72, 1.79) | 1.24 | (0.79, 1.96) |
| COPD | Wave 2 | Other | 2372 | 32 | 17549 | 182.35 | 0.9 | (0.58, 1.4) | 0.88 | (0.57, 1.38) | 0.97 | (0.62, 1.51) |
| COPD | Easing 2 | White | 478271 | 2532 | 507374 | 499.04 | 1.57 | (0.87, 2.85) | 1.43 | (0.79, 2.61) | 1.6 | (0.88, 2.91) |
| COPD | Easing 2 | Asian | 10036 | 30 | 10656 | 281.52 | 0.88 | (0.44, 1.75) | 0.86 | (0.43, 1.73) | 0.88 | (0.44, 1.76) |
| COPD | Easing 2 | Black | 3242 | 11 | 3446 | 319.17 | Reference |  | Reference |  | Reference |  |
| COPD | Easing 2 | Mixed | redacted |  |  |  |  |  |  |  |  |  |
| COPD | Easing 2 | Other | redacted |  |  |  |  |  |  |  |  |  |
| COPD | Wave 3 | White | 477395 | 12942 | 3083669 | 419.69 | 1.75 | (1.33, 2.31) | 1.62 | (1.23, 2.14) | 1.82 | (1.38, 2.4) |
| COPD | Wave 3 | Asian | 10023 | 181 | 65161 | 277.77 | 1.12 | (0.82, 1.53) | 1.11 | (0.81, 1.51) | 1.14 | (0.84, 1.56) |
| COPD | Wave 3 | Black | 3229 | 51 | 21022 | 242.61 | Reference |  | Reference |  | Reference |  |
| COPD | Wave 3 | Mixed | 1809 | 30 | 11751 | 255.29 | 1.08 | (0.68, 1.69) | 1.1 | (0.7, 1.72) | 1.2 | (0.77, 1.89) |
| COPD | Wave 3 | Other | 2422 | 42 | 15769 | 266.35 | 1.14 | (0.75, 1.71) | 1.12 | (0.75, 1.69) | 1.25 | (0.83, 1.88) |
| COPD | Easing 3 | White | 472883 | 9016 | 2093051 | 430.76 | 1.52 | (1.12, 2.07) | 1.4 | (1.03, 1.9) | 1.57 | (1.16, 2.14) |
| COPD | Easing 3 | Asian | 9987 | 132 | 44390 | 297.37 | 1.01 | (0.71, 1.44) | 1 | (0.7, 1.41) | 1.03 | (0.72, 1.46) |
| COPD | Easing 3 | Black | 3230 | 41 | 14355 | 285.62 | Reference |  | Reference |  | Reference |  |
| COPD | Easing 3 | Mixed | 1849 | 31 | 8222 | 377.01 | 1.35 | (0.85, 2.16) | 1.4 | (0.88, 2.23) | 1.54 | (0.97, 2.46) |
| COPD | Easing 3 | Other | 2428 | 31 | 10808 | 286.83 | 1.05 | (0.66, 1.68) | 1.04 | (0.65, 1.66) | 1.16 | (0.73, 1.85) |

### Table 6: Number of events, rate, hazard ratio and confidence intervals by ethnic group, time period and outcome with those of Mixed ethnicity as the reference group.

| **Outcome** | **Period** | **Ethnicity** | **Denominator** | **events** | **Total person months** | **Rate per month** | **Unadjusted** | | **Age and sex adjusted** | | **Fully adjusted*** | |
| --- | --- | --- | --- | --- | --- | --- | --- | --- | --- | --- | --- | --- |
|  |  |  |  |  |  |  | **Hazard ratio** | **95% confidence interval** | **Hazard ratio** | **95% confidence interval** | **Hazard ratio** | **95% confidence interval** |
| Stroke | Pre | White | 11951861 | 42033 | 2.99E+08 | 14.05 | 2.17 | (1.92, 2.46) | 0.95 | (0.84, 1.08) | 1.03 | (0.91, 1.17) |
| Stroke | Pre | Asian | 947198 | 1835 | 23717609 | 7.74 | 1.35 | (1.18, 1.54) | 1.03 | (0.9, 1.18) | 1 | (0.87, 1.14) |
| Stroke | Pre | Black | 323687 | 683 | 8104085 | 8.43 | 1.48 | (1.28, 1.71) | 1.1 | (0.95, 1.28) | 1.01 | (0.88, 1.17) |
| Stroke | Pre | Mixed | 167862 | 249 | 4204083 | 5.92 | Reference |  | Reference |  | Reference |  |
| Stroke | Pre | Other | 255875 | 311 | 6408427 | 4.85 | 0.84 | (0.71, 0.99) | 0.74 | (0.62, 0.87) | 0.77 | (0.66, 0.91) |
| Stroke | Pandemic | White | 13007778 | 62025 | 3.21E+08 | 19.3 | 2.5 | (2.25, 2.77) | 1.03 | (0.92, 1.14) | 1.11 | (1, 1.23) |
| Stroke | Pandemic | Asian | 1116583 | 2669 | 27539949 | 9.69 | 1.41 | (1.26, 1.57) | 1.05 | (0.94, 1.17) | 1.02 | (0.91, 1.14) |
| Stroke | Pandemic | Black | 389039 | 1107 | 9530751 | 11.62 | 1.7 | (1.51, 1.91) | 1.22 | (1.08, 1.37) | 1.13 | (1.01, 1.28) |
| Stroke | Pandemic | Mixed | 206060 | 357 | 5033434 | 7.09 | Reference |  | Reference |  | Reference |  |
| Stroke | Pandemic | Other | 334274 | 485 | 8011353 | 6.05 | 0.88 | (0.77, 1.01) | 0.79 | (0.69, 0.91) | 0.82 | (0.72, 0.94) |
| Stroke | Wave 1 | White | 13008002 | 4886 | 29408734 | 16.61 | 3.24 | (2.13, 4.93) | 1.3 | (0.86, 1.98) | 1.42 | (0.94, 2.17) |
| Stroke | Wave 1 | Asian | 1116586 | 210 | 2525075 | 8.32 | 1.81 | (1.17, 2.81) | 1.32 | (0.85, 2.05) | 1.28 | (0.83, 1.99) |
| Stroke | Wave 1 | Black | 389021 | 79 | 879114 | 8.99 | 2.01 | (1.25, 3.22) | 1.41 | (0.88, 2.26) | 1.3 | (0.81, 2.09) |
| Stroke | Wave 1 | Mixed | 206091 | 22 | 465920 | 4.72 | Reference |  | Reference |  | Reference |  |
| Stroke | Wave 1 | Other | 334305 | 35 | 754927 | 4.64 | 1 | (0.59, 1.71) | 0.91 | (0.53, 1.54) | 0.94 | (0.55, 1.61) |
| Stroke | Easing 1 | White | 13059172 | 8104 | 42509205 | 19.06 | 2.41 | (1.82, 3.19) | 0.97 | (0.73, 1.29) | 1.05 | (0.79, 1.39) |
| Stroke | Easing 1 | Asian | 1128784 | 343 | 3674505 | 9.33 | 1.34 | (0.99, 1.8) | 0.99 | (0.73, 1.33) | 0.96 | (0.71, 1.29) |
| Stroke | Easing 1 | Black | 393360 | 143 | 1279287 | 11.18 | 1.62 | (1.17, 2.24) | 1.15 | (0.83, 1.59) | 1.07 | (0.77, 1.48) |
| Stroke | Easing 1 | Mixed | 208793 | 49 | 678885 | 7.22 | Reference |  | Reference |  | Reference |  |
| Stroke | Easing 1 | Other | 338288 | 66 | 1098220 | 6.01 | 0.87 | (0.6, 1.25) | 0.79 | (0.54, 1.14) | 0.81 | (0.56, 1.18) |
| Stroke | Wave 2 | White | 12967133 | 19186 | 97788237 | 19.62 | 2.44 | (2.03, 2.93) | 0.99 | (0.82, 1.19) | 1.07 | (0.89, 1.28) |
| Stroke | Wave 2 | Asian | 1122606 | 839 | 8458675 | 9.92 | 1.38 | (1.14, 1.68) | 1.02 | (0.84, 1.24) | 0.99 | (0.82, 1.21) |
| Stroke | Wave 2 | Black | 390035 | 338 | 2934168 | 11.52 | 1.61 | (1.31, 1.99) | 1.15 | (0.93, 1.42) | 1.06 | (0.86, 1.31) |
| Stroke | Wave 2 | Mixed | 207204 | 115 | 1555135 | 7.39 | Reference |  | Reference |  | Reference |  |
| Stroke | Wave 2 | Other | 332901 | 156 | 2485211 | 6.28 | 0.88 | (0.69, 1.12) | 0.79 | (0.62, 1) | 0.82 | (0.64, 1.04) |
| Stroke | Easing 2 | White | 12640411 | 2984 | 13461555 | 22.17 | 3.58 | (2.03, 6.31) | 1.48 | (0.84, 2.61) | 1.61 | (0.91, 2.85) |
| Stroke | Easing 2 | Asian | 1095134 | 114 | 1166215 | 9.78 | 1.8 | (0.99, 3.26) | 1.33 | (0.73, 2.41) | 1.29 | (0.71, 2.35) |
| Stroke | Easing 2 | Black | 379052 | 59 | 403523 | 14.62 | 2.74 | (1.47, 5.09) | 1.96 | (1.05, 3.65) | 1.8 | (0.97, 3.35) |
| Stroke | Easing 2 | Mixed | 201010 | 12 | 213928 | 5.61 | Reference |  | Reference |  | Reference |  |
| Stroke | Easing 2 | Other | 317013 | 23 | 337061 | 6.82 | 1.24 | (0.61, 2.48) | 1.1 | (0.55, 2.22) | 1.15 | (0.57, 2.31) |
| Stroke | Wave 3 | White | 12602238 | 16633 | 83144516 | 20 | 2.46 | (2.02, 3) | 1.03 | (0.84, 1.25) | 1.11 | (0.91, 1.36) |
| Stroke | Wave 3 | Asian | 1091687 | 756 | 7193014 | 10.51 | 1.46 | (1.18, 1.8) | 1.1 | (0.89, 1.36) | 1.07 | (0.86, 1.32) |
| Stroke | Wave 3 | Black | 377684 | 312 | 2482157 | 12.57 | 1.74 | (1.39, 2.19) | 1.26 | (1, 1.58) | 1.16 | (0.93, 1.46) |
| Stroke | Wave 3 | Mixed | 200227 | 98 | 1314892 | 7.45 | Reference |  | Reference |  | Reference |  |
| Stroke | Wave 3 | Other | 314766 | 148 | 2054547 | 7.2 | 1 | (0.77, 1.29) | 0.89 | (0.69, 1.15) | 0.92 | (0.72, 1.19) |
| Stroke | Easing 3 | White | 12411670 | 11376 | 55895206 | 20.35 | 2.33 | (1.85, 2.95) | 0.95 | (0.75, 1.2) | 1.02 | (0.81, 1.29) |
| Stroke | Easing 3 | Asian | 1074695 | 487 | 4837290 | 10.07 | 1.3 | (1.01, 1.67) | 0.97 | (0.76, 1.25) | 0.95 | (0.74, 1.21) |
| Stroke | Easing 3 | Black | 369667 | 210 | 1661122 | 12.64 | 1.64 | (1.25, 2.14) | 1.17 | (0.89, 1.53) | 1.09 | (0.83, 1.42) |
| Stroke | Easing 3 | Mixed | 197112 | 71 | 885370 | 8.020001 | Reference |  | Reference |  | Reference |  |
| Stroke | Easing 3 | Other | 304150 | 76 | 1360142 | 5.59 | 0.72 | (0.52, 0.99) | 0.63 | (0.46, 0.88) | 0.66 | (0.47, 0.91) |
| VTE | Pre | White | 11951881 | 28859 | 299215565 | 9.64 | 1.95 | (1.7, 2.25) | 1.29 | (1.12, 1.49) | 1.12 | (1.23, 1.63) |
| VTE | Pre | Asian | 947196 | 752 | 23730820 | 3.17 | 0.71 | (0.61, 0.83) | 0.63 | (0.53, 0.73) | 0.53 | (0.51, 0.7) |
| VTE | Pre | Black | 323685 | 501 | 8106277 | 6.18 | 1.37 | (1.16, 1.62) | 1.18 | (1, 1.39) | 1 | (0.89, 1.24) |
| VTE | Pre | Mixed | 167862 | 195 | 4204866 | 4.64 | Reference |  | Reference |  | Reference |  |
| VTE | Pre | Other | 255875 | 181 | 6409861 | 2.82 | 0.63 | (0.51, 0.77) | 0.59 | (0.48, 0.72) | 0.48 | (0.51, 0.76) |
| VTE | Pandemic | White | 13007823 | 38710 | 321532860 | 12.04 | 1.96 | (1.74, 2.2) | 1.23 | (1.1, 1.39) | 1.1 | (1.2, 1.52) |
| VTE | Pandemic | Asian | 1116586 | 939 | 27555834 | 3.41 | 0.63 | (0.55, 0.71) | 0.54 | (0.47, 0.61) | 0.47 | (0.45, 0.58) |
| VTE | Pandemic | Black | 389040 | 785 | 9532588 | 8.23 | 1.46 | (1.28, 1.68) | 1.22 | (1.07, 1.4) | 1.07 | (0.96, 1.26) |
| VTE | Pandemic | Mixed | 206060 | 290 | 5033737 | 5.76 | Reference |  | Reference |  | Reference |  |
| VTE | Pandemic | Other | 334273 | 275 | 8013201 | 3.43 | 0.61 | (0.52, 0.72) | 0.57 | (0.49, 0.68) | 0.49 | (0.52, 0.72) |
| VTE | Wave 1 | White | 13008047 | 2634 | 29410861 | 8.96 | 2.33 | (1.44, 3.75) | 1.45 | (0.9, 2.34) | 0.9 | (1, 2.61) |
| VTE | Wave 1 | Asian | 1116589 | 59 | 2525212 | 2.34 | 0.65 | (0.38, 1.12) | 0.55 | (0.32, 0.95) | 0.32 | (0.3, 0.9) |
| VTE | Wave 1 | Black | 389022 | 65 | 879116 | 7.39 | 2.07 | (1.21, 3.53) | 1.7 | (1, 2.9) | 1 | (0.88, 2.55) |
| VTE | Wave 1 | Mixed | 206091 | 17 | 465927 | 3.65 | Reference |  | Reference |  | Reference |  |
| VTE | Wave 1 | Other | 334304 | 17 | 754936 | 2.25 | 0.63 | (0.32, 1.24) | 0.6 | (0.3, 1.17) | 0.3 | (0.33, 1.26) |
| VTE | Easing 1 | White | 13059222 | 5157 | 42512892 | 12.13 | 2.15 | (1.55, 2.99) | 1.36 | (0.98, 1.89) | 0.98 | (1.09, 2.1) |
| VTE | Easing 1 | Asian | 1128788 | 116 | 3674863 | 3.16 | 0.63 | (0.43, 0.92) | 0.54 | (0.37, 0.78) | 0.37 | (0.35, 0.74) |
| VTE | Easing 1 | Black | 393361 | 93 | 1279337 | 7.27 | 1.42 | (0.96, 2.08) | 1.17 | (0.8, 1.72) | 0.8 | (0.71, 1.52) |
| VTE | Easing 1 | Mixed | 208793 | 36 | 678890 | 5.3 | Reference |  | Reference |  | Reference |  |
| VTE | Easing 1 | Other | 338288 | 39 | 1098249 | 3.55 | 0.68 | (0.43, 1.08) | 0.64 | (0.41, 1.01) | 0.41 | (0.44, 1.09) |
| VTE | Wave 2 | White | 12967150 | 12627 | 97803435 | 12.91 | 1.85 | (1.52, 2.25) | 1.15 | (0.94, 1.4) | 0.94 | (1.03, 1.53) |
| VTE | Wave 2 | Asian | 1122608 | 324 | 8460268 | 3.83 | 0.63 | (0.5, 0.79) | 0.54 | (0.43, 0.67) | 0.43 | (0.41, 0.64) |
| VTE | Wave 2 | Black | 390036 | 253 | 2934299 | 8.62 | 1.36 | (1.08, 1.72) | 1.13 | (0.89, 1.42) | 0.89 | (0.8, 1.27) |
| VTE | Wave 2 | Mixed | 207205 | 101 | 1555115 | 6.49 | Reference |  | Reference |  | Reference |  |
| VTE | Wave 2 | Other | 332902 | 80 | 2485458 | 3.22 | 0.51 | (0.38, 0.69) | 0.48 | (0.36, 0.64) | 0.36 | (0.38, 0.68) |
| VTE | Easing 2 | White | 12640457 | 1801 | 13462095 | 13.38 | 1.93 | (1.14, 3.26) | 1.19 | (0.7, 2.01) | 0.7 | (0.77, 2.22) |
| VTE | Easing 2 | Asian | 1095139 | 41 | 1166258 | 3.52 | 0.56 | (0.3, 1.02) | 0.47 | (0.26, 0.87) | 0.26 | (0.24, 0.82) |
| VTE | Easing 2 | Black | 379052 | 38 | 403537 | 9.42 | 1.47 | (0.79, 2.71) | 1.21 | (0.66, 2.24) | 0.66 | (0.58, 1.98) |
| VTE | Easing 2 | Mixed | 201011 | 14 | 213929 | 6.54 | Reference |  | Reference |  | Reference |  |
| VTE | Easing 2 | Other | 317012 | 12 | 337066 | 3.56 | 0.55 | (0.25, 1.19) | 0.52 | (0.24, 1.12) | 0.24 | (0.26, 1.2) |
| VTE | Wave 3 | White | 12602274 | 10091 | 83160465 | 12.13 | 1.87 | (1.5, 2.33) | 1.2 | (0.96, 1.5) | 0.96 | (1.06, 1.64) |
| VTE | Wave 3 | Asian | 1091690 | 249 | 7194558 | 3.46 | 0.61 | (0.47, 0.78) | 0.52 | (0.41, 0.67) | 0.41 | (0.39, 0.64) |
| VTE | Wave 3 | Black | 377684 | 202 | 2482470 | 8.14 | 1.37 | (1.06, 1.78) | 1.16 | (0.89, 1.5) | 0.89 | (0.8, 1.34) |
| VTE | Wave 3 | Mixed | 200227 | 80 | 1314939 | 6.08 | Reference |  | Reference |  | Reference |  |
| VTE | Wave 3 | Other | 314767 | 80 | 2054751 | 3.89 | 0.65 | (0.47, 0.88) | 0.61 | (0.45, 0.83) | 0.45 | (0.47, 0.88) |
| VTE | Easing 3 | White | 12411687 | 6834 | 55902234 | 12.22 | 2.19 | (1.64, 2.93) | 1.38 | (1.03, 1.85) | 1.03 | (1.11, 2) |
| VTE | Easing 3 | Asian | 1074698 | 184 | 4837902 | 3.8 | 0.78 | (0.56, 1.08) | 0.67 | (0.49, 0.93) | 0.49 | (0.47, 0.89) |
| VTE | Easing 3 | Black | 369670 | 136 | 1661279 | 8.19 | 1.61 | (1.15, 2.25) | 1.35 | (0.97, 1.89) | 0.97 | (0.88, 1.73) |
| VTE | Easing 3 | Mixed | 197112 | 46 | 885409 | 5.2 | Reference |  | Reference |  | Reference |  |
| VTE | Easing 3 | Other | 304148 | 41 | 1360203 | 3.01 | 0.59 | (0.39, 0.9) | 0.55 | (0.36, 0.84) | 0.36 | (0.38, 0.88) |
| Heart failure | Pre | White | 11951886 | 31037 | 2.99E+08 | 10.37 | 2.59 | (2.23, 3.02) | 0.91 | (0.78, 1.06) | 1.11 | (0.95, 1.29) |
| Heart failure | Pre | Asian | 947193 | 1925 | 23716286 | 8.12 | 2.02 | (1.72, 2.36) | 1.41 | (1.21, 1.66) | 1.35 | (1.15, 1.59) |
| Heart failure | Pre | Black | 323687 | 597 | 8104779 | 7.37 | 1.85 | (1.56, 2.2) | 1.28 | (1.07, 1.52) | 1.1 | (0.93, 1.31) |
| Heart failure | Pre | Mixed | 167861 | 166 | 4204968 | 3.95 | Reference |  | Reference |  | Reference |  |
| Heart failure | Pre | Other | 255875 | 222 | 6409411 | 3.46 | 0.9 | (0.73, 1.1) | 0.76 | (0.62, 0.93) | 0.85 | (0.7, 1.04) |
| Heart failure | Pandemic | White | 13007814 | 50729 | 3.22E+08 | 15.78 | 2.71 | (2.41, 3.04) | 0.89 | (0.79, 1) | 1.07 | (0.95, 1.2) |
| Heart failure | Pandemic | Asian | 1116584 | 2508 | 27542692 | 9.11 | 1.61 | (1.42, 1.82) | 1.1 | (0.97, 1.24) | 1.05 | (0.93, 1.19) |
| Heart failure | Pandemic | Black | 389038 | 842 | 9532933 | 8.83 | 1.57 | (1.37, 1.8) | 1.03 | (0.9, 1.18) | 0.9 | (0.78, 1.03) |
| Heart failure | Pandemic | Mixed | 206060 | 284 | 5034330 | 5.64 | Reference |  | Reference |  | Reference |  |
| Heart failure | Pandemic | Other | 334275 | 362 | 8013004 | 4.52 | 0.83 | (0.71, 0.96) | 0.72 | (0.61, 0.84) | 0.79 | (0.67, 0.92) |
| Heart failure | Wave 1 | White | 13008038 | 3540 | 29410481 | 12.04 | 3.17 | (1.97, 5.1) | 1.02 | (0.63, 1.64) | 1.23 | (0.76, 1.99) |
| Heart failure | Wave 1 | Asian | 1116587 | 185 | 2525095 | 7.33 | 2 | (1.22, 3.29) | 1.35 | (0.82, 2.21) | 1.28 | (0.78, 2.11) |
| Heart failure | Wave 1 | Black | 389020 | 65 | 879134 | 7.39 | 2.07 | (1.21, 3.53) | 1.33 | (0.78, 2.27) | 1.16 | (0.68, 1.98) |
| Heart failure | Wave 1 | Mixed | 206091 | 17 | 465929 | 3.65 | Reference |  | Reference |  | Reference |  |
| Heart failure | Wave 1 | Other | 334306 | 27 | 754939 | 3.58 | 0.97 | (0.52, 1.78) | 0.85 | (0.46, 1.57) | 0.94 | (0.51, 1.72) |
| Heart failure | Easing 1 | White | 13059194 | 6970 | 42510238 | 16.4 | 2.8 | (2.03, 3.85) | 0.9 | (0.65, 1.24) | 1.1 | (0.8, 1.52) |
| Heart failure | Easing 1 | Asian | 1128784 | 376 | 3674476 | 10.23 | 1.85 | (1.32, 2.58) | 1.25 | (0.9, 1.75) | 1.19 | (0.86, 1.67) |
| Heart failure | Easing 1 | Black | 393360 | 121 | 1279279 | 9.46 | 1.7 | (1.18, 2.44) | 1.11 | (0.77, 1.6) | 0.95 | (0.66, 1.37) |
| Heart failure | Easing 1 | Mixed | 208793 | 38 | 678881 | 5.6 | Reference |  | Reference |  | Reference |  |
| Heart failure | Easing 1 | Other | 338286 | 37 | 1098249 | 3.37 | 0.61 | (0.39, 0.96) | 0.54 | (0.34, 0.85) | 0.6 | (0.38, 0.94) |
| Heart failure | Wave 2 | White | 12967138 | 16378 | 97799415 | 16.75 | 2.84 | (2.3, 3.49) | 0.92 | (0.75, 1.14) | 1.12 | (0.91, 1.38) |
| Heart failure | Wave 2 | Asian | 1122605 | 844 | 8458766 | 9.98 | 1.74 | (1.39, 2.16) | 1.18 | (0.95, 1.47) | 1.12 | (0.9, 1.4) |
| Heart failure | Wave 2 | Black | 390036 | 311 | 2934208 | 10.6 | 1.87 | (1.47, 2.36) | 1.22 | (0.97, 1.55) | 1.06 | (0.84, 1.34) |
| Heart failure | Wave 2 | Mixed | 207204 | 89 | 1555247 | 5.72 | Reference |  | Reference |  | Reference |  |
| Heart failure | Wave 2 | Other | 332902 | 131 | 2485311 | 5.27 | 0.95 | (0.72, 1.24) | 0.83 | (0.64, 1.09) | 0.92 | (0.7, 1.2) |
| Heart failure | Easing 2 | White | 12640422 | 3464 | 13461250 | 25.73 | 2.8 | (1.78, 4.4) | 0.88 | (0.56, 1.38) | 1.08 | (0.68, 1.69) |
| Heart failure | Easing 2 | Asian | 1095135 | 199 | 1166170 | 17.06 | 1.93 | (1.2, 3.09) | 1.31 | (0.81, 2.09) | 1.24 | (0.78, 1.99) |
| Heart failure | Easing 2 | Black | 379052 | 52 | 403527 | 12.89 | 1.46 | (0.86, 2.47) | 0.94 | (0.56, 1.6) | 0.81 | (0.48, 1.36) |
| Heart failure | Easing 2 | Mixed | 201011 | 19 | 213926 | 8.88 | Reference |  | Reference |  | Reference |  |
| Heart failure | Easing 2 | Other | 317012 | 30 | 337056 | 8.9 | 1.03 | (0.58, 1.84) | 0.9 | (0.51, 1.6) | 0.99 | (0.56, 1.76) |
| Heart failure | Wave 3 | White | 12602204 | 14580 | 83149534 | 17.53 | 2.63 | (2.13, 3.25) | 0.87 | (0.71, 1.08) | 1.06 | (0.86, 1.31) |
| Heart failure | Wave 3 | Asian | 1091682 | 767 | 7192954 | 10.66 | 1.59 | (1.27, 1.99) | 1.09 | (0.87, 1.36) | 1.05 | (0.84, 1.3) |
| Heart failure | Wave 3 | Black | 377683 | 239 | 2482401 | 9.63 | 1.45 | (1.13, 1.85) | 0.94 | (0.74, 1.21) | 0.81 | (0.64, 1.04) |
| Heart failure | Wave 3 | Mixed | 200224 | 87 | 1314956 | 6.62 | Reference |  | Reference |  | Reference |  |
| Heart failure | Wave 3 | Other | 314766 | 119 | 2054623 | 5.79 | 0.89 | (0.68, 1.18) | 0.77 | (0.59, 1.02) | 0.85 | (0.65, 1.12) |
| Heart failure | Easing 3 | White | 12411683 | 11000 | 55896009 | 19.68 | 2.63 | (2.06, 3.35) | 0.86 | (0.68, 1.1) | 1.03 | (0.81, 1.32) |
| Heart failure | Easing 3 | Asian | 1074692 | 571 | 4837135 | 11.8 | 1.6 | (1.23, 2.06) | 1.1 | (0.85, 1.43) | 1.05 | (0.81, 1.36) |
| Heart failure | Easing 3 | Black | 369669 | 187 | 1661225 | 11.26 | 1.53 | (1.15, 2.03) | 1 | (0.75, 1.33) | 0.87 | (0.66, 1.15) |
| Heart failure | Easing 3 | Mixed | 197111 | 65 | 885385 | 7.34 | Reference |  | Reference |  | Reference |  |
| Heart failure | Easing 3 | Other | 304148 | 69 | 1360166 | 5.07 | 0.71 | (0.51, 1) | 0.61 | (0.43, 0.85) | 0.66 | (0.47, 0.92) |
| MI | Pre | White | 11951868 | 41681 | 2.99E+08 | 13.94 | 2.55 | (2.23, 2.92) | 1.25 | (1.09, 1.43) | 1.38 | (1.21, 1.58) |
| MI | Pre | Asian | 947193 | 3206 | 23700749 | 13.53 | 2.69 | (2.34, 3.1) | 2.1 | (1.83, 2.42) | 2.02 | (1.76, 2.33) |
| MI | Pre | Black | 323687 | 425 | 8107170 | 5.24 | 1.07 | (0.9, 1.26) | 0.79 | (0.67, 0.93) | 0.72 | (0.61, 0.85) |
| MI | Pre | Mixed | 167862 | 212 | 4204384 | 5.04 | Reference |  | Reference |  | Reference |  |
| MI | Pre | Other | 255872 | 329 | 6407889 | 5.13 | 1.05 | (0.89, 1.25) | 0.94 | (0.79, 1.11) | 0.98 | (0.83, 1.17) |
| MI | Pandemic | White | 13007792 | 49713 | 3.21E+08 | 15.47 | 2.83 | (2.5, 3.2) | 1.36 | (1.2, 1.54) | 1.49 | (1.31, 1.69) |
| MI | Pandemic | Asian | 1116586 | 3864 | 27525109 | 14.04 | 2.8 | (2.47, 3.18) | 2.15 | (1.89, 2.44) | 2.08 | (1.83, 2.36) |
| MI | Pandemic | Black | 389038 | 603 | 9534880 | 6.32 | 1.3 | (1.12, 1.51) | 0.95 | (0.82, 1.1) | 0.88 | (0.76, 1.01) |
| MI | Pandemic | Mixed | 206059 | 252 | 5033918 | 5.01 | Reference |  | Reference |  | Reference |  |
| MI | Pandemic | Other | 334275 | 480 | 8011100 | 5.99 | 1.24 | (1.06, 1.44) | 1.13 | (0.97, 1.31) | 1.17 | (1, 1.36) |
| MI | Wave 1 | White | 13008016 | 3585 | 29409854 | 12.19 | 3.06 | (1.9, 4.93) | 1.48 | (0.92, 2.38) | 1.64 | (1.01, 2.64) |
| MI | Wave 1 | Asian | 1116589 | 271 | 2525011 | 10.73 | 2.94 | (1.8, 4.8) | 2.23 | (1.36, 3.64) | 2.15 | (1.31, 3.51) |
| MI | Wave 1 | Black | 389020 | 33 | 879155 | 3.75 | 1.07 | (0.6, 1.92) | 0.77 | (0.43, 1.38) | 0.7 | (0.39, 1.26) |
| MI | Wave 1 | Mixed | 206090 | 17 | 465920 | 3.65 | Reference |  | Reference |  | Reference |  |
| MI | Wave 1 | Other | 334306 | 21 | 754936 | 2.78 | 0.75 | (0.39, 1.43) | 0.69 | (0.36, 1.32) | 0.72 | (0.38, 1.38) |
| MI | Easing 1 | White | 13059185 | 6342 | 42511189 | 14.92 | 2.94 | (2.06, 4.18) | 1.36 | (0.95, 1.93) | 1.5 | (1.05, 2.14) |
| MI | Easing 1 | Asian | 1128784 | 459 | 3674351 | 12.49 | 2.79 | (1.94, 4.02) | 2.11 | (1.46, 3.03) | 2.03 | (1.41, 2.92) |
| MI | Easing 1 | Black | 393362 | 71 | 1279392 | 5.55 | 1.27 | (0.83, 1.94) | 0.92 | (0.6, 1.4) | 0.84 | (0.55, 1.28) |
| MI | Easing 1 | Mixed | 208793 | 31 | 678906 | 4.57 | Reference |  | Reference |  | Reference |  |
| MI | Easing 1 | Other | 338288 | 66 | 1098217 | 6.01 | 1.37 | (0.89, 2.1) | 1.25 | (0.82, 1.92) | 1.31 | (0.85, 2) |
| MI | Wave 2 | White | 12967152 | 15520 | 97794466 | 15.87 | 2.75 | (2.21, 3.42) | 1.29 | (1.04, 1.61) | 1.41 | (1.13, 1.75) |
| MI | Wave 2 | Asian | 1122602 | 1172 | 8457313 | 13.86 | 2.68 | (2.14, 3.36) | 2.03 | (1.62, 2.55) | 1.96 | (1.56, 2.45) |
| MI | Wave 2 | Black | 390035 | 182 | 2934586 | 6.2 | 1.24 | (0.95, 1.61) | 0.9 | (0.69, 1.17) | 0.83 | (0.64, 1.08) |
| MI | Wave 2 | Mixed | 207205 | 81 | 1555226 | 5.21 | Reference |  | Reference |  | Reference |  |
| MI | Wave 2 | Other | 332901 | 152 | 2485197 | 6.12 | 1.22 | (0.93, 1.6) | 1.11 | (0.85, 1.46) | 1.16 | (0.88, 1.51) |
| MI | Easing 2 | White | 12640414 | 2489 | 13461750 | 18.49 | 3.99 | (2.07, 7.69) | 1.85 | (0.96, 3.57) | 2 | (1.04, 3.85) |
| MI | Easing 2 | Asian | 1095133 | 172 | 1166183 | 14.75 | 3.5 | (1.79, 6.85) | 2.66 | (1.36, 5.21) | 2.58 | (1.32, 5.05) |
| MI | Easing 2 | Black | 379051 | 39 | 403537 | 9.66 | 2.39 | (1.16, 4.94) | 1.74 | (0.84, 3.6) | 1.62 | (0.78, 3.34) |
| MI | Easing 2 | Mixed | redacted |  |  |  |  |  |  |  |  |  |
| MI | Easing 2 | Other | 317013 | 19 | 337062 | 5.64 | 1.37 | (0.62, 3.04) | 1.24 | (0.56, 2.75) | 1.29 | (0.58, 2.85) |
| MI | Wave 3 | White | 12602261 | 13201 | 83150575 | 15.88 | 3.23 | (2.5, 4.16) | 1.54 | (1.2, 1.99) | 1.69 | (1.31, 2.18) |
| MI | Wave 3 | Asian | 1091685 | 1085 | 7191830 | 15.09 | 3.29 | (2.53, 4.26) | 2.52 | (1.94, 3.27) | 2.43 | (1.87, 3.15) |
| MI | Wave 3 | Black | 377686 | 169 | 2482621 | 6.81 | 1.52 | (1.13, 2.04) | 1.11 | (0.82, 1.49) | 1.02 | (0.76, 1.37) |
| MI | Wave 3 | Mixed | 200225 | 60 | 1314987 | 4.56 | Reference |  | Reference |  | Reference |  |
| MI | Wave 3 | Other | 314768 | 133 | 2054569 | 6.47 | 1.48 | (1.09, 2.01) | 1.33 | (0.98, 1.8) | 1.38 | (1.02, 1.87) |
| MI | Easing 3 | White | 12411685 | 9252 | 55898155 | 16.55 | 2.58 | (1.97, 3.38) | 1.23 | (0.94, 1.61) | 1.35 | (1.03, 1.77) |
| MI | Easing 3 | Asian | 1074694 | 783 | 4836634 | 16.19 | 2.65 | (2, 3.5) | 2.02 | (1.53, 2.67) | 1.96 | (1.48, 2.58) |
| MI | Easing 3 | Black | 369668 | 115 | 1661334 | 6.92 | 1.17 | (0.85, 1.62) | 0.85 | (0.62, 1.18) | 0.79 | (0.57, 1.09) |
| MI | Easing 3 | Mixed | 197112 | 53 | 885392 | 5.99 | Reference |  | Reference |  | Reference |  |
| MI | Easing 3 | Other | 304150 | 98 | 1360084 | 7.21 | 1.24 | (0.88, 1.73) | 1.1 | (0.79, 1.54) | 1.14 | (0.81, 1.59) |
| DKA | Pre | White | 841518 | 4898 | 21022238 | 23.3 | 1.04 | (0.79, 1.37) | 1.67 | (1.27, 2.19) | 1.79 | (1.36, 2.35) |
| DKA | Pre | Asian | 115505 | 188 | 2892286 | 6.5 | 0.28 | (0.2, 0.37) | 0.31 | (0.23, 0.43) | 0.31 | (0.23, 0.42) |
| DKA | Pre | Black | 28706 | 121 | 717714 | 16.86 | 0.69 | (0.5, 0.96) | 0.83 | (0.6, 1.15) | 0.8 | (0.58, 1.1) |
| DKA | Pre | Mixed | 9109 | 53 | 227450 | 23.3 | Reference |  | Reference |  | Reference |  |
| DKA | Pre | Other | 12222 | 34 | 305866 | 11.12 | 0.47 | (0.3, 0.72) | 0.6 | (0.39, 0.92) | 0.61 | (0.4, 0.94) |
| DKA | Pandemic | White | 995361 | 5666 | 24030509 | 23.58 | 1.08 | (0.84, 1.38) | 1.64 | (1.28, 2.1) | 1.77 | (1.38, 2.26) |
| DKA | Pandemic | Asian | 142353 | 244 | 3500862 | 6.97 | 0.31 | (0.23, 0.41) | 0.35 | (0.27, 0.46) | 0.35 | (0.26, 0.46) |
| DKA | Pandemic | Black | 36530 | 214 | 887198 | 24.12 | 1.04 | (0.79, 1.37) | 1.23 | (0.94, 1.63) | 1.18 | (0.89, 1.56) |
| DKA | Pandemic | Mixed | 11738 | 65 | 286457 | 22.69 | Reference |  | Reference |  | Reference |  |
| DKA | Pandemic | Other | 16225 | 43 | 394708 | 10.89 | 0.47 | (0.32, 0.69) | 0.58 | (0.39, 0.85) | 0.59 | (0.4, 0.87) |
| DKA | Wave 1 | White | 995392 | 588 | 2242264 | 26.22 | 1.37 | (0.57, 3.33) | 2.23 | (0.92, 5.39) | 2.49 | (1.03, 6.03) |
| DKA | Wave 1 | Asian | 142342 | 23 | 321261 | 7.16 | 0.38 | (0.14, 1) | 0.44 | (0.17, 1.15) | 0.43 | (0.16, 1.13) |
| DKA | Wave 1 | Black | 36526 | 21 | 82234 | 25.54 | 1.35 | (0.51, 3.58) | 1.64 | (0.62, 4.36) | 1.53 | (0.57, 4.05) |
| DKA | Wave 1 | Mixed | redacted |  |  |  |  |  |  |  |  |  |
| DKA | Wave 1 | Other | redacted |  |  |  |  |  |  |  |  |  |
| DKA | Easing 1 | White | 992829 | 891 | 3223673 | 27.64 | 1.26 | (0.65, 2.45) | 2.03 | (1.05, 3.93) | 2.22 | (1.15, 4.29) |
| DKA | Easing 1 | Asian | 142905 | 31 | 465180 | 6.66 | 0.29 | (0.14, 0.6) | 0.33 | (0.16, 0.7) | 0.33 | (0.16, 0.69) |
| DKA | Easing 1 | Black | 36652 | 37 | 119132 | 31.06 | 1.28 | (0.62, 2.66) | 1.59 | (0.77, 3.29) | 1.5 | (0.72, 3.11) |
| DKA | Easing 1 | Mixed | redacted |  |  |  |  |  |  |  |  |  |
| DKA | Easing 1 | Other | redacted |  |  |  |  |  |  |  |  |  |
| DKA | Wave 2 | White | 993240 | 2005 | 7442036 | 26.94 | 0.73 | (0.52, 1.03) | 1.16 | (0.82, 1.63) | 1.26 | (0.89, 1.79) |
| DKA | Wave 2 | Asian | 143331 | 89 | 1079487 | 8.24 | 0.22 | (0.15, 0.33) | 0.26 | (0.17, 0.39) | 0.26 | (0.17, 0.38) |
| DKA | Wave 2 | Black | 36803 | 65 | 276312 | 23.52 | 0.63 | (0.42, 0.97) | 0.77 | (0.51, 1.17) | 0.73 | (0.48, 1.11) |
| DKA | Wave 2 | Mixed | 11829 | 33 | 88847 | 37.14 | Reference |  | Reference |  | Reference |  |
| DKA | Wave 2 | Other | 16467 | 13 | 123610 | 10.52 | 0.28 | (0.15, 0.53) | 0.35 | (0.18, 0.67) | 0.36 | (0.19, 0.68) |
| DKA | Easing 2 | White | 997807 | 363 | 1062042 | 34.18 | 0.82 | (0.36, 1.86) | 1.3 | (0.58, 2.93) | 1.42 | (0.63, 3.21) |
| DKA | Easing 2 | Asian | 144411 | 17 | 153794 | 11.05 | 0.22 | (0.09, 0.57) | 0.27 | (0.11, 0.68) | 0.26 | (0.1, 0.67) |
| DKA | Easing 2 | Black | 37160 | 13 | 39575 | 32.85 | 0.65 | (0.25, 1.71) | 0.81 | (0.31, 2.12) | 0.76 | (0.29, 2) |
| DKA | Easing 2 | Mixed | redacted |  |  |  |  |  |  |  |  |  |
| DKA | Easing 2 | Other | redacted |  |  |  |  |  |  |  |  |  |
| DKA | Wave 3 | White | 999832 | 1974 | 6568038 | 30.05 | 0.97 | (0.65, 1.44) | 1.54 | (1.03, 2.29) | 1.66 | (1.11, 2.46) |
| DKA | Wave 3 | Asian | 144709 | 84 | 955023 | 8.8 | 0.28 | (0.18, 0.44) | 0.33 | (0.21, 0.52) | 0.33 | (0.21, 0.51) |
| DKA | Wave 3 | Black | 37309 | 70 | 245487 | 28.51 | 0.89 | (0.56, 1.4) | 1.09 | (0.69, 1.73) | 1.04 | (0.66, 1.64) |
| DKA | Wave 3 | Mixed | 12024 | 25 | 79183 | 31.57 | Reference |  | Reference |  | Reference |  |
| DKA | Wave 3 | Other | 16735 | 18 | 110066 | 16.35 | 0.52 | (0.28, 0.95) | 0.65 | (0.36, 1.2) | 0.66 | (0.36, 1.21) |
| DKA | Easing 3 | White | 1004850 | 1402 | 4506363 | 31.11 | 1.07 | (0.66, 1.73) | 1.66 | (1.03, 2.69) | 1.76 | (1.09, 2.84) |
| DKA | Easing 3 | Asian | 146317 | 65 | 658544 | 9.87 | 0.32 | (0.19, 0.55) | 0.38 | (0.22, 0.64) | 0.37 | (0.22, 0.63) |
| DKA | Easing 3 | Black | 37795 | 55 | 169620 | 32.43 | 1.02 | (0.59, 1.75) | 1.23 | (0.72, 2.13) | 1.18 | (0.68, 2.04) |
| DKA | Easing 3 | Mixed | 12233 | 17 | 54888 | 30.97 | Reference |  | Reference |  | Reference |  |
| DKA | Easing 3 | Other | 16941 | 13 | 76042 | 17.1 | 0.54 | (0.26, 1.11) | 0.67 | (0.32, 1.37) | 0.67 | (0.33, 1.38) |
| T1 DM | Pre | White | 87911 | 5492 | 2126366 | 258.28 | 0.92 | (0.72, 1.16) | 1.07 | (0.85, 1.36) | 1.22 | (0.96, 1.55) |
| T1 DM | Pre | Asian | 5914 | 141 | 146226 | 96.43 | 0.3 | (0.23, 0.4) | 0.39 | (0.29, 0.52) | 0.38 | (0.29, 0.51) |
| T1 DM | Pre | Black | 2450 | 105 | 59963 | 175.11 | 0.54 | (0.4, 0.73) | 0.67 | (0.49, 0.91) | 0.62 | (0.45, 0.83) |
| T1 DM | Pre | Mixed | 956 | 70 | 22919 | 305.42 | Reference |  | Reference |  | Reference |  |
| T1 DM | Pre | Other | 949 | 40 | 23225 | 172.23 | 0.54 | (0.37, 0.8) | 0.62 | (0.42, 0.91) | 0.63 | (0.43, 0.94) |
| T1 DM | Pandemic | White | 100454 | 5530 | 2385604 | 231.81 | 1.09 | (0.85, 1.4) | 1.26 | (0.98, 1.62) | 1.46 | (1.14, 1.88) |
| T1 DM | Pandemic | Asian | 7274 | 143 | 175440 | 81.51 | 0.34 | (0.25, 0.46) | 0.43 | (0.32, 0.58) | 0.42 | (0.31, 0.57) |
| T1 DM | Pandemic | Black | 3004 | 125 | 70660 | 176.9 | 0.74 | (0.55, 1) | 0.89 | (0.66, 1.21) | 0.81 | (0.6, 1.1) |
| T1 DM | Pandemic | Mixed | 1180 | 63 | 27871 | 226.04 | Reference |  | Reference |  | Reference |  |
| T1 DM | Pandemic | Other | 1255 | 52 | 29573 | 175.84 | 0.74 | (0.51, 1.07) | 0.83 | (0.57, 1.19) | 0.85 | (0.59, 1.23) |
| T1 DM | Wave 1 | White | 100463 | 616 | 226053 | 272.5 | 0.99 | (0.47, 2.09) | 1.19 | (0.56, 2.52) | 1.42 | (0.67, 3.01) |
| T1 DM | Wave 1 | Asian | 7275 | 13 | 16397 | 79.28 | 0.31 | (0.12, 0.77) | 0.4 | (0.16, 1.01) | 0.38 | (0.15, 0.96) |
| T1 DM | Wave 1 | Black | 3001 | 11 | 6726 | 163.55 | 0.63 | (0.24, 1.63) | 0.79 | (0.31, 2.04) | 0.69 | (0.27, 1.79) |
| T1 DM | Wave 1 | Mixed | redacted |  |  |  |  |  |  |  |  |  |
| T1 DM | Wave 1 | Other | redacted |  |  |  |  |  |  |  |  |  |
| T1 DM | Easing 1 | White | 100755 | 1042 | 325820 | 319.81 | 0.97 | (0.57, 1.64) | 1.14 | (0.67, 1.94) | 1.31 | (0.77, 2.23) |
| T1 DM | Easing 1 | Asian | 7311 | 23 | 23742 | 96.87 | 0.25 | (0.13, 0.49) | 0.34 | (0.17, 0.65) | 0.33 | (0.17, 0.64) |
| T1 DM | Easing 1 | Black | 3027 | 29 | 9786 | 296.35 | 0.77 | (0.41, 1.47) | 0.95 | (0.5, 1.8) | 0.86 | (0.46, 1.64) |
| T1 DM | Easing 1 | Mixed | 1194 | 14 | 3863 | 362.4 | Reference |  | Reference |  | Reference |  |
| T1 DM | Easing 1 | Other | redacted |  |  |  |  |  |  |  |  |  |
| T1 DM | Wave 2 | White | 100749 | 2105 | 749144 | 280.99 | 0.86 | (0.6, 1.24) | 1.02 | (0.71, 1.46) | 1.21 | (0.84, 1.73) |
| T1 DM | Wave 2 | Asian | 7314 | 50 | 54660 | 91.47 | 0.25 | (0.16, 0.4) | 0.33 | (0.21, 0.52) | 0.32 | (0.2, 0.5) |
| T1 DM | Wave 2 | Black | 3052 | 46 | 22633 | 203.24 | 0.56 | (0.35, 0.89) | 0.68 | (0.43, 1.09) | 0.61 | (0.39, 0.97) |
| T1 DM | Wave 2 | Mixed | 1187 | 30 | 8769 | 342.1 | Reference |  | Reference |  | Reference |  |
| T1 DM | Wave 2 | Other | 1263 | 15 | 9374 | 160.02 | 0.45 | (0.24, 0.83) | 0.51 | (0.27, 0.94) | 0.53 | (0.28, 0.99) |
| T1 DM | Easing 2 | White | 100533 | 408 | 106809 | 381.99 | 0.84 | (0.37, 1.88) | 1.03 | (0.46, 2.31) | 1.18 | (0.53, 2.67) |
| T1 DM | Easing 2 | Asian | 7334 | 14 | 7801 | 179.46 | 0.36 | (0.14, 0.93) | 0.48 | (0.19, 1.26) | 0.46 | (0.18, 1.2) |
| T1 DM | Easing 2 | Black | redacted |  |  |  |  |  |  |  |  |  |
| T1 DM | Easing 2 | Mixed | redacted |  |  |  |  |  |  |  |  |  |
| T1 DM | Easing 2 | Other | redacted |  |  |  |  |  |  |  |  |  |
| T1 DM | Wave 3 | White | 100692 | 1975 | 656541 | 300.82 | 0.87 | (0.6, 1.28) | 1.06 | (0.73, 1.56) | 1.2 | (0.82, 1.76) |
| T1 DM | Wave 3 | Asian | 7359 | 58 | 48340 | 119.98 | 0.33 | (0.21, 0.52) | 0.44 | (0.28, 0.69) | 0.42 | (0.27, 0.67) |
| T1 DM | Wave 3 | Black | 3075 | 63 | 19944 | 315.88 | 0.86 | (0.55, 1.35) | 1.08 | (0.69, 1.7) | 0.98 | (0.62, 1.54) |
| T1 DM | Wave 3 | Mixed | 1172 | 27 | 7621 | 354.27 | Reference |  | Reference |  | Reference |  |
| T1 DM | Wave 3 | Other | 1257 | 17 | 8156 | 208.44 | 0.57 | (0.31, 1.05) | 0.66 | (0.36, 1.2) | 0.67 | (0.36, 1.23) |
| T1 DM | Easing 3 | White | 100982 | 1461 | 450456 | 324.34 | 1.02 | (0.64, 1.63) | 1.25 | (0.78, 1.99) | 1.42 | (0.89, 2.27) |
| T1 DM | Easing 3 | Asian | 7439 | 46 | 33317 | 138.07 | 0.39 | (0.22, 0.67) | 0.52 | (0.3, 0.89) | 0.49 | (0.29, 0.85) |
| T1 DM | Easing 3 | Black | 3126 | 33 | 13941 | 236.72 | 0.66 | (0.37, 1.18) | 0.83 | (0.47, 1.47) | 0.74 | (0.42, 1.32) |
| T1 DM | Easing 3 | Mixed | 1196 | 18 | 5313 | 338.81 | Reference |  | Reference |  | Reference |  |
| T1 DM | Easing 3 | Other | 1243 | 16 | 5550 | 288.3 | 0.82 | (0.42, 1.61) | 0.94 | (0.48, 1.85) | 0.95 | (0.48, 1.86) |
| T2 DM | Pre | White | 791860 | 8195 | 19744444 | 41.51 | 1.26 | (1, 1.59) | 1.29 | (1.02, 1.62) | 1.5 | (1.19, 1.89) |
| T2 DM | Pre | Asian | 114073 | 783 | 2849512 | 27.48 | 0.79 | (0.63, 1.01) | 0.79 | (0.62, 1) | 0.82 | (0.65, 1.04) |
| T2 DM | Pre | Black | 27906 | 348 | 695157 | 50.06 | 1.39 | (1.08, 1.78) | 1.38 | (1.07, 1.77) | 1.27 | (0.99, 1.63) |
| T2 DM | Pre | Mixed | 8600 | 76 | 214680 | 35.4 | Reference |  | Reference |  | Reference |  |
| T2 DM | Pre | Other | 11805 | 87 | 294856 | 29.51 | 0.84 | (0.62, 1.15) | 0.85 | (0.62, 1.16) | 0.89 | (0.65, 1.21) |
| T2 DM | Pandemic | White | 941455 | 11040 | 22648478 | 48.74 | 1.11 | (0.93, 1.33) | 1.1 | (0.92, 1.32) | 1.28 | (1.07, 1.53) |
| T2 DM | Pandemic | Asian | 140656 | 892 | 3452607 | 25.84 | 0.58 | (0.48, 0.7) | 0.58 | (0.48, 0.7) | 0.6 | (0.49, 0.72) |
| T2 DM | Pandemic | Black | 35567 | 482 | 861384 | 55.96 | 1.2 | (0.99, 1.47) | 1.19 | (0.98, 1.45) | 1.1 | (0.9, 1.34) |
| T2 DM | Pandemic | Mixed | 11131 | 124 | 271208 | 45.72 | Reference |  | Reference |  | Reference |  |
| T2 DM | Pandemic | Other | 15672 | 122 | 380633 | 32.05 | 0.7 | (0.54, 0.89) | 0.69 | (0.54, 0.89) | 0.73 | (0.56, 0.93) |
| T2 DM | Wave 1 | White | 941483 | 870 | 2120264 | 41.03 | 1.51 | (0.72, 3.18) | 1.52 | (0.72, 3.22) | 1.84 | (0.87, 3.89) |
| T2 DM | Wave 1 | Asian | 140644 | 57 | 317405 | 17.96 | 0.68 | (0.31, 1.49) | 0.68 | (0.31, 1.49) | 0.7 | (0.32, 1.55) |
| T2 DM | Wave 1 | Black | 35564 | 40 | 80057 | 49.96 | 1.81 | (0.81, 4.04) | 1.78 | (0.8, 3.98) | 1.61 | (0.72, 3.6) |
| T2 DM | Wave 1 | Mixed | redacted |  |  |  |  |  |  |  |  |  |
| T2 DM | Wave 1 | Other | 15687 | 11 | 35357 | 31.11 | 1.11 | (0.43, 2.88) | 1.11 | (0.43, 2.86) | 1.16 | (0.45, 3) |
| T2 DM | Easing 1 | White | 938717 | 1720 | 3046173 | 56.46 | 1.67 | (0.96, 2.88) | 1.63 | (0.94, 2.82) | 1.91 | (1.1, 3.31) |
| T2 DM | Easing 1 | Asian | 141193 | 122 | 459473 | 26.55 | 0.77 | (0.43, 1.37) | 0.77 | (0.43, 1.36) | 0.8 | (0.45, 1.42) |
| T2 DM | Easing 1 | Black | 35668 | 77 | 115876 | 66.45 | 1.81 | (1.01, 3.27) | 1.79 | (0.99, 3.23) | 1.65 | (0.92, 2.97) |
| T2 DM | Easing 1 | Mixed | 11151 | 13 | 36284 | 35.83 | Reference |  | Reference |  | Reference |  |
| T2 DM | Easing 1 | Other | 15777 | 20 | 51264 | 39.01 | 1.09 | (0.54, 2.2) | 1.08 | (0.54, 2.18) | 1.14 | (0.57, 2.29) |
| T2 DM | Wave 2 | White | 939511 | 3523 | 7032361 | 50.1 | 0.97 | (0.72, 1.3) | 0.97 | (0.73, 1.31) | 1.15 | (0.86, 1.54) |
| T2 DM | Wave 2 | Asian | 141615 | 291 | 1065878 | 27.3 | 0.51 | (0.37, 0.7) | 0.51 | (0.37, 0.7) | 0.53 | (0.39, 0.72) |
| T2 DM | Wave 2 | Black | 35811 | 160 | 268616 | 59.56 | 1.07 | (0.77, 1.49) | 1.07 | (0.77, 1.48) | 0.98 | (0.7, 1.36) |
| T2 DM | Wave 2 | Mixed | 11220 | 46 | 84263 | 54.59 | Reference |  | Reference |  | Reference |  |
| T2 DM | Wave 2 | Other | 15915 | 43 | 119405 | 36.01 | 0.65 | (0.43, 0.98) | 0.65 | (0.43, 0.98) | 0.68 | (0.45, 1.03) |
| T2 DM | Easing 2 | White | 945374 | 624 | 1006055 | 62.02 | 0.98 | (0.49, 1.99) | 0.96 | (0.48, 1.94) | 1.14 | (0.56, 2.3) |
| T2 DM | Easing 2 | Asian | 142718 | 61 | 151971 | 40.14 | 0.62 | (0.3, 1.31) | 0.62 | (0.3, 1.3) | 0.65 | (0.31, 1.36) |
| T2 DM | Easing 2 | Black | 36188 | 31 | 38532 | 80.45 | 1.19 | (0.55, 2.59) | 1.17 | (0.54, 2.55) | 1.08 | (0.49, 2.35) |
| T2 DM | Easing 2 | Mixed | redacted |  |  |  |  |  |  |  |  |  |
| T2 DM | Easing 2 | Other | redacted |  |  |  |  |  |  |  |  |  |
| T2 DM | Wave 3 | White | 947473 | 3295 | 6218927 | 52.98 | 0.96 | (0.71, 1.29) | 0.94 | (0.7, 1.27) | 1.09 | (0.81, 1.47) |
| T2 DM | Wave 3 | Asian | 143015 | 289 | 943099 | 30.64 | 0.54 | (0.39, 0.75) | 0.54 | (0.39, 0.74) | 0.56 | (0.41, 0.77) |
| T2 DM | Wave 3 | Black | 36330 | 127 | 238909 | 53.16 | 0.89 | (0.63, 1.26) | 0.89 | (0.63, 1.25) | 0.82 | (0.58, 1.15) |
| T2 DM | Wave 3 | Mixed | 11430 | 44 | 75226 | 58.49 | Reference |  | Reference |  | Reference |  |
| T2 DM | Wave 3 | Other | 16203 | 39 | 106510 | 36.62 | 0.63 | (0.41, 0.96) | 0.62 | (0.4, 0.95) | 0.65 | (0.42, 1) |
| T2 DM | Easing 3 | White | 953549 | 2229 | 4273597 | 52.16 | 0.9 | (0.63, 1.28) | 0.89 | (0.62, 1.26) | 1.01 | (0.71, 1.44) |
| T2 DM | Easing 3 | Asian | 144636 | 182 | 650752 | 27.97 | 0.45 | (0.31, 0.66) | 0.45 | (0.31, 0.66) | 0.47 | (0.32, 0.68) |
| T2 DM | Easing 3 | Black | 36828 | 113 | 165179 | 68.41 | 1.08 | (0.73, 1.6) | 1.07 | (0.72, 1.59) | 1 | (0.68, 1.49) |
| T2 DM | Easing 3 | Mixed | 11643 | 32 | 52210 | 61.29 | Reference |  | Reference |  | Reference |  |
| T2 DM | Easing 3 | Other | 16434 | 13 | 73768 | 17.62 | 0.28 | (0.15, 0.54) | 0.28 | (0.15, 0.53) | 0.29 | (0.15, 0.55) |
| Anxiety | Pre | White | 11951915 | 4087 | 3E+08 | 1.36 | 1.35 | (0.99, 1.82) | 1.27 | (0.94, 1.73) | 1.41 | (1.04, 1.91) |
| Anxiety | Pre | Asian | 947198 | 279 | 23736235 | 1.18 | 1.19 | (0.86, 1.65) | 1.19 | (0.86, 1.65) | 1.13 | (0.82, 1.57) |
| Anxiety | Pre | Black | 323687 | 60 | 8111470 | 0.74 | 0.73 | (0.49, 1.08) | 0.73 | (0.49, 1.08) | 0.66 | (0.44, 0.97) |
| Anxiety | Pre | Mixed | 167862 | 42 | 4206466 | 1 | Reference |  | Reference |  | Reference |  |
| Anxiety | Pre | Other | 255875 | 53 | 6411343 | 0.83 | 0.88 | (0.59, 1.32) | 0.89 | (0.59, 1.34) | 0.94 | (0.62, 1.41) |
| Anxiety | Pandemic | White | 13007851 | 4604 | 3.22E+08 | 1.43 | 1 | (0.78, 1.27) | 0.97 | (0.76, 1.24) | 1.06 | (0.83, 1.35) |
| Anxiety | Pandemic | Asian | 1116587 | 316 | 27562426 | 1.15 | 0.86 | (0.66, 1.12) | 0.87 | (0.67, 1.13) | 0.83 | (0.64, 1.08) |
| Anxiety | Pandemic | Black | 389040 | 80 | 9540013 | 0.84 | 0.61 | (0.44, 0.85) | 0.62 | (0.45, 0.86) | 0.56 | (0.41, 0.78) |
| Anxiety | Pandemic | Mixed | 206060 | 68 | 5035801 | 1.35 | Reference |  | Reference |  | Reference |  |
| Anxiety | Pandemic | Other | 334275 | 76 | 8015275 | 0.95 | 0.74 | (0.54, 1.03) | 0.75 | (0.54, 1.05) | 0.78 | (0.56, 1.09) |
| Anxiety | Wave 1 | White | 13008075 | 365 | 29413099 | 1.24 | 1.04 | (0.43, 2.51) | 0.98 | (0.4, 2.38) | 1.1 | (0.45, 2.67) |
| Anxiety | Wave 1 | Asian | 1116590 | 27 | 2525244 | 1.07 | 1.1 | (0.42, 2.87) | 1.09 | (0.42, 2.84) | 1.03 | (0.4, 2.69) |
| Anxiety | Wave 1 | Black | redacted |  |  |  |  |  |  |  |  |  |
| Anxiety | Wave 1 | Mixed | redacted |  |  |  |  |  |  |  |  |  |
| Anxiety | Wave 1 | Other | redacted |  |  |  |  |  |  |  |  |  |
| Anxiety | Easing 1 | White | 13059230 | 725 | 42519578 | 1.71 | 0.72 | (0.43, 1.21) | 0.68 | (0.4, 1.13) | 0.76 | (0.45, 1.27) |
| Anxiety | Easing 1 | Asian | 1128788 | 45 | 3674977 | 1.22 | 0.58 | (0.32, 1.04) | 0.58 | (0.32, 1.04) | 0.54 | (0.3, 0.98) |
| Anxiety | Easing 1 | Black | redacted |  |  |  |  |  |  |  |  |  |
| Anxiety | Easing 1 | Mixed | 208793 | 15 | 678914 | 2.21 | Reference |  | Reference |  | Reference |  |
| Anxiety | Easing 1 | Other | 338288 | 12 | 1098296 | 1.09 | 0.54 | (0.25, 1.16) | 0.54 | (0.25, 1.16) | 0.57 | (0.27, 1.23) |
| Anxiety | Wave 2 | White | 12967234 | 1399 | 97842210 | 1.43 | 0.77 | (0.53, 1.13) | 0.73 | (0.5, 1.08) | 0.8 | (0.54, 1.17) |
| Anxiety | Wave 2 | Asian | 1122609 | 82 | 8461143 | 0.97 | 0.57 | (0.37, 0.87) | 0.57 | (0.37, 0.88) | 0.54 | (0.35, 0.84) |
| Anxiety | Wave 2 | Black | 390037 | 29 | 2935073 | 0.99 | 0.57 | (0.34, 0.96) | 0.57 | (0.34, 0.96) | 0.52 | (0.31, 0.88) |
| Anxiety | Wave 2 | Mixed | 207205 | 27 | 1555417 | 1.74 | Reference |  | Reference |  | Reference |  |
| Anxiety | Wave 2 | Other | 332902 | 17 | 2485666 | 0.68 | 0.42 | (0.23, 0.77) | 0.42 | (0.23, 0.77) | 0.44 | (0.24, 0.8) |
| Anxiety | Easing 2 | White | 12640489 | 237 | 13462945 | 1.76 | 1.24 | (0.4, 3.9) | 1.18 | (0.37, 3.7) | 1.3 | (0.41, 4.07) |
| Anxiety | Easing 2 | Asian | 1095138 | 19 | 1166268 | 1.63 | 1.2 | (0.36, 4.07) | 1.21 | (0.36, 4.09) | 1.15 | (0.34, 3.91) |
| Anxiety | Easing 2 | Black | redacted |  |  |  |  |  |  |  |  |  |
| Anxiety | Easing 2 | Mixed | redacted |  |  |  |  |  |  |  |  |  |
| Anxiety | Easing 2 | Other | redacted |  |  |  |  |  |  |  |  |  |
| Anxiety | Wave 3 | White | 12602332 | 1185 | 83187132 | 1.42 | 1.15 | (0.69, 1.92) | 1.16 | (0.7, 1.93) | 1.25 | (0.75, 2.08) |
| Anxiety | Wave 3 | Asian | 1091692 | 94 | 7195031 | 1.31 | 1.19 | (0.69, 2.06) | 1.22 | (0.71, 2.11) | 1.17 | (0.68, 2.01) |
| Anxiety | Wave 3 | Black | 377686 | 21 | 2483064 | 0.85 | 0.72 | (0.37, 1.4) | 0.74 | (0.38, 1.44) | 0.68 | (0.35, 1.32) |
| Anxiety | Wave 3 | Mixed | 200227 | 15 | 1315159 | 1.14 | Reference |  | Reference |  | Reference |  |
| Anxiety | Wave 3 | Other | 314768 | 22 | 2054920 | 1.07 | 1.02 | (0.53, 1.96) | 1.04 | (0.54, 2) | 1.07 | (0.55, 2.05) |
| Anxiety | Easing 3 | White | 12411761 | 691 | 55915750 | 1.24 | 2.57 | (0.96, 6.87) | 2.57 | (0.96, 6.89) | 2.77 | (1.03, 7.42) |
| Anxiety | Easing 3 | Asian | 1074699 | 48 | 4838179 | 0.99 | 2.16 | (0.78, 5.98) | 2.19 | (0.79, 6.07) | 2.07 | (0.74, 5.74) |
| Anxiety | Easing 3 | Black | redacted |  |  |  |  |  |  |  |  |  |
| Anxiety | Easing 3 | Mixed | redacted |  |  |  |  |  |  |  |  |  |
| Anxiety | Easing 3 | Other | 304150 | 11 | 1360271 | 0.81 | 1.85 | (0.59, 5.82) | 1.88 | (0.6, 5.92) | 1.94 | (0.62, 6.09) |
| Depression | Pre | White | 11951915 | 2218 | 3E+08 | 0.74 | 0.93 | (0.67, 1.3) | 0.95 | (0.68, 1.32) | 1.08 | (0.77, 1.51) |
| Depression | Pre | Asian | 947198 | 83 | 23738551 | 0.35 | 0.46 | (0.31, 0.68) | 0.47 | (0.31, 0.69) | 0.44 | (0.29, 0.65) |
| Depression | Pre | Black | 323687 | 35 | 8111737 | 0.43 | 0.49 | (0.3, 0.78) | 0.5 | (0.31, 0.8) | 0.44 | (0.27, 0.7) |
| Depression | Pre | Mixed | 167862 | 35 | 4206541 | 0.83 | Reference |  | Reference |  | Reference |  |
| Depression | Pre | Other | 255875 | 23 | 6411754 | 0.36 | 0.49 | (0.29, 0.84) | 0.5 | (0.29, 0.84) | 0.52 | (0.31, 0.89) |
| Depression | Pandemic | White | 13007854 | 2181 | 3.22E+08 | 0.68 | 1.21 | (0.83, 1.77) | 1.12 | (0.76, 1.63) | 1.25 | (0.86, 1.84) |
| Depression | Pandemic | Asian | 1116587 | 97 | 27564956 | 0.35 | 0.76 | (0.5, 1.17) | 0.76 | (0.49, 1.16) | 0.71 | (0.47, 1.1) |
| Depression | Pandemic | Black | 389040 | 41 | 9540463 | 0.43 | 0.78 | (0.48, 1.27) | 0.79 | (0.48, 1.28) | 0.7 | (0.43, 1.13) |
| Depression | Pandemic | Mixed | 206060 | 27 | 5036547 | 0.54 | 1 |  | 1 |  | 1 |  |
| Depression | Pandemic | Other | 334275 | 29 | 8015812 | 0.36 | 0.77 | (0.46, 1.31) | 0.77 | (0.45, 1.3) | 0.8 | (0.47, 1.35) |
| Depression | Wave 1 | White | 13008078 | 185 | 29413291 | 0.63 | 3.9E+09 | (540000000, 28000000000) | 3.6E+09 | (500000000, 26000000000) | 4E+09 | (560000000, 29000000000) |
| Depression | Wave 1 | Asian | redacted |  |  |  |  |  |  |  |  |  |
| Depression | Wave 1 | Black | redacted |  |  |  |  |  |  |  |  |  |
| Depression | Wave 1 | Mixed | redacted |  |  |  |  |  |  |  |  |  |
| Depression | Wave 1 | Other | redacted |  |  |  |  |  |  |  |  |  |
| Depression | Easing 1 | White | 13059246 | 406 | 42520051 | 0.95 | 1.23 | (0.51, 2.97) | 1.17 | (0.48, 2.85) | 1.35 | (0.56, 3.27) |
| Depression | Easing 1 | Asian | 1128788 | 16 | 3675015 | 0.44 | 0.74 | (0.27, 2.03) | 0.75 | (0.27, 2.04) | 0.7 | (0.26, 1.91) |
| Depression | Easing 1 | Black | 393362 | 12 | 1279474 | 0.94 | 1.24 | (0.44, 3.51) | 1.25 | (0.44, 3.55) | 1.09 | (0.39, 3.11) |
| Depression | Easing 1 | Mixed | redacted |  |  |  |  |  |  |  |  |  |
| Depression | Easing 1 | Other | redacted |  |  |  |  |  |  |  |  |  |
| Depression | Wave 2 | White | 12967236 | 697 | 97844796 | 0.71 | 1.81 | (0.81, 4.04) | 1.68 | (0.75, 3.76) | 1.87 | (0.83, 4.19) |
| Depression | Wave 2 | Asian | 1122609 | 29 | 8461282 | 0.34 | 0.99 | (0.41, 2.4) | 0.99 | (0.41, 2.38) | 0.94 | (0.39, 2.26) |
| Depression | Wave 2 | Black | 390037 | 12 | 2935109 | 0.41 | 1.01 | (0.38, 2.68) | 1.02 | (0.38, 2.71) | 0.91 | (0.34, 2.42) |
| Depression | Wave 2 | Mixed | redacted |  |  |  |  |  |  |  |  |  |
| Depression | Wave 2 | Other | redacted |  |  |  |  |  |  |  |  |  |
| Depression | Easing 2 | White | 12640487 | 108 | 13463005 | 0.8 | 0.56 | (0.18, 1.78) | 0.5 | (0.16, 1.6) | 0.55 | (0.17, 1.74) |
| Depression | Easing 2 | Asian | redacted |  |  |  |  |  |  |  |  |  |
| Depression | Easing 2 | Black | redacted |  |  |  |  |  |  |  |  |  |
| Depression | Easing 2 | Mixed | redacted |  |  |  |  |  |  |  |  |  |
| Depression | Easing 2 | Other | redacted |  |  |  |  |  |  |  |  |  |
| Depression | Wave 3 | White | 12602338 | 504 | 83189402 | 0.61 | 1.89 | (0.71, 5.06) | 1.73 | (0.65, 4.65) | 1.96 | (0.73, 5.26) |
| Depression | Wave 3 | Asian | 1091692 | 26 | 7195242 | 0.36 | 1.41 | (0.49, 4.06) | 1.41 | (0.49, 4.05) | 1.32 | (0.46, 3.8) |
| Depression | Wave 3 | Black | redacted |  |  |  |  |  |  |  |  |  |
| Depression | Wave 3 | Mixed | redacted |  |  |  |  |  |  |  |  |  |
| Depression | Wave 3 | Other | redacted |  |  |  |  |  |  |  |  |  |
| Depression | Easing 3 | White | 12411765 | 310 | 55916565 | 0.55 | 0.81 | (0.36, 1.81) | 0.71 | (0.31, 1.6) | 0.8 | (0.35, 1.8) |
| Depression | Easing 3 | Asian | 1074699 | 15 | 4838248 | 0.31 | 0.48 | (0.19, 1.25) | 0.47 | (0.18, 1.23) | 0.44 | (0.17, 1.14) |
| Depression | Easing 3 | Black | redacted |  |  |  |  |  |  |  |  |  |
| Depression | Easing 3 | Mixed | redacted |  |  |  |  |  |  |  |  |  |
| Depression | Easing 3 | Other | redacted |  |  |  |  |  |  |  |  |  |
| Asthma | Pre | White | 1104943 | 14508 | 27504757 | 52.75 | 0.86 | (0.75, 0.98) | 0.86 | (0.75, 0.98) | 0.91 | (0.8, 1.04) |
| Asthma | Pre | Asian | 72238 | 1870 | 1785399 | 104.74 | 1.53 | (1.33, 1.76) | 1.57 | (1.37, 1.8) | 1.43 | (1.24, 1.64) |
| Asthma | Pre | Black | 21726 | 436 | 538585 | 80.95 | 1.21 | (1.03, 1.42) | 1.2 | (1.02, 1.41) | 1.06 | (0.9, 1.25) |
| Asthma | Pre | Mixed | 14090 | 227 | 350142 | 64.83 | Reference |  | Reference |  | Reference |  |
| Asthma | Pre | Other | 11220 | 176 | 278865 | 63.11 | 0.96 | (0.79, 1.17) | 0.97 | (0.8, 1.19) | 1.04 | (0.85, 1.27) |
| Asthma | Pandemic | White | 1230577 | 9804 | 30424471 | 32.22 | 0.92 | (0.78, 1.08) | 0.95 | (0.81, 1.11) | 1.01 | (0.86, 1.18) |
| Asthma | Pandemic | Asian | 84572 | 1128 | 2090544 | 53.96 | 1.39 | (1.17, 1.64) | 1.45 | (1.23, 1.72) | 1.34 | (1.13, 1.58) |
| Asthma | Pandemic | Black | 25738 | 279 | 632429 | 44.12 | 1.14 | (0.93, 1.38) | 1.15 | (0.94, 1.4) | 1.03 | (0.84, 1.25) |
| Asthma | Pandemic | Mixed | 17059 | 155 | 418542 | 37.03 | Reference |  | Reference |  | Reference |  |
| Asthma | Pandemic | Other | 13884 | 115 | 340269 | 33.8 | 0.91 | (0.71, 1.16) | 0.94 | (0.74, 1.2) | 0.99 | (0.78, 1.27) |
| Asthma | Wave 1 | White | 1230560 | 1011 | 2780998 | 36.35 | 1.08 | (0.61, 1.92) | 1.11 | (0.63, 1.96) | 1.17 | (0.66, 2.07) |
| Asthma | Wave 1 | Asian | 84566 | 68 | 191126 | 35.58 | 1.11 | (0.6, 2.05) | 1.14 | (0.62, 2.12) | 1.02 | (0.55, 1.88) |
| Asthma | Wave 1 | Black | 25739 | 18 | 58115 | 30.97 | 1.05 | (0.51, 2.18) | 1.04 | (0.5, 2.15) | 0.9 | (0.43, 1.86) |
| Asthma | Wave 1 | Mixed | 17045 | 12 | 38540 | 31.14 | Reference |  | Reference |  | Reference |  |
| Asthma | Wave 1 | Other | redacted |  |  |  |  |  |  |  |  |  |
| Asthma | Easing 1 | White | 1239227 | 1453 | 4033325 | 36.02 | 1.11 | (0.71, 1.76) | 1.16 | (0.73, 1.83) | 1.25 | (0.79, 1.96) |
| Asthma | Easing 1 | Asian | 85465 | 110 | 278243 | 39.53 | 1.18 | (0.72, 1.92) | 1.23 | (0.75, 2) | 1.09 | (0.67, 1.77) |
| Asthma | Easing 1 | Black | 25983 | 34 | 84514 | 40.23 | 1.17 | (0.67, 2.06) | 1.18 | (0.67, 2.07) | 1.02 | (0.58, 1.78) |
| Asthma | Easing 1 | Mixed | 17414 | 19 | 56614 | 33.56 | Reference |  | Reference |  | Reference |  |
| Asthma | Easing 1 | Other | 14127 | 16 | 45931 | 34.83 | 1.08 | (0.56, 2.11) | 1.11 | (0.57, 2.17) | 1.22 | (0.63, 2.38) |
| Asthma | Wave 2 | White | 1235344 | 3169 | 9316516 | 34.01 | 1.11 | (0.82, 1.51) | 1.19 | (0.88, 1.62) | 1.27 | (0.94, 1.73) |
| Asthma | Wave 2 | Asian | 85717 | 311 | 646184 | 48.13 | 1.43 | (1.04, 1.98) | 1.54 | (1.12, 2.13) | 1.39 | (1.01, 1.93) |
| Asthma | Wave 2 | Black | 25961 | 101 | 195315 | 51.71 | 1.55 | (1.08, 2.22) | 1.59 | (1.11, 2.29) | 1.4 | (0.97, 2) |
| Asthma | Wave 2 | Mixed | 17437 | 42 | 130946 | 32.07 | Reference |  | Reference |  | Reference |  |
| Asthma | Wave 2 | Other | 14058 | 33 | 105601 | 31.25 | 0.98 | (0.62, 1.55) | 1.03 | (0.65, 1.63) | 1.12 | (0.71, 1.77) |
| Asthma | Easing 2 | White | 1207813 | 677 | 1286418 | 52.63 | 1.06 | (0.56, 1.98) | 1.14 | (0.61, 2.15) | 1.21 | (0.65, 2.28) |
| Asthma | Easing 2 | Asian | 84468 | 84 | 89957 | 93.38 | 1.58 | (0.82, 3.04) | 1.69 | (0.88, 3.27) | 1.5 | (0.78, 2.89) |
| Asthma | Easing 2 | Black | 25562 | 20 | 27222 | 73.47 | 1.26 | (0.59, 2.69) | 1.3 | (0.61, 2.78) | 1.12 | (0.52, 2.4) |
| Asthma | Easing 2 | Mixed | redacted |  |  |  |  |  |  |  |  |  |
| Asthma | Easing 2 | Other | redacted |  |  |  |  |  |  |  |  |  |
| Asthma | Wave 3 | White | 1207064 | 3813 | 7964754 | 47.87 | 0.8 | (0.63, 1.01) | 0.85 | (0.67, 1.06) | 0.89 | (0.71, 1.12) |
| Asthma | Wave 3 | Asian | 84457 | 482 | 556459 | 86.62 | 1.21 | (0.95, 1.54) | 1.29 | (1.01, 1.65) | 1.18 | (0.93, 1.51) |
| Asthma | Wave 3 | Black | 25521 | 120 | 168043 | 71.41 | 1 | (0.75, 1.33) | 1.02 | (0.77, 1.37) | 0.92 | (0.69, 1.22) |
| Asthma | Wave 3 | Mixed | 17084 | 75 | 112349 | 66.76 | Reference |  | Reference |  | Reference |  |
| Asthma | Wave 3 | Other | 13693 | 52 | 89958 | 57.8 | 0.83 | (0.58, 1.19) | 0.87 | (0.61, 1.24) | 0.92 | (0.64, 1.31) |
| Asthma | Easing 3 | White | 1197790 | 2330 | 5394041 | 43.2 | 0.78 | (0.58, 1.05) | 0.8 | (0.59, 1.07) | 0.85 | (0.63, 1.14) |
| Asthma | Easing 3 | Asian | 84615 | 327 | 380842 | 85.86 | 1.33 | (0.97, 1.81) | 1.38 | (1.01, 1.87) | 1.27 | (0.93, 1.73) |
| Asthma | Easing 3 | Black | 25401 | 76 | 114225 | 66.54 | 1.03 | (0.71, 1.49) | 1.03 | (0.71, 1.48) | 0.92 | (0.64, 1.33) |
| Asthma | Easing 3 | Mixed | 16959 | 46 | 76258 | 60.32 | Reference |  | Reference |  | Reference |  |
| Asthma | Easing 3 | Other | 13585 | 35 | 61096 | 57.29 | 0.94 | (0.6, 1.46) | 0.96 | (0.62, 1.5) | 1.02 | (0.65, 1.58) |
| COPD | Pre | White | 409471 | 34264 | 9831716 | 348.5 | 1.09 | (0.9, 1.32) | 1.02 | (0.84, 1.23) | 1.04 | (0.86, 1.26) |
| COPD | Pre | Asian | 8290 | 531 | 200489 | 264.85 | 0.8 | (0.65, 0.98) | 0.78 | (0.64, 0.96) | 0.76 | (0.62, 0.93) |
| COPD | Pre | Black | 2556 | 144 | 62265 | 231.27 | 0.7 | (0.54, 0.89) | 0.69 | (0.54, 0.89) | 0.65 | (0.51, 0.84) |
| COPD | Pre | Mixed | 1379 | 107 | 33202 | 322.27 | Reference |  | Reference |  | Reference |  |
| COPD | Pre | Other | 1851 | 123 | 44939 | 273.7 | 0.85 | (0.66, 1.1) | 0.84 | (0.65, 1.08) | 0.85 | (0.65, 1.1) |
| COPD | Pandemic | White | 497200 | 33511 | 11478967 | 291.93 | 1.32 | (1.08, 1.62) | 1.2 | (0.98, 1.47) | 1.23 | (1, 1.51) |
| COPD | Pandemic | Asian | 10220 | 443 | 240147 | 184.47 | 0.81 | (0.65, 1.02) | 0.78 | (0.62, 0.97) | 0.74 | (0.59, 0.92) |
| COPD | Pandemic | Black | 3277 | 138 | 77025 | 179.16 | 0.79 | (0.61, 1.03) | 0.78 | (0.6, 1.01) | 0.71 | (0.55, 0.93) |
| COPD | Pandemic | Mixed | 1766 | 92 | 41773 | 220.24 | Reference |  | Reference |  | Reference |  |
| COPD | Pandemic | Other | 2405 | 97 | 56013 | 173.17 | 0.79 | (0.6, 1.06) | 0.76 | (0.57, 1.02) | 0.77 | (0.58, 1.02) |
| COPD | Wave 1 | White | 497196 | 3088 | 1114997 | 276.95 | 1.21 | (0.63, 2.32) | 1.12 | (0.58, 2.15) | 1.15 | (0.6, 2.21) |
| COPD | Wave 1 | Asian | 10221 | 32 | 22957 | 139.39 | 0.59 | (0.28, 1.23) | 0.56 | (0.27, 1.18) | 0.53 | (0.25, 1.1) |
| COPD | Wave 1 | Black | redacted |  |  |  |  |  |  |  |  |  |
| COPD | Wave 1 | Mixed | redacted |  |  |  |  |  |  |  |  |  |
| COPD | Wave 1 | Other | redacted |  |  |  |  |  |  |  |  |  |
| COPD | Easing 1 | White | 493737 | 5495 | 1591832 | 345.2 | 1.47 | (0.87, 2.48) | 1.35 | (0.8, 2.28) | 1.38 | (0.82, 2.34) |
| COPD | Easing 1 | Asian | 10226 | 64 | 33100 | 193.36 | 0.78 | (0.43, 1.38) | 0.74 | (0.42, 1.33) | 0.7 | (0.39, 1.25) |
| COPD | Easing 1 | Black | 3267 | 22 | 10548 | 208.57 | 0.82 | (0.42, 1.6) | 0.8 | (0.41, 1.57) | 0.74 | (0.38, 1.45) |
| COPD | Easing 1 | Mixed | 1768 | 14 | 5735 | 244.1 | Reference |  | Reference |  | Reference |  |
| COPD | Easing 1 | Other | 2407 | 16 | 7765 | 206.06 | 0.83 | (0.41, 1.71) | 0.8 | (0.39, 1.65) | 0.81 | (0.4, 1.66) |
| COPD | Wave 2 | White | 489153 | 10994 | 3607014 | 304.8 | 1.34 | (0.93, 1.91) | 1.21 | (0.85, 1.73) | 1.24 | (0.87, 1.78) |
| COPD | Wave 2 | Asian | 10158 | 146 | 75174 | 194.22 | 0.83 | (0.56, 1.23) | 0.79 | (0.53, 1.17) | 0.75 | (0.5, 1.11) |
| COPD | Wave 2 | Black | 3229 | 50 | 23911 | 209.11 | 0.9 | (0.57, 1.42) | 0.88 | (0.56, 1.38) | 0.8 | (0.51, 1.26) |
| COPD | Wave 2 | Mixed | 1784 | 30 | 13200 | 227.26 | Reference |  | Reference |  | Reference |  |
| COPD | Wave 2 | Other | 2372 | 32 | 17549 | 182.35 | 0.81 | (0.49, 1.33) | 0.78 | (0.47, 1.28) | 0.78 | (0.47, 1.28) |
| COPD | Easing 2 | White | 478271 | 2532 | 507374 | 499.04 | 1.19 | (0.59, 2.39) | 1.07 | (0.53, 2.14) | 1.09 | (0.54, 2.18) |
| COPD | Easing 2 | Asian | 10036 | 30 | 10656 | 281.52 | 0.67 | (0.31, 1.45) | 0.64 | (0.29, 1.4) | 0.6 | (0.27, 1.3) |
| COPD | Easing 2 | Black | 3242 | 11 | 3446 | 319.17 | 0.76 | (0.3, 1.89) | 0.74 | (0.3, 1.85) | 0.68 | (0.27, 1.69) |
| COPD | Easing 2 | Mixed | redacted |  |  |  |  |  |  |  |  |  |
| COPD | Easing 2 | Other | redacted |  |  |  |  |  |  |  |  |  |
| COPD | Wave 3 | White | 477395 | 12942 | 3083669 | 419.69 | 1.63 | (1.14, 2.33) | 1.48 | (1.03, 2.12) | 1.51 | (1.06, 2.17) |
| COPD | Wave 3 | Asian | 10023 | 181 | 65161 | 277.77 | 1.04 | (0.71, 1.53) | 1.01 | (0.69, 1.49) | 0.95 | (0.65, 1.4) |
| COPD | Wave 3 | Black | 3229 | 51 | 21022 | 242.61 | 0.93 | (0.59, 1.46) | 0.91 | (0.58, 1.43) | 0.83 | (0.53, 1.31) |
| COPD | Wave 3 | Mixed | 1809 | 30 | 11751 | 255.29 | Reference |  | Reference |  | Reference |  |
| COPD | Wave 3 | Other | 2422 | 42 | 15769 | 266.35 | 1.06 | (0.66, 1.69) | 1.03 | (0.64, 1.64) | 1.04 | (0.65, 1.66) |
| COPD | Easing 3 | White | 472883 | 9016 | 2093051 | 430.76 | 1.12 | (0.79, 1.6) | 1 | (0.7, 1.42) | 1.02 | (0.72, 1.45) |
| COPD | Easing 3 | Asian | 9987 | 132 | 44390 | 297.37 | 0.75 | (0.51, 1.11) | 0.71 | (0.48, 1.05) | 0.67 | (0.45, 0.98) |
| COPD | Easing 3 | Black | 3230 | 41 | 14355 | 285.62 | 0.74 | (0.46, 1.18) | 0.71 | (0.45, 1.14) | 0.65 | (0.41, 1.04) |
| COPD | Easing 3 | Mixed | 1849 | 31 | 8222 | 377.01 | Reference |  | Reference |  | Reference |  |
| COPD | Easing 3 | Other | 2428 | 31 | 10808 | 286.83 | 0.78 | (0.47, 1.28) | 0.74 | (0.45, 1.22) | 0.75 | (0.46, 1.24) |

### Table 7: Number of events, rate, hazard ratio and confidence intervals by ethnic group, time period and outcome with those of Other ethnicity as the reference group.

| **Outcome** | **Period** | **Ethnicity** | **Denominator** | **events** | **Total person months** | **Rate per month** | **Unadjusted** | | **Age and sex adjusted** | | **Fully adjusted*** | |
| --- | --- | --- | --- | --- | --- | --- | --- | --- | --- | --- | --- | --- |
|  |  |  |  |  |  |  | **Hazard ratio** | **95% confidence interval** | **Hazard ratio** | **95% confidence interval** | **Hazard ratio** | **95% confidence interval** |
| Stroke | Pre | White | 11951861 | 42033 | 299064047 | 14.05 | 2.58 | (2.31, 2.89) | 1.29 | (1.15, 1.44) | 1.34 | (1.19, 1.5) |
| Stroke | Pre | Asian | 947198 | 1835 | 23717609 | 7.74 | 1.6 | (1.42, 1.81) | 1.4 | (1.24, 1.57) | 1.29 | (1.14, 1.45) |
| Stroke | Pre | Black | 323687 | 683 | 8104085 | 8.43 | 1.76 | (1.54, 2.01) | 1.49 | (1.31, 1.71) | 1.31 | (1.15, 1.5) |
| Stroke | Pre | Mixed | 167862 | 249 | 4204083 | 5.92 | 1.19 | (1.01, 1.4) | 1.35 | (1.15, 1.6) | 1.29 | (1.09, 1.53) |
| Stroke | Pre | Other | 255875 | 311 | 6408427 | 4.85 | Reference |  | Reference |  | Reference |  |
| Stroke | Pandemic | White | 13007778 | 62025 | 321371341 | 19.3 | 2.83 | (2.59, 3.1) | 1.3 | (1.19, 1.42) | 1.35 | (1.24, 1.48) |
| Stroke | Pandemic | Asian | 1116583 | 2669 | 27539949 | 9.69 | 1.6 | (1.45, 1.76) | 1.33 | (1.21, 1.46) | 1.24 | (1.13, 1.37) |
| Stroke | Pandemic | Black | 389039 | 1107 | 9530751 | 11.62 | 1.93 | (1.73, 2.15) | 1.54 | (1.39, 1.72) | 1.38 | (1.24, 1.54) |
| Stroke | Pandemic | Mixed | 206060 | 357 | 5033434 | 7.09 | 1.13 | (0.99, 1.3) | 1.26 | (1.1, 1.45) | 1.22 | (1.06, 1.4) |
| Stroke | Pandemic | Other | 334274 | 485 | 8011353 | 6.05 | Reference |  | Reference |  | Reference |  |
| Stroke | Wave 1 | White | 13008002 | 4886 | 29408734 | 16.61 | 3.24 | (2.32, 4.52) | 1.44 | (1.03, 2.01) | 1.51 | (1.08, 2.11) |
| Stroke | Wave 1 | Asian | 1116586 | 210 | 2525075 | 8.32 | 1.81 | (1.26, 2.59) | 1.46 | (1.02, 2.09) | 1.36 | (0.95, 1.94) |
| Stroke | Wave 1 | Black | 389021 | 79 | 879114 | 8.99 | 2.01 | (1.35, 2.99) | 1.56 | (1.04, 2.32) | 1.38 | (0.93, 2.06) |
| Stroke | Wave 1 | Mixed | 206091 | 22 | 465920 | 4.72 | 1 | (0.59, 1.7) | 1.1 | (0.65, 1.88) | 1.06 | (0.62, 1.81) |
| Stroke | Wave 1 | Other | 334305 | 35 | 754927 | 4.64 | Reference |  | Reference |  | Reference |  |
| Stroke | Easing 1 | White | 13059172 | 8104 | 42509205 | 19.06 | 2.78 | (2.18, 3.55) | 1.23 | (0.97, 1.57) | 1.29 | (1.01, 1.65) |
| Stroke | Easing 1 | Asian | 1128784 | 343 | 3674505 | 9.33 | 1.54 | (1.18, 2.01) | 1.25 | (0.96, 1.63) | 1.17 | (0.9, 1.53) |
| Stroke | Easing 1 | Black | 393360 | 143 | 1279287 | 11.18 | 1.87 | (1.39, 2.5) | 1.47 | (1.09, 1.96) | 1.31 | (0.98, 1.76) |
| Stroke | Easing 1 | Mixed | 208793 | 49 | 678885 | 7.22 | 1.15 | (0.8, 1.67) | 1.27 | (0.88, 1.84) | 1.23 | (0.85, 1.78) |
| Stroke | Easing 1 | Other | 338288 | 66 | 1098220 | 6.01 | Reference |  | Reference |  | Reference |  |
| Stroke | Wave 2 | White | 12967133 | 19186 | 97788237 | 19.62 | 2.78 | (2.37, 3.25) | 1.25 | (1.07, 1.47) | 1.31 | (1.12, 1.53) |
| Stroke | Wave 2 | Asian | 1122606 | 839 | 8458675 | 9.92 | 1.57 | (1.33, 1.87) | 1.3 | (1.09, 1.54) | 1.22 | (1.02, 1.44) |
| Stroke | Wave 2 | Black | 390035 | 338 | 2934168 | 11.52 | 1.84 | (1.52, 2.22) | 1.46 | (1.2, 1.76) | 1.3 | (1.08, 1.58) |
| Stroke | Wave 2 | Mixed | 207204 | 115 | 1555135 | 7.39 | 1.14 | (0.9, 1.45) | 1.27 | (1, 1.62) | 1.23 | (0.96, 1.56) |
| Stroke | Wave 2 | Other | 332901 | 156 | 2485211 | 6.28 | Reference |  | Reference |  | Reference |  |
| Stroke | Easing 2 | White | 12640411 | 2984 | 13461555 | 22.17 | 2.9 | (1.92, 4.37) | 1.34 | (0.89, 2.03) | 1.4 | (0.93, 2.12) |
| Stroke | Easing 2 | Asian | 1095134 | 114 | 1166215 | 9.78 | 1.45 | (0.93, 2.28) | 1.21 | (0.77, 1.89) | 1.12 | (0.72, 1.76) |
| Stroke | Easing 2 | Black | 379052 | 59 | 403523 | 14.62 | 2.21 | (1.37, 3.59) | 1.78 | (1.1, 2.88) | 1.56 | (0.97, 2.54) |
| Stroke | Easing 2 | Mixed | 201010 | 12 | 213928 | 5.61 | 0.81 | (0.4, 1.63) | 0.91 | (0.45, 1.82) | 0.87 | (0.43, 1.75) |
| Stroke | Easing 2 | Other | 317013 | 23 | 337061 | 6.82 | Reference |  | Reference |  | Reference |  |
| Stroke | Wave 3 | White | 12602238 | 16633 | 83144516 | 20 | 2.47 | (2.1, 2.9) | 1.15 | (0.98, 1.36) | 1.21 | (1.02, 1.42) |
| Stroke | Wave 3 | Asian | 1091687 | 756 | 7193014 | 10.51 | 1.46 | (1.23, 1.75) | 1.23 | (1.03, 1.47) | 1.15 | (0.97, 1.38) |
| Stroke | Wave 3 | Black | 377684 | 312 | 2482157 | 12.57 | 1.75 | (1.44, 2.13) | 1.41 | (1.16, 1.72) | 1.26 | (1.03, 1.53) |
| Stroke | Wave 3 | Mixed | 200227 | 98 | 1314892 | 7.45 | 1 | (0.78, 1.29) | 1.12 | (0.87, 1.45) | 1.08 | (0.84, 1.4) |
| Stroke | Wave 3 | Other | 314766 | 148 | 2054547 | 7.2 | Reference |  | Reference |  | Reference |  |
| Stroke | Easing 3 | White | 12411670 | 11376 | 55895206 | 20.35 | 3.24 | (2.59, 4.07) | 1.5 | (1.2, 1.88) | 1.56 | (1.24, 1.96) |
| Stroke | Easing 3 | Asian | 1074695 | 487 | 4837290 | 10.07 | 1.8 | (1.42, 2.3) | 1.53 | (1.2, 1.95) | 1.44 | (1.13, 1.84) |
| Stroke | Easing 3 | Black | 369667 | 210 | 1661122 | 12.64 | 2.28 | (1.75, 2.96) | 1.84 | (1.41, 2.39) | 1.66 | (1.28, 2.16) |
| Stroke | Easing 3 | Mixed | 197112 | 71 | 885370 | 8.020001 | 1.39 | (1.01, 1.92) | 1.58 | (1.14, 2.18) | 1.52 | (1.1, 2.11) |
| Stroke | Easing 3 | Other | 304150 | 76 | 1360142 | 5.59 | Reference |  | Reference |  | Reference |  |
| VTE | Pre | White | 11951881 | 28859 | 299215565 | 9.64 | 3.12 | (2.69, 3.61) | 2.2 | (1.9, 2.55) | 2.27 | (1.96, 2.63) |
| VTE | Pre | Asian | 947196 | 752 | 23730820 | 3.17 | 1.13 | (0.96, 1.33) | 1.07 | (0.91, 1.26) | 0.95 | (0.81, 1.12) |
| VTE | Pre | Black | 323685 | 501 | 8106277 | 6.18 | 2.19 | (1.85, 2.59) | 2.01 | (1.69, 2.38) | 1.69 | (1.42, 2) |
| VTE | Pre | Mixed | 167862 | 195 | 4204866 | 4.64 | 1.6 | (1.3, 1.95) | 1.71 | (1.39, 2.09) | 1.6 | (1.31, 1.96) |
| VTE | Pre | Other | 255875 | 181 | 6409861 | 2.82 | Reference |  | Reference |  | Reference |  |
| VTE | Pandemic | White | 13007823 | 38710 | 321532860 | 12.04 | 3.22 | (2.86, 3.63) | 2.15 | (1.91, 2.42) | 2.23 | (1.97, 2.51) |
| VTE | Pandemic | Asian | 1116586 | 939 | 27555834 | 3.41 | 1.03 | (0.9, 1.18) | 0.93 | (0.82, 1.07) | 0.84 | (0.73, 0.96) |
| VTE | Pandemic | Black | 389040 | 785 | 9532588 | 8.23 | 2.41 | (2.1, 2.76) | 2.13 | (1.86, 2.45) | 1.81 | (1.58, 2.08) |
| VTE | Pandemic | Mixed | 206060 | 290 | 5033737 | 5.76 | 1.64 | (1.39, 1.94) | 1.74 | (1.48, 2.06) | 1.64 | (1.39, 1.94) |
| VTE | Pandemic | Other | 334273 | 275 | 8013201 | 3.43 | Reference |  | Reference |  | Reference |  |
| VTE | Wave 1 | White | 13008047 | 2634 | 29410861 | 8.96 | 3.69 | (2.29, 5.95) | 2.43 | (1.51, 3.93) | 2.51 | (1.55, 4.05) |
| VTE | Wave 1 | Asian | 1116589 | 59 | 2525212 | 2.34 | 1.04 | (0.6, 1.78) | 0.92 | (0.54, 1.59) | 0.81 | (0.47, 1.39) |
| VTE | Wave 1 | Black | 389022 | 65 | 879116 | 7.39 | 3.28 | (1.92, 5.59) | 2.85 | (1.67, 4.87) | 2.32 | (1.36, 3.97) |
| VTE | Wave 1 | Mixed | 206091 | 17 | 465927 | 3.65 | 1.58 | (0.81, 3.1) | 1.68 | (0.86, 3.29) | 1.55 | (0.79, 3.04) |
| VTE | Wave 1 | Other | 334304 | 17 | 754936 | 2.25 | Reference |  | Reference |  | Reference |  |
| VTE | Easing 1 | White | 13059222 | 5157 | 42512892 | 12.13 | 3.15 | (2.3, 4.32) | 2.11 | (1.54, 2.9) | 2.19 | (1.59, 3.01) |
| VTE | Easing 1 | Asian | 1128788 | 116 | 3674863 | 3.16 | 0.92 | (0.64, 1.33) | 0.83 | (0.58, 1.2) | 0.73 | (0.51, 1.06) |
| VTE | Easing 1 | Black | 393361 | 93 | 1279337 | 7.27 | 2.07 | (1.43, 3.02) | 1.82 | (1.25, 2.64) | 1.5 | (1.03, 2.18) |
| VTE | Easing 1 | Mixed | 208793 | 36 | 678890 | 5.3 | 1.46 | (0.93, 2.3) | 1.55 | (0.99, 2.44) | 1.45 | (0.92, 2.28) |
| VTE | Easing 1 | Other | 338288 | 39 | 1098249 | 3.55 | Reference |  | Reference |  | Reference |  |
| VTE | Wave 2 | White | 12967150 | 12627 | 97803435 | 12.91 | 3.62 | (2.9, 4.51) | 2.39 | (1.91, 2.98) | 2.47 | (1.98, 3.08) |
| VTE | Wave 2 | Asian | 1122608 | 324 | 8460268 | 3.83 | 1.23 | (0.96, 1.57) | 1.11 | (0.87, 1.42) | 1 | (0.78, 1.28) |
| VTE | Wave 2 | Black | 390036 | 253 | 2934299 | 8.62 | 2.67 | (2.07, 3.43) | 2.35 | (1.83, 3.02) | 1.98 | (1.54, 2.55) |
| VTE | Wave 2 | Mixed | 207205 | 101 | 1555115 | 6.49 | 1.96 | (1.46, 2.62) | 2.08 | (1.55, 2.79) | 1.96 | (1.46, 2.63) |
| VTE | Wave 2 | Other | 332902 | 80 | 2485458 | 3.22 | Reference |  | Reference |  | Reference |  |
| VTE | Easing 2 | White | 12640457 | 1801 | 13462095 | 13.38 | 3.5 | (1.98, 6.18) | 2.3 | (1.3, 4.07) | 2.37 | (1.34, 4.2) |
| VTE | Easing 2 | Asian | 1095139 | 41 | 1166258 | 3.52 | 1.01 | (0.53, 1.92) | 0.91 | (0.48, 1.74) | 0.81 | (0.42, 1.54) |
| VTE | Easing 2 | Black | 379052 | 38 | 403537 | 9.42 | 2.66 | (1.39, 5.1) | 2.35 | (1.23, 4.5) | 1.94 | (1.01, 3.72) |
| VTE | Easing 2 | Mixed | 201011 | 14 | 213929 | 6.54 | 1.82 | (0.84, 3.93) | 1.94 | (0.9, 4.19) | 1.81 | (0.84, 3.91) |
| VTE | Easing 2 | Other | 317012 | 12 | 337066 | 3.56 | Reference |  | Reference |  | Reference |  |
| VTE | Wave 3 | White | 12602274 | 10091 | 83160465 | 12.13 | 2.89 | (2.31, 3.6) | 1.96 | (1.57, 2.45) | 2.04 | (1.64, 2.55) |
| VTE | Wave 3 | Asian | 1091690 | 249 | 7194558 | 3.46 | 0.94 | (0.73, 1.21) | 0.86 | (0.67, 1.11) | 0.77 | (0.6, 0.99) |
| VTE | Wave 3 | Black | 377684 | 202 | 2482470 | 8.14 | 2.12 | (1.64, 2.75) | 1.9 | (1.46, 2.46) | 1.6 | (1.24, 2.08) |
| VTE | Wave 3 | Mixed | 200227 | 80 | 1314939 | 6.08 | 1.55 | (1.13, 2.11) | 1.64 | (1.2, 2.23) | 1.55 | (1.14, 2.11) |
| VTE | Wave 3 | Other | 314767 | 80 | 2054751 | 3.89 | Reference |  | Reference |  | Reference |  |
| VTE | Easing 3 | White | 12411687 | 6834 | 55902234 | 12.22 | 3.71 | (2.73, 5.05) | 2.5 | (1.83, 3.4) | 2.59 | (1.9, 3.52) |
| VTE | Easing 3 | Asian | 1074698 | 184 | 4837902 | 3.8 | 1.32 | (0.94, 1.85) | 1.21 | (0.86, 1.7) | 1.12 | (0.8, 1.57) |
| VTE | Easing 3 | Black | 369670 | 136 | 1661279 | 8.19 | 2.72 | (1.92, 3.86) | 2.44 | (1.72, 3.46) | 2.14 | (1.51, 3.04) |
| VTE | Easing 3 | Mixed | 197112 | 46 | 885409 | 5.2 | 1.69 | (1.11, 2.58) | 1.81 | (1.18, 2.75) | 1.73 | (1.14, 2.64) |
| VTE | Easing 3 | Other | 304148 | 41 | 1360203 | 3.01 | Reference |  | Reference |  | Reference |  |
| Heart failure | Pre | White | 11951886 | 31037 | 299209045 | 10.37 | 2.89 | (2.54, 3.3) | 1.2 | (1.05, 1.37) | 1.3 | (1.14, 1.49) |
| Heart failure | Pre | Asian | 947193 | 1925 | 23716286 | 8.12 | 2.25 | (1.96, 2.59) | 1.87 | (1.63, 2.15) | 1.59 | (1.38, 1.83) |
| Heart failure | Pre | Black | 323687 | 597 | 8104779 | 7.37 | 2.06 | (1.77, 2.41) | 1.69 | (1.45, 1.97) | 1.29 | (1.11, 1.51) |
| Heart failure | Pre | Mixed | 167861 | 166 | 4204968 | 3.95 | 1.12 | (0.91, 1.36) | 1.32 | (1.08, 1.62) | 1.18 | (0.96, 1.44) |
| Heart failure | Pre | Other | 255875 | 222 | 6409411 | 3.46 | Reference |  | Reference |  | Reference |  |
| Heart failure | Pandemic | White | 13007814 | 50729 | 321522961 | 15.78 | 3.28 | (2.96, 3.64) | 1.24 | (1.12, 1.37) | 1.35 | (1.22, 1.5) |
| Heart failure | Pandemic | Asian | 1116584 | 2508 | 27542692 | 9.11 | 1.95 | (1.74, 2.18) | 1.53 | (1.37, 1.71) | 1.33 | (1.19, 1.49) |
| Heart failure | Pandemic | Black | 389038 | 842 | 9532933 | 8.83 | 1.9 | (1.68, 2.15) | 1.44 | (1.27, 1.63) | 1.14 | (1.01, 1.29) |
| Heart failure | Pandemic | Mixed | 206060 | 284 | 5034330 | 5.64 | 1.21 | (1.04, 1.42) | 1.4 | (1.19, 1.63) | 1.27 | (1.09, 1.48) |
| Heart failure | Pandemic | Other | 334275 | 362 | 8013004 | 4.52 | Reference |  | Reference |  | Reference |  |
| Heart failure | Wave 1 | White | 13008038 | 3540 | 29410481 | 12.04 | 3.27 | (2.22, 4.82) | 1.2 | (0.81, 1.77) | 1.32 | (0.89, 1.94) |
| Heart failure | Wave 1 | Asian | 1116587 | 185 | 2525095 | 7.33 | 2.07 | (1.37, 3.13) | 1.58 | (1.05, 2.39) | 1.37 | (0.91, 2.07) |
| Heart failure | Wave 1 | Black | 389020 | 65 | 879134 | 7.39 | 2.14 | (1.36, 3.37) | 1.57 | (0.99, 2.47) | 1.24 | (0.78, 1.95) |
| Heart failure | Wave 1 | Mixed | 206091 | 17 | 465929 | 3.65 | 1.03 | (0.56, 1.91) | 1.18 | (0.64, 2.17) | 1.07 | (0.58, 1.97) |
| Heart failure | Wave 1 | Other | 334306 | 27 | 754939 | 3.58 | Reference |  | Reference |  | Reference |  |
| Heart failure | Easing 1 | White | 13059194 | 6970 | 42510238 | 16.4 | 4.57 | (3.29, 6.35) | 1.68 | (1.21, 2.33) | 1.85 | (1.33, 2.57) |
| Heart failure | Easing 1 | Asian | 1128784 | 376 | 3674476 | 10.23 | 3.03 | (2.15, 4.26) | 2.34 | (1.66, 3.3) | 2.01 | (1.42, 2.83) |
| Heart failure | Easing 1 | Black | 393360 | 121 | 1279279 | 9.46 | 2.78 | (1.91, 4.03) | 2.07 | (1.42, 3) | 1.6 | (1.1, 2.32) |
| Heart failure | Easing 1 | Mixed | 208793 | 38 | 678881 | 5.6 | 1.64 | (1.04, 2.58) | 1.87 | (1.18, 2.94) | 1.68 | (1.06, 2.65) |
| Heart failure | Easing 1 | Other | 338286 | 37 | 1098249 | 3.37 | Reference |  | Reference |  | Reference |  |
| Heart failure | Wave 2 | White | 12967138 | 16378 | 97799415 | 16.75 | 2.99 | (2.52, 3.55) | 1.11 | (0.93, 1.32) | 1.22 | (1.03, 1.45) |
| Heart failure | Wave 2 | Asian | 1122605 | 844 | 8458766 | 9.98 | 1.83 | (1.52, 2.2) | 1.42 | (1.18, 1.71) | 1.23 | (1.02, 1.47) |
| Heart failure | Wave 2 | Black | 390036 | 311 | 2934208 | 10.6 | 1.97 | (1.6, 2.41) | 1.47 | (1.2, 1.8) | 1.15 | (0.94, 1.42) |
| Heart failure | Wave 2 | Mixed | 207204 | 89 | 1555247 | 5.72 | 1.05 | (0.8, 1.38) | 1.2 | (0.92, 1.57) | 1.09 | (0.83, 1.43) |
| Heart failure | Wave 2 | Other | 332902 | 131 | 2485311 | 5.27 | Reference |  | Reference |  | Reference |  |
| Heart failure | Easing 2 | White | 12640422 | 3464 | 13461250 | 25.73 | 2.71 | (1.89, 3.89) | 0.98 | (0.68, 1.4) | 1.09 | (0.76, 1.57) |
| Heart failure | Easing 2 | Asian | 1095135 | 199 | 1166170 | 17.06 | 1.87 | (1.27, 2.74) | 1.45 | (0.99, 2.14) | 1.26 | (0.86, 1.85) |
| Heart failure | Easing 2 | Black | 379052 | 52 | 403527 | 12.89 | 1.41 | (0.9, 2.21) | 1.05 | (0.67, 1.65) | 0.82 | (0.52, 1.28) |
| Heart failure | Easing 2 | Mixed | 201011 | 19 | 213926 | 8.88 | 0.97 | (0.54, 1.72) | 1.11 | (0.63, 1.98) | 1.01 | (0.57, 1.8) |
| Heart failure | Easing 2 | Other | 317012 | 30 | 337056 | 8.9 | Reference |  | Reference |  | Reference |  |
| Heart failure | Wave 3 | White | 12602204 | 14580 | 83149534 | 17.53 | 2.94 | (2.46, 3.53) | 1.13 | (0.94, 1.36) | 1.25 | (1.04, 1.5) |
| Heart failure | Wave 3 | Asian | 1091682 | 767 | 7192954 | 10.66 | 1.78 | (1.47, 2.16) | 1.41 | (1.16, 1.71) | 1.23 | (1.01, 1.49) |
| Heart failure | Wave 3 | Black | 377683 | 239 | 2482401 | 9.63 | 1.62 | (1.3, 2.01) | 1.22 | (0.98, 1.52) | 0.95 | (0.77, 1.19) |
| Heart failure | Wave 3 | Mixed | 200224 | 87 | 1314956 | 6.62 | 1.12 | (0.85, 1.47) | 1.29 | (0.98, 1.7) | 1.17 | (0.89, 1.55) |
| Heart failure | Wave 3 | Other | 314766 | 119 | 2054623 | 5.79 | Reference |  | Reference |  | Reference |  |
| Heart failure | Easing 3 | White | 12411683 | 11000 | 55896009 | 19.68 | 3.69 | (2.91, 4.68) | 1.42 | (1.12, 1.81) | 1.57 | (1.23, 1.99) |
| Heart failure | Easing 3 | Asian | 1074692 | 571 | 4837135 | 11.8 | 2.24 | (1.75, 2.88) | 1.82 | (1.41, 2.33) | 1.6 | (1.24, 2.05) |
| Heart failure | Easing 3 | Black | 369669 | 187 | 1661225 | 11.26 | 2.15 | (1.63, 2.83) | 1.65 | (1.25, 2.17) | 1.32 | (1, 1.74) |
| Heart failure | Easing 3 | Mixed | 197111 | 65 | 885385 | 7.34 | 1.41 | (1, 1.97) | 1.65 | (1.17, 2.31) | 1.52 | (1.08, 2.13) |
| Heart failure | Easing 3 | Other | 304148 | 69 | 1360166 | 5.07 | Reference |  | Reference |  | Reference |  |
| MI | Pre | White | 11951868 | 41681 | 299044045 | 13.94 | 2.42 | (2.17, 2.7) | 1.33 | (1.2, 1.49) | 1.4 | (1.26, 1.57) |
| MI | Pre | Asian | 947193 | 3206 | 23700749 | 13.53 | 2.56 | (2.28, 2.87) | 2.24 | (2, 2.51) | 2.06 | (1.83, 2.3) |
| MI | Pre | Black | 323687 | 425 | 8107170 | 5.24 | 1.01 | (0.88, 1.17) | 0.84 | (0.73, 0.97) | 0.73 | (0.63, 0.84) |
| MI | Pre | Mixed | 167862 | 212 | 4204384 | 5.04 | 0.95 | (0.8, 1.13) | 1.07 | (0.9, 1.27) | 1.02 | (0.85, 1.21) |
| MI | Pre | Other | 255872 | 329 | 6407889 | 5.13 | Reference |  | Reference |  | Reference |  |
| MI | Pandemic | White | 13007792 | 49713 | 321413154 | 15.47 | 2.28 | (2.09, 2.5) | 1.21 | (1.1, 1.32) | 1.27 | (1.16, 1.39) |
| MI | Pandemic | Asian | 1116586 | 3864 | 27525109 | 14.04 | 2.26 | (2.06, 2.49) | 1.91 | (1.74, 2.1) | 1.78 | (1.61, 1.95) |
| MI | Pandemic | Black | 389038 | 603 | 9534880 | 6.32 | 1.05 | (0.93, 1.18) | 0.84 | (0.75, 0.95) | 0.75 | (0.66, 0.84) |
| MI | Pandemic | Mixed | 206059 | 252 | 5033918 | 5.01 | 0.81 | (0.69, 0.94) | 0.89 | (0.76, 1.03) | 0.85 | (0.73, 1) |
| MI | Pandemic | Other | 334275 | 480 | 8011100 | 5.99 | Reference |  | Reference |  | Reference |  |
| MI | Wave 1 | White | 13008016 | 3585 | 29409854 | 12.19 | 4.08 | (2.63, 6.34) | 2.14 | (1.37, 3.32) | 2.27 | (1.46, 3.53) |
| MI | Wave 1 | Asian | 1116589 | 271 | 2525011 | 10.73 | 3.91 | (2.48, 6.17) | 3.22 | (2.04, 5.08) | 2.98 | (1.89, 4.7) |
| MI | Wave 1 | Black | 389020 | 33 | 879155 | 3.75 | 1.43 | (0.82, 2.48) | 1.11 | (0.64, 1.94) | 0.98 | (0.56, 1.7) |
| MI | Wave 1 | Mixed | 206090 | 17 | 465920 | 3.65 | 1.33 | (0.7, 2.54) | 1.44 | (0.76, 2.76) | 1.39 | (0.73, 2.65) |
| MI | Wave 1 | Other | 334306 | 21 | 754936 | 2.78 | Reference |  | Reference |  | Reference |  |
| MI | Easing 1 | White | 13059185 | 6342 | 42511189 | 14.92 | 2.15 | (1.68, 2.74) | 1.08 | (0.85, 1.39) | 1.15 | (0.9, 1.47) |
| MI | Easing 1 | Asian | 1128784 | 459 | 3674351 | 12.49 | 2.04 | (1.58, 2.64) | 1.68 | (1.3, 2.18) | 1.55 | (1.2, 2.01) |
| MI | Easing 1 | Black | 393362 | 71 | 1279392 | 5.55 | 0.93 | (0.66, 1.3) | 0.73 | (0.52, 1.02) | 0.64 | (0.46, 0.9) |
| MI | Easing 1 | Mixed | 208793 | 31 | 678906 | 4.57 | 0.73 | (0.48, 1.12) | 0.8 | (0.52, 1.22) | 0.77 | (0.5, 1.17) |
| MI | Easing 1 | Other | 338288 | 66 | 1098217 | 6.01 | Reference |  | Reference |  | Reference |  |
| MI | Wave 2 | White | 12967152 | 15520 | 97794466 | 15.87 | 2.25 | (1.92, 2.64) | 1.16 | (0.99, 1.36) | 1.22 | (1.04, 1.43) |
| MI | Wave 2 | Asian | 1122602 | 1172 | 8457313 | 13.86 | 2.19 | (1.85, 2.6) | 1.83 | (1.54, 2.16) | 1.69 | (1.43, 2.01) |
| MI | Wave 2 | Black | 390035 | 182 | 2934586 | 6.2 | 1.01 | (0.82, 1.26) | 0.81 | (0.65, 1) | 0.72 | (0.58, 0.89) |
| MI | Wave 2 | Mixed | 207205 | 81 | 1555226 | 5.21 | 0.82 | (0.62, 1.07) | 0.9 | (0.69, 1.18) | 0.87 | (0.66, 1.13) |
| MI | Wave 2 | Other | 332901 | 152 | 2485197 | 6.12 | Reference |  | Reference |  | Reference |  |
| MI | Easing 2 | White | 12640414 | 2489 | 13461750 | 18.49 | 2.9 | (1.85, 4.57) | 1.49 | (0.94, 2.34) | 1.55 | (0.99, 2.45) |
| MI | Easing 2 | Asian | 1095133 | 172 | 1166183 | 14.75 | 2.55 | (1.58, 4.1) | 2.14 | (1.33, 3.44) | 2 | (1.25, 3.22) |
| MI | Easing 2 | Black | 379051 | 39 | 403537 | 9.66 | 1.74 | (1, 3.01) | 1.4 | (0.81, 2.42) | 1.26 | (0.72, 2.17) |
| MI | Easing 2 | Mixed | redacted |  |  |  |  |  |  |  |  |  |
| MI | Easing 2 | Other | 317013 | 19 | 337062 | 5.64 | Reference |  | Reference |  | Reference |  |
| MI | Wave 3 | White | 12602261 | 13201 | 83150575 | 15.88 | 2.18 | (1.84, 2.59) | 1.16 | (0.98, 1.38) | 1.23 | (1.03, 1.46) |
| MI | Wave 3 | Asian | 1091685 | 1085 | 7191830 | 15.09 | 2.22 | (1.86, 2.66) | 1.9 | (1.58, 2.27) | 1.76 | (1.47, 2.11) |
| MI | Wave 3 | Black | 377686 | 169 | 2482621 | 6.81 | 1.03 | (0.82, 1.29) | 0.83 | (0.66, 1.05) | 0.74 | (0.59, 0.93) |
| MI | Wave 3 | Mixed | 200225 | 60 | 1314987 | 4.56 | 0.68 | (0.5, 0.92) | 0.75 | (0.55, 1.02) | 0.73 | (0.54, 0.99) |
| MI | Wave 3 | Other | 314768 | 133 | 2054569 | 6.47 | Reference |  | Reference |  | Reference |  |
| MI | Easing 3 | White | 12411685 | 9252 | 55898155 | 16.55 | 2.08 | (1.7, 2.55) | 1.12 | (0.91, 1.37) | 1.18 | (0.97, 1.45) |
| MI | Easing 3 | Asian | 1074694 | 783 | 4836634 | 16.19 | 2.14 | (1.73, 2.64) | 1.84 | (1.49, 2.28) | 1.72 | (1.39, 2.12) |
| MI | Easing 3 | Black | 369668 | 115 | 1661334 | 6.92 | 0.95 | (0.72, 1.24) | 0.78 | (0.59, 1.02) | 0.69 | (0.53, 0.9) |
| MI | Easing 3 | Mixed | 197112 | 53 | 885392 | 5.99 | 0.81 | (0.58, 1.13) | 0.91 | (0.65, 1.27) | 0.88 | (0.63, 1.23) |
| MI | Easing 3 | Other | 304150 | 98 | 1360084 | 7.21 | Reference |  | Reference |  | Reference |  |
| DKA | Pre | White | 841518 | 4898 | 21022238 | 23.3 | 2.22 | (1.58, 3.13) | 2.77 | (1.97, 3.9) | 2.94 | (2.1, 4.13) |
| DKA | Pre | Asian | 115505 | 188 | 2892286 | 6.5 | 0.59 | (0.41, 0.85) | 0.52 | (0.36, 0.75) | 0.51 | (0.35, 0.73) |
| DKA | Pre | Black | 28706 | 121 | 717714 | 16.86 | 1.48 | (1.01, 2.17) | 1.38 | (0.95, 2.03) | 1.31 | (0.9, 1.92) |
| DKA | Pre | Mixed | 9109 | 53 | 227450 | 23.3 | 2.14 | (1.39, 3.29) | 1.67 | (1.08, 2.56) | 1.64 | (1.07, 2.53) |
| DKA | Pre | Other | 12222 | 34 | 305866 | 11.12 | Reference |  | Reference |  | Reference |  |
| DKA | Pandemic | White | 995361 | 5666 | 24030509 | 23.58 | 2.28 | (1.68, 3.09) | 2.83 | (2.09, 3.83) | 3.01 | (2.22, 4.07) |
| DKA | Pandemic | Asian | 142353 | 244 | 3500862 | 6.97 | 0.65 | (0.47, 0.9) | 0.6 | (0.44, 0.83) | 0.59 | (0.42, 0.81) |
| DKA | Pandemic | Black | 36530 | 214 | 887198 | 24.12 | 2.2 | (1.59, 3.06) | 2.13 | (1.53, 2.95) | 2 | (1.44, 2.78) |
| DKA | Pandemic | Mixed | 11738 | 65 | 286457 | 22.69 | 2.12 | (1.44, 3.11) | 1.72 | (1.17, 2.54) | 1.7 | (1.15, 2.5) |
| DKA | Pandemic | Other | 16225 | 43 | 394708 | 10.89 | Reference |  | Reference |  | Reference |  |
| DKA | Wave 1 | White | 995392 | 588 | 2242264 | 26.22 | 1.58 | (0.7, 3.57) | 2.04 | (0.91, 4.59) | 2.24 | (0.99, 5.04) |
| DKA | Wave 1 | Asian | 142342 | 23 | 321261 | 7.16 | 0.44 | (0.18, 1.07) | 0.4 | (0.16, 0.99) | 0.39 | (0.16, 0.95) |
| DKA | Wave 1 | Black | 36526 | 21 | 82234 | 25.54 | 1.55 | (0.63, 3.86) | 1.5 | (0.61, 3.73) | 1.37 | (0.55, 3.4) |
| DKA | Wave 1 | Mixed | redacted |  |  |  |  |  |  |  |  |  |
| DKA | Wave 1 | Other | redacted |  |  |  |  |  |  |  |  |  |
| DKA | Easing 1 | White | 992829 | 891 | 3223673 | 27.64 | 2.27 | (1.07, 4.8) | 2.89 | (1.37, 6.12) | 3.12 | (1.47, 6.6) |
| DKA | Easing 1 | Asian | 142905 | 31 | 465180 | 6.66 | 0.51 | (0.23, 1.17) | 0.48 | (0.21, 1.08) | 0.46 | (0.2, 1.05) |
| DKA | Easing 1 | Black | 36652 | 37 | 119132 | 31.06 | 2.3 | (1.02, 5.16) | 2.26 | (1.01, 5.07) | 2.11 | (0.94, 4.74) |
| DKA | Easing 1 | Mixed | redacted |  |  |  |  |  |  |  |  |  |
| DKA | Easing 1 | Other | redacted |  |  |  |  |  |  |  |  |  |
| DKA | Wave 2 | White | 993240 | 2005 | 7442036 | 26.94 | 2.6 | (1.5, 4.5) | 3.3 | (1.91, 5.72) | 3.55 | (2.05, 6.14) |
| DKA | Wave 2 | Asian | 143331 | 89 | 1079487 | 8.24 | 0.79 | (0.44, 1.42) | 0.74 | (0.41, 1.33) | 0.72 | (0.4, 1.29) |
| DKA | Wave 2 | Black | 36803 | 65 | 276312 | 23.52 | 2.26 | (1.25, 4.11) | 2.2 | (1.21, 3.99) | 2.04 | (1.12, 3.7) |
| DKA | Wave 2 | Mixed | 11829 | 33 | 88847 | 37.14 | 3.57 | (1.88, 6.78) | 2.86 | (1.5, 5.43) | 2.81 | (1.48, 5.34) |
| DKA | Wave 2 | Other | 16467 | 13 | 123610 | 10.52 | Reference |  | Reference |  | Reference |  |
| DKA | Easing 2 | White | 997807 | 363 | 1062042 | 34.18 | 1.19 | (0.52, 2.72) | 1.48 | (0.65, 3.37) | 1.62 | (0.71, 3.67) |
| DKA | Easing 2 | Asian | 144411 | 17 | 153794 | 11.05 | 0.33 | (0.13, 0.83) | 0.31 | (0.12, 0.78) | 0.3 | (0.12, 0.76) |
| DKA | Easing 2 | Black | 37160 | 13 | 39575 | 32.85 | 0.94 | (0.36, 2.48) | 0.92 | (0.35, 2.43) | 0.86 | (0.33, 2.27) |
| DKA | Easing 2 | Mixed | redacted |  |  |  |  |  |  |  |  |  |
| DKA | Easing 2 | Other | redacted |  |  |  |  |  |  |  |  |  |
| DKA | Wave 3 | White | 999832 | 1974 | 6568038 | 30.05 | 1.86 | (1.16, 2.97) | 2.36 | (1.48, 3.76) | 2.5 | (1.57, 4) |
| DKA | Wave 3 | Asian | 144709 | 84 | 955023 | 8.8 | 0.55 | (0.33, 0.91) | 0.51 | (0.31, 0.85) | 0.5 | (0.3, 0.83) |
| DKA | Wave 3 | Black | 37309 | 70 | 245487 | 28.51 | 1.7 | (1.01, 2.86) | 1.67 | (1, 2.81) | 1.57 | (0.94, 2.64) |
| DKA | Wave 3 | Mixed | 12024 | 25 | 79183 | 31.57 | 1.92 | (1.05, 3.53) | 1.53 | (0.83, 2.81) | 1.51 | (0.82, 2.77) |
| DKA | Wave 3 | Other | 16735 | 18 | 110066 | 16.35 | Reference |  | Reference |  | Reference |  |
| DKA | Easing 3 | White | 1004850 | 1402 | 4506363 | 31.11 | 1.98 | (1.14, 3.45) | 2.49 | (1.44, 4.33) | 2.62 | (1.51, 4.55) |
| DKA | Easing 3 | Asian | 146317 | 65 | 658544 | 9.87 | 0.59 | (0.33, 1.08) | 0.56 | (0.31, 1.02) | 0.55 | (0.3, 1) |
| DKA | Easing 3 | Black | 37795 | 55 | 169620 | 32.43 | 1.88 | (1.03, 3.45) | 1.85 | (1.01, 3.39) | 1.76 | (0.96, 3.22) |
| DKA | Easing 3 | Mixed | 12233 | 17 | 54888 | 30.97 | 1.85 | (0.9, 3.82) | 1.5 | (0.73, 3.09) | 1.49 | (0.72, 3.07) |
| DKA | Easing 3 | Other | 16941 | 13 | 76042 | 17.1 | Reference |  | Reference |  | Reference |  |
| T1 DM | Pre | White | 87911 | 5492 | 2126366 | 258.28 | 1.69 | (1.24, 2.32) | 1.74 | (1.27, 2.39) | 1.92 | (1.4, 2.63) |
| T1 DM | Pre | Asian | 5914 | 141 | 146226 | 96.43 | 0.56 | (0.4, 0.8) | 0.63 | (0.44, 0.9) | 0.6 | (0.42, 0.85) |
| T1 DM | Pre | Black | 2450 | 105 | 59963 | 175.11 | 1 | (0.7, 1.45) | 1.09 | (0.76, 1.57) | 0.97 | (0.67, 1.4) |
| T1 DM | Pre | Mixed | 956 | 70 | 22919 | 305.42 | 1.85 | (1.25, 2.73) | 1.63 | (1.1, 2.4) | 1.58 | (1.07, 2.33) |
| T1 DM | Pre | Other | 949 | 40 | 23225 | 172.23 | Reference |  | Reference |  | Reference |  |
| T1 DM | Pandemic | White | 100454 | 5530 | 2385604 | 231.81 | 1.47 | (1.12, 1.94) | 1.53 | (1.16, 2.02) | 1.71 | (1.3, 2.25) |
| T1 DM | Pandemic | Asian | 7274 | 143 | 175440 | 81.51 | 0.46 | (0.34, 0.63) | 0.52 | (0.38, 0.72) | 0.5 | (0.36, 0.68) |
| T1 DM | Pandemic | Black | 3004 | 125 | 70660 | 176.9 | 1 | (0.72, 1.38) | 1.08 | (0.78, 1.49) | 0.95 | (0.69, 1.31) |
| T1 DM | Pandemic | Mixed | 1180 | 63 | 27871 | 226.04 | 1.35 | (0.93, 1.95) | 1.21 | (0.84, 1.75) | 1.17 | (0.81, 1.69) |
| T1 DM | Pandemic | Other | 1255 | 52 | 29573 | 175.84 | Reference |  | Reference |  | Reference |  |
| T1 DM | Wave 1 | White | 100463 | 616 | 226053 | 272.5 | 1.21 | (0.53, 2.72) | 1.27 | (0.57, 2.87) | 1.45 | (0.64, 3.27) |
| T1 DM | Wave 1 | Asian | 7275 | 13 | 16397 | 79.28 | 0.37 | (0.14, 0.99) | 0.43 | (0.16, 1.13) | 0.39 | (0.15, 1.02) |
| T1 DM | Wave 1 | Black | 3001 | 11 | 6726 | 163.55 | 0.77 | (0.29, 2.09) | 0.85 | (0.31, 2.29) | 0.7 | (0.26, 1.9) |
| T1 DM | Wave 1 | Mixed | redacted |  |  |  |  |  |  |  |  |  |
| T1 DM | Wave 1 | Other | redacted |  |  |  |  |  |  |  |  |  |
| T1 DM | Easing 1 | White | 100755 | 1042 | 325820 | 319.81 | 2.17 | (1.02, 4.58) | 2.27 | (1.07, 4.8) | 2.54 | (1.2, 5.37) |
| T1 DM | Easing 1 | Asian | 7311 | 23 | 23742 | 96.87 | 0.57 | (0.24, 1.33) | 0.67 | (0.29, 1.56) | 0.63 | (0.27, 1.47) |
| T1 DM | Easing 1 | Black | 3027 | 29 | 9786 | 296.35 | 1.73 | (0.76, 3.96) | 1.89 | (0.83, 4.32) | 1.67 | (0.73, 3.81) |
| T1 DM | Easing 1 | Mixed | 1194 | 14 | 3863 | 362.4 | 2.24 | (0.9, 5.56) | 1.99 | (0.8, 4.94) | 1.93 | (0.78, 4.8) |
| T1 DM | Easing 1 | Other | redacted |  |  |  |  |  |  |  |  |  |
| T1 DM | Wave 2 | White | 100749 | 2105 | 749144 | 280.99 | 1.92 | (1.15, 3.21) | 2.01 | (1.2, 3.35) | 2.27 | (1.36, 3.79) |
| T1 DM | Wave 2 | Asian | 7314 | 50 | 54660 | 91.47 | 0.57 | (0.32, 1.01) | 0.65 | (0.37, 1.16) | 0.6 | (0.34, 1.08) |
| T1 DM | Wave 2 | Black | 3052 | 46 | 22633 | 203.24 | 1.25 | (0.7, 2.25) | 1.35 | (0.75, 2.42) | 1.15 | (0.64, 2.07) |
| T1 DM | Wave 2 | Mixed | 1187 | 30 | 8769 | 342.1 | 2.23 | (1.2, 4.15) | 1.97 | (1.06, 3.67) | 1.89 | (1.01, 3.51) |
| T1 DM | Wave 2 | Other | 1263 | 15 | 9374 | 160.02 | Reference |  | Reference |  | Reference |  |
| T1 DM | Easing 2 | White | 100533 | 408 | 106809 | 381.99 | 0.71 | (0.35, 1.45) | 0.75 | (0.36, 1.52) | 0.85 | (0.41, 1.73) |
| T1 DM | Easing 2 | Asian | 7334 | 14 | 7801 | 179.46 | 0.3 | (0.13, 0.72) | 0.35 | (0.15, 0.84) | 0.33 | (0.14, 0.79) |
| T1 DM | Easing 2 | Black | redacted |  |  |  |  |  |  |  |  |  |
| T1 DM | Easing 2 | Mixed | redacted |  |  |  |  |  |  |  |  |  |
| T1 DM | Easing 2 | Other | redacted |  |  |  |  |  |  |  |  |  |
| T1 DM | Wave 3 | White | 100692 | 1975 | 656541 | 300.82 | 1.52 | (0.94, 2.47) | 1.62 | (1, 2.63) | 1.8 | (1.11, 2.92) |
| T1 DM | Wave 3 | Asian | 7359 | 58 | 48340 | 119.98 | 0.57 | (0.33, 0.98) | 0.67 | (0.39, 1.15) | 0.63 | (0.37, 1.09) |
| T1 DM | Wave 3 | Black | 3075 | 63 | 19944 | 315.88 | 1.5 | (0.88, 2.56) | 1.64 | (0.96, 2.81) | 1.46 | (0.86, 2.5) |
| T1 DM | Wave 3 | Mixed | 1172 | 27 | 7621 | 354.27 | 1.74 | (0.95, 3.2) | 1.52 | (0.83, 2.8) | 1.5 | (0.82, 2.75) |
| T1 DM | Wave 3 | Other | 1257 | 17 | 8156 | 208.44 | Reference |  | Reference |  | Reference |  |
| T1 DM | Easing 3 | White | 100982 | 1461 | 450456 | 324.34 | 1.24 | (0.75, 2.05) | 1.32 | (0.8, 2.18) | 1.51 | (0.91, 2.48) |
| T1 DM | Easing 3 | Asian | 7439 | 46 | 33317 | 138.07 | 0.47 | (0.27, 0.84) | 0.55 | (0.31, 0.97) | 0.52 | (0.3, 0.93) |
| T1 DM | Easing 3 | Black | 3126 | 33 | 13941 | 236.72 | 0.81 | (0.44, 1.46) | 0.88 | (0.48, 1.6) | 0.78 | (0.43, 1.43) |
| T1 DM | Easing 3 | Mixed | 1196 | 18 | 5313 | 338.81 | 1.22 | (0.62, 2.39) | 1.06 | (0.54, 2.09) | 1.06 | (0.54, 2.08) |
| T1 DM | Easing 3 | Other | 1243 | 16 | 5550 | 288.3 | Reference |  | Reference |  | Reference |  |
| T2 DM | Pre | White | 791860 | 8195 | 19744444 | 41.51 | 1.49 | (1.21, 1.85) | 1.52 | (1.23, 1.88) | 1.68 | (1.36, 2.09) |
| T2 DM | Pre | Asian | 114073 | 783 | 2849512 | 27.48 | 0.94 | (0.75, 1.17) | 0.93 | (0.75, 1.16) | 0.92 | (0.74, 1.15) |
| T2 DM | Pre | Black | 27906 | 348 | 695157 | 50.06 | 1.64 | (1.3, 2.08) | 1.62 | (1.28, 2.05) | 1.43 | (1.13, 1.8) |
| T2 DM | Pre | Mixed | 8600 | 76 | 214680 | 35.4 | 1.18 | (0.87, 1.61) | 1.18 | (0.87, 1.61) | 1.12 | (0.82, 1.53) |
| T2 DM | Pre | Other | 11805 | 87 | 294856 | 29.51 | Reference |  | Reference |  | Reference |  |
| T2 DM | Pandemic | White | 941455 | 11040 | 22648478 | 48.74 | 1.6 | (1.33, 1.91) | 1.59 | (1.33, 1.91) | 1.77 | (1.47, 2.11) |
| T2 DM | Pandemic | Asian | 140656 | 892 | 3452607 | 25.84 | 0.83 | (0.69, 1.01) | 0.83 | (0.69, 1.01) | 0.82 | (0.68, 1) |
| T2 DM | Pandemic | Black | 35567 | 482 | 861384 | 55.96 | 1.73 | (1.42, 2.11) | 1.72 | (1.41, 2.1) | 1.52 | (1.24, 1.85) |
| T2 DM | Pandemic | Mixed | 11131 | 124 | 271208 | 45.72 | 1.44 | (1.12, 1.84) | 1.44 | (1.12, 1.85) | 1.38 | (1.07, 1.77) |
| T2 DM | Pandemic | Other | 15672 | 122 | 380633 | 32.05 | Reference |  | Reference |  | Reference |  |
| T2 DM | Wave 1 | White | 941483 | 870 | 2120264 | 41.03 | 1.35 | (0.74, 2.47) | 1.37 | (0.75, 2.51) | 1.58 | (0.86, 2.89) |
| T2 DM | Wave 1 | Asian | 140644 | 57 | 317405 | 17.96 | 0.61 | (0.32, 1.17) | 0.61 | (0.32, 1.17) | 0.61 | (0.32, 1.16) |
| T2 DM | Wave 1 | Black | 35564 | 40 | 80057 | 49.96 | 1.62 | (0.83, 3.17) | 1.61 | (0.82, 3.13) | 1.38 | (0.71, 2.7) |
| T2 DM | Wave 1 | Mixed | redacted |  |  |  |  |  |  |  |  |  |
| T2 DM | Wave 1 | Other | 15687 | 11 | 35357 | 31.11 | Reference |  | Reference |  | Reference |  |
| T2 DM | Easing 1 | White | 938717 | 1720 | 3046173 | 56.46 | 1.53 | (0.98, 2.38) | 1.5 | (0.96, 2.35) | 1.68 | (1.07, 2.62) |
| T2 DM | Easing 1 | Asian | 141193 | 122 | 459473 | 26.55 | 0.7 | (0.44, 1.13) | 0.71 | (0.44, 1.14) | 0.7 | (0.44, 1.13) |
| T2 DM | Easing 1 | Black | 35668 | 77 | 115876 | 66.45 | 1.66 | (1.01, 2.72) | 1.66 | (1.01, 2.71) | 1.44 | (0.88, 2.37) |
| T2 DM | Easing 1 | Mixed | 11151 | 13 | 36284 | 35.83 | 0.92 | (0.45, 1.84) | 0.92 | (0.46, 1.86) | 0.88 | (0.44, 1.76) |
| T2 DM | Easing 1 | Other | 15777 | 20 | 51264 | 39.01 | Reference |  | Reference |  | Reference |  |
| T2 DM | Wave 2 | White | 939511 | 3523 | 7032361 | 50.1 | 1.49 | (1.1, 2.03) | 1.51 | (1.11, 2.05) | 1.7 | (1.25, 2.3) |
| T2 DM | Wave 2 | Asian | 141615 | 291 | 1065878 | 27.3 | 0.79 | (0.57, 1.09) | 0.79 | (0.57, 1.09) | 0.78 | (0.56, 1.07) |
| T2 DM | Wave 2 | Black | 35811 | 160 | 268616 | 59.56 | 1.66 | (1.18, 2.33) | 1.65 | (1.18, 2.31) | 1.45 | (1.03, 2.03) |
| T2 DM | Wave 2 | Mixed | 11220 | 46 | 84263 | 54.59 | 1.54 | (1.02, 2.34) | 1.55 | (1.02, 2.35) | 1.48 | (0.97, 2.24) |
| T2 DM | Wave 2 | Other | 15915 | 43 | 119405 | 36.01 | Reference |  | Reference |  | Reference |  |
| T2 DM | Easing 2 | White | 945374 | 624 | 1006055 | 62.02 | 1.87 | (0.83, 4.21) | 1.85 | (0.82, 4.16) | 2.08 | (0.92, 4.7) |
| T2 DM | Easing 2 | Asian | 142718 | 61 | 151971 | 40.14 | 1.18 | (0.51, 2.75) | 1.19 | (0.51, 2.76) | 1.19 | (0.51, 2.77) |
| T2 DM | Easing 2 | Black | 36188 | 31 | 38532 | 80.45 | 2.26 | (0.94, 5.42) | 2.25 | (0.94, 5.4) | 1.97 | (0.82, 4.74) |
| T2 DM | Easing 2 | Mixed | redacted |  |  |  |  |  |  |  |  |  |
| T2 DM | Easing 2 | Other | redacted |  |  |  |  |  |  |  |  |  |
| T2 DM | Wave 3 | White | 947473 | 3295 | 6218927 | 52.98 | 1.53 | (1.12, 2.11) | 1.53 | (1.11, 2.1) | 1.68 | (1.22, 2.32) |
| T2 DM | Wave 3 | Asian | 143015 | 289 | 943099 | 30.64 | 0.87 | (0.62, 1.21) | 0.87 | (0.62, 1.22) | 0.86 | (0.61, 1.2) |
| T2 DM | Wave 3 | Black | 36330 | 127 | 238909 | 53.16 | 1.43 | (1, 2.05) | 1.44 | (1, 2.06) | 1.26 | (0.88, 1.81) |
| T2 DM | Wave 3 | Mixed | 11430 | 44 | 75226 | 58.49 | 1.6 | (1.04, 2.46) | 1.62 | (1.05, 2.49) | 1.54 | (1, 2.37) |
| T2 DM | Wave 3 | Other | 16203 | 39 | 106510 | 36.62 | Reference |  | Reference |  | Reference |  |
| T2 DM | Easing 3 | White | 953549 | 2229 | 4273597 | 52.16 | 3.2 | (1.85, 5.54) | 3.18 | (1.84, 5.5) | 3.47 | (2.01, 6.02) |
| T2 DM | Easing 3 | Asian | 144636 | 182 | 650752 | 27.97 | 1.61 | (0.92, 2.84) | 1.62 | (0.92, 2.85) | 1.61 | (0.92, 2.83) |
| T2 DM | Easing 3 | Black | 36828 | 113 | 165179 | 68.41 | 3.84 | (2.16, 6.82) | 3.84 | (2.16, 6.82) | 3.45 | (1.94, 6.13) |
| T2 DM | Easing 3 | Mixed | 11643 | 32 | 52210 | 61.29 | 3.55 | (1.86, 6.77) | 3.59 | (1.88, 6.84) | 3.44 | (1.81, 6.56) |
| T2 DM | Easing 3 | Other | 16434 | 13 | 73768 | 17.62 | Reference |  | Reference |  | Reference |  |
| Anxiety | Pre | White | 11951915 | 4087 | 299505903 | 1.36 | 1.52 | (1.16, 2) | 1.43 | (1.09, 1.87) | 1.5 | (1.14, 1.97) |
| Anxiety | Pre | Asian | 947198 | 279 | 23736235 | 1.18 | 1.35 | (1, 1.81) | 1.34 | (1, 1.8) | 1.21 | (0.9, 1.62) |
| Anxiety | Pre | Black | 323687 | 60 | 8111470 | 0.74 | 0.83 | (0.57, 1.2) | 0.82 | (0.56, 1.18) | 0.7 | (0.48, 1.01) |
| Anxiety | Pre | Mixed | 167862 | 42 | 4206466 | 1 | 1.13 | (0.76, 1.7) | 1.12 | (0.75, 1.68) | 1.07 | (0.71, 1.6) |
| Anxiety | Pre | Other | 255875 | 53 | 6411343 | 0.83 | Reference |  | Reference |  | Reference |  |
| Anxiety | Pandemic | White | 13007851 | 4604 | 321888666 | 1.43 | 1.34 | (1.06, 1.68) | 1.29 | (1.03, 1.62) | 1.35 | (1.08, 1.7) |
| Anxiety | Pandemic | Asian | 1116587 | 316 | 27562426 | 1.15 | 1.16 | (0.9, 1.49) | 1.16 | (0.9, 1.49) | 1.06 | (0.82, 1.36) |
| Anxiety | Pandemic | Black | 389040 | 80 | 9540013 | 0.84 | 0.82 | (0.6, 1.13) | 0.82 | (0.6, 1.13) | 0.72 | (0.52, 0.98) |
| Anxiety | Pandemic | Mixed | 206060 | 68 | 5035801 | 1.35 | 1.34 | (0.97, 1.86) | 1.33 | (0.96, 1.84) | 1.28 | (0.92, 1.77) |
| Anxiety | Pandemic | Other | 334275 | 76 | 8015275 | 0.95 | Reference |  | Reference |  | Reference |  |
| Anxiety | Wave 1 | White | 13008075 | 365 | 29413099 | 1.24 | 1.05 | (0.5, 2.23) | 0.99 | (0.47, 2.11) | 1.06 | (0.5, 2.25) |
| Anxiety | Wave 1 | Asian | 1116590 | 27 | 2525244 | 1.07 | 1.12 | (0.48, 2.57) | 1.1 | (0.48, 2.54) | 0.99 | (0.43, 2.29) |
| Anxiety | Wave 1 | Black | redacted |  |  |  |  |  |  |  |  |  |
| Anxiety | Wave 1 | Mixed | redacted |  |  |  |  |  |  |  |  |  |
| Anxiety | Wave 1 | Other | redacted |  |  |  |  |  |  |  |  |  |
| Anxiety | Easing 1 | White | 13059230 | 725 | 42519578 | 1.71 | 1.33 | (0.75, 2.37) | 1.24 | (0.7, 2.21) | 1.32 | (0.74, 2.35) |
| Anxiety | Easing 1 | Asian | 1128788 | 45 | 3674977 | 1.22 | 1.07 | (0.56, 2.02) | 1.06 | (0.56, 2.01) | 0.95 | (0.5, 1.8) |
| Anxiety | Easing 1 | Black | redacted |  |  |  |  |  |  |  |  |  |
| Anxiety | Easing 1 | Mixed | 208793 | 15 | 678914 | 2.21 | 1.85 | (0.87, 3.96) | 1.84 | (0.86, 3.93) | 1.74 | (0.81, 3.72) |
| Anxiety | Easing 1 | Other | 338288 | 12 | 1098296 | 1.09 | Reference |  | Reference |  | Reference |  |
| Anxiety | Wave 2 | White | 12967234 | 1399 | 97842210 | 1.43 | 1.85 | (1.14, 2.99) | 1.74 | (1.07, 2.81) | 1.82 | (1.12, 2.94) |
| Anxiety | Wave 2 | Asian | 1122609 | 82 | 8461143 | 0.97 | 1.35 | (0.8, 2.29) | 1.35 | (0.8, 2.27) | 1.24 | (0.73, 2.09) |
| Anxiety | Wave 2 | Black | 390037 | 29 | 2935073 | 0.99 | 1.37 | (0.75, 2.49) | 1.35 | (0.74, 2.46) | 1.19 | (0.65, 2.16) |
| Anxiety | Wave 2 | Mixed | 207205 | 27 | 1555417 | 1.74 | 2.39 | (1.3, 4.4) | 2.37 | (1.29, 4.35) | 2.28 | (1.24, 4.19) |
| Anxiety | Wave 2 | Other | 332902 | 17 | 2485666 | 0.68 | Reference |  | Reference |  | Reference |  |
| Anxiety | Easing 2 | White | 12640489 | 237 | 13462945 | 1.76 | 6.1 | (0.85, 43.69) | 5.7 | (0.79, 40.92) | 6.13 | (0.85, 43.97) |
| Anxiety | Easing 2 | Asian | 1095138 | 19 | 1166268 | 1.63 | 5.89 | (0.79, 44.08) | 5.86 | (0.78, 43.83) | 5.46 | (0.73, 40.86) |
| Anxiety | Easing 2 | Black | redacted |  |  |  |  |  |  |  |  |  |
| Anxiety | Easing 2 | Mixed | redacted |  |  |  |  |  |  |  |  |  |
| Anxiety | Easing 2 | Other | redacted |  |  |  |  |  |  |  |  |  |
| Anxiety | Wave 3 | White | 12602332 | 1185 | 83187132 | 1.42 | 1.13 | (0.74, 1.73) | 1.12 | (0.73, 1.71) | 1.17 | (0.77, 1.8) |
| Anxiety | Wave 3 | Asian | 1091692 | 94 | 7195031 | 1.31 | 1.17 | (0.74, 1.87) | 1.18 | (0.74, 1.88) | 1.09 | (0.69, 1.75) |
| Anxiety | Wave 3 | Black | 377686 | 21 | 2483064 | 0.85 | 0.71 | (0.39, 1.29) | 0.72 | (0.39, 1.3) | 0.64 | (0.35, 1.17) |
| Anxiety | Wave 3 | Mixed | 200227 | 15 | 1315159 | 1.14 | 0.98 | (0.51, 1.9) | 0.97 | (0.5, 1.86) | 0.94 | (0.49, 1.81) |
| Anxiety | Wave 3 | Other | 314768 | 22 | 2054920 | 1.07 | Reference |  | Reference |  | Reference |  |
| Anxiety | Easing 3 | White | 12411761 | 691 | 55915750 | 1.24 | 1.39 | (0.76, 2.53) | 1.37 | (0.75, 2.49) | 1.43 | (0.78, 2.61) |
| Anxiety | Easing 3 | Asian | 1074699 | 48 | 4838179 | 0.99 | 1.16 | (0.6, 2.25) | 1.16 | (0.6, 2.24) | 1.07 | (0.55, 2.06) |
| Anxiety | Easing 3 | Black | redacted |  |  |  |  |  |  |  |  |  |
| Anxiety | Easing 3 | Mixed | redacted |  |  |  |  |  |  |  |  |  |
| Anxiety | Easing 3 | Other | 304150 | 11 | 1360271 | 0.81 | Reference |  | Reference |  | Reference |  |
| Depression | Pre | White | 11951915 | 2218 | 299528329 | 0.74 | 1.88 | (1.25, 2.84) | 1.9 | (1.26, 2.88) | 2.07 | (1.37, 3.13) |
| Depression | Pre | Asian | 947198 | 83 | 23738551 | 0.35 | 0.93 | (0.59, 1.48) | 0.94 | (0.59, 1.49) | 0.84 | (0.53, 1.33) |
| Depression | Pre | Black | 323687 | 35 | 8111737 | 0.43 | 0.98 | (0.58, 1.67) | 1.01 | (0.59, 1.7) | 0.83 | (0.49, 1.41) |
| Depression | Pre | Mixed | 167862 | 35 | 4206541 | 0.83 | 2.02 | (1.2, 3.43) | 2.01 | (1.19, 3.41) | 1.91 | (1.13, 3.24) |
| Depression | Pre | Other | 255875 | 23 | 6411754 | 0.36 | Reference |  | Reference |  | Reference |  |
| Depression | Pandemic | White | 13007854 | 2181 | 321918259 | 0.68 | 1.57 | (1.08, 2.26) | 1.45 | (1, 2.1) | 1.57 | (1.09, 2.27) |
| Depression | Pandemic | Asian | 1116587 | 97 | 27564956 | 0.35 | 0.99 | (0.65, 1.5) | 0.98 | (0.65, 1.49) | 0.89 | (0.59, 1.36) |
| Depression | Pandemic | Black | 389040 | 41 | 9540463 | 0.43 | 1.01 | (0.63, 1.63) | 1.03 | (0.64, 1.65) | 0.87 | (0.54, 1.41) |
| Depression | Pandemic | Mixed | 206060 | 27 | 5036547 | 0.54 | 1.29 | (0.76, 2.18) | 1.3 | (0.77, 2.2) | 1.25 | (0.74, 2.11) |
| Depression | Pandemic | Other | 334275 | 29 | 8015812 | 0.36 | Reference |  | Reference |  | Reference |  |
| Depression | Wave 1 | White | 13008078 | 185 | 29413291 | 0.63 | 1.72 | (0.43, 6.98) | 1.59 | (0.39, 6.46) | 1.66 | (0.41, 6.75) |
| Depression | Wave 1 | Asian | redacted |  |  |  |  |  |  |  |  |  |
| Depression | Wave 1 | Black | redacted |  |  |  |  |  |  |  |  |  |
| Depression | Wave 1 | Mixed | redacted |  |  |  |  |  |  |  |  |  |
| Depression | Wave 1 | Other | redacted |  |  |  |  |  |  |  |  |  |
| Depression | Easing 1 | White | 13059246 | 406 | 42520051 | 0.95 | 1.15 | (0.54, 2.44) | 1.1 | (0.52, 2.34) | 1.21 | (0.57, 2.57) |
| Depression | Easing 1 | Asian | 1128788 | 16 | 3675015 | 0.44 | 0.7 | (0.29, 1.7) | 0.7 | (0.29, 1.71) | 0.63 | (0.26, 1.53) |
| Depression | Easing 1 | Black | 393362 | 12 | 1279474 | 0.94 | 1.16 | (0.46, 2.96) | 1.17 | (0.46, 2.98) | 0.98 | (0.39, 2.51) |
| Depression | Easing 1 | Mixed | redacted |  |  |  |  |  |  |  |  |  |
| Depression | Easing 1 | Other | redacted |  |  |  |  |  |  |  |  |  |
| Depression | Wave 2 | White | 12967236 | 697 | 97844796 | 0.71 | 2.57 | (1.15, 5.76) | 2.41 | (1.07, 5.41) | 2.59 | (1.16, 5.82) |
| Depression | Wave 2 | Asian | 1122609 | 29 | 8461282 | 0.34 | 1.41 | (0.59, 3.41) | 1.41 | (0.59, 3.41) | 1.3 | (0.54, 3.13) |
| Depression | Wave 2 | Black | 390037 | 12 | 2935109 | 0.41 | 1.43 | (0.54, 3.82) | 1.46 | (0.55, 3.89) | 1.26 | (0.47, 3.36) |
| Depression | Wave 2 | Mixed | redacted |  |  |  |  |  |  |  |  |  |
| Depression | Wave 2 | Other | redacted |  |  |  |  |  |  |  |  |  |
| Depression | Easing 2 | White | 12640487 | 108 | 13463005 | 0.8 | 1.15 | (0.28, 4.7) | 1.03 | (0.25, 4.23) | 1.08 | (0.26, 4.46) |
| Depression | Easing 2 | Asian | redacted |  |  |  |  |  |  |  |  |  |
| Depression | Easing 2 | Black | redacted |  |  |  |  |  |  |  |  |  |
| Depression | Easing 2 | Mixed | redacted |  |  |  |  |  |  |  |  |  |
| Depression | Easing 2 | Other | redacted |  |  |  |  |  |  |  |  |  |
| Depression | Wave 3 | White | 12602338 | 504 | 83189402 | 0.61 | 1.71 | (0.76, 3.83) | 1.57 | (0.7, 3.53) | 1.71 | (0.76, 3.84) |
| Depression | Wave 3 | Asian | 1091692 | 26 | 7195242 | 0.36 | 1.28 | (0.52, 3.11) | 1.28 | (0.53, 3.12) | 1.15 | (0.47, 2.81) |
| Depression | Wave 3 | Black | redacted |  |  |  |  |  |  |  |  |  |
| Depression | Wave 3 | Mixed | redacted |  |  |  |  |  |  |  |  |  |
| Depression | Wave 3 | Other | redacted |  |  |  |  |  |  |  |  |  |
| Depression | Easing 3 | White | 12411765 | 310 | 55916565 | 0.55 | 2.23 | (0.71, 6.97) | 1.98 | (0.63, 6.21) | 2.15 | (0.68, 6.74) |
| Depression | Easing 3 | Asian | 1074699 | 15 | 4838248 | 0.31 | 1.33 | (0.38, 4.61) | 1.32 | (0.38, 4.57) | 1.18 | (0.34, 4.11) |
| Depression | Easing 3 | Black | redacted |  |  |  |  |  |  |  |  |  |
| Depression | Easing 3 | Mixed | redacted |  |  |  |  |  |  |  |  |  |
| Depression | Easing 3 | Other | redacted |  |  |  |  |  |  |  |  |  |
| Asthma | Pre | White | 1104943 | 14508 | 27504757 | 52.75 | 0.9 | (0.77, 1.04) | 0.88 | (0.76, 1.02) | 0.88 | (0.75, 1.02) |
| Asthma | Pre | Asian | 72238 | 1870 | 1785399 | 104.74 | 1.6 | (1.37, 1.86) | 1.61 | (1.38, 1.88) | 1.37 | (1.17, 1.6) |
| Asthma | Pre | Black | 21726 | 436 | 538585 | 80.95 | 1.26 | (1.06, 1.5) | 1.23 | (1.03, 1.47) | 1.02 | (0.85, 1.21) |
| Asthma | Pre | Mixed | 14090 | 227 | 350142 | 64.83 | 1.04 | (0.86, 1.27) | 1.03 | (0.84, 1.25) | 0.96 | (0.79, 1.17) |
| Asthma | Pre | Other | 11220 | 176 | 278865 | 63.11 | Reference |  | Reference |  | Reference |  |
| Asthma | Pandemic | White | 1230577 | 9804 | 30424471 | 32.22 | 1.01 | (0.84, 1.22) | 1.01 | (0.84, 1.22) | 1.01 | (0.84, 1.22) |
| Asthma | Pandemic | Asian | 84572 | 1128 | 2090544 | 53.96 | 1.52 | (1.26, 1.85) | 1.54 | (1.27, 1.87) | 1.35 | (1.11, 1.63) |
| Asthma | Pandemic | Black | 25738 | 279 | 632429 | 44.12 | 1.25 | (1, 1.55) | 1.22 | (0.98, 1.52) | 1.03 | (0.83, 1.28) |
| Asthma | Pandemic | Mixed | 17059 | 155 | 418542 | 37.03 | 1.1 | (0.86, 1.4) | 1.06 | (0.83, 1.35) | 1.01 | (0.79, 1.28) |
| Asthma | Pandemic | Other | 13884 | 115 | 340269 | 33.8 | Reference |  | Reference |  | Reference |  |
| Asthma | Wave 1 | White | 1230560 | 1011 | 2780998 | 36.35 | 2.03 | (0.84, 4.9) | 2.05 | (0.85, 4.95) | 1.96 | (0.81, 4.74) |
| Asthma | Wave 1 | Asian | 84566 | 68 | 191126 | 35.58 | 2.07 | (0.83, 5.15) | 2.11 | (0.85, 5.25) | 1.71 | (0.69, 4.25) |
| Asthma | Wave 1 | Black | 25739 | 18 | 58115 | 30.97 | 1.96 | (0.73, 5.28) | 1.91 | (0.71, 5.15) | 1.5 | (0.56, 4.06) |
| Asthma | Wave 1 | Mixed | 17045 | 12 | 38540 | 31.14 | 1.87 | (0.66, 5.31) | 1.85 | (0.65, 5.25) | 1.68 | (0.59, 4.77) |
| Asthma | Wave 1 | Other | redacted |  |  |  |  |  |  |  |  |  |
| Asthma | Easing 1 | White | 1239227 | 1453 | 4033325 | 36.02 | 1.03 | (0.63, 1.69) | 1.04 | (0.63, 1.71) | 1.02 | (0.62, 1.68) |
| Asthma | Easing 1 | Asian | 85465 | 110 | 278243 | 39.53 | 1.09 | (0.64, 1.84) | 1.11 | (0.65, 1.87) | 0.89 | (0.53, 1.51) |
| Asthma | Easing 1 | Black | 25983 | 34 | 84514 | 40.23 | 1.08 | (0.6, 1.97) | 1.06 | (0.59, 1.92) | 0.83 | (0.46, 1.51) |
| Asthma | Easing 1 | Mixed | 17414 | 19 | 56614 | 33.56 | 0.92 | (0.47, 1.8) | 0.9 | (0.46, 1.75) | 0.82 | (0.42, 1.6) |
| Asthma | Easing 1 | Other | 14127 | 16 | 45931 | 34.83 | Reference |  | Reference |  | Reference |  |
| Asthma | Wave 2 | White | 1235344 | 3169 | 9316516 | 34.01 | 1.14 | (0.8, 1.61) | 1.16 | (0.82, 1.63) | 1.14 | (0.8, 1.61) |
| Asthma | Wave 2 | Asian | 85717 | 311 | 646184 | 48.13 | 1.47 | (1.02, 2.1) | 1.49 | (1.04, 2.14) | 1.25 | (0.87, 1.79) |
| Asthma | Wave 2 | Black | 25961 | 101 | 195315 | 51.71 | 1.58 | (1.07, 2.35) | 1.54 | (1.04, 2.29) | 1.25 | (0.84, 1.85) |
| Asthma | Wave 2 | Mixed | 17437 | 42 | 130946 | 32.07 | 1.02 | (0.65, 1.61) | 0.97 | (0.61, 1.53) | 0.89 | (0.57, 1.41) |
| Asthma | Wave 2 | Other | 14058 | 33 | 105601 | 31.25 | Reference |  | Reference |  | Reference |  |
| Asthma | Easing 2 | White | 1207813 | 677 | 1286418 | 52.63 | 0.83 | (0.44, 1.57) | 0.86 | (0.46, 1.61) | 0.84 | (0.44, 1.58) |
| Asthma | Easing 2 | Asian | 84468 | 84 | 89957 | 93.38 | 1.24 | (0.64, 2.4) | 1.27 | (0.66, 2.45) | 1.03 | (0.53, 2) |
| Asthma | Easing 2 | Black | 25562 | 20 | 27222 | 73.47 | 0.99 | (0.46, 2.12) | 0.97 | (0.46, 2.08) | 0.77 | (0.36, 1.65) |
| Asthma | Easing 2 | Mixed | redacted |  |  |  |  |  |  |  |  |  |
| Asthma | Easing 2 | Other | redacted |  |  |  |  |  |  |  |  |  |
| Asthma | Wave 3 | White | 1207064 | 3813 | 7964754 | 47.87 | 0.96 | (0.73, 1.27) | 0.97 | (0.74, 1.28) | 0.97 | (0.74, 1.28) |
| Asthma | Wave 3 | Asian | 84457 | 482 | 556459 | 86.62 | 1.45 | (1.09, 1.94) | 1.48 | (1.11, 1.98) | 1.29 | (0.97, 1.72) |
| Asthma | Wave 3 | Black | 25521 | 120 | 168043 | 71.41 | 1.2 | (0.87, 1.66) | 1.17 | (0.85, 1.63) | 1 | (0.72, 1.39) |
| Asthma | Wave 3 | Mixed | 17084 | 75 | 112349 | 66.76 | 1.2 | (0.84, 1.71) | 1.15 | (0.81, 1.64) | 1.09 | (0.77, 1.56) |
| Asthma | Wave 3 | Other | 13693 | 52 | 89958 | 57.8 | Reference |  | Reference |  | Reference |  |
| Asthma | Easing 3 | White | 1197790 | 2330 | 5394041 | 43.2 | 0.83 | (0.59, 1.17) | 0.83 | (0.59, 1.16) | 0.84 | (0.6, 1.17) |
| Asthma | Easing 3 | Asian | 84615 | 327 | 380842 | 85.86 | 1.41 | (1, 2.01) | 1.43 | (1.01, 2.03) | 1.25 | (0.88, 1.77) |
| Asthma | Easing 3 | Black | 25401 | 76 | 114225 | 66.54 | 1.1 | (0.74, 1.64) | 1.07 | (0.72, 1.6) | 0.9 | (0.61, 1.35) |
| Asthma | Easing 3 | Mixed | 16959 | 46 | 76258 | 60.32 | 1.07 | (0.69, 1.66) | 1.04 | (0.67, 1.61) | 0.98 | (0.63, 1.53) |
| Asthma | Easing 3 | Other | 13585 | 35 | 61096 | 57.29 | Reference |  | Reference |  | Reference |  |
| COPD | Pre | White | 409471 | 34264 | 9831716 | 348.5 | 1.28 | (1.07, 1.53) | 1.22 | (1.02, 1.45) | 1.23 | (1.03, 1.47) |
| COPD | Pre | Asian | 8290 | 531 | 200489 | 264.85 | 0.93 | (0.77, 1.14) | 0.94 | (0.77, 1.14) | 0.89 | (0.74, 1.09) |
| COPD | Pre | Black | 2556 | 144 | 62265 | 231.27 | 0.82 | (0.64, 1.04) | 0.83 | (0.65, 1.06) | 0.77 | (0.61, 0.98) |
| COPD | Pre | Mixed | 1379 | 107 | 33202 | 322.27 | 1.17 | (0.91, 1.52) | 1.2 | (0.92, 1.55) | 1.18 | (0.91, 1.53) |
| COPD | Pre | Other | 1851 | 123 | 44939 | 273.7 | Reference |  | Reference |  | Reference |  |
| COPD | Pandemic | White | 497200 | 33511 | 11478967 | 291.93 | 1.66 | (1.36, 2.03) | 1.57 | (1.29, 1.92) | 1.6 | (1.31, 1.95) |
| COPD | Pandemic | Asian | 10220 | 443 | 240147 | 184.47 | 1.02 | (0.82, 1.28) | 1.02 | (0.82, 1.27) | 0.96 | (0.77, 1.19) |
| COPD | Pandemic | Black | 3277 | 138 | 77025 | 179.16 | 1 | (0.77, 1.3) | 1.02 | (0.78, 1.32) | 0.93 | (0.72, 1.21) |
| COPD | Pandemic | Mixed | 1766 | 92 | 41773 | 220.24 | 1.26 | (0.95, 1.68) | 1.31 | (0.98, 1.74) | 1.3 | (0.98, 1.73) |
| COPD | Pandemic | Other | 2405 | 97 | 56013 | 173.17 | Reference |  | Reference |  | Reference |  |
| COPD | Wave 1 | White | 497196 | 3088 | 1114997 | 276.95 | 1.61 | (0.83, 3.1) | 1.54 | (0.8, 2.97) | 1.57 | (0.81, 3.03) |
| COPD | Wave 1 | Asian | 10221 | 32 | 22957 | 139.39 | 0.78 | (0.37, 1.64) | 0.78 | (0.37, 1.63) | 0.72 | (0.34, 1.51) |
| COPD | Wave 1 | Black | redacted |  |  |  |  |  |  |  |  |  |
| COPD | Wave 1 | Mixed | redacted |  |  |  |  |  |  |  |  |  |
| COPD | Wave 1 | Other | redacted |  |  |  |  |  |  |  |  |  |
| COPD | Easing 1 | White | 493737 | 5495 | 1591832 | 345.2 | 1.76 | (1.08, 2.88) | 1.68 | (1.02, 2.74) | 1.7 | (1.04, 2.79) |
| COPD | Easing 1 | Asian | 10226 | 64 | 33100 | 193.36 | 0.93 | (0.54, 1.61) | 0.93 | (0.53, 1.6) | 0.86 | (0.5, 1.49) |
| COPD | Easing 1 | Black | 3267 | 22 | 10548 | 208.57 | 0.98 | (0.51, 1.87) | 1 | (0.52, 1.9) | 0.91 | (0.48, 1.74) |
| COPD | Easing 1 | Mixed | 1768 | 14 | 5735 | 244.1 | 1.2 | (0.59, 2.46) | 1.25 | (0.61, 2.55) | 1.23 | (0.6, 2.53) |
| COPD | Easing 1 | Other | 2407 | 16 | 7765 | 206.06 | Reference |  | Reference |  | Reference |  |
| COPD | Wave 2 | White | 489153 | 10994 | 3607014 | 304.8 | 1.65 | (1.16, 2.34) | 1.56 | (1.1, 2.21) | 1.6 | (1.13, 2.26) |
| COPD | Wave 2 | Asian | 10158 | 146 | 75174 | 194.22 | 1.03 | (0.7, 1.5) | 1.02 | (0.69, 1.49) | 0.96 | (0.66, 1.41) |
| COPD | Wave 2 | Black | 3229 | 50 | 23911 | 209.11 | 1.11 | (0.71, 1.74) | 1.13 | (0.73, 1.76) | 1.03 | (0.66, 1.61) |
| COPD | Wave 2 | Mixed | 1784 | 30 | 13200 | 227.26 | 1.23 | (0.75, 2.03) | 1.29 | (0.78, 2.12) | 1.29 | (0.78, 2.12) |
| COPD | Wave 2 | Other | 2372 | 32 | 17549 | 182.35 | Reference |  | Reference |  | Reference |  |
| COPD | Easing 2 | White | 478271 | 2532 | 507374 | 499.04 | 2.15 | (0.96, 4.82) | 2 | (0.9, 4.48) | 2.01 | (0.9, 4.49) |
| COPD | Easing 2 | Asian | 10036 | 30 | 10656 | 281.52 | 1.2 | (0.5, 2.9) | 1.21 | (0.5, 2.9) | 1.1 | (0.46, 2.65) |
| COPD | Easing 2 | Black | 3242 | 11 | 3446 | 319.17 | 1.37 | (0.51, 3.71) | 1.39 | (0.52, 3.77) | 1.25 | (0.46, 3.39) |
| COPD | Easing 2 | Mixed | redacted |  |  |  |  |  |  |  |  |  |
| COPD | Easing 2 | Other | redacted |  |  |  |  |  |  |  |  |  |
| COPD | Wave 3 | White | 477395 | 12942 | 3083669 | 419.69 | 1.54 | (1.14, 2.09) | 1.44 | (1.06, 1.96) | 1.46 | (1.07, 1.97) |
| COPD | Wave 3 | Asian | 10023 | 181 | 65161 | 277.77 | 0.99 | (0.7, 1.38) | 0.99 | (0.7, 1.38) | 0.91 | (0.65, 1.28) |
| COPD | Wave 3 | Black | 3229 | 51 | 21022 | 242.61 | 0.88 | (0.59, 1.33) | 0.89 | (0.59, 1.34) | 0.8 | (0.53, 1.2) |
| COPD | Wave 3 | Mixed | 1809 | 30 | 11751 | 255.29 | 0.95 | (0.59, 1.51) | 0.98 | (0.61, 1.56) | 0.96 | (0.6, 1.54) |
| COPD | Wave 3 | Other | 2422 | 42 | 15769 | 266.35 | Reference |  | Reference |  | Reference |  |
| COPD | Easing 3 | White | 472883 | 9016 | 2093051 | 430.76 | 1.44 | (1.01, 2.06) | 1.34 | (0.94, 1.91) | 1.36 | (0.95, 1.94) |
| COPD | Easing 3 | Asian | 9987 | 132 | 44390 | 297.37 | 0.96 | (0.65, 1.42) | 0.96 | (0.65, 1.41) | 0.89 | (0.6, 1.31) |
| COPD | Easing 3 | Black | 3230 | 41 | 14355 | 285.62 | 0.95 | (0.6, 1.51) | 0.96 | (0.6, 1.53) | 0.86 | (0.54, 1.38) |
| COPD | Easing 3 | Mixed | 1849 | 31 | 8222 | 377.01 | 1.28 | (0.78, 2.11) | 1.34 | (0.82, 2.21) | 1.33 | (0.81, 2.19) |
| COPD | Easing 3 | Other | 2428 | 31 | 10808 | 286.83 | Reference |  | Reference |  | Reference |  |

### Schoenfeld residual plots for pre-pandemic and pandemic time-periods where scaled residuals for each ethnicity versus White are shown for each outcome. All plots are for age and sex adjusted models.


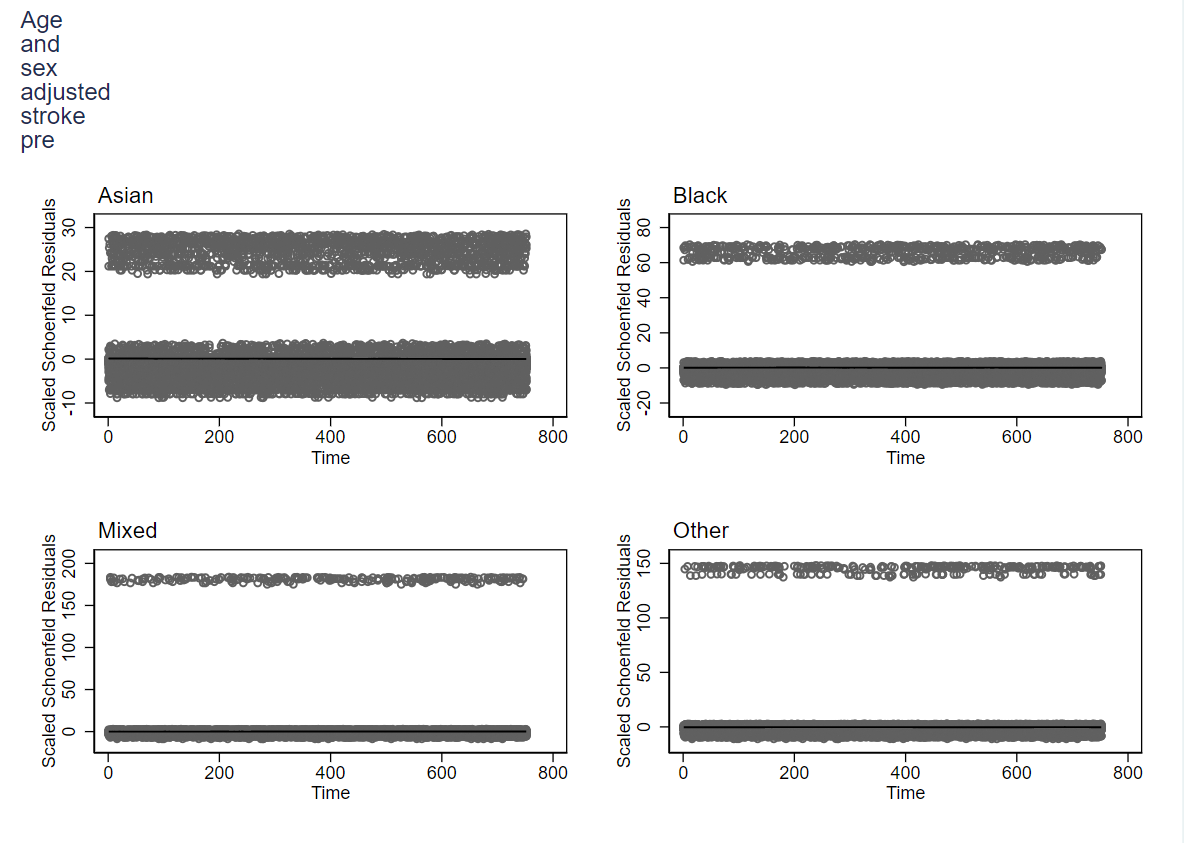


Outcome: Stroke

Time period: Pandemic

Outcome: Stroke

Time period: Pre-pandemic


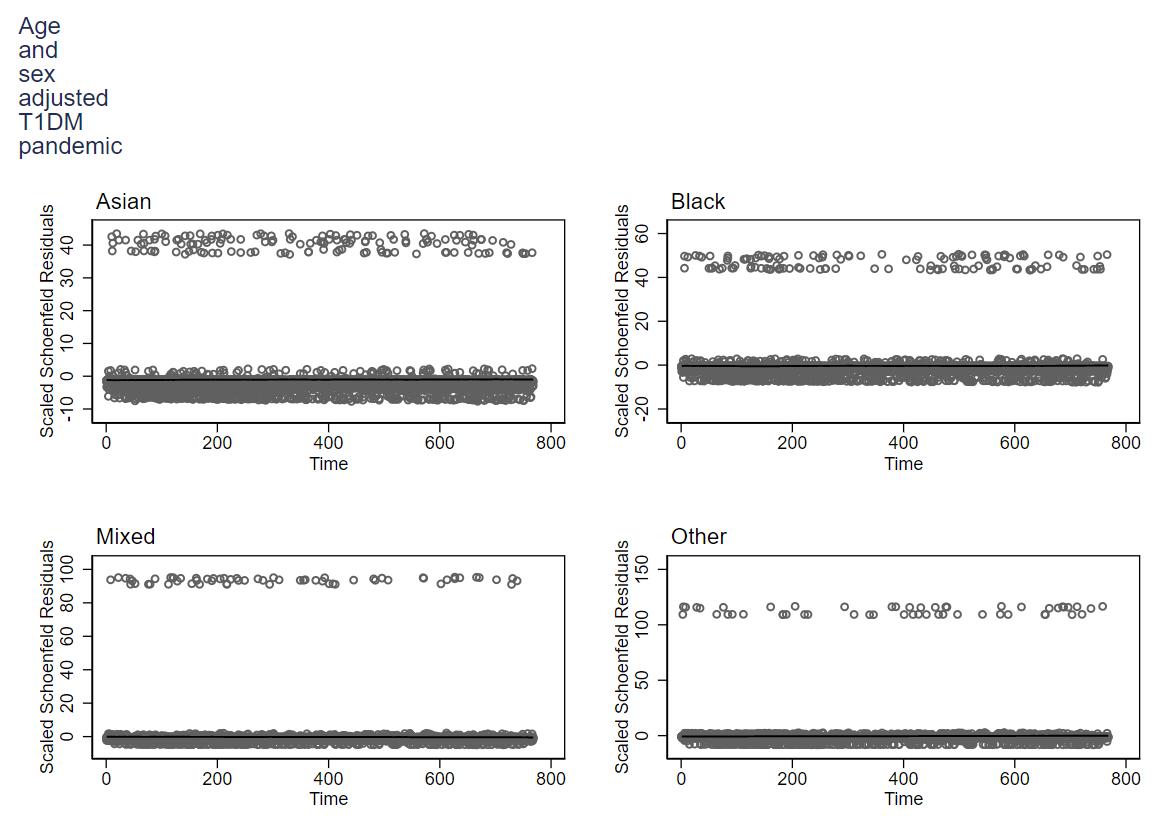


Outcome: Venous thromboembolism

Time period: Pre-pandemic


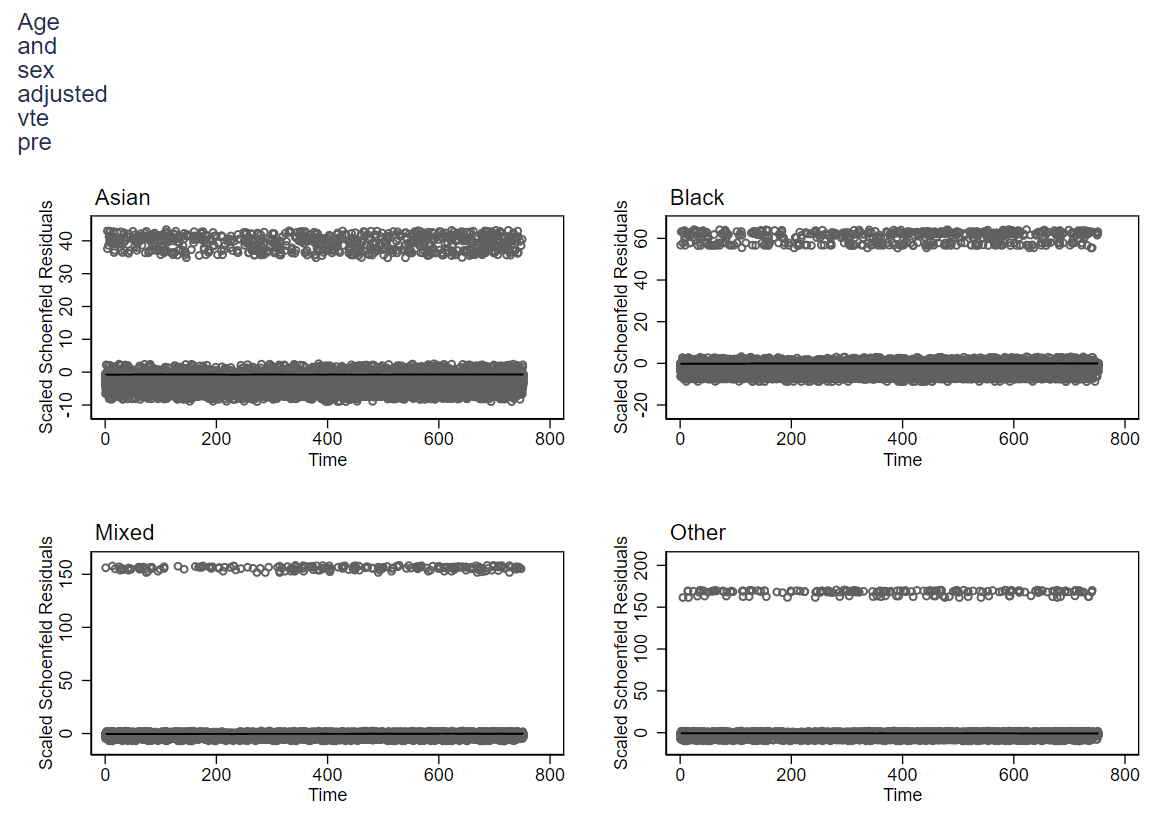


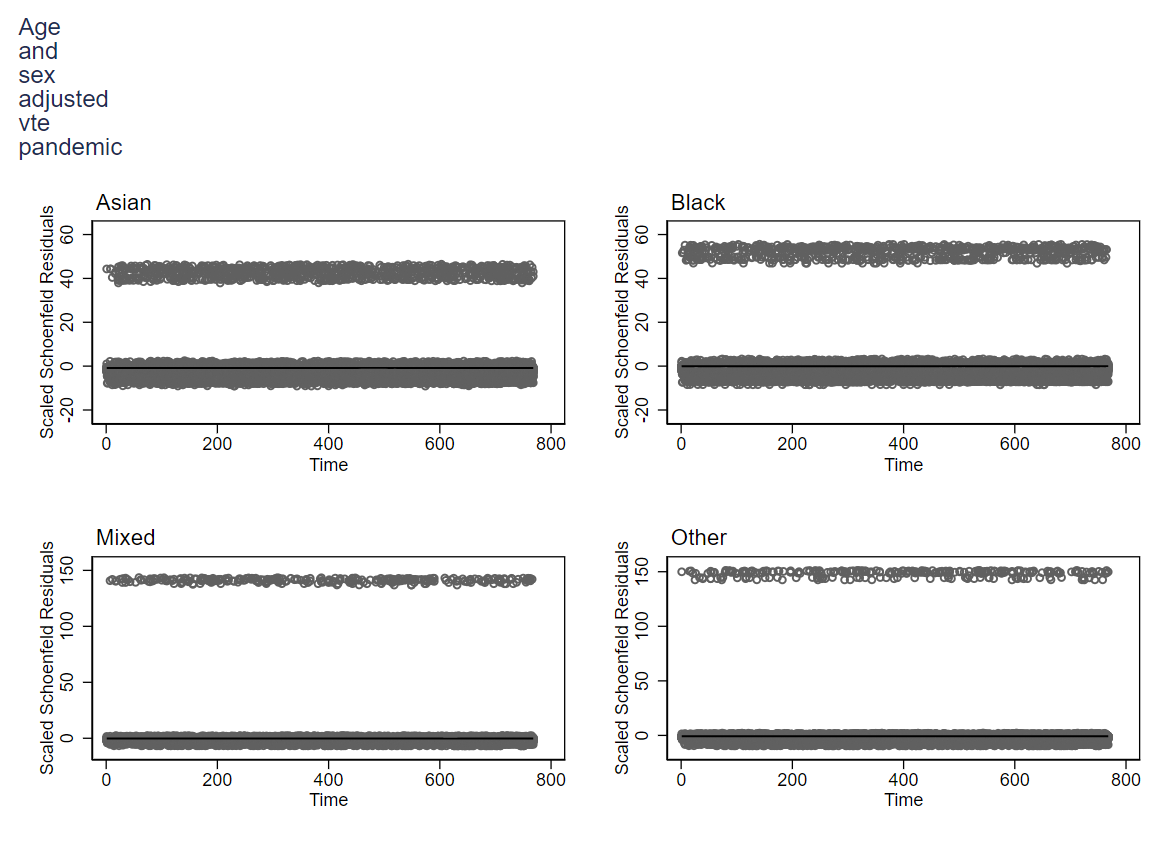


Outcome: Venous thromboembolism

Time period: Pandemic

Outcome: Heart failure

Time period: Pre-pandemic


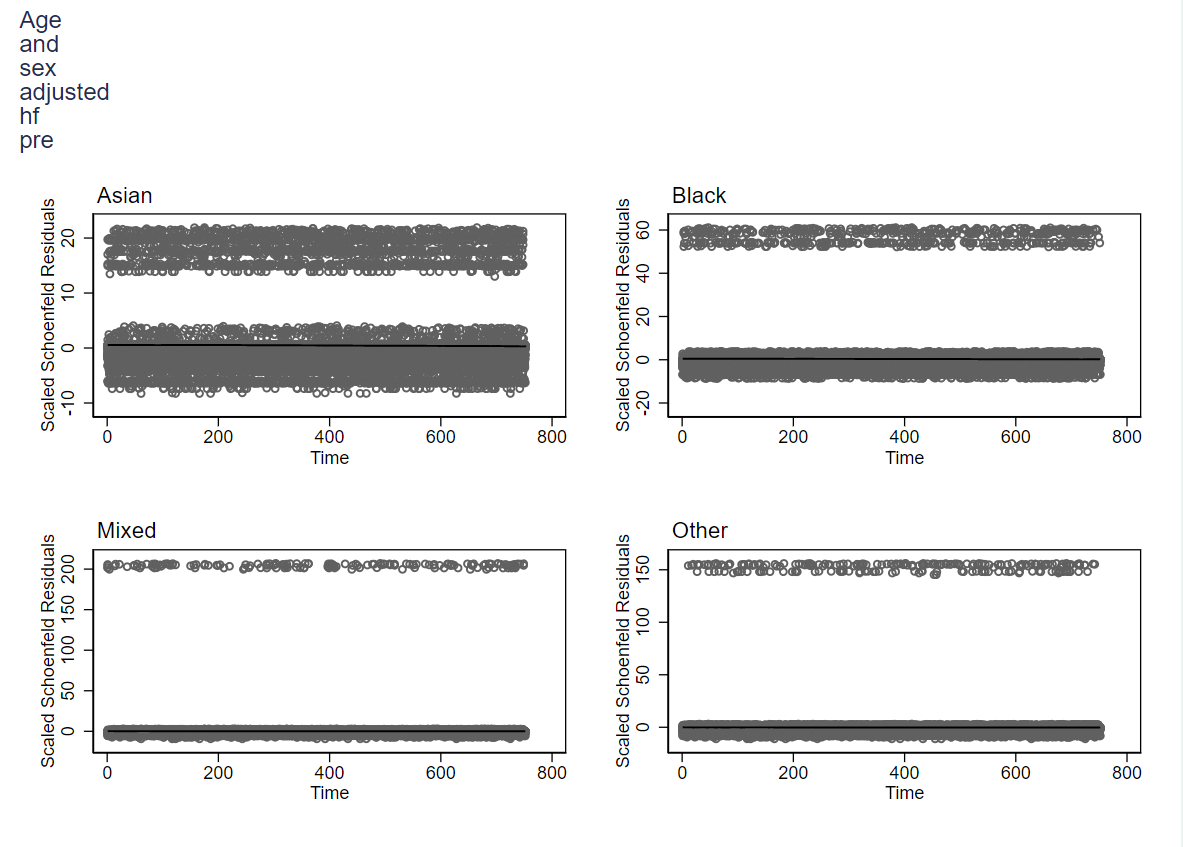


Outcome: Heart failure

Time period: Pandemic


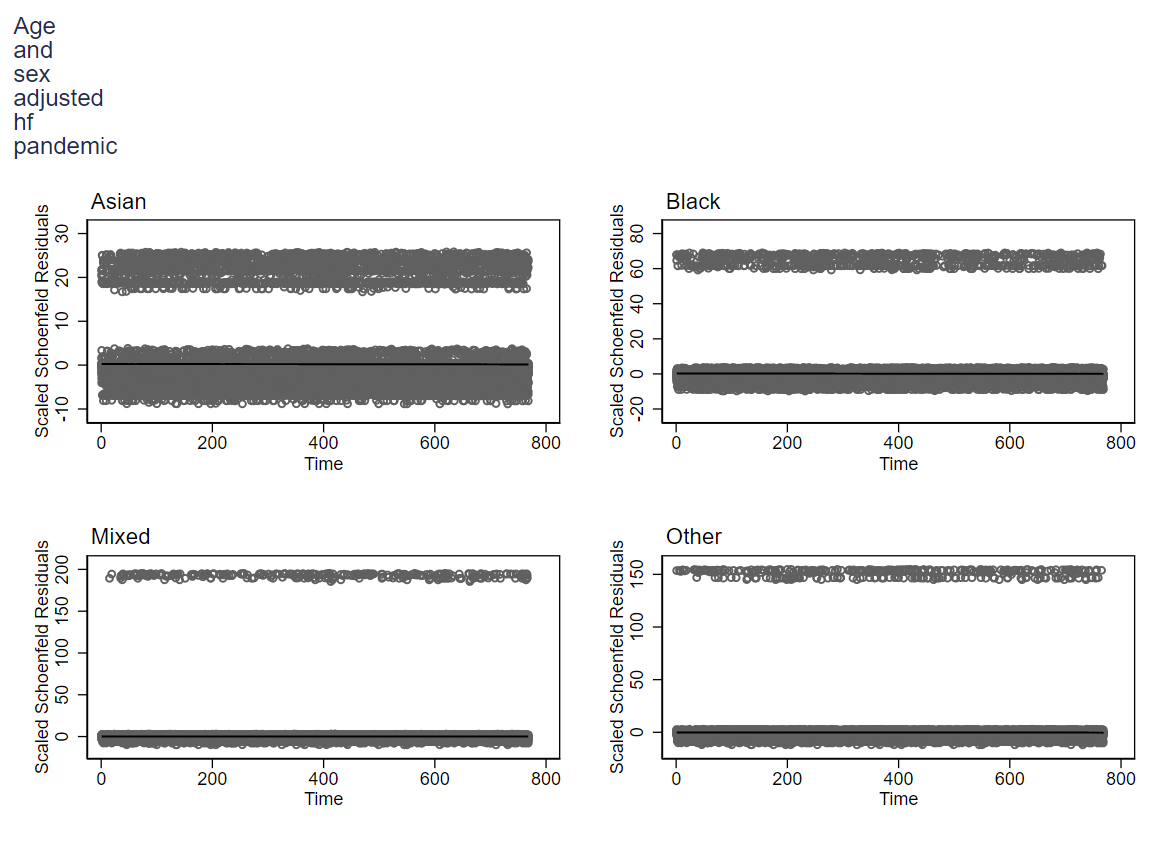


Outcome: Myocardial infarction

Time period: Pre-pandemic


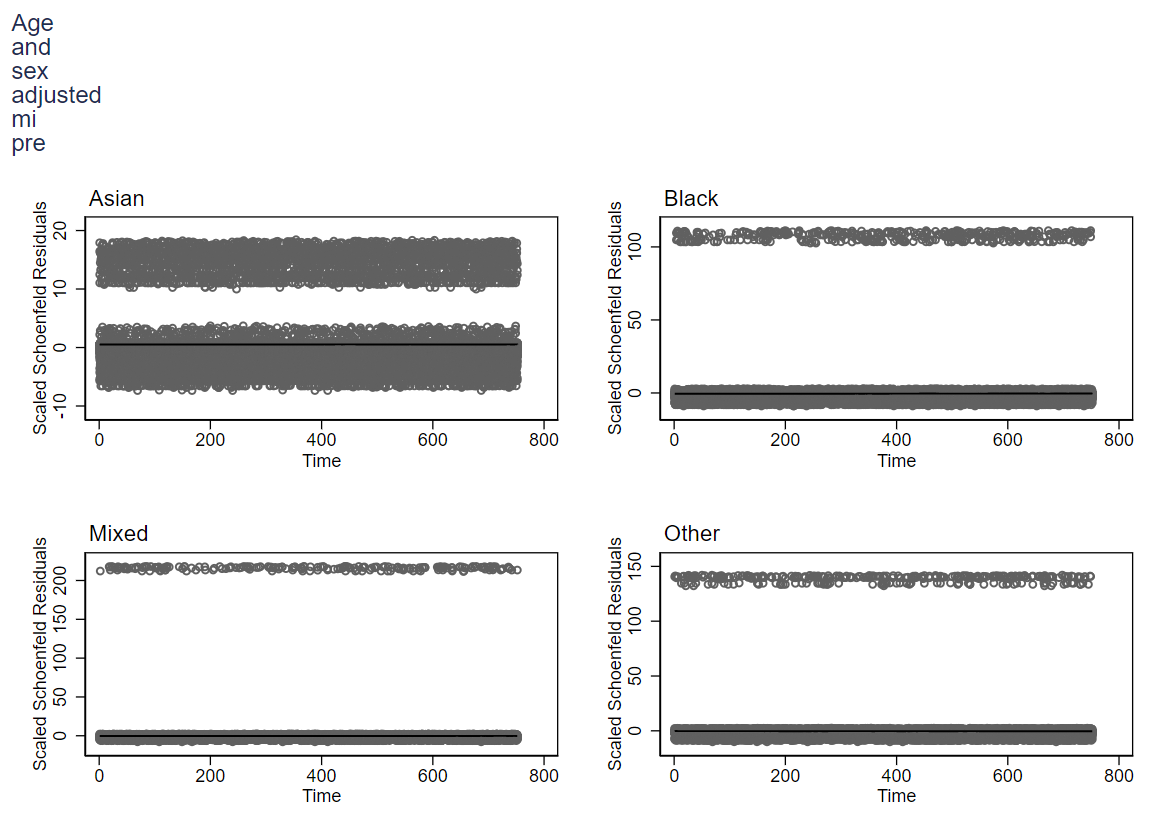


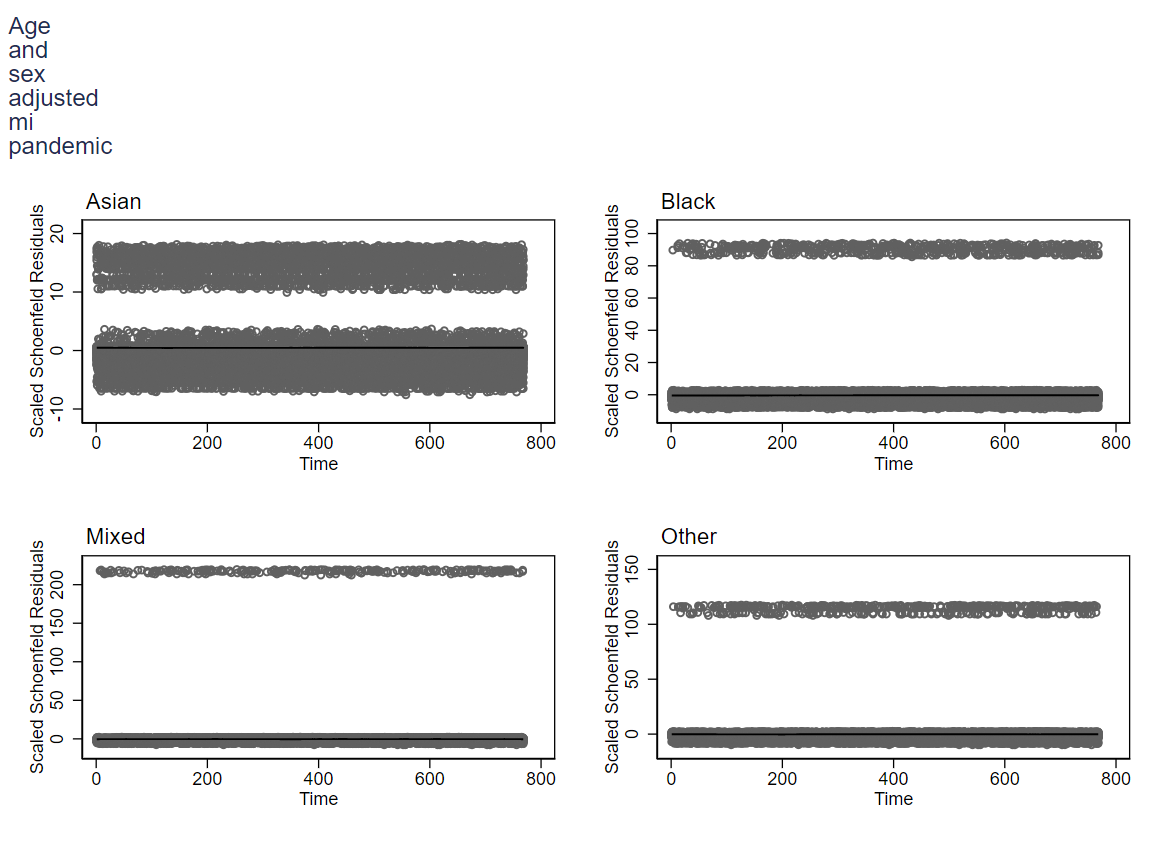


Outcome: Myocardial infarction

Time period: Pandemic


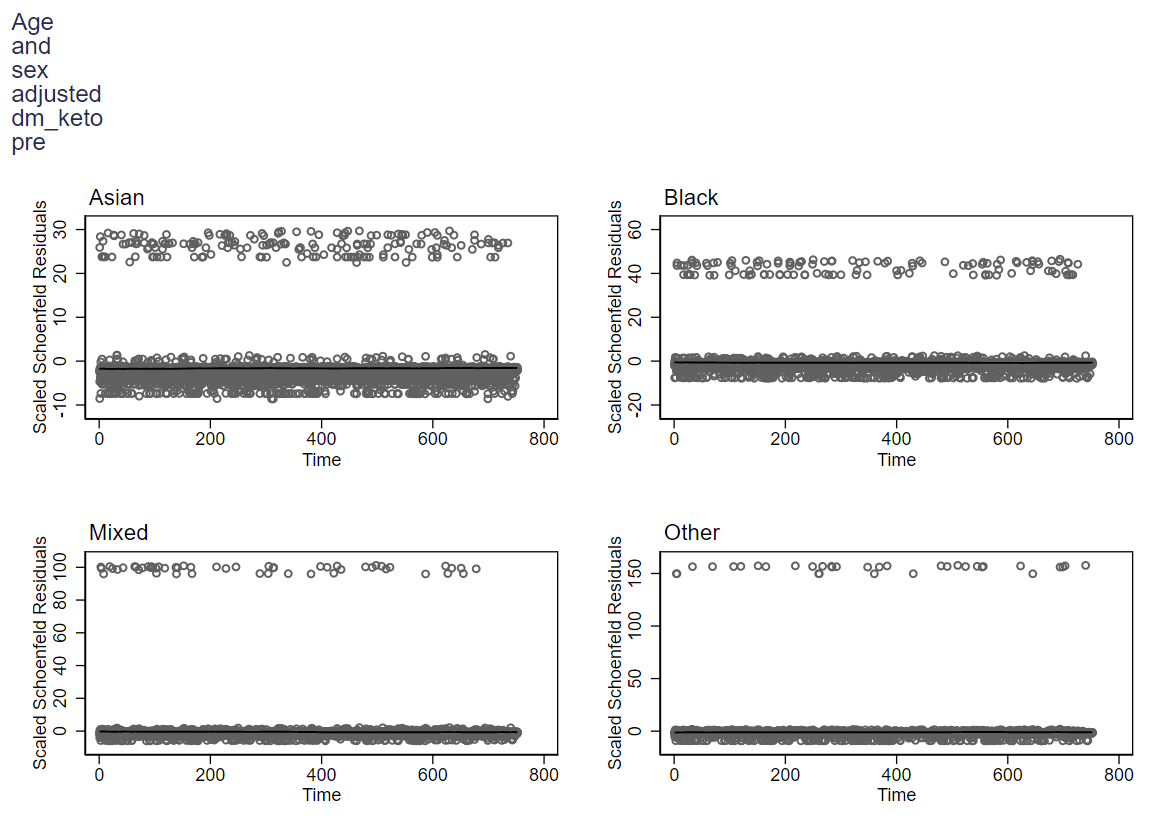


Outcome: Diabetic ketoacidosis

Time period: Pandemic

Outcome: Diabetic ketoacidosis

Time period: Pre-pandemic


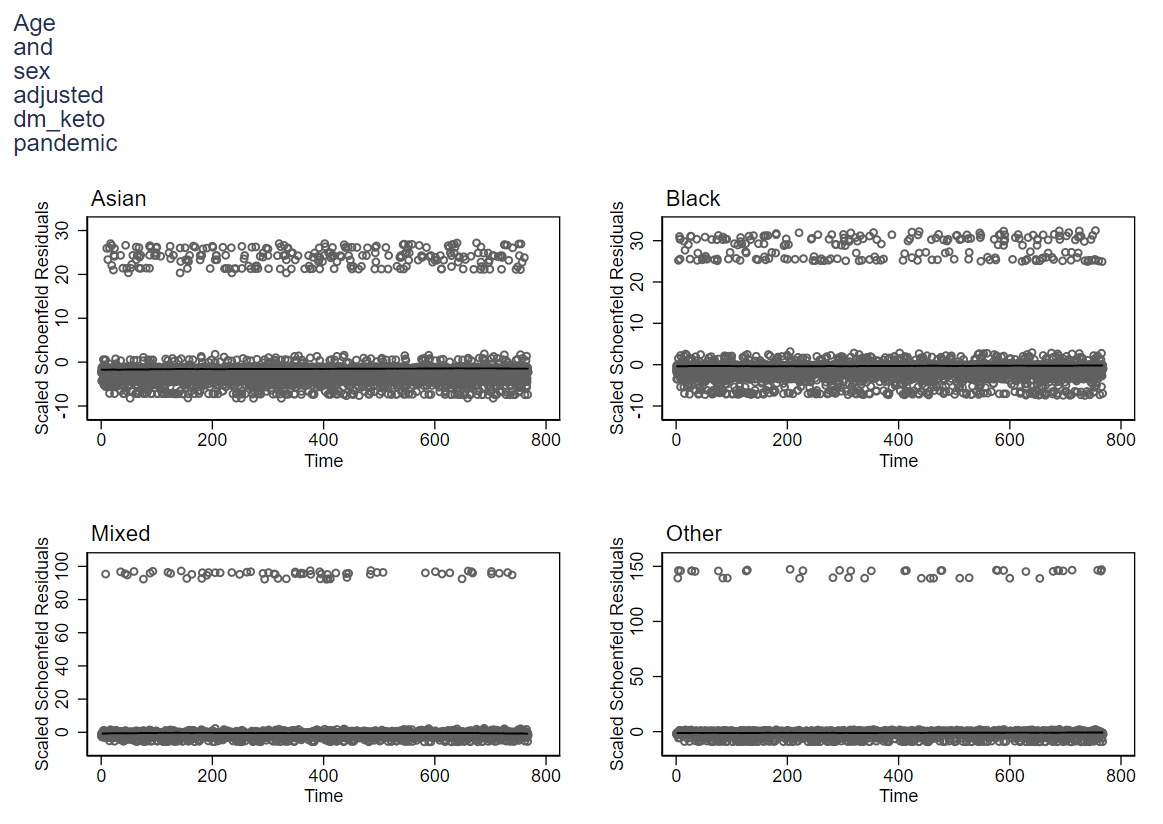


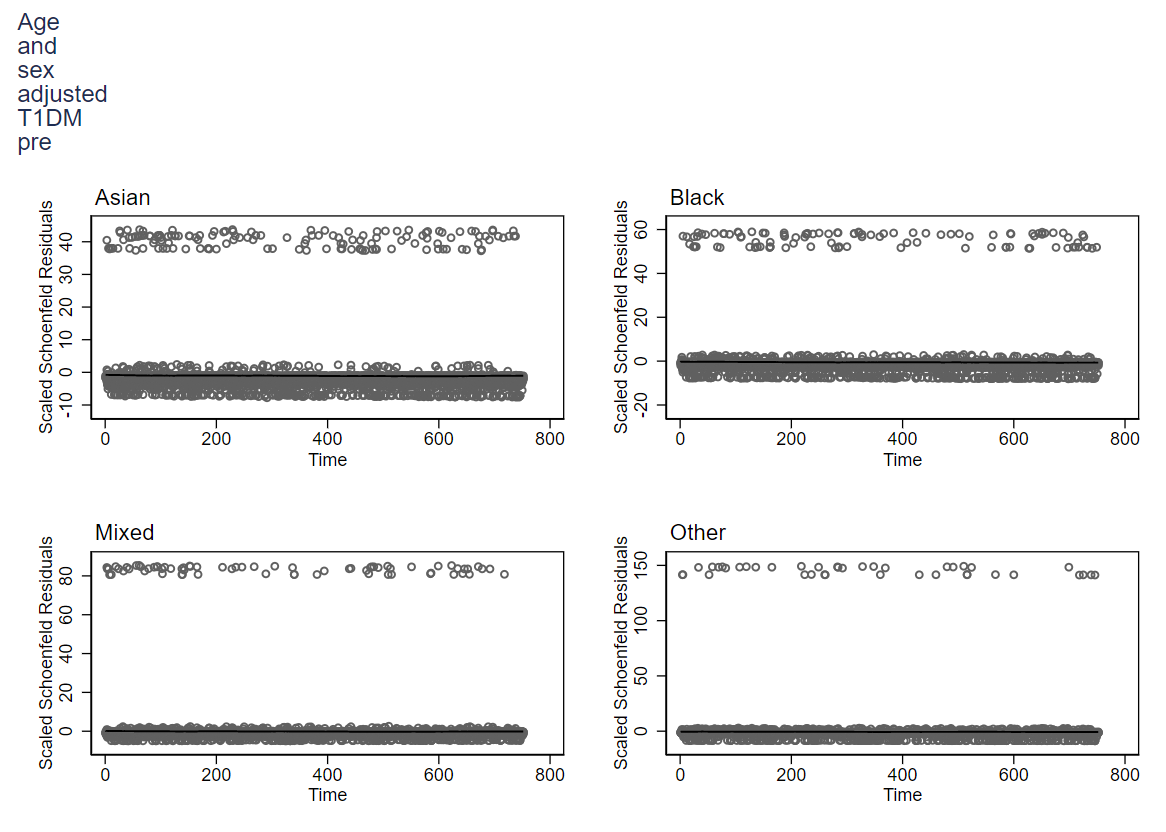


Outcome: Type 1 diabetes

Time period: Pre-pandemic


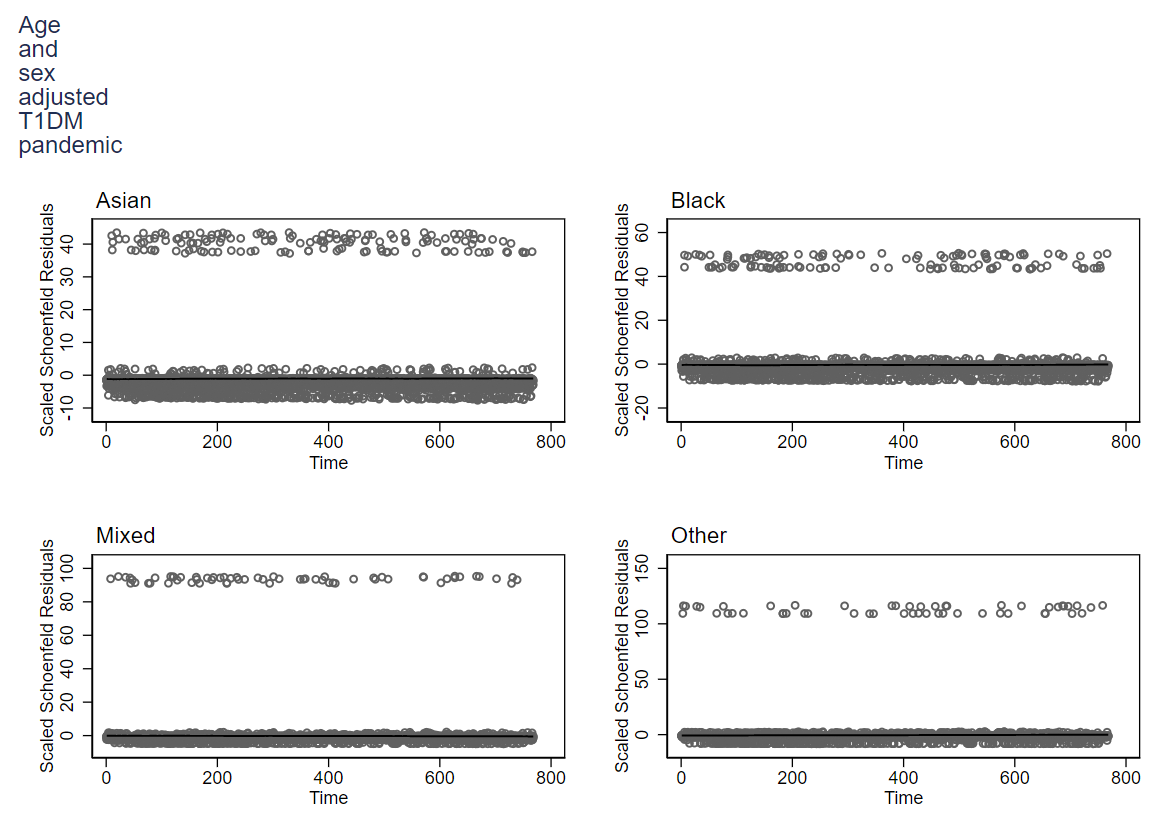


Outcome: Type 1 diabetes

Time period: Pandemic


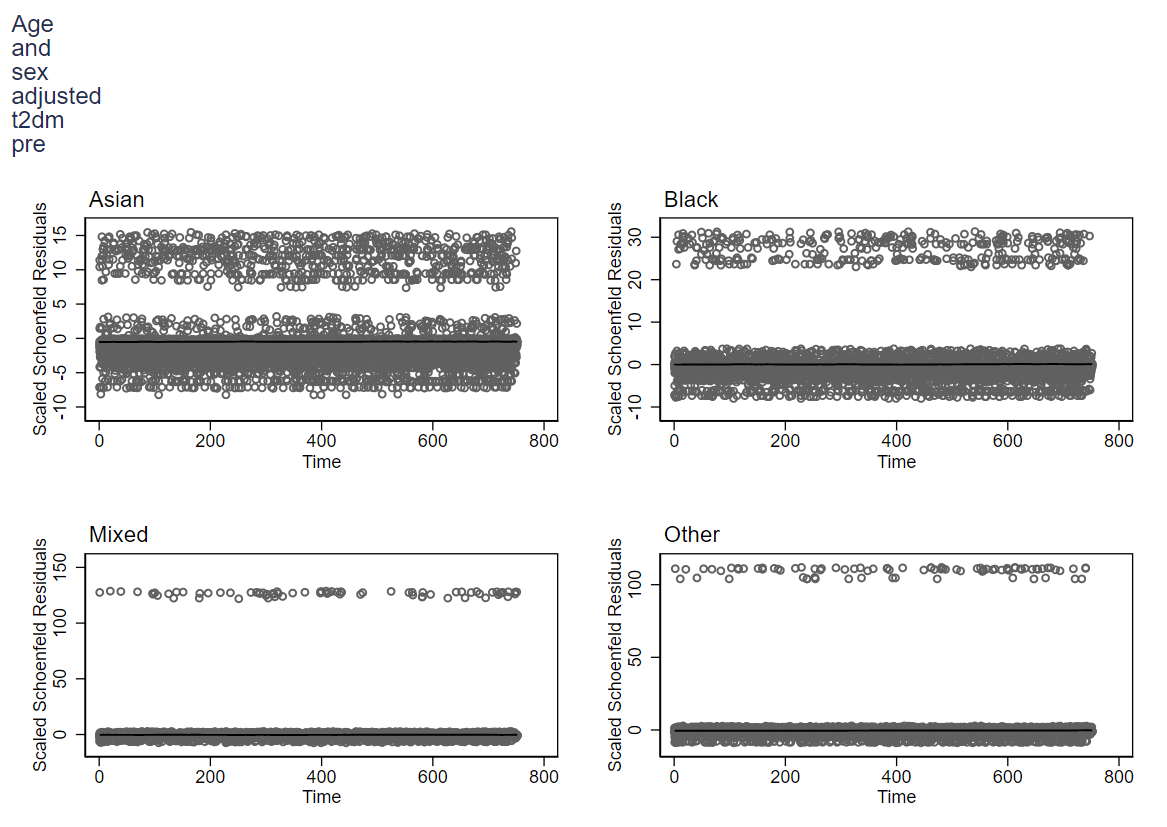


Outcome: Type 2 diabetes

Time period: Pre-pandemic


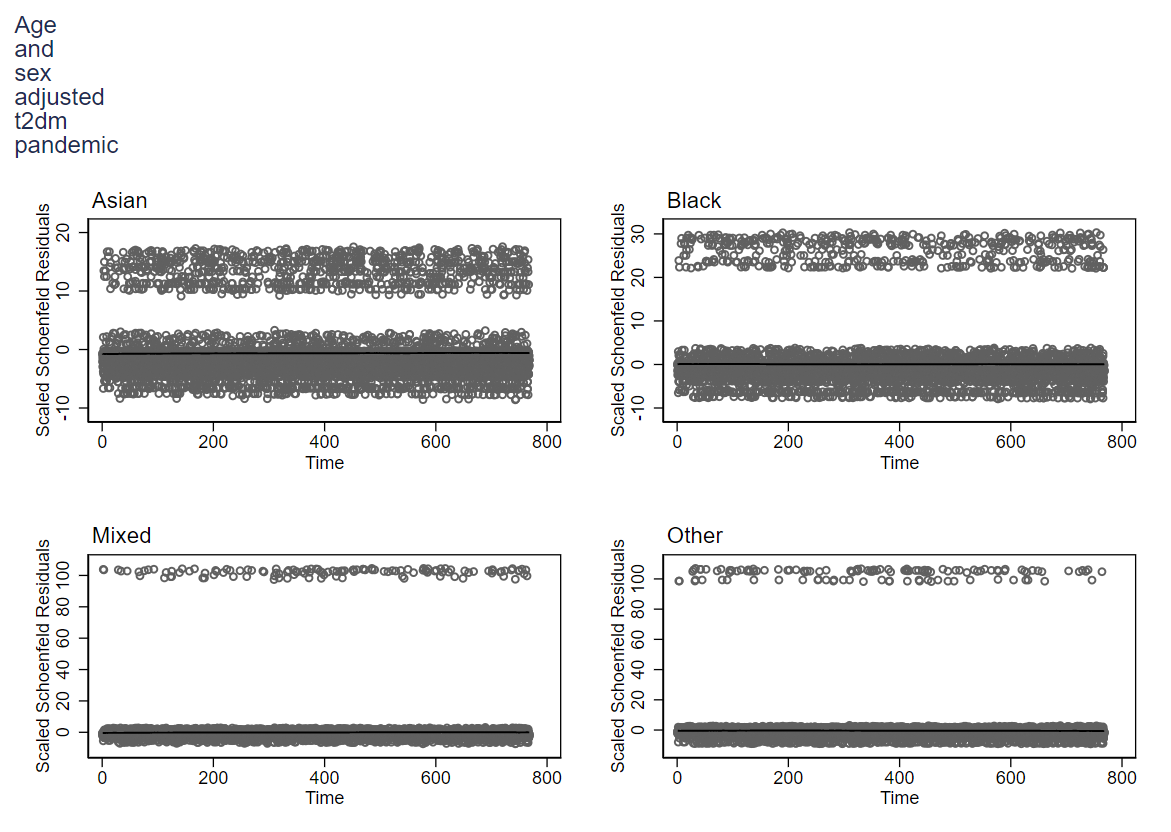


Outcome: Type 2 diabetes

Time period: Pandemic


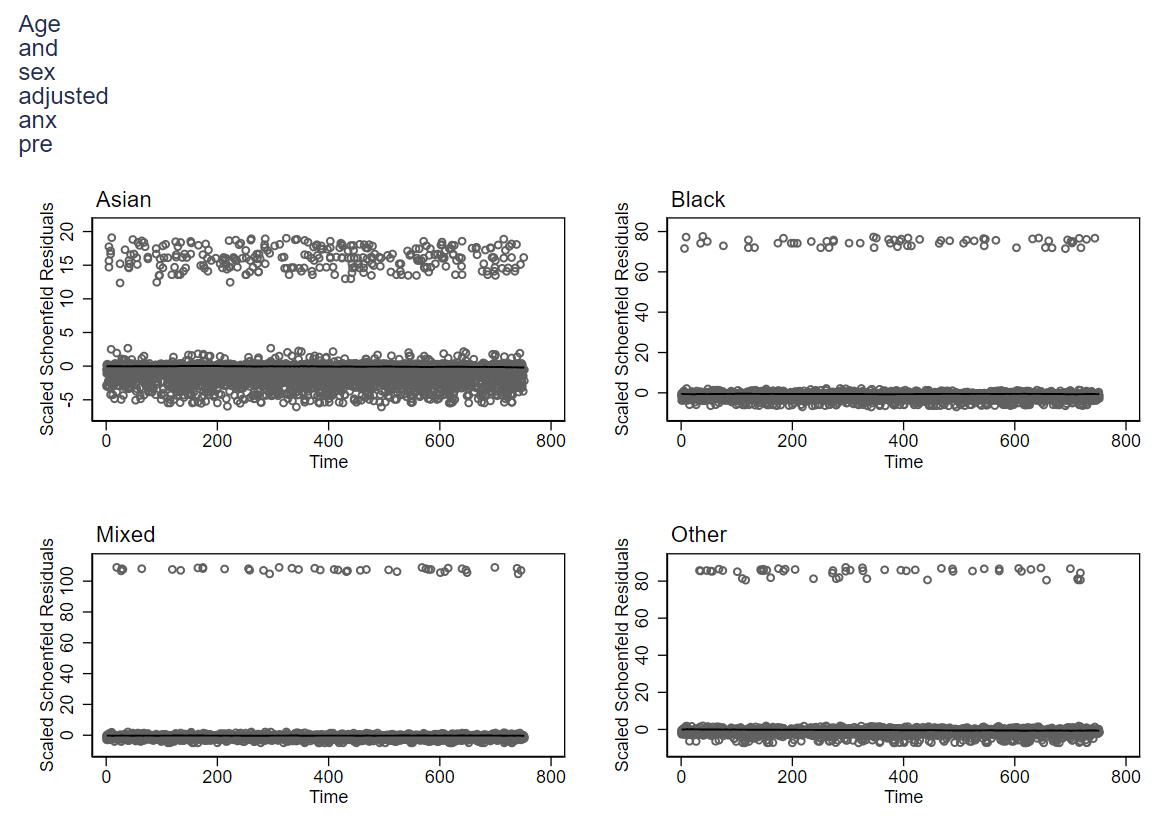


Outcome: Anxiety

Time period: Pre-pandemic


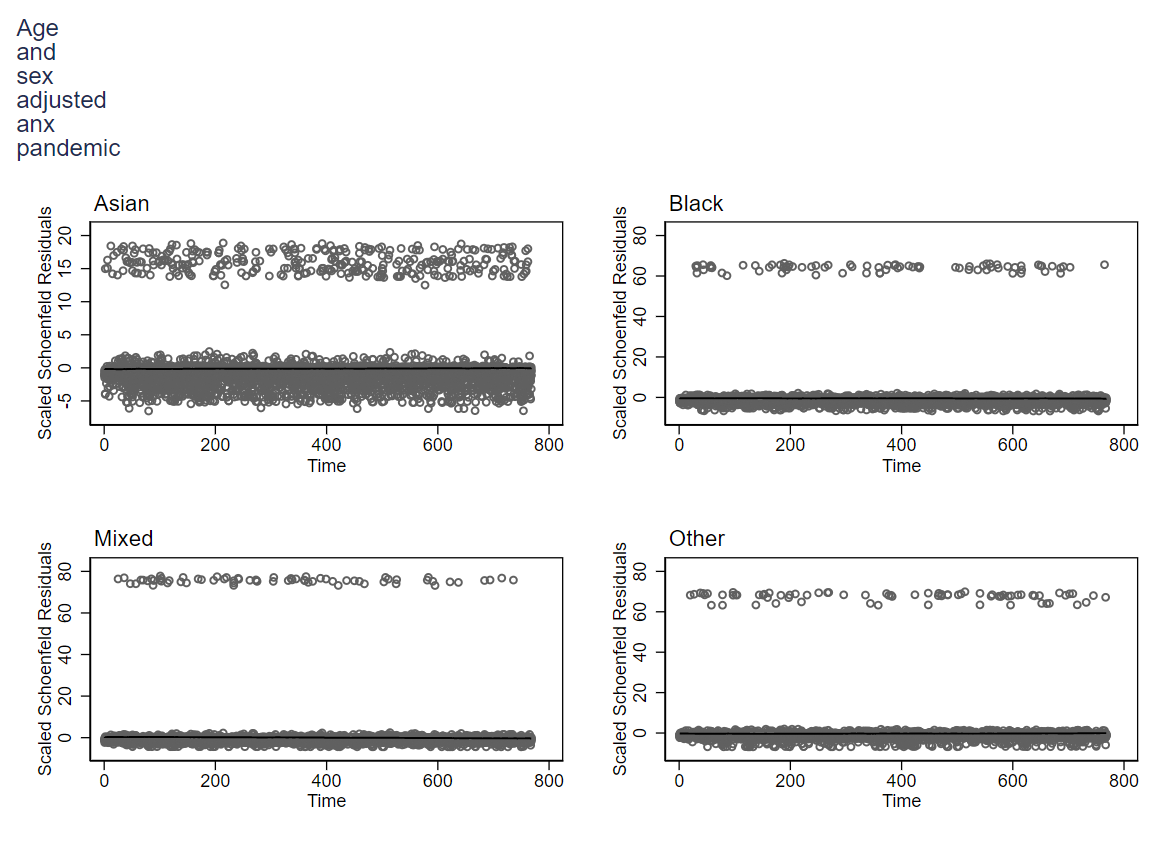


Outcome: Anxiety

Time period: Pandemic


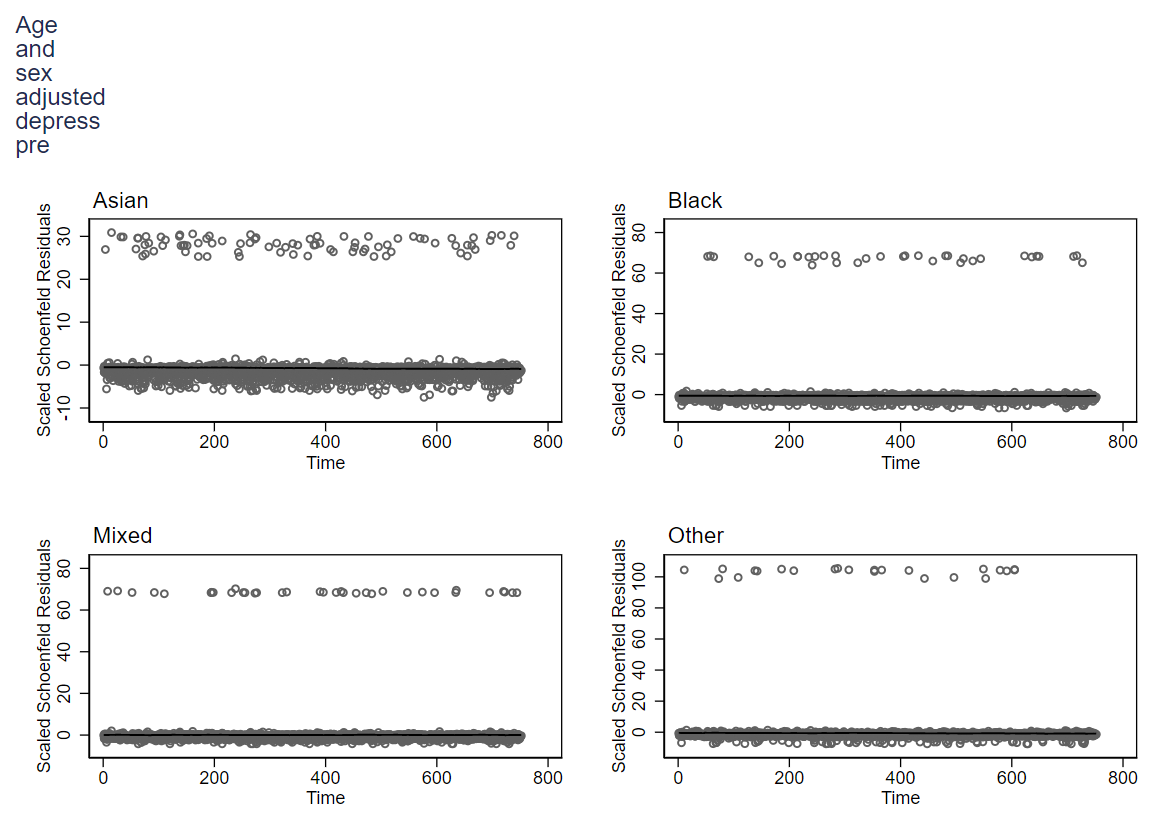


Outcome: Depression

Time period: Pre-pandemic


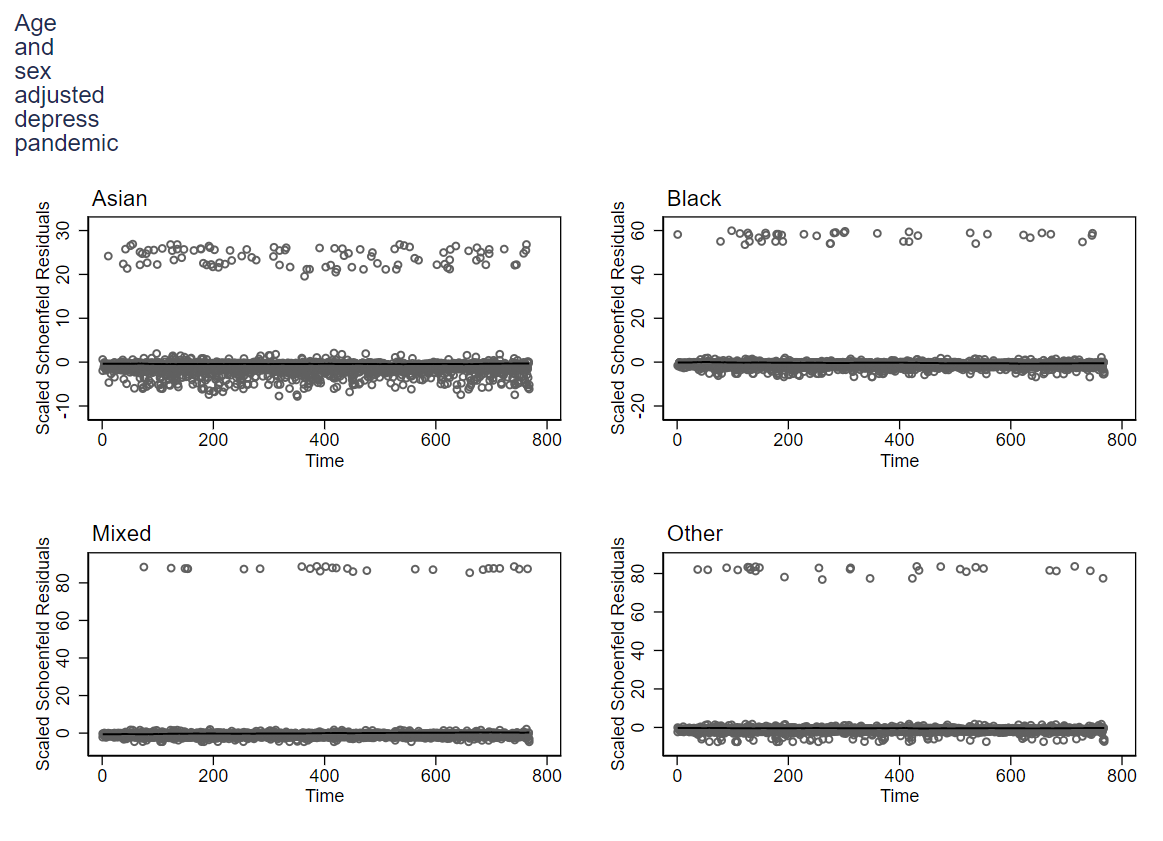


Outcome: Depression

Time period: Pandemic


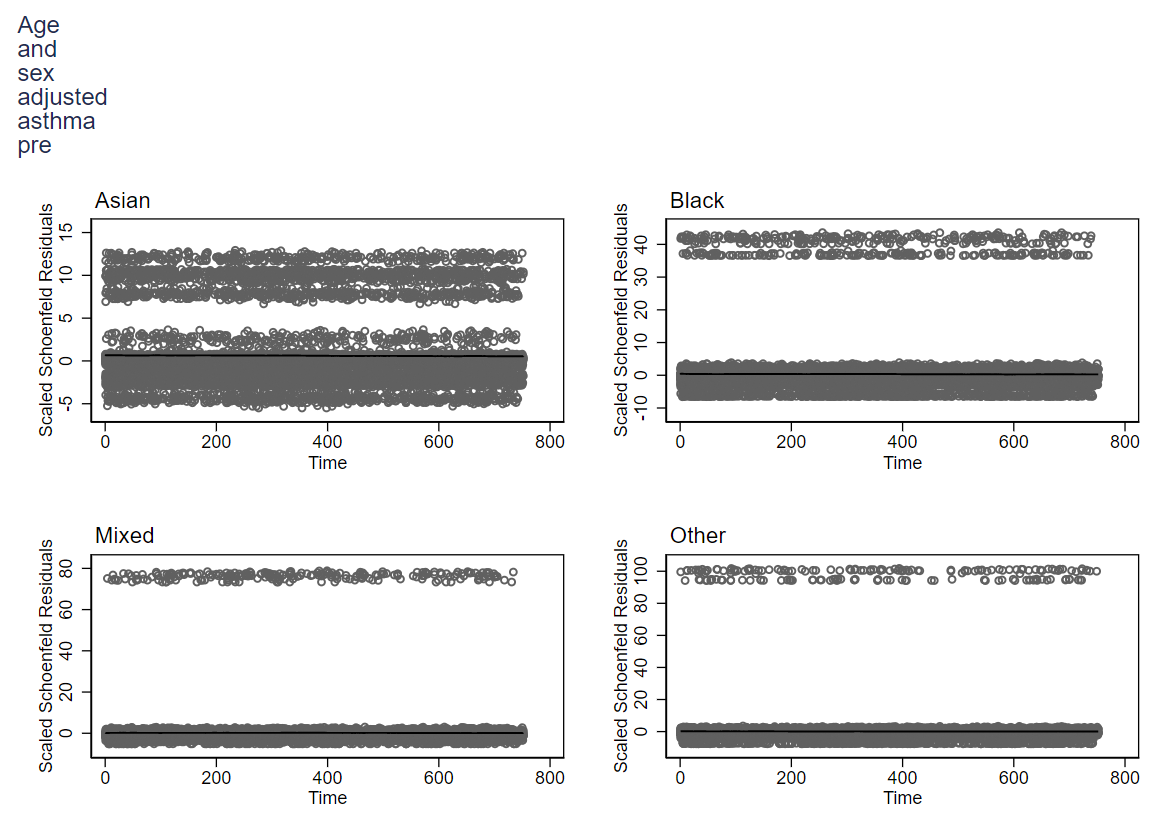


Outcome: Asthma

Time period: Pre-pandemic


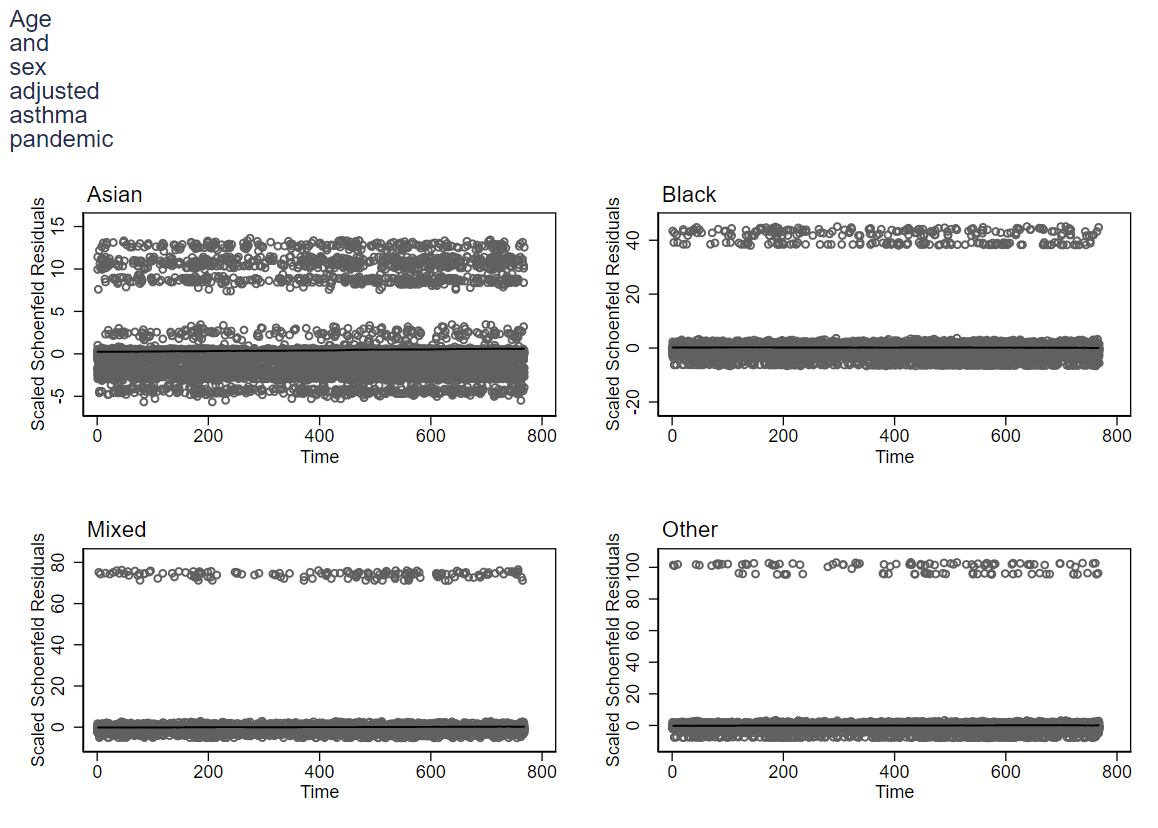


Outcome: Asthma

Time period: Pandemic


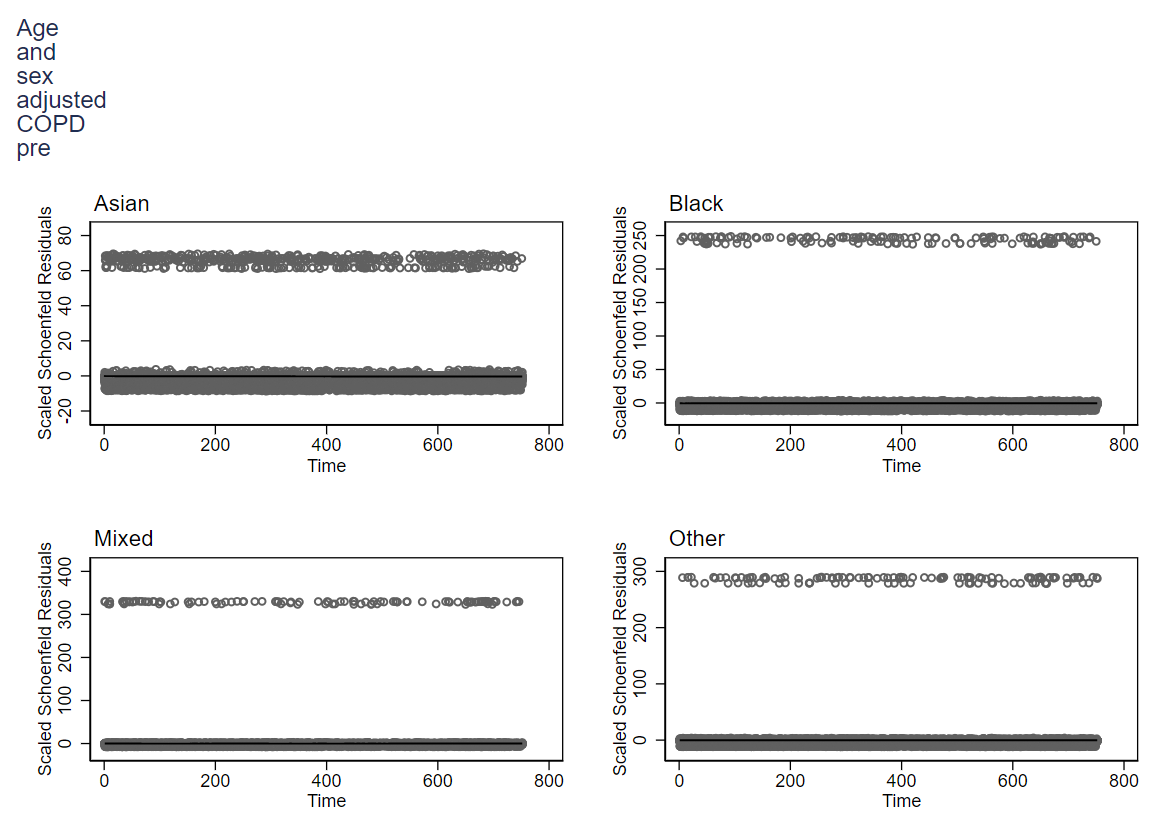


Outcome: Chronic obstructive pulmonary disease

Time period: Pre-pandemic


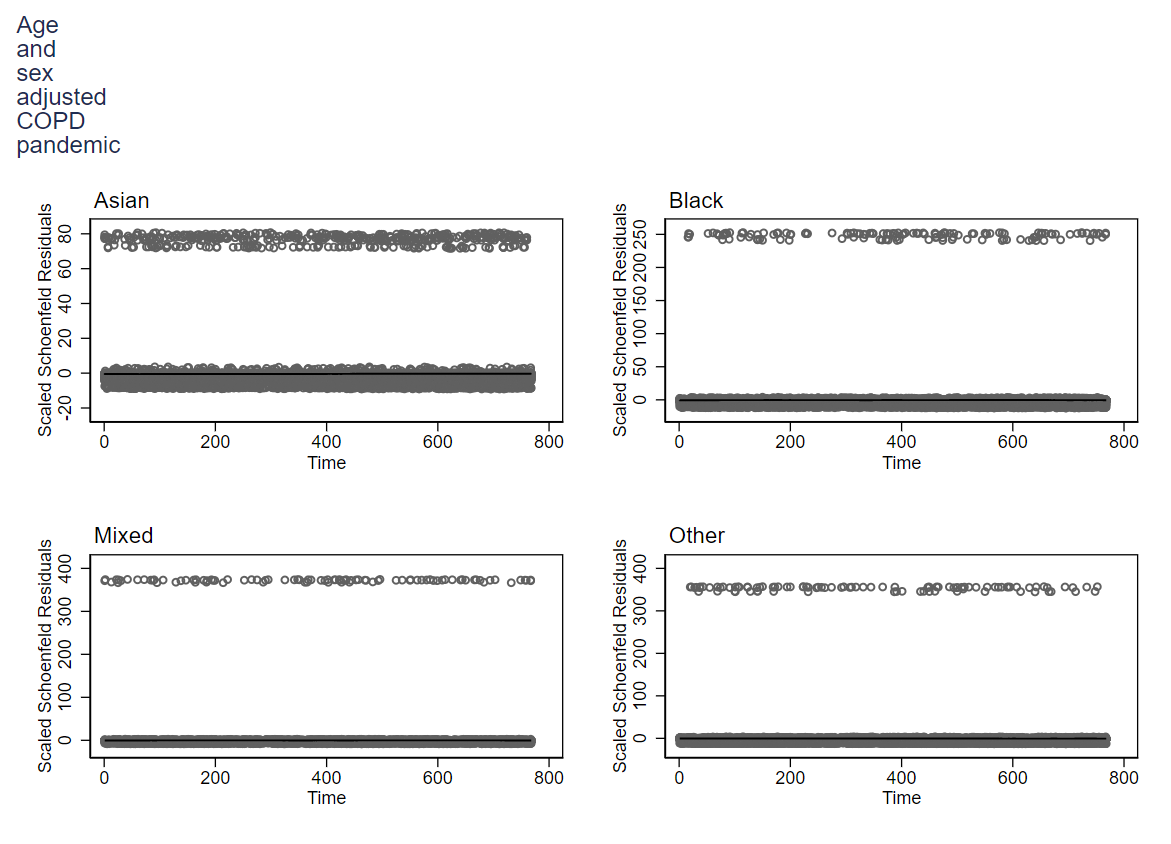


Outcome: Chronic obstructive pulmonary disease

Time period: Pandemic
